# Ligand-Tuned Photophysical Properties and Geometrical Isomerism of Bis-Cyclometalated [Ir(C^N)_2_(CNXyl)X] (X = Cl, C≡CR) Complexes

**DOI:** 10.1021/acs.inorgchem.6c01800

**Published:** 2026-07-29

**Authors:** Andrea Corral-Zorzano, Mónica Martínez-Junquera, Elena Lalinde, M. Teresa Moreno

**Affiliations:** Departamento de Química, Instituto de Investigación en Química (IQUR), Complejo Científico Tecnológico, 16764 Universidad de La Rioja, Madre de Dios 53, 26006 Logroño, Spain

## Abstract

This study reports the synthesis, characterization, and photophysical and electrochemical properties of several bis-cyclometalated Ir­(III) complexes based on the three-ligand molecular framework [Ir­(C^N)_2_(CNXyl)­(C≡CR)] (CNXyl = 2,6-dimethylphenyl isocyanide), varying the cyclometalated chromophore [C^N = 2,4-difluorophenylpyridinate (dfppy) **1**, 2-phenylbenzothiazolate (pbt) **2**, and phenylquinolinate (pq) **3**] and the alkynyl ligand [R = Tol **a**, C_6_H_2_(OMe)_3_ **b**, and pyrene **c**], obtained from the corresponding [Ir­(C^N)_2_(CNXyl)­Cl] (**1**_**Cl**_ **–3**_**Cl**_). By synthetic control using dfppy, four diastereomers of the precursor [Ir­(dfppy)_2_(CNXyl)­Cl] (*N,N-trans* **-1**_**Cl**_, *N,N-cis* **-(** *OC* **-6–24)/(** *OC* **-6–44)**-**1**_**Cl**_ and *C,C-trans* **-1**_**Cl**_) were isolated, enabling access to three geometrical isomers (*N,N-trans* **-1a/1b**, *N,N-cis* **-(** *OC* **-6–32)**-**1a/1b**, and *C,C-trans* **-1a/1b**) of [Ir­(dfppy)_2_(CNXyl)­(C≡CR)] [R = Tol **a**, C_6_H_2_(OMe)_3_ **b**]. Complexes of the ***N,N-trans-*** series (**1a,1b**–**3a,3b**) exhibit blue to orange phosphorescence, depending on the cyclometalated ligand with quantum yields (ϕ) up to 47% in deoxygenated solution and 28.8% in PS films. Experimental and DFT calculations indicate a change in the nature of the low-lying excited state from ^3^ILCT/^3^MLCT in the chloride complexes to ^3^L^′^LCT­(C≡CR→C^∧^N) with some ^3^MLCT for the C≡CR derivatives. Geometrical isomerism has little effect on emission energy, but significantly influences efficiency, with better results for ***N,N-trans*** isomers. The pyrene-substituted alkynyl complexes *N,N-trans* **-1c**–**3c** exhibit three different emission bands arising from ^3^L′_pyr_LCT/^3^MLCT, ^3^IL′_pyr_, and ^1^IL′_pyr_, depending of the cyclometalated ligand and media.

## Graphical abstract

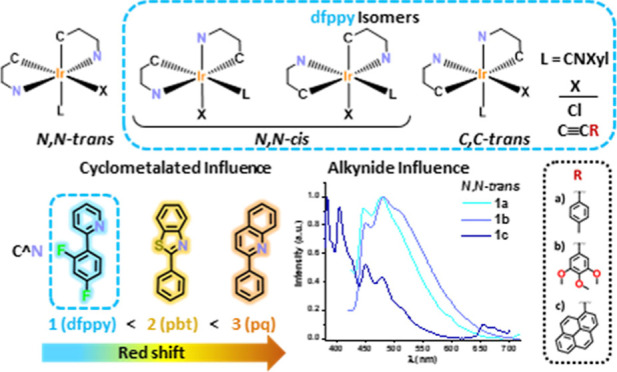

## Introduction

Cyclometalated Ir­(III) complexes are an important class of molecular phosphors that have become prominent in a great number of optical applications including light-emitting devices,^1^ electrochemiluminescence,^2^ luminescent-based sensing,^2a,3^ photocatalysis,^1f,4^ and bioimaging.^3b,3c,5^ Their promising properties are due to their impressive emission quantum yields, short lifetimes, and, particularly, facile color tuneability depending on the electronic and steric properties of the ligands and photochemical stability. It is well-known that the emissive properties of these complexes are primarily determined by both the cyclometalated and ancillary ligands. Their emissions come from ligand-centered (^3^LC), and metal-to-ligand charge transfer (^3^MLCT) mixed excited states, being the nonradiative ^3^MC states to much higher energy and thus depopulated. The incorporation of strong σ-donor coligands is able to further destabilize d–d (^3^MC) states, allowing more efficient phosphorescence from low-lying T_1_ and greater stability.^6^ Generally, in heteroleptic compounds, the triplet energy of the most highly conjugated cyclometalated (HC^N, HC^N′) or auxiliary (X^L, N^N) ligand usually determines the nature of the emissive state (^3^LC, ^3^LL′C, or ^3^ML′CT).

Many works describe bis-cyclometalated (C^N) iridium­(III) complexes with a third chelate or two identical monodentate ligands. Among the latter, several families of cationic bis-cyclometalated Ir­(III) with two strong field electron-withdrawing isocyanide ligands have been described.^7^ The synthetic chemistry of heteroleptic Ir­(III) compounds with two C^N ligands and two different monodentate ligands [Ir­(C^N)_2_LX] is much less explored, although there are examples, including isocyanide/chloride^8^ or isocyanide/cyanide^7a,8a,8c^ complexes. A useful alternative to the cyanide group is the isoelectronic alkynyl (C≡CR) ligand that displays strong σ-donor and moderate π-acceptor ability, participating in metal-to-ligand back bonding. Furthermore, with the C≡C–R ligands, both the σ/π ligand-to-metal and π-metal-to-ligand bonding components can be modulated through the R substituent, thus allowing modifications in the metal–alkynyl interaction being relevant in the optical properties. However, surprisingly, only a few examples of cyclometalated Ir­(III) derivatives bearing alkynyl ligands have been published.^9^

Heteroleptic complexes are generally prepared using the dichloro-bridged binuclear Ir­(III) complexes [Ir­(C^N)_2_(μ-Cl)]_2_ as precursors.^10^ In almost all cases, the characterized dimers have an *N,N* **-** *trans* geometry,^10a,11^ and the subsequent reaction with the corresponding coligands retains the *N,N* **-** *trans* arrangement of the C^N ligands, although, considering the multiple *C,C* **/** *N,N-cis* **/** *trans* disposition of the C^N ligands, several stereoisomers can exist. However, synthetic procedures to obtain *N,N* **-** *cis* complexes have rarely been described, and complexes with these arrangements are quite limited. Particularly for luminescent *N,N* **-** *cis* isomers, only a few examples have been reported.^12^ For some homo and hetero tris-cyclometalated complexes of type [Ir­(C^N)_2_(C′^N′)]/[Ir­(C^N)_3_], thermal or photochemical isomerization from *mer* to *fac* isomers has been reported,^13^ showing that the *fac* isomer usually exhibits a higher emission efficiency than the corresponding *mer*-isomer, a fact of great relevance for different photophysical applications.^13f,14^ Isomerization in neutral [Ir­(C^N)_2_(L^X)][Bibr ref15] and cationic [Ir­(C^N)_2_(L^L′)]^+^ ^12a–12c^ complexes has also been described. In comparison, the exploration of geometrical isomerism in neutral bis-heteroleptic [Ir­(C^N)_2_LX] remains very limited. Notably, under adequate thermal conditions, *N,N* **-** *trans* [Ir­(C^N)_2_ClL] complexes featuring L = diphenyl­(1-naphthyl) or (benzyldiphenyl)­phosphine ligands initially evolve to the corresponding *N,N* **-** *cis* isomers, which subsequently activate the naphthyl or benzyl groups of the coordinated phosphine to afford *N,N* **-** *cis* [Ir­(C^N)_2_C^P].^12d,16^

In this work, we report a series of bis-cyclometalated Ir­(III) complexes with a three-ligand molecular framework [Ir­(C^N)_2_(CNXyl)­(C≡CR)] from the corresponding [Ir­(C^N)_2_(CNXyl)­Cl] complexes (CNXyl = 2,6-dimethylphenyl isocyanide) varying the cyclometalated [C^N=C-deprotonated 2,4-difluorophenylpyridine (dfppy) **1**; 2-phenylbenzothiazol (pbt) **2**; and phenylquinoline (pq) **3**] and the alkynyl-chromophore with tolyl (Tol) (**a**), 1,2,3-trimethoxy-benzene [C_6_H_2_(OMe)_3_] (**b**), or pyrene (**c**) substituents. As expected, the *N,N* **-** *trans* isomers were synthesized. Interestingly, in the case of dfppy complexes, we have been able to isolate four of the six possible diastereomers (see Chart 1) of [Ir­(dfppy)_2_(CNXyl)­Cl], depending on the reaction conditions, allowing us to prepare related diastereomers for [Ir­(dfppy)_2_(CNXyl)­(C≡CR)] [R = Tol **1a**, C_6_H_2_(OMe)_3_ **1b**]. The influence of each ligand and the effect of the isomerization on the photophysical properties have been investigated with the support of TD-DFT and DFT theoretical calculations on selected species.

**Chart 1:**
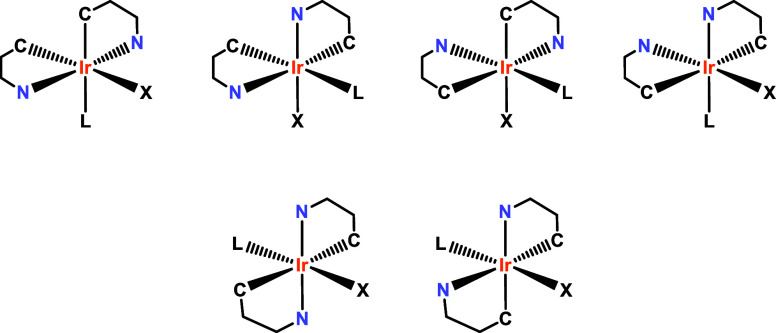
Possible Diastereomers of the Proposed Ir­(III) Complexes [Ir­(C^N)_2_LX].

## Results and Discussion

### Synthesis of Complexes

The synthesis of these target Ir­(III) complexes involves three different cyclometalated groups and three alkynyl ligands in two-step reactions. First, the treatment of the corresponding cyclometalated Ir­(III) μ-chloro-bridged dimer^10b,17^ with the CNXyl ligand gives [Ir­(C^N)_2_(CNXyl)­Cl] complexes, and in the second step, the replacement of the Cl ligand with the corresponding alkynyl ligand gives the desired [Ir­(C^N)_2_(CNXyl)­(C≡CR)] derivatives (Scheme 1). For the synthesis of the starting dimers [Ir­(C^N)_2_(μ-Cl)]_2_, we followed the classic Nonoyama procedure,^10b,17^ involving the reaction of IrCl_3_·3H_2_O and ∼ 2.2 equiv of the corresponding HC^N ligand in a 3:1 (v/v) mixture of 2-ethoxyethanol:H_2_O at reflux for 20 h. The complexes [Ir­(C^N)_2_(CNXyl)­Cl] (C^N = pbt **2**_**Cl**_,^8b^ pq **3**_**Cl**_) were obtained from the corresponding [Ir­(C^N)_2_(μ-Cl)]_2_ by the reaction with 2 equiv of CNXyl, as the ***N,N-trans*** isomers. Complex *N,N-trans*-[Ir­(dfppy)_2_(CNXyl)­Cl] (**1**_**Cl**_) has been previously reported^8a^ following a similar synthesis, but with subsequent purification by a chromatographic column. The reaction of our [Ir­(dfppy)_2_(μ-Cl)]_2_ with CNXyl in CH_2_Cl_2_ resulted in a mixture of two isomers, *N,N-trans* **-1**_**Cl**_ and *N,N-cis* **-(** *OC* **-6–24)-1**_**Cl**_ (with the Cl *trans* to N), which were separated by a chromatographic column [silica, CH_2_Cl_2_:MeOH (95:5)] with final isolated yields of 40 and 23%, respectively (Scheme 1a). The formation of both isomers is not surprising and can be attributed to the presence of a mixture of diastereomers (*C,C-cis*; *N,N*-*trans* and *C,C*-*cis*; *N,N*-*cis*) in the dimer precursor [Ir­(dfppy)_2_(μ-Cl)]_2_, as it has been previously reported.^12c^ In a parallel project aiming to prepare carbazolate/isocyanide derivatives, we examined the reaction of *N,N-trans* **-1**_**Cl**_ with lithium carbazolate (formed in situ with carbazole and *n*-butyllithium at −30 °C) in THF under reflux for 7 h (see the Experimental Section). To our surprise, this reaction evolves with isomerization of complex *N,N-trans* **-1**_**Cl**_ giving rise to a new *cis*-configured isomer *N,N-cis* **-(** *OC* **-6–44)-1**_**Cl**_ with the Cl *trans* to C (Scheme 1b). We checked the stability of these isomers, under reflux in 1,2-dichloroethane for 10 h, and no isomerization between them was observed, indicating that these isomers are thermally stable.

**Scheme 1:**
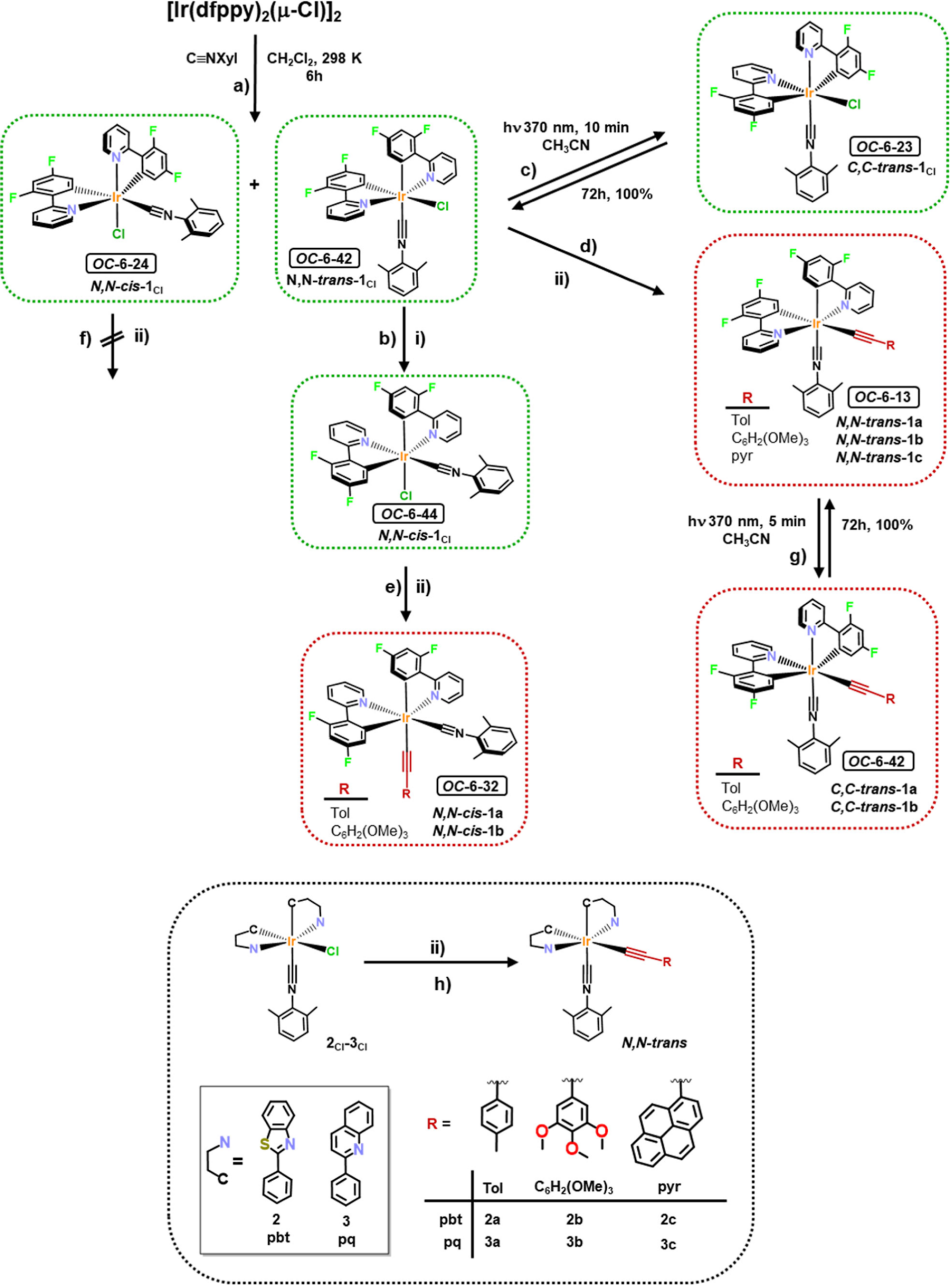
Synthesis of the Complexes^a^

a Reagents and conditions: (i) ^n^BuLi, carbazole, THF, reflux 7 h; (ii) Ag­(C≡CR) 1.1 equiv, 1,2-dichloroethane (reflux **1a**–**c**, **2a**–**c**; 298 K **3a**–**c**).

According to the literature, fluorine-substituted cyclometalated Ir complexes can isomerize upon photoirradiation because the energy level of the phosphorescent state is close to that of the d–d excited state.^12b,12c^ We discovered that isomer *N,N-trans* **-1**_**Cl**_ is photoisomerizable. Thus, a CD_3_CN solution of *N,N-trans* **-1**_**Cl**_ was photoirradiated using a Kessil lamp (370 nm, 40 W) for 5 min, resulting in the appearance of a new pattern in the ^1^H and ^19^F NMR spectra having two sets of new dfppy signals, which, therefore, correspond to the fourth and more rare asymmetrical isomer with the cyclometalated carbons mutually trans (*C,C-trans* **-1**_**Cl**_) (Scheme 1c). Curiously, no similar photoisomerization takes place in CDCl_3_, pointing to the key role of the donor CH_3_CN solvent in the photoisomerization process. Not unexpectedly, this new isomer having a configuration with the two strong C_dfppy_ donors in *trans* disposition is not stable and spontaneously reverts to the isomer *N,N-trans* **-1**_**Cl**_, as confirmed by NMR. Thus, upon complete photoformation of *C,C-trans* **-1**_**Cl**_, the solution was monitored by ^19^F NMR, showing that this isomer reverted on standing at 298 K to the *N,N-trans* **-1**_**Cl**_ in a 14% after 7 h, reaching the 38% after 22 h and, finally, after 3 days on standing, the reversion was complete. Unfortunately, the photoisomerization of the *N,N-cis* **-1**_**Cl**_ isomers (*OC* **-6–44**) or (*OC* **-6–24**) in CH_3_CN, under the same conditions, resulted in a complex mixture of species that were difficult to assign unambiguously.

According to the computational data, the geometry optimizations of the four isomers of **1**_**Cl**_ confirm that *C,C-trans* *-* **1**_**Cl**_ is the least stable and *N,N-cis* **-(** *OC* **-6–44)-1**_**Cl**_, obtained under reflux conditions, is the most stable. Typical *N,N-trans-* **1**_**Cl**_ is more stable than *N,N-cis* **-(** *OC* **-6–24)-1**_**Cl**_ by 5.96 kcal/mol (Figure S1). Therefore, the order of stability of all four isomers is *N,N-cis* **-(** *OC* **-6–44)-1**_**Cl**_ > *N,N-trans* **-1**_**Cl**_ > *N,N-cis* **-(** *OC* **-6–24)-1**_**Cl**_ ≫ *C,C-trans-* **1**_**Cl**_.

The substitution of chloride by selected alkynyls was carried out using as precursors the three stable isomers of dfppy system **1**_**Cl**_ [*N,N-trans* **-1**_**Cl**_, *N,N-cis* **-(** *OC* **-6–24)-1**_**Cl**_, and *N,N-cis* **-(** *OC* **-6–44)-1**_**Cl**_], **2**_**Cl**_, and **3**_**Cl**_. These precursors were treated with AgC≡CR in refluxing 1,2-dichloroethane. The reactions and final products, which are detailed in Scheme 1, confirm that the substitution reactions take place keeping the geometry of the precursors. By starting from dfppy *N,N-trans* **- 1**_**Cl**_, the reactions resulted in the formation of ***N,N-trans***-[Ir­(dfppy)_2_(CNXyl)­(C≡CR)] (R = Tol *N,N-trans*-**1a**, C_6_H_2_(OMe)_3_ *N,N-trans* **-1b**, and pyrene *N,N-trans* **-1c)**, complexes (Scheme 1d). From the ***N,N-cis*** isomers of **1**_**Cl**_, we obtained the *N,N-cis* **-(** *OC* **-6–32)**-[Ir­(dfppy)_2_(CNXyl)­(C≡CR)] (R = Tol *N,N-cis* **-1a**, C_6_H_2_(OMe)_3_ *N,N-cis* **-1b**) compounds with the C≡CR *trans* to C_dfppy_ and a final *mer* disposition of the metalated C atoms (2C_dfppy_, C≡CR, Scheme 1e). However, the substitution of the chloride in the *N,N-cis* **-(** *OC* **-6–24)-1**_**Cl**_ isomer failed under similar reaction conditions (1,2 dichloroethane or toluene under reflux during 18 h), a fact that could be attributed to the strength of the Ir–Cl bond in this isomer as it is located *trans* to the N_dfppy_ atom with lower *trans* influence (Scheme 1f). Furthermore, as the *N,N-trans* **-1**_**Cl**_ precursors, the acetylide *N,N-trans* **-1** **a** **/1b** complexes also photoisomerize to the corresponding *C,C-trans* **-1** **a** **/1b** isomers. Thus, as shown in Scheme 1g, upon irradiation (370 nm, 40 W) of a CD_3_CN solution of *N,N-trans* **- 1a** or -**1b** for 5 min, a quantitative conversion to diastereomer *C,C-trans* **-1a** or **-1b** was obtained. On standing the final CD_3_CN solutions of *C,C-trans* **-1** **a** **/1b** at 298 K for 72 h, this photochemical isomer reverted again to the corresponding *N,N-trans* **-1** *a* **/1b** complex (100% conversion), indicating that, as expected, the ***N,N-trans*** isomer is thermodynamically more stable than the corresponding ***C,C-tran*** ***s***-. Upon similar irradiation conditions, complex *N,N-trans* **-1c** bearing the π-expanded pyrene evolves to a complex mixture of different products that could not be definitively identified. It should be noted that for dfppy cyclometalated species with the C≡CTol and C≡CC_6_H_2_(OMe)_3_ ligands, three different isomers, respectively, have been isolated. Computational data with the tolylacetylide isomers of **1a** reveal that *N,N-cis* **-(** *OC* **-6–32)-1a** is more stable than *N,N-trans-* **1a** by 7.14 kcal/mol, but they fail in relation to the stability of *C,C-trans* **-1a** (Figure S1).

In the same way, the reaction of the related precursors *N,N trans-* **2**_**Cl**_ or **3**_**Cl**_ with AgC≡CR in refluxing 1,2-dichloroethane for **2a**–**c** or room temperature for **3a**–**c** resulted in the ***N,N-trans*** complexes [Ir­(C^N)_2_(CNXyl)­(C≡CR)] [C^N = pbt **2**, pq **3**; R = Tol **a**, C_6_H_2_(OMe)_3_ **b**, pyrene **c** (Scheme 1h)]. Complexes *N,N-trans* **-2b**, **3a**, and **3c** were obtained pure after recrystallization, whereas the rest of counterparts were isolated as analytically pure solids in high yields, without the need for additional purification.

### Characterization of Complexes

All complexes were thoroughly characterized by elemental analyses, IR spectroscopy, electrospray ionization (ESI) mass spectrometry, 1D-NMR (^1^H, ^19^F, ^19^F­{^1^H}, and ^13^C­{^1^H}) and 2D-NMR experiments [^1^H–^1^H (COSY, TOCSY, and NOESY), ^1^H–^13^C­{^1^H} (HSQC, HMBC) and ^19^F–^19^F (TOCSY) (see the Experimental Section and Figures S2–S24), and, when possible, X-ray crystallography. For complexes **1**_**Cl**_ **– 3**_**Cl**_, the most intense peak found in the MS spectra was [M–Cl]^+^, whereas for alkynyl complexes **1a, b, c–3a, b, c**, the most intense peak found was [M–C≡CR]^+^. For the latter, their infrared absorption spectra feature a peak in the range of 2114–2141 cm^–1^, characteristic of the stretching vibrations of the isocyanide C≡N bond, and another peak in the range of 2074–2101 cm^–1^, attributed to the ν­(C≡C) of the corresponding alkynyl ligand. The frequency (wavenumber) of these peaks reflects the strength of both the σ bonding and the π back-bonding between each type of ligand and the metal.^18^

The ^19^F­{^1^H} and ^1^H NMR spectra of all **1**_**Cl**_ **–3**_**Cl**_ complexes show the expected four fluorine signals (doublets, ^4^ *J*_F–F_∼ 9.9–10.4 Hz) and the corresponding two sets of cyclometalated protons corresponding to diastereomers with two nonequivalent cyclometalated ligands (Chart 1). The four isomers of **1**_**Cl**_ display distinctive aromatic (^1^H) and ^19^F resonances, confirming the different arrangement of the ligands around the Ir center. The most characteristic and significative proton and fluorine resonances are highlighted in Figure 1 [H^2/2′^ (*ortho* to N_dfppy_) and H^11/11′^ (*ortho* to C_dfppy_) and in the Supporting Information (F^8/8′^ and F^10/10′^) (Figure S20).

**Figure 1:**
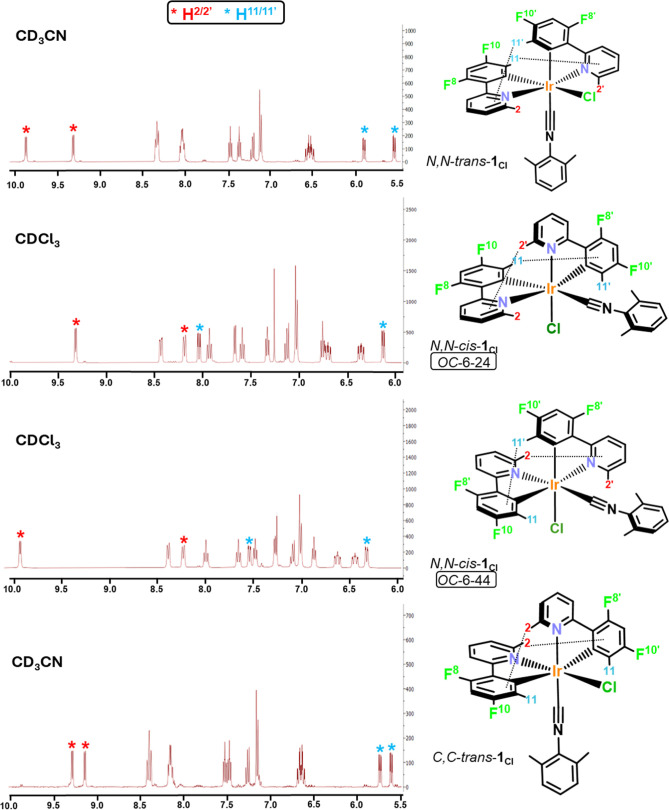
^1^H NMR **(**aromatic region) spectra at 298 K of *N,N-trans* **-1**_**Cl**_, *N,N-cis* **-(** *OC* **-6–24)-1**_**Cl**_, *N,N-cis* **-(** *OC* **-6–44)-1**_**Cl**_, and *C,C-trans* **-1**_**Cl**_.

In *N,N-trans* **-1**_**Cl**_, the H^2,2′^ protons, *ortho* to the N atoms, appear as the most deshielded (δ_H_^2,2′^ 9.88/9.32 CD_3_CN), whereas the H^11,11′^ protons appear as the most shielded signals (δ_H_^11^ 5.55/5.90) because the latter protons are located just over the anisotropic diamagnetic current of the pyridine rings. As shown in Figure 1, the most remarkable changes in these protons take place in the ***N,N-cis*** isomers and affect mainly one of the resonances of these protons (H^2/2′^ and H^11/11′^). One of the two H^2/2′^ protons shifts upfield [8.19 ppm (*OC* **-6–24**); 8.23 ppm (*OC* **-6–44**)] because it now lies over a pyridine ring, while one of the H^11/11′^ moves downfield [8.04 ppm (*OC* **-6–24**); 7.55 ppm (*OC* **-6–44**)], as it loses the diamagnetic effect of the pyridine ring. In the *C,C-trans-* **1**_**Cl**_ isomer, these protons, whose assignment was confirmed with the ^1^H­{^19^F} NMR spectrum (Figure S5b), shift to a lesser extent (δ_H_^2/2′^ 9.30, 9.15; δ_H_^11/11′^ 5.74, 5.61) with respect to the *N,N-trans* **-1**_**Cl**_ isomer. Similar shifts are observed in the ^19^F­{^1^H} NMR spectra (Figure S20), whose assignments were made on the basis of their proton-coupled ^19^F NMR spectra. For *N,N-trans* **1**_**Cl**_, the F^10/10′^ and F^8/8′^ of the dpppy ligands appear as doublets at −109.5; −109.7 (F^10′/10^) and −111.2; −111.1 (F^8′/8^), respectively, and they move for the *N,N-cis*-**1**_**Cl**_ isomers [(*OC* **-6–24**) δ_F_^10/10′^ −105.8, −107.6; δ_F_^8/8′^-109.4, −111.2; (*OC* **-6–44**) δ_F_^10/10′^ −107.2, −107.6; δ_F_^8/8′^-109.3, −109.4] and for the photoisomerized C,C-trans-**11**_**Cl**_ isomer [δ_F_^10/10′^ −108.1, −108.2; δ_F_^8/8′^-109.9, −110.2].

The ^1^H, ^13^C­{^1^H}, and ^19^F­{^1^H} NMR spectra of all alkynyl/isocyanide [Ir­(C^N)_2_(CNXyl)­(C≡CR)] complexes also confirm the presence of the two nonequivalent cyclometalated groups (see Supporting Information for details and assignments). For *N,N-trans* **-1a/1b/1c**, the two most deshielded signals are attributed to the H^2/2′^ protons (in dfppy complexes **1a**–**c**), H^7/7′^ (in pbt complexes **2a**–**c**), and H^8/8′^ (in pq ones **3a**–**c**), whereas the two lowest resonances for the cyclometalated correspond to H^11/11′^ (**1a**–**c**, **2a**–**c**) and H^12/12′^ (**3a**–**c**) protons. The methyl of CNXyl (δ_H_ 1.85–2.23; δ13_C_ 18.2–19.2) and Tol (δ 2.19–2.25; δ13_C_ 21.1–21.3) groups appears as a singlet in the ^1^H and ^13^C­{^1^H} NMR spectra, respectively. As expected, their ^19^F­{^1^H} NMR spectra show four fluorine resonances due to the two nonequivalent dfppy ligands (F^10/10′^ and F^8/8′^), which appear as doublets (^4^ *J*_F–F_ ∼9 Hz). Upon photoisomerization, the aromatic protons and fluorine resonances of the ***C,C-trans*** isomers of **1a** and **1b** clearly change with respect to the ***N,N-trans*** isomers. Notably, one of the two H^2/2′^ and H^11/11′^resonances shifted up- and downfield, respectively, due to their localization with respect to the aromatic rings (Figure S21 for **1a** and Figure S23 for **1b**). Similar shifts were found for the ***N,N-cis*** isomers in the proton and in the corresponding ^19^F NMR spectra (Figures S22 for **1a** and S24 for **1b**).

### X-ray Diffraction Studies

The structures of the three stable stereoisomers of **1**_**Cl**_ [*N,N-trans* **-1**_**Cl**_·CH_2_Cl_2_, *N,N-cis* **-(** *OC* **-6–44)-1**_**Cl,**_ and *N,N-cis* **-(** *OC* **-6–24)-1**_**Cl**_] and of **3**_**Cl**_·CHCl_3_ were confirmed by X-ray (Figure 2 and S25 and Tables S1 and S3). All molecular structures are chiral, but each species crystallizes as the racemate in a centrosymmetric space group. All of them show the typical distorted octahedral geometry. For *N,N-trans*-**1**_**Cl**_·CH_2_Cl_2_ or *N,N-trans*-**3**_**Cl**_·CHCl_3_, the N(1)–Ir–N(2) angles are 170.7(9)° for **1**_**Cl**_ and 168.4(17)° for **3**_**Cl**_. Interestingly, the Ir–N(1) in *N,N-cis* **-(** *OC* **-6–44)-1**_**Cl**_ and Ir–N(2) *N,N-cis* **-(** *OC* **-6–24)-1**_**Cl**_ were elongated [2.147(2) and 2.146(2) Å, respectively] with respect to the rest of Ir–N distances because of the higher *trans* influence of the metalated carbon. The crystal packing of these structures reveals π···π interactions between two neighboring molecules involving Xyl···aryl of dfppy (*N,N-trans* **–1**_**Cl**_), dfppy···dfppy (*N,N-cis-OC* **-6–24–1**_**Cl**_), and Xyl···Xyl rings (*N,N-cis* *-OC* **-6–44–1**_**Cl**_) (Figures S26–S29).

**Figure 2:**
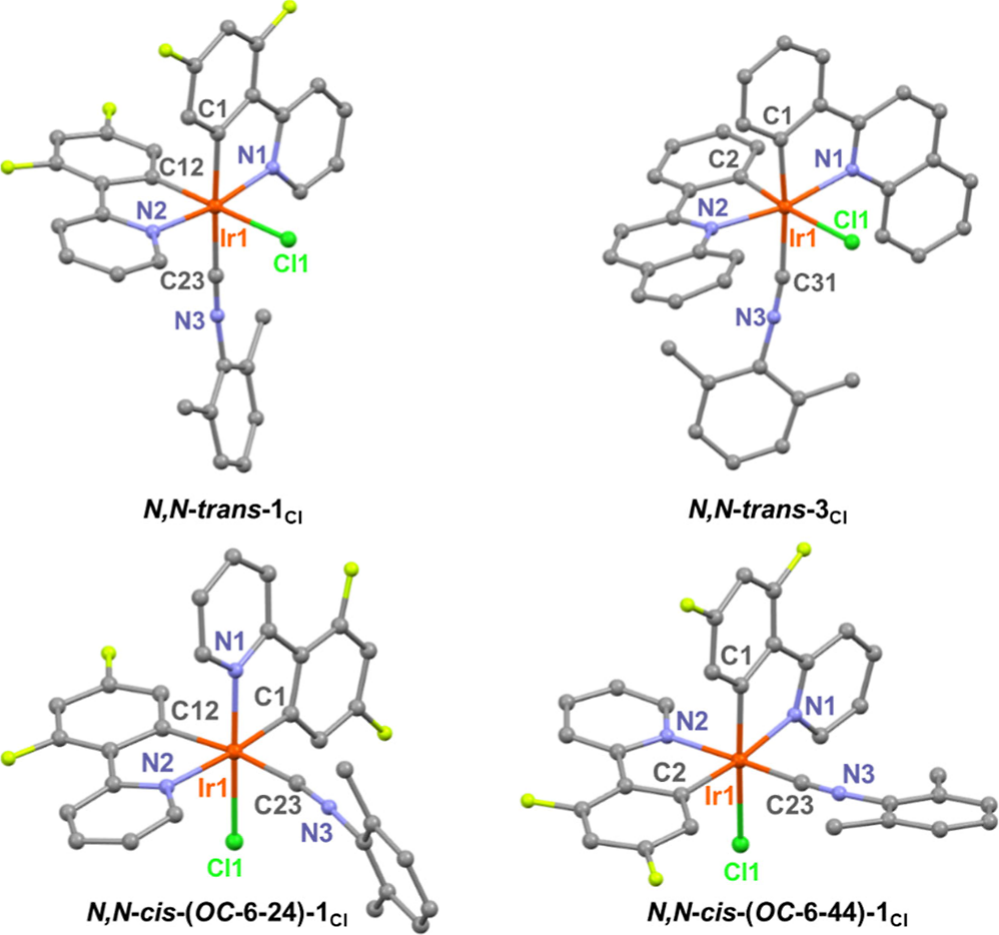
Crystal structures of *N,N-trans* **-1**_**Cl**_·CH_2_Cl_2_, *N,N-trans* **-3**_**Cl**_·CHCl_3_, *N,N-cis* **-(** *OC* **-6–24)**-**1**_**Cl**_, and *N,N-cis* **-(** *OC* **-6–44)-1**_**Cl**_.

Single crystals were also obtained for complexes *N,N-trans* **-1a**, **1c**, **2a**, **2c** and *N,N-cis* **-1a**, **1b**, and their molecular structures, determined by single-crystal X-ray diffraction, are shown in Figure 3 and S25. Data collection and refinement parameters are summarized in Table S2, and a selection of distances and angles are collected in Tables S4 and S5. In general, the structures of the ***N,N-trans*** isomers are reminiscent of several other isocyanide-ligated cyclometalated iridium complexes,^7b,7c,7d,7e,7f,7g,7h,8^−,,^19,8,19^ with the expected *trans* arrangement of the N_(C^N)_ nitrogen atoms and an overall distorted octahedral coordination environment in all cases. The molecular structures are chiral, but in each case, the complexes crystallize as racemates in a centrosymmetric space group (see Supporting Information). In these, the N(1)–Ir–N(2) angles for the two *trans N,N* atom are between 166.6(19) and 171.9(11)°. The Ir–C bonds with isocyanides are slightly shorter [1.983(5)–1.986(4) Å] than the Ir–C bonds with the acetylides [2.060(6)–2.110(5) Å] and the Ir–C_C^N_ ones [Ir–C(1) 2.045(3)–2.075(4) Å; Ir–C(2) 2.055(5)–2.080(6) Å]. Comparing the structures, there are some differences in bond metrics brought out by changing the C^N ligand and the geometry (***N,N* **-** *trans*** vs ***N,N-cis***). In ***N,N-trans***, the Ir–N distances are slightly shorter [2.049(6)–2.080(6) Å] than those in the ***N,N-cis*** isomers [2.090(5)–2.156(5) Å], due to the higher *trans* influence of the C atoms in the latter. Both the Ir–C–C_(C≡CR)_ and Ir–C–N_(C≡NXyl)_ bond angles are slightly distorted from the linearity [166.6(4)–177.1(5)°; 169.5(3)–174.6(4)°]. The aryl group of CNXyl twisted over 51.1° (**1a**), 45.2° (**1c**), 39.3° (**2a**), and 46.2° (**2c**) against the plane formed by the four carbons (C1, C2, C≡C, and C≡N). In the alkynyl derivatives, the dihedral angles formed by the aryl substituent (Tol, C_6_H_2_OMe_3_, pyrene) in relation to the mutually *trans* aryl cyclometalated ring are 17.4° (**1a**), 85.3° (**1c**), 21.1° (**2a**), 51.6° (**2c**) in the ***N,N-trans*** isomers and 22.4° (*N,N-cis-* **1a)** and 57.7° (*N,N-cis-* **1b**) in the ***N,N-cis*** configured. The rings on the substituents on the CNXyl and C≡CR ligands form dihedral angles between them of 87.54° (**1a**), 48.36° (**1c**), 76.76° (**2a**), and 57.41° (**2c**). Finally, the crystal packing of these structures reveals π···π interactions between two neighboring molecules involving Xyl···Xyl rings (*N,N-trans* **-1a**, *N,N-cis* **-(** *OC* **-6–32)-1a**), dfppy···dfppy (*N,N-cis* **-(** *OC* **-6–32)-1b**), Xyl···pyrene (**1c**), and pbt···pbt (**2a**, **2c**) (Figures S30–S35).

**Figure 3:**
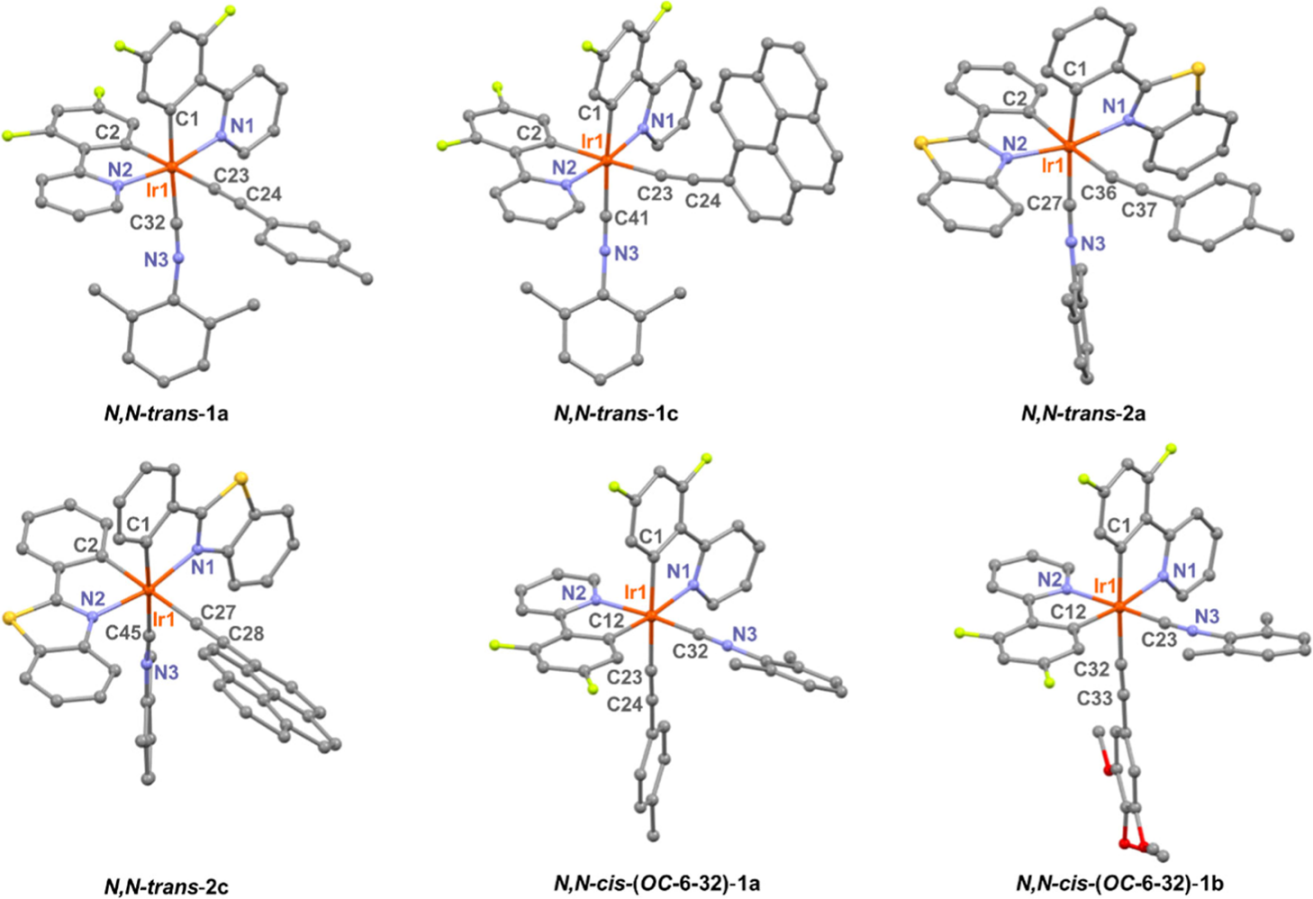
Crystal structures of *N,N-trans*-**1a**, *N,N-trans*-**1c**·0.6CHCl_3_, *N,N-trans*-**2a**, *N,N-trans*-**2c**, *N,N-cis*-**1a**·1.75CH_2_Cl_2_, and ***N,N-cis***-**1b**·0.9CH_2_Cl_2_.

### Optical Properties

#### Absorption Properties and TD-DFT Calculations

The UV–vis absorption spectra data of all complexes are summarized in Table S6, and relevant spectra are provided in Figures 4 and S36–S38. Additionally, TD-DFT calculations were performed on the four isomers of **1**_**Cl**_ [*N,N-trans* **-1**_**Cl**_, *N,N-cis* **-1**_**Cl**_ (*OC*-**6–24**) and (*OC*-**6–44**), and *C,C-trans* **-1**_**Cl**_] and on the alkynyl/CNXyl derivatives *N,N-trans* **-1a**, *N,N-cis* **-1a**, and *N,N-trans* *-* **1c**, **2a**–**c**, and **3a,c** to support the assignments in this study (see details in the Supporting Information, Tables S7–S42 and Figures S39–S62). A schematic representation of selected frontier orbitals and calculated low-lying excitations in the alkynyl derivatives is shown in Figure 5.

**Figure 4:**
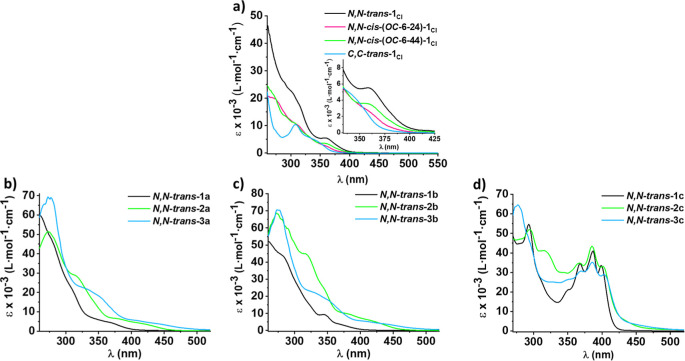
UV–vis absorption spectra of (a) different isomers of **1**_**Cl**_ and (b–d) *N,N-trans* **-1–3a**–**c** in CH_2_Cl_2_ solution (5 × 10^–5^ M) and *C,C-trans* **-1**_**Cl**_ in CH_3_CN at 298 K.

**Figure 5:**
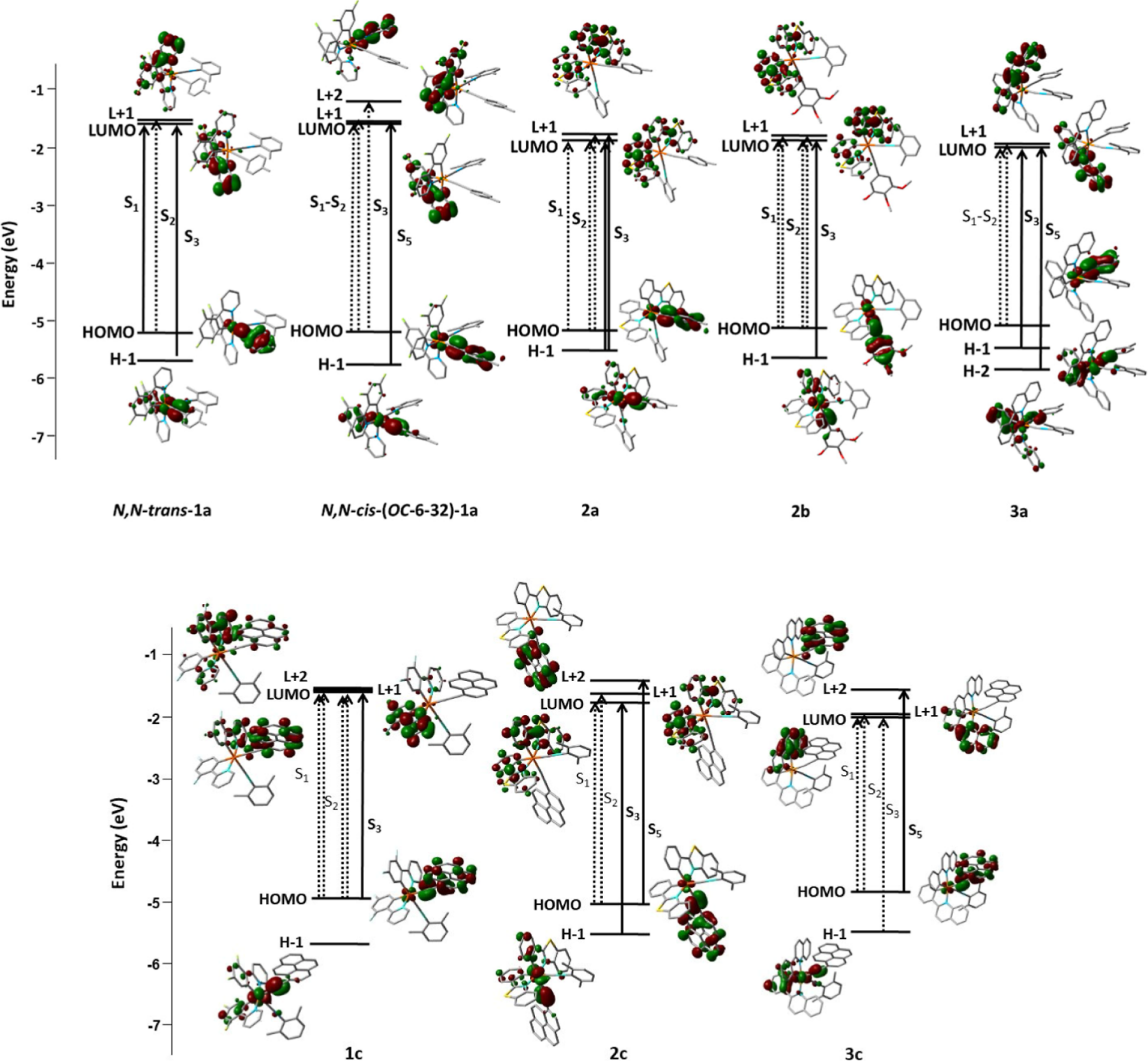
Schematic representation of selected frontier orbitals and excitations of *N,N-trans* **-1a**, *N,N* **-** *cis* **-(** *OC* **-6–32)-1a**, **2a**, **2b, 3a** (up), and **1c**, **2c**, and **3c** (down). Transition with the highest *f* (oscillator strength) is in bold.It should be "C^N"

All complexes show high-energy absorption peaks (λ < 330 nm), assigned to spin-allowed ^1^ππ* ligand-centered (^1^IL) transitions from all ligands. The low-energy profile of the different isomers of precursor **1**_**Cl**_ (CH_2_Cl_2_ solution or CH_3_CN for *C,C-trans-* **1**_**Cl**_) is rather similar, exhibiting a band at 360 with a shoulder at 380 nm, extending to 400–420 nm for *N,N-trans* **-1**_**Cl**_ and *N,N-cis* **-(** *OC* **-6–44)-1**_**Cl**_, which is slightly blue-shifted for *N,N-cis* **-(** *OC* **-6–24)-1**_**Cl**_ and *C,C-trans-* **1**_**Cl**_ (∼340 nm) (Figure 4a). The two lowest calculated excitations have ^1^ILCT/^1^MLCT nature with some minor chloride-to-cyclometalated [XLCT for *N,N-trans* **-1**_**Cl**_ and *N,N,-cis*-**(** *OC* **-6–44)-1**_**Cl**_] or isocyanide-to-cyclometalated [L″LCT for *N,N-cis*-**(** *OC* **-6–24)-1**_**Cl**_] character. The blue shift for the *C,C-trans-* **1**_**Cl**_ and *N,N-cis* **-(** *OC* **-6–24)-1**_**Cl**_ isomers is reflected in the calculated value for the S_1_ transition [361 *N,N-trans* **-1**_**Cl**_; 360 *N,N-cis* **-(** *OC* **-6–44)-1**_**Cl**_ vs 354 nm *N,N-cis* **-(** *OC* **-6–24)-1**_**Cl**_ **/** *C,C-trans-* **1**_**Cl**_]. As expected, the influence of the cyclometalated ligand in the low-energy region is visible in the chloride/CNXyl precursors *N,N-trans* **-1**_**Cl**_ **–3**_**Cl**_ (dfppy 380; pbt 400; 430 nm pq, with tails to 420–480 nm), in line with the energy of the aryl-based HOMO (dfppy lower than pbt) and the higher delocalization and π-acceptor character of the pq ligand (Figure S36).

From the alkynyl complexes, the different isomers of C≡CTol (**1a**) [*N,N-trans* **-1a**, *N,N-cis­(OC-* **6–32)-1a**, and *C,C-trans-* **1a**] or C≡CC_6_H_2_OMe_3_ (**1b**) [*N,N-trans* **-1b**, *N,N-cis-* **(** *OC* **-6–32)-1b**, and *C,C-trans-* **1b**] (Table S6 and Figure S37) exhibit similar low-energy profiles to the corresponding precursor with a slight bathochromic shift, thus reflecting the donor character of the alkynyl ligand. According to calculations on *N,N-trans* **-1a** and *N,N-cis­(OC-* **6–32)-1a**, the calculated absorption spectra are in reasonable agreement with the experimental data (Figures S48 and S50). As illustrated in Figure 5, for *N,N-trans*-**1a**, the most intense low-lying excitations (S_1_/S_3_ 405/368 nm) involve alkynyl/Ir-to-cyclometalated charge transfer having ^1^L^′^LCT/^1^MLCT (L^′^ = C≡CR) character. For isomer *N,N-cis*-(*OC* **-6–32)-1a**, the L+2 located on CNXyl is closer in energy to LUMO and L+1, and therefore, whereas S_1,2;4,5_ (∼409; 353, 348 nm) excitations have ^1^L^′^LCT/^1^MLCT character, S_3_ (357 nm) has mixed metal/alkynyl-to-CNXyl nature (Figure 5).

In ***N,N-trans*** pbt and pq derivatives **2a**, **3a** and **2b**, **3b**, the low-energy bands are red-shifted in relation to dfppy **1a**,**b** spanning to 460 (**2**) or 480–500 nm (**3**) (Table S6 and Figure 4b,c), thus mimicking the influence of the C^N ligand as in the *N,N-trans-* **1a**,**b** chloride precursors. However, the influence of the aryl (C≡CTol **a** vs C≡CC_6_H_2_(OMe)_3_ **b**) is minimal (Figure S38). These bands are ascribed to mixed alkynyl/Ir-to-C^N (^1^L^′^LCT/^1^MLCT) charge transfer transitions. For comparison, the low-energy calculated excitations S_1–3_ for complexes **2a**, **2b**, and **3a** are included in Figure 5. The S_1_ (cal. 453 **2a**, 461 **2b**, 501 nm **3a**) and S_2_ excitations have very low oscillator strength, S_3_ (417 **2a**, 395 **2b,** 438 nm **3a**) being the most intense.

Consistent with the increased π-conjugation of the pyrene alkynyl ligand, ***N,N-trans*** complexes **1c**–**3c** exhibit a well-structured and intense low-energy absorption (λ_max_ 400 **1c**; 401 **2c**; 403 **3c** nm), with extending tails to tower energies, depending on the C^N group (up 425 **1c**; 460 **2c**; 500 nm **3c**) (Figure 4d). As expected, these absorptions are substantially red-shifted relative to related ***N,N-trans-*** (C≡CTol **a**, C≡CC_6_H_2_(OMe)_3_ **b**) complexes, due to the stronger conjugation of the pyrenyl group.^20^ According to calculations, these are ascribed to transitions located on the pyrenyl or C≡C-pyr group (^1^ ππ*_pyr_ here ^1^IL^′^_pyr_, Figures 5 and S38) with a variable contribution of ligand (C≡C-pyr) to C^N, depending on the cyclometalated group. As shown in Figure 5, for dfppy complex **1c**, the most intense excitation is S_3_ (393 nm, HOMO→L+2 88%), which involves C≡C-pyrene-to-cyclometalated (^1^L^′^_py__r_LCT) charge transfer character with minor ^1^MLCT contribution. The calculated S_1,2_ transitions (435/429 nm), with low intensity, exhibit mixed ^1^IL^′^_C≡C_/^1^L^′^_py__r_LCT nature with somewhat MLCT contribution. For the pbt **2c** complex, the most intense excitation is S_5_ (381 nm, HOMO→L+2) and is located on the pyrenyl group (^1^IL^′^_py__r_) with minor ML^′^_py__r_CT contribution. The related S_3_ with moderate intensity (403 nm, H-1→LUMO) has mixed CC-ligand^′^-to-ligand and metal-to-ligand ^1^L^′^_C≡C_LCT/^1^MLCT nature, whereas the calculated S_1,2_ (439, 422 nm) reinforces the ligand^′^-to-ligand character (^1^L^′^_py__r_LCT) with some ^1^MLCT contribution. Finally, for pq complex **3c**, the most intense calculated transition S_5_, found slightly red-shifted (411 nm, HOMO→L+2), is located on C≡Cpyrenyl with some charge transfer from C≡C to pyr (^1^IL^′^_pyr_CT). The low-lying calculated S_1_–_4_ transitions, with moderate (S_4_ 431 nm) or low intensity (S_1–3_ 535, 513, 439 nm; HOMO, H-1 → LUMO, L+1), have mainly ^1^L^′^_py__r_LCT/^1^MLCT character. For **3c**, the calculated values are bathochromically shifted in relation to **1c** and **2c**, in agreement with the experimental UV spectra (Figure 4d).

### Emission Properties and DFT Calculations

A summary of photophysical data of all complexes is provided in Table 1. The four isomers of **1**_**Cl**_ [*N,N-trans*-**1**_**Cl**_, *N,N-cis*-**(** *OC* **-6–24/44)-1**_**Cl**_, and *C,C-trans*-**1**_**Cl**_] show in CH_2_Cl_2_ (Figure S63, CD_3_CN for *C,C-trans*-**1**_**Cl**_) and PS film (Figure 6a) a similar vibronic emission spectrum with minimal blue shifts in the λ_max_ for the *cis* and *C,C-trans* isomers (λ_max_ *N,N-trans*-**1**_**Cl**_ 454 nm vs 450–451 nm). In solution, *N,N-cis*-**(** *OC* **-6–24**) also shows a short-lived high-energy shoulder at 430 nm (τ 10 ns) attributed to residual fluorescence, likely due to a minor metal contribution in the excited state (Figure S63). As shown in Figure S63b, the excitation spectra monitored at 430 and 450 nm are essentially identical, thus indicating that these peaks share the same origin. The measured quantum yield for *N,N-trans*-**1**_**Cl**_ in fluid solution was notably higher than for the other isomers (ϕ = 24% vs ϕ *OC* **-6–24**, 1.9%; *OC* **-6–44** 4.4%; *C,C-trans-* **1**_**Cl**_ 0.7%). The efficiency of the *cis* isomers increases notably in the PS film due to the rigidity of the medium (Table 1). The DFT calculations show that the two lowest triplet excited states (T_1, 2_) of different isomers of **1**_**Cl**_ are close in energy and are generated from a complex mixture of transitions (HOMO, H-1 to H-4→LUMO, L+1) (see Tables S8, S11, and S14). T_1_ shows mixed ^3^ILCT/^3^MLCT character with minor ^3^XLCT and/or ^3^L^′′^LCT (L^′′^ = CNXyl) character, and the calculated values follow the experimental data. Optimization of the T_1_ states reveals that spin density is primarily located in one of the dfppy ligands in the three stable isomers [*trans*-to Cl in *N,N-trans*-**1**_**Cl**_ or *trans* to CNXyl in *N,N-cis*-**1**_**Cl**_ **(** *OC* **-6–24/44)**] having mainly a ^3^ILCT nature with minor ^3^MLCT contribution (Tables S9, S12, and S15). For unstable photoisomerized *C,C-trans-* **1**_**Cl**_, optimized T_1_ in MeCN is mainly located on the chloride and Ir atom with minor contribution of the aryl groups of dfppy ligands, suggesting a mixed ^3^XMCT/^3^XLCT character for the emission (Tables S17 and S18). The pbt (**2**_**Cl**_) complex shows in all media structured emission, whereas the pq (**3**_**Cl**_) complex exhibits a broad band in CH_2_Cl_2_ and PS1%, indicative of higher contribution of ^3^MLCT in the excited state (Figures 6b and S64). As expected, the wavelength of the emission depends on the cyclometalated employed (454 nm *N,N-trans*-**1**_**Cl**_, 525 nm *N,N-trans*-**2**_**Cl**_, 573 nm *N,N-trans*-**3**_**Cl**_), with a clear red shift for the most delocalized phenylquinoline. It should be noted that the measured quantum yield in solution for all *N,N-trans* **-1**_**Cl**_ **–3**_**Cl**_ complexes is lower in film than in CH_2_Cl_2_ solution, suggesting the occurrence of some triplet–triplet quenching in rigid media.

**Table 1:** Photophysical Data for All Complexes

	CH_2_Cl_2_						PS 1% wt				
	298 K					77 K	298 K				
compound	λ_max_/nm^a^	τ/μs^b^	ϕ_P_ ^c^	*k*_r_	*k*_nr_	λ_max_/nm^a^	λ_max_/nm^a^	τ/μs	ϕ_PL_	*k*_r_	*k*_nr_
***N,N-trans***-**1**_**Cl**_	454	0.47 (452)	24	5.1 × 10^5^	1.6 × 10^6^	448	453	1.64 (453)	20.7	1.3 × 10^5^	4.8 × 10^5^
***N,N-cis-*(*OC-*6–24**)-**1**_**Cl**_	430_sh_	0.01 (430)	1.9	2.9 × 10^4^	1.5 × 10^6^	445	452	0.63	10.2	1.6 × 10^5^	1.4 × 10^6^
	451	0.66 (450)									
***N,N-cis-*(*OC-*6–44**)-**1**_**Cl**_	450	0.79 (450)	4.4	5.6 × 10^4^	1.2 × 10^6^	443	448	0.36	17.6	4.9 × 10^5^	2.3 × 10^6^
***C,C-trans***-**1**_**Cl**_ ^d^	451	0.14 (450)^e^	0.7	5.0 × 10^4^	7.1 × 10^6^	443	-	^g^	-	-	-
						474					
***N,N-trans***-**1a**	452	0.12 (452)	4.5	3.8 × 10^5^	8 × 10^6^	444	452	1.43 (452)	17.7	1.2 × 10^5^	5.8 × 10^5^
***N,N-cis-*(*OC-*6–32**)-**1a**	447	0.11 (450)	2	1.8 × 10^5^	8.9 × 10^6^	441	446	0.47	15.1	3.2 × 10^5^	1.8 × 10^6^
***C,C-trans-*1a** ^d^	455	0.16 (450)^e^	0.2	1.3 × 10^4^	6.2 × 10^6^	457	-	^g^	-	-	-
	495					477					
***N,N-trans***-**1b**	452	0.11 (452)	2.6	2.4 × 10^5^	9 × 10^6^	444	453	0.77 (453)	17.5	2.3 × 10^5^	1.1 × 10^6^
***N,N-cis-*(*OC-*6–32**)-**1b**	454	0.30 (450)	1.3	4.3 × 10^4^	3.3 × 10^6^	441	446	0.45	12.8	2.8 × 10^5^	1.9 × 10^6^
***C,C-trans***,-**1b** ^d^	453	0.21 (450)^e^	0.6	2.8 × 10^4^	4.7 × 10^6^	447	-	^g^	-	-	-
	508					477					
***N,N-trans-*1c**	384^h^	0.07 (405)^e^	1	2.1 × 10^4^	2.1 × 10^6^	443^h^	453_max_ ^h^	1.54 (453)	1	6.5 × 10^4^	6.4 × 10^5^
	405,420^h^	0.48 (480)^f^				475^h^	657^h^				
	450, 480^h^	0.23 (657)^f^				650^h^	675^h^				
	657^h^					670^h^					
	672^h^										
***N,N-trans-*2**_**Cl**_	525	0.38 (525)	60.4	1.6 × 10^6^	1.1 × 10^6^	513	560	2.38 (560)	51.5	9.9 × 10^4^	3.2 × 10^5^
***N,N-trans-*2a**	523	1.55 (523)	47	3.0 × 10^5^	3.4 × 10^5^	512	558	2.66 (558)	28.8	1.1 × 10^5^	2.7 × 10^5^
***N,N-trans-*2b**	525	0.80 (525)	43	5.4 × 10^5^	7.1 × 10^6^	513	559	2.11 (559)	23.5	1.1 × 10^5^	3.6 × 10^5^
***N,N-trans-*2c**	405	0.04 (405)	1.4	1,5 × 10^4^	1.1 × 10^6^	516	520	1.98 (506)	3.5	1.8 × 10^4^	4.9 × 10^5^
	520	0.89 (520)				650					
***N,N-trans-*3**_**Cl**_	573	0.56 (573)	22	3.9 × 10^5^	1.4 × 10^6^	551	557	1.67 (557)	14.4	8.6 × 10^4^	5.1 × 10^5^
***N,N-trans-*3a**	581	0.32 (581)	29.8	9.3 × 10^5^	2.2 × 10^7^	573	582	1.71 (582)	19.7	1.2 × 10^5^	4.7 × 10^5^
***N,N-trans-*3b**	584	0.52 (584)	25.1	4.8 × 10^5^	1.4 × 10^6^	570	589	1.69 (589)	19.4	1.1 × 10^5^	4.8 × 10^5^
***N,N-trans-*3c**	588	0.10 (588)	1.6	1.6 × 10^5^	9.8 × 10^6^	588	589	1.51 (588)	3.1	2.1 × 10^4^	6.4 × 10^5^
	656_sh_	0.61 (656)				556	656_sh_	2.13 (658)			
						656_sh_					

a Highest energy peak of the band.

b (τ_average_) the luminescence days fit to two components.

c Under N_2_.

d Emission and τ in CH_3_CN solution (5 × 10^–5^ M) at 298 K, ϕ_P_ = CH_3_CN (1 × 10^–3^ M).

e LED, λ_ex_ = 370 nm.

f LED, λ_ex_ = 390 nm.

g These photoisomers were obtained in CH_3_CN.

h Emission data results of λ_ex_ = 360–370 nm.

**Figure 6:**
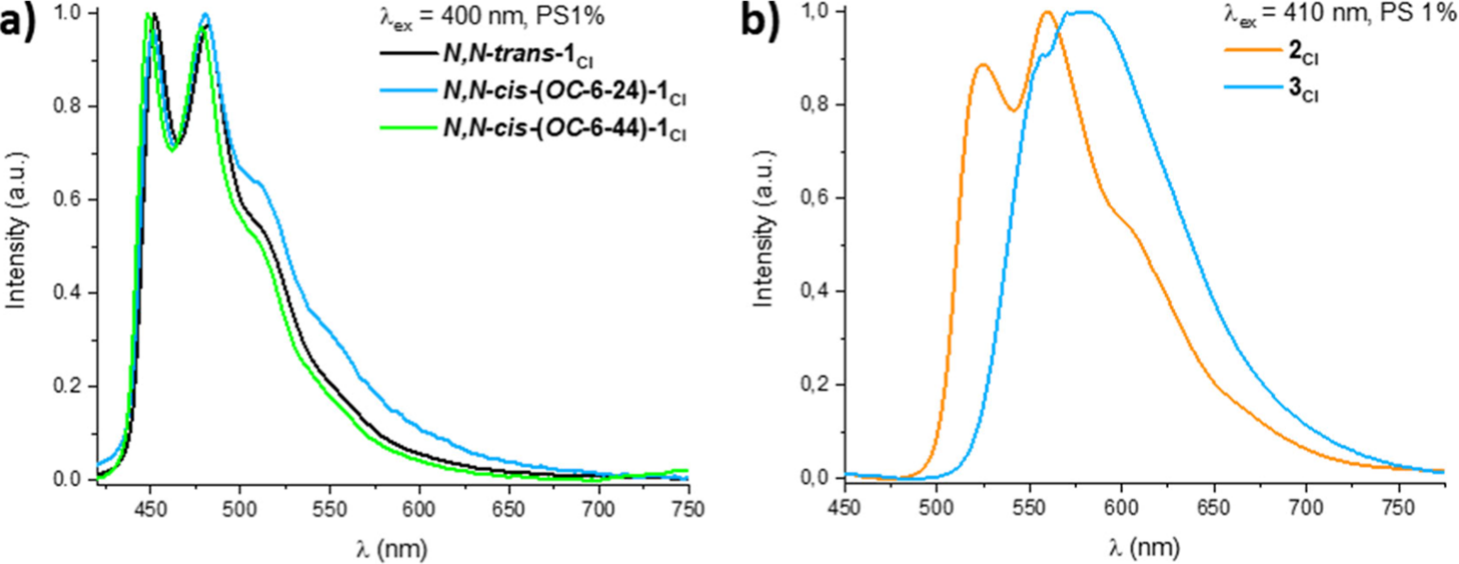
Emission spectra in PS film 1% of (a) *N,N-trans*-**1**_**Cl**_, *N,N-cis-* **(** *OC* **-6–24)-1**_**Cl**_, and *N,N-cis-* **(** *OC* **-6–44)-1**_**Cl**_ and (b) complexes **2**_**Cl**_ and **3**_**Cl**_.

The ***N,N-trans***-dfppy (**1**) and -pbt (**2**) complexes with C≡CTol (**a**) (Figure 7) and C≡CC_6_H_2_(OMe)_3_ (**b**) (Figures S65 and S66) display in all media (Table 1) a structured emission, with small rigidochromism at 77 K, whose λ_max_ essentially depend on the cyclometalated ligand [452 nm (CH_2_Cl_2_ or PS) *N,N-trans*-**1a**, **1b**; 525 (CH_2_Cl_2_), 558 nm (PS) *N,N-trans*-**2a**, **2b**]. The pq complexes *N,N-trans* (**3a**, **3b**) show a red-shifted broad unstructured band at 580–590 nm at 298 K, which is slightly blue-shifted in glasses (573 **3a**, 570 nm **3b**) and somewhat more structured. The phosphorescence decays for these complexes are biexponential (range 0.11–1.55 μs, CH_2_Cl_2_ 298 K; 0.45–2.66 μs, PS films), with **2a** showing the longest lifetime. The phosphorescence quantum yields (ϕ) were measured in CH_2_Cl_2_ and PS films at 298 K. The pbt (**2a**, **2b**) and pq (**3a**, **3b**) complexes exhibit brighter emission in solution (ϕ_PL_ = 47 **2a**, 43% **2b**; 29.8 **3a**, 25.1% **3b**) than the dfppy ones (ϕ_PL_ = 4.5 **1a**, 2.6% **1b**). The quantum yields also show a certain dependence with the alkynyl substituents, being slightly brighter with C≡C-Tol (**2a**, **3a**) than C≡C–C_6_H_2_(OMe)_3_ (**2b, 3b**). These results are also observed in PS 1 wt % under air, but whereas the ϕ_PL_ in **1a** and **1b** (17.7 **1a**, 17.5% **1b**) increases notably with respect to those in solution, the ϕ_PL_ in **2a**,**b** and **3a**,**b** decreases (28.8 **2a**, 23.5% **2b**; 19.7 **3a**, 19.4% **3b**), probably due to self-quenching by triple–triplet aniquilation, as observed in the precursors.^21^

**Figure 7:**
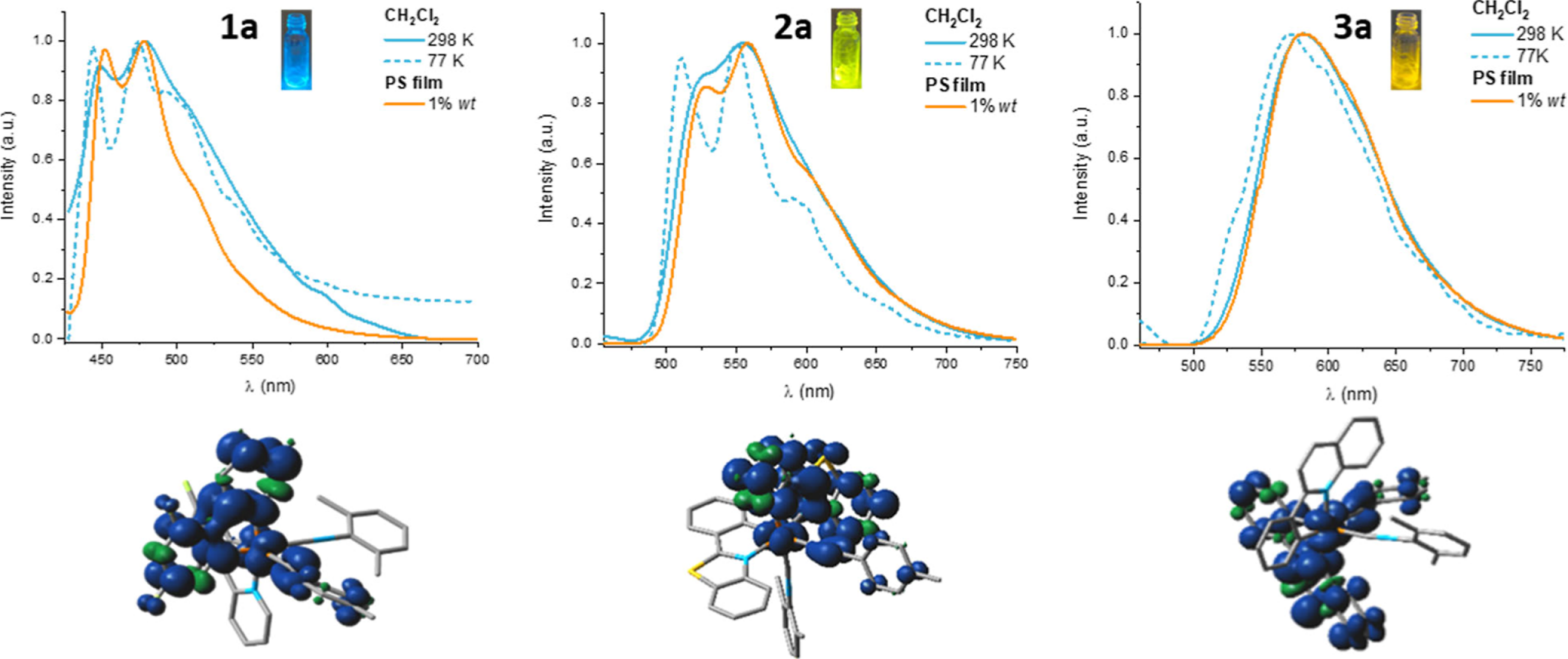
Emission spectra in CH_2_Cl_2_ solution at 298 K and 77 K and PS 1% (λ_ex_ = 400–420 nm) of *N,N-trans*- **1a**–**3a** complexes and their spin density distribution for the lowest triplet excited state below (photographs show the emission of PS).

Notably, the geometrical isomers *N,N-cis* **-(** *OC* **-6–32)-1** **a** **/1b** exhibited nearly identical emission bands to the corresponding ***N,N-trans***-**1** **a** **/1b** isomers (Table 1 and Figure S66), although with lower quantum yields than *N,N-trans*-**1** **a** **/1b** (i.e, ϕ, CH_2_Cl_2_/PS *N,N-trans*-**1a** 4.5/17.7% vs *N,N-cis* **-(** *OC* **-6–32)-1a** 2/15.1%). However, in the case of photoisomers *C,C-trans* **-1** *a* **/1b**, their emission spectra exhibited a broad band at 495 nm (**1a**) or 508 nm (**1b**), respectively, clearly different from their corresponding *N,N-trans* **-1** **a** **/1b** precursors, although with lower quantum yields in both species (ϕ 0.2% **1a**, 0.6% **1b**) (Figures S67).

It should be mentioned that, despite the relative strong field nature of the alkynyl ligands, in the series of complexes with dfppy and pbt cyclometalated groups, the substitution of Cl with C≡CR groups does not produce the expected improvement in emission efficiency (Table 1). The incorporation of the electron donor alkynyl ligands modifies the nature of the low-lying excited state from ^3^ILCT/^3^MLCT in the Cl/CNXyl complexes to a lowest excited state having strong ^3^L^′^LCT (L^′^ = C≡CR) charge transfer with minor ^3^MLCT character. This change decreases *k*_r_, and additionally, the presence of substituents (R) on the alkynyl ligands increases nonradiative decay rates, resulting in a lower luminous efficiency (see Table 1). By contrast, in pq derivatives **3a** and **3b**, the yield increases in relation to **3**_**C**l_, mainly due to an increase of *K*_r_ (see Table 1). These results and the unstructured shape of the emission suggest a ^3^L^′^LCT excited state with remarkable ^3^MLCT contribution, as supported by calculations.

The nature of the emissions was examined on selected complexes, *trans-*configured *N,N-trans*-**1a, 2a,b, 3a** and *cis*-configured, *N,N-cis* **-(** *OC* **-6–32)-1a** by TD-DFT calculations. In the *N,N-trans*-**1a, 2a,b, 3a** derivatives, the S_0_→T_1,2_ transitions are close in energy and involve mainly a mixture of transitions of ^3^L^′^LCT/^3^MLCT/^3^ILCT character. The calculated wavelengths for low-lying T_1_ agree well with the tendency observed for the experimental data (T_1_ 424 **1a**, 482 **2a**, 487 nm **2b**, 521 **3a**). The spin density at the optimized T_1_ state (Figure 7), as well as the single occupied orbital (SOMO) and SOMO-1 orbitals, is located on the cyclometalated ligand *trans* to CNXyl, iridium, and C≡CR, in variable extension, thus supporting an emission mainly attributed to metal–ligand-to-ligand charge transfer, M/C≡CR→C^∧^N (^3^L^′^LCT/^3^MLCT), with some ^3^ILCT contribution. The calculated spin density on the Ir center has similar values in **1a** and **2a,b**, increasing in pq derivative **3a** (0.1331 **1a**, 0.125 **2a**, 0.122 **2b** vs 0.180 **3a**). The geometrical isomer *N,N-cis* **-(** *OC* **-6–32)-1a** does not show significant differences in the HOMOs and LUMOs distribution in relation to *N,N-trans* *-* **1a**. However, the calculated S_0_→T_1_ transition (red-shifted to 430 vs 424 nm in *trans* **-1a**) does not agree with the observed blue shift in the experimental values (447 *cis-* **1a** vs 452 nm *trans* **-1a**). The spin density at the optimized T_1_ state shows a lower C≡CTol and Ir contribution at SOMO-1 (Ir/C≡CTol/dfppy 21/21/55% in *cis-* **1a** vs 23/27/47% in *trans* **-1a)**, suggesting a similar ^3^L^′^LCT/^3^MLCT/^3^ILCT nature but with higher ^3^ILCT contribution.

The emission spectra of pyrene-substituted alkynyl complexes *N,N-trans*-**1c**–**3c**, which depend markedly on the cyclometalated ligand, are collected in Figure 8. The dfppy complex, *N,N-trans* **-1c**, presents an emission profile with three bands whose intensity depends on temperature, media, and wavelength excitation. Thus, upon exciting at 360 nm in CH_2_Cl_2_ at 77 K (or in a rigid PS film at 298 K), the emission is formed by a structured band with λ_max_ at 450 nm, similar to that observed in the other dfppy complexes, ascribed to the ^3^L^′^_py__r_LCT/^3^MLCT phosphorescence of the “Ir­(dfppy)_2_(C≡C)” chromophore, and a low energy structured band (657, 725 nm), attributed to the ^3^(π→π*) state of C≡C-pyrene (^3^IL^′^_py__r_). This assignment follows similar assignments made in related Pt­(II)–C≡C-pyrene complexes^20,22^ and is further supported by the calculated spin density of optimized T_1_, mainly located on C≡C-pyr with small Ir contribution (Figure 8). However, in CH_2_Cl_2_ at 298 K, an additional short-lived high-energy structured band (384, 405, and 420 nm) ascribed to fluorescence (^1^IL^′^_py__r_) is developed. This band overlaps partially with the phosphorescence, ascribed to the “Ir­(dfppy)_2_(C≡C)” chromophore (λ_max_ 450 nm). The calculations indicate that the T_2,3_ excited states associated with the ^3^L^′^_py__r_LCT/^3^MLCT of the “Ir­(dfppy)_2_(C≡C)” fragment are quasiisoenergetic with S_1_, which could enable electronic energy transfer between ^3^L^′^_pyr_LCT/^3^MLCT and ^1^IL^′^_pyr_ at room temperature. A similar behavior is observed upon exciting at 390 nm, but in this case, the contributions of the high- and low-energy band emissions associated to the C≡C-pyr chromophore (^1^IL^′^_pyr_ and ^3^IL^′^_pyr_) increase in relation to the ^3^L^′^_pyr_LCT/^3^MLCT of “Ir­(dfppy)_2_(C≡C)” (Figure S68a). The measured lifetimes in solution are shorter in the fluorescence peak [τ_av_ 70 ns (405 nm)] than those for the phosphorescence bands [τ 0.48 μs (480 nm), 0.23 μs (657 nm)], and, as expected, in nondegassed CH_2_Cl_2_ solution, the ^3^IL^′^_pyr_ phosphorescence band is lost, although the phosphorescence associated with the “Ir­(C^N)_2_(C≡C)” core is still visible (Figure S68b).

**Figure 8:**
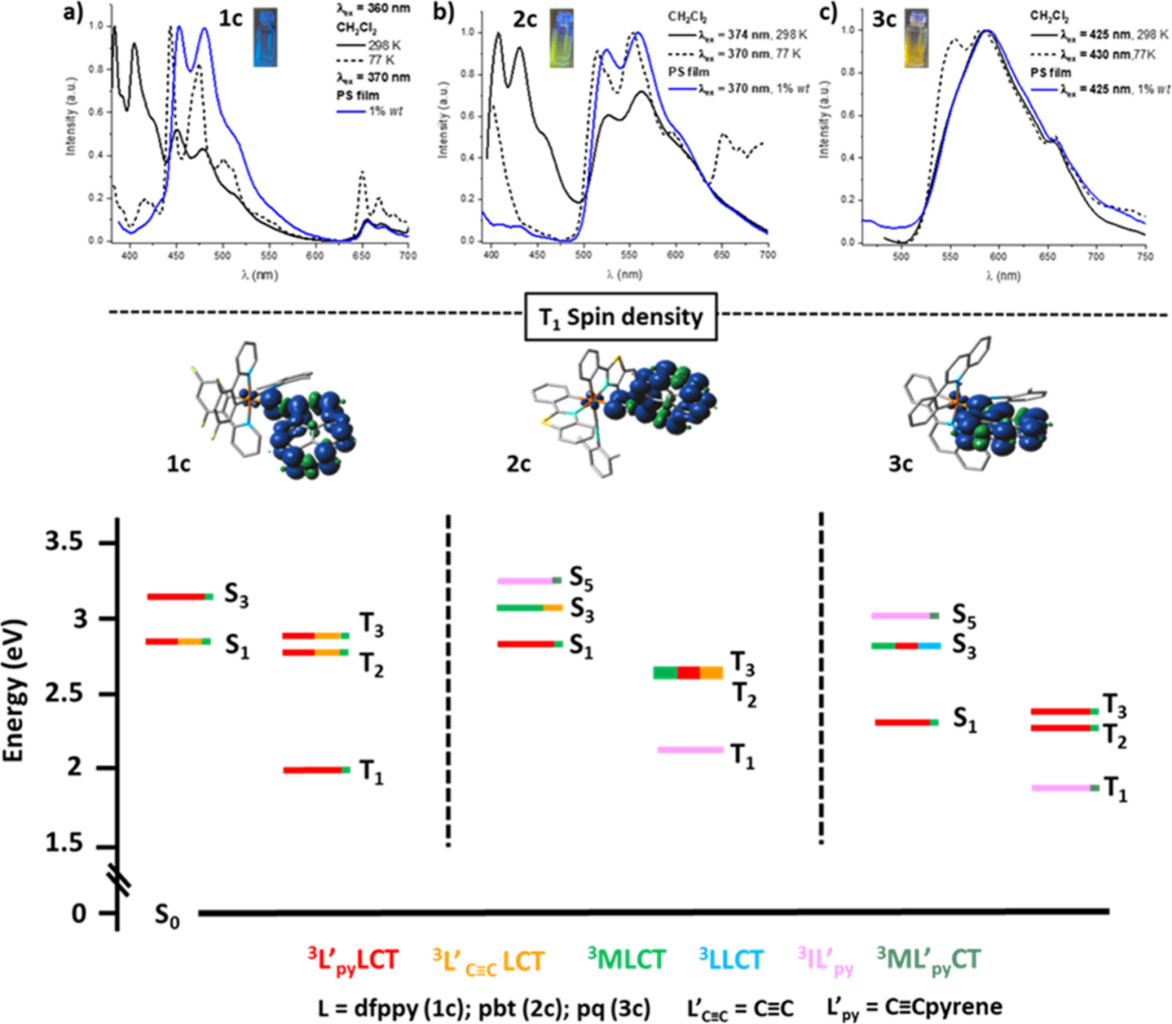
Emission spectra in deaerated CH_2_Cl_2_ solution at 298 K and 77 K and in PS 1 wt % of (a) *N,N-trans* **- 1c** (λ_ex_ = 390 nm, see Figure S68a), (b) **2c** (λ_ex_ = 320–420 nm), and (c) **3c** (λ_ex_ = 374–430 nm) (upper panel). Representation of T_1_ spin density and the contribution of different ligands in the selected triplet and singlet states (lower panel).

For pbt/pyrene *N,N-trans* **-2c**, the emission profile does not depend on λ_exc_ (range 320–420 nm). In glassy CH_2_Cl_2_ (77 K), a dual emission formed by the structured phosphorescence associated with the “Ir­(pbt)_2_(C≡C)” core in the green region (λ_max_ 520 nm, τ 0.89 μs) and the emission from ^3^IL^′^_pyr_ in the red region (650 nm) are observed. Upon increasing the temperature to 298 K, the red emission is essentially lost and only fluorescence located on C≡C-pyr (^1^IL^′^_pyr_, 405 nm, τ 0.04 μs) and the phosphorescence of the “Ir­(pbt)_2_(C≡C)” core are observed. According to calculations, the small energy gap between S_1_ and the T_2,3_ excited states (∼0.18 eV) seems to favor the population of ^1^IL^′^_pyr_ through efficient energy transfer at room temperature. Indeed, in a rigid PS system, only the structured emission at 520 nm ascribed to phosphorescence of the “Ir­(pbt)_2_(C≡C)” fragment is visible (Figure 8b). The emission spectra of **2c** were also recorded in solvents of different polarity [CH_3_CN (ε = 36.64) and toluene (ε = 2.38)] at room temperature. We observe the same pattern and we do not note shifts of the emission bands with changing solvent polarity (Figure S69). As expected, for pq/pyrene complex *N,N-trans* **-3c**, the two low-lying chromophores are closer in energy and overlap. Similar to the related **3a, b** complexes, this complex displays in solution and film a broad band (588–589 nm), slightly structured at 77 K (glassy, 556 nm) due to ^3^L^′^_pyr_LCT/^3^MLCT. As shown in Figure 8c, this band overlaps, in the low energy region, with the ^3^IL^′^_pyr_ phosphorescence (shoulder at 656 nm).

In all cases, the measured lifetimes in the PS films increase slightly in relation to those in solution, and the yields are relatively low, likely due to the ready availability of nonradiative pathways in these systems (Table 1). We would like to note that obtaining room-temperature phosphorescence in aromatic fluorophores is rather rare mainly due to the low spin–orbit coupling effect of aromatic fluorophores (π–π* transitions) and the large singlet–triplet energy gap, which makes S_1_→T_1_ intersystem crossing (ISC) difficult. However, the generation of triplet pyrene (^3^pyrene) and dual fluorescence/phosphorescence excited states has been reported previously in cyclometalated d^6^ Ir­(III) or Pt­(IV)^23^ and d^8^ Pt­(II) complexes.^24^

### Electrochemical Properties

The electrochemical activity of ***N,N-trans*** complexes **1c**, **2a**, **2b**, **2c**, and **3c** was determined in CH_2_Cl_2_ using tetrabutylammonium hexafluorophosphate as the supporting electrolyte (electrochemical window +1.8 to −1.8 V), and the results are collected in Table 2, presented vs Ag/AgCl reference electrode, and in Figure S70. Complexes **2a** and **2b** exhibit an irreversible Ir^III/IV^ oxidation wave at 0.94 V, demonstrating that variation in the alkynyl ligand structure has no electronic effect on the redox activity of the Ir­(III) center. No cathodic redox activity could be observed for these complexes until the edge of the solvent window. These results have been observed in other “Ir­(C^N)_2_” derivatives.^23c,25^ The cyclic voltammograms of pyrene complexes **1c**, **2c**, and **3c** show metal-based irreversible oxidation waves at 0.87 V **1c**, 0.83 V **2c**, and 0.80 V **3c** and an additional process with respect to **2a** and **2b**, due to the redox activity of the pyrene alkynyl substituent, which showed an irreversible oxidation peak at 1.29 V **1c**, 1.34 V **2c**, and 1.38 V **3c** (Figure S70). These results are in line with the experimental data published of free 1-ethynylpyrene,^23c^ which exhibits irreversible oxidation to +1.30 V and reduction at −2.02 V vs SCE and in other pyrene-linked Ir­(III) complexes.^23c^ It is reasonable to assume that the reduction of our complexes surely falls outside our electrochemical solvent window.

**Table 2:** Electrochemical Data^a^ and LUMO Energy Estimations for Selected ***N,N-trans*** Complexes

compound	**1c**	**2a**	**2b**	**2c**	**3c**
*E* ^ox^ (V)^b^	0.87^c^	0.94^c^	0.94^c^	0.83^c^	0.80^c^
	1.29^d^			1.34^d^	1.38^d^
*E*_onset,ox_(V)	0.79	0.85	0.82	0.71	0.70
*E*_HOMO_ (eV)^e^	–4.94	–5.16	–5.12	–5.04	–4.85
*E*_HOMO_ (eV) calc^f^	–5.44	–5.50	–5.47	–5.36	–5.35

a All measurements were carried out at 298 K in a 0.1 M solution of (NBu_4_)­PF_6_ in dry CH_2_Cl_2_ at 100 mVs^–1^ vs Ag/AgCl reference electrode.

b Irreversible anodic peak potentials.

c Redox processes associated with the iridium center.

d Redox processes associated with pyrene.

e Estimated HOMO energy by electrochemistry data [E_HOMO_ = −(E_onset,ox_ + 5.1 – E_Fc/Fc_^+^)].

f Estimated HOMO energies by DFT calculations.

The HOMO energy levels were also estimated from the oxidation potentials data by using the equation E_HOMO_ = −(E_onset,ox_ + 5.1 – E_Fc/Fc_^+^),^26^ where 0.45 V is the potential of ferrocene vs Ag/AgCl, and 5.1 eV is the energy level of ferrocene to the vacuum energy level (Table 2). The calculated HOMO energies are very similar between them (−5.35 to −5.50 eV) and follow the same tendency as the HOMO energies estimated by DFT calculations (*N,N-trans* **-2a** > **2b** > **2c** > **1c** > **3c**).

## Conclusions

The synthesis, structural characterization, and photophysical properties of a series of heteroleptic bis-cyclometalated CNXyl/alkynyl Ir­(III) complexes [Ir­(C^N)_2_(CNXyl)­(C≡CR)] [C^N = dfppy **1**, pbt **2**, pq **3**; R = Tol **a**, C_6_H_2_(OMe)_3_ **b**, and pyrene **c**], obtained from the corresponding [Ir­(C^N)_2_(CNXyl)­Cl] (**1**_**Cl**_, **2**_**Cl**_, and **3**_**Cl**_), are reported. In the case of dfppy, we have obtained not only the typical ***N,N-trans-*** [Ir­(dfppy)_2_(CNXyl)­Cl] (*N,N-trans-* **1**_**Cl**_) complex but also three additional geometrical isomers (***N,N-cis***-(*OC* **-6–24)**/(*OC* **-6–44)-1**_**Cl**_ and *C,C-trans* **-1**_**Cl**_), about which computational data reveals the following order of stability: *N,N-cis* **-(** *OC* **-6–44)-1**_**Cl**_ > *N,N-trans* **-1**_**Cl**_ > *N,N-cis* **-(** *OC* **-6–24)-1**_**Cl**_ ≫ *C,C-trans-* **1**_**Cl**_. Such geometric control opened a route to three different diastereomers of the complexes [Ir­(dfppy)_2_(CNXyl)­(C≡CR)] (R = Tol **a**, C_6_H_2_(OMe)_3_ **b**) (*N,N-trans* **-1** *a* **/1b**, *N,N-cis* **-(** *OC* **-6–32)**- **1** **a** **/1b**, and *C,C-trans* **-1a/1b**). In general, the geometrical isomers of each complex show similar photophysical properties independently of the mutual orientation of the ligands, although with significantly lower efficiencies of ***N,N-cis*** and ***C,C-trans*** isomers with respect to ***N,N-trans***. The incorporation of the electron donor alkynyl ligands modifies the nature of the low-lying excited state from ^3^ILCT/^3^MLCT in the Cl/CNXyl complexes to a lowest excited state having ^3^L^′^LCT/^3^MLCT (Ir/C≡CR→ C^N) characteristics in the C≡CR (**a** or **b**)/CNXyl complexes, and contrary to expectations, their incorporation does not enhance luminescence efficiency. The cyclometalated C^N ligand has a decisive influence on the emission wavelength, while alkynyl ligand **a** or **b** alters the molecular frontier orbital distributions, thereby affecting the luminous efficiency. With strongly delocalized pyrene fluorophores connected to the alkynyl ligand, there are three distinct excited state behaviors among the complexes described **1c**, **2c**, and **3c**, depending on the complex, media, and, on occasions, the excitation wavelength. A dual fluorescence (^1^IL^′^_py__r_)/phosphorescence (^3^IL^′^_pyr_) is located on the pyrene group and a phosphorescence is associated with ^3^L^′^_py__r_LCT/^3^MLCT, ascribed to the “Ir­(C^∧^N)_2_(C≡C)” fragment. Overall, this study provides valuable structure–property relationships for Ir­(III) complexes with a three-ligand molecular framework and underscores the importance of ligand design, stereochemistry, and excited-state dynamics in the development of advanced luminescent materials.

## Experimental Section

### General Comments

All reactions were carried out under an atmosphere of dry argon, using standard Schlenk techniques. Solvents were obtained from a solvent purification system (M-BRAUN MS SPS-800). Elemental analyses were carried out with an EA FLASH 2000 (Thermo Fisher Scientific) microanalyzer. Mass spectra were recorded by electrospray ionization on an interphase ESI/APCI Bruker Microtof-Q spectrometer with positive ion mode, with MeOH/H_2_O 90/10 and 0.1% formic acid as a mobile phase and in a quadrupole time-of-flight (QTOF) mass spectrometer, Bruker Impact II, equipped with a Bruker Apollo II electrospray ionization source. IR spectra were obtained on a Fourier Transform PerkinElmer Spectrum UATR Two spectrophotometer, with the diamond crystal ATR attachment, which covers the region between 4000 and 450 cm^–1^; data processing was carried out with Omnic. NMR spectra were recorded on a Bruker AVANCE ARX 400 spectrometer at 298 K. Chemical shifts are reported in parts per million (ppm) relative to external standards (SiMe_4_ for ^1^H and ^13^C­{^1^H} and CFCl_3_ for ^19^F­{^1^H}), and all coupling constants are given in hertz (Hz). The UV–vis absorption spectra were measured with a Hewlett-Packard 8453 spectrophotometer. Excitation and emission spectra were obtained with Shimazdu RF-60000 and Edinburg FLS 1000 spectrofluorimeters, respectively. The lifetime measurements were performed with a Jobin Yvon Horiba Fluorolog operating in the phosphorimeter mode (with an F1–1029 lifetime emission PMT assembly, using a 450 W Xe lamp) or with a Datastation HUB-B with a nanoLED controller and software DAS6. The nanoLEDs employed for lifetime measurements have 370 or 390 nm wavelengths with pulse lengths of 1.1–1.2 ns. Quantum yields were measured with a Hamamatsu Absolute PL Quantum Yield Measurement System; the estimated uncertainty is ±5% or better. Cyclic voltammograms were registered on a potentiostat Voltalab PST 050 with the CV cell consisting of a platinum disk as the working electrode, a Pt wire as the counter electrode, and an Ag/AgCl reference electrode. The measurements were carried out at 298 K under a N_2_ atmosphere, using degassed 5 × 10^–4^ M solutions of the complexes in dry CH_2_Cl_2_ and 0.1 M (NBu_4_)­PF_6_ as the supporting electrolyte. The ferrocene/ferricinium couple served as the internal reference (+0.45 V vs Ag/AgCl). 2,6-Dimethylphenylisocyanide (CNXyl) and different commercially available alkynyls were used as received. The dichloro-bridged dimers [Ir­(C^N)_2_(μ-Cl)]_2_ ^10b,17^ and *N,N* **-** *trans* **-**[Ir­(pbt)_2_(CNXyl)­Cl] (**2**_**Cl**_)^8b^ were synthesized following literature methods. No uncommon hazards are noted.

### Synthesis of the Isomers of [Ir­(dffpy)_2_(CNXyl)­Cl] (**1**_**Cl**_)

#### Synthesis of *N*,*N*-*trans*-**(*OC*-6–42)-1**_**Cl**_ (Major) and *N*,*N*-*cis* **-(*OC*-6–24)-1**_**Cl**_ (Minor)

A solution of [Ir­(dfppy)_2_(μ-Cl)]_2_ (0.142 g, 0.117 mmol) and (2,6-dimethylphenyl)­isocyanide (0.029 g, 0.222 mmol) in CH_2_Cl_2_ (25 mL) was stirred for 2 h at room temperature and filtered through Celite. The filtrate was evaporated to dryness and treated with *n*-hexane (10 mL) to give a solid (0.1334 g) that resulted to be a mixture of isomers, which were separated by column chromatography using CH_2_Cl_2_:MeOH (95:5) as the eluent. Two different isomers were isolated: *N,N* **-** *trans* **-(** *OC* **-6–42)-1**_**Cl**_ as the major species (40%) and the minor isomer *N,N* **-** *cis* **-(** *OC* **-6–24)-1**_**Cl**_ (23%).

Major isomer, *N,N* **-** *trans* **-(** *OC* **-6–42)-1**_**Cl**_: ESI (+): *m*/*z* (%) 704.1 [M–Cl]^+^ (100). ^1^H NMR (400 MHz, CD_3_CN): δ 9.88 (dd, ^3^ *J*_H–H_ = 5.8, ^4^ *J*_H–H_ = 1, H^2/2′^), 9.32 (dd, ^3^ *J*_H–H_ = 5.9, ^4^ *J*_H–H_ = 1, H^2′/2^), 8.34 (t, ^3^ *J*_H–H_ = 6.9, H^5,5′^), 8.07–8.02 (m, H^4,4′^), 7.47 (td, ^3^ *J*_H–H_ = 6.8, ^4^ *J*_H–H_ = 1.2, H^3/3′^), 7.37 (td, ^3^ *J*_H–H_ = 6.6, ^4^ *J*_H–H_ = 1.2, H^3′/3^), 7.21 (t, ^3^ *J*_H–H_ = 7.5, H^*p*^), 7.12 (d, ^3^ *J*_H–H_ = 7.8, 2H, H^*m*^), 6.59–6.49 (m, H^9,9′^), 5.90 (dd, ^3^ *J*_H–F_ = 8.8, ^4^ *J*_H–H_ = 2.4, H^11′/11^), 5.55 (dd, ^3^ *J*_H–F_ = 8.4, ^4^ *J*_H–H_ = 2.4, H^11/11′^), 2.15 (s, 6H, Me _Xyl_). ^19^F­{^1^H} NMR (376.5 MHz, CD_3_CN): δ −109.5 (d, ^4^ *J*_F–F_ = 9.9, F^10/10′^), −109.7 (d, ^4^ *J*_F–F_ = 10.0, F^10′/10^), −111.1 (d, ^4^ *J*_F–F_ = 9.9, F^8/8′^), −111.2 (d, ^4^ *J*_F–F_ = 9.9, F^8′/8^).

Minor isomer, *N,N* **-** *cis* **-(** *OC* **-6–24)-1**_**Cl**_: ESI (+): *m*/*z* (%) 704.1 [M–Cl]^+^ (100). ^1^H NMR (400 MHz, CDCl_3_): δ 9.32 (dd, ^3^ *J*_H–H_ = 5.6, ^4^ *J*_H–H_ = 1.0, H^2/2′^), 8.44 (dd, ^3^ *J*_H–H_ = 8.2, ^4^ *J*_H–H_ = 2.4, H^5/5′^), 8.19 (d, ^3^ *J*_H–H_ = 8.5, H^2′/2^), 8.04 (dd, ^3^ *J*_H–F_ = 9.0, ^4^ *J*_H–H_ = 2.4, H^11/11′^), 7.93 (td, ^3^ *J*_H–H_ = 7.9, ^4^ *J*_H–H_ = 2.4, H^4/4′^), 7.67 (dd, ^3^ *J*_H–H_ = 5.9, ^4^ *J*_H–H_ = 1.0, H^5′/5^), 7.59 (t, ^3^ *J*_H–H_ = 7.9, H^4′/4^), 7.33 (td, ^3^ *J*_H–H_ = 6.6, ^4^ *J*_H–H_ = 1.0, H^3/3′^), 7.03 (t, ^3^ *J*_H–H_ = 7.6, H^*p*^), 7.03 (d, ^3^ *J*_H–H_ = 7.6, 2H, H^*m*^), 6.76 (td, ^3^ *J*_H–H_ = 6.9, ^4^ *J*_H–H_ = 1.2, H^3′/3^), 6.71 (td, ^3^ *J*_H–F_ = 10.7, ^4^ *J*_H–H_ = 2.5, H^9/9′^), 6.36 (td, ^3^ *J*_H–F_ = 10.9, ^4^ *J*_H–H_ = 2.5, H^9′/9^), 6.13 (dd, ^3^ *J*_H–F_ = 8.5, ^4^ *J*_H–H_ = 2.4, H^11′/11^), 2.18 (s, 6H, Me _Xyl_). ^19^F­{^1^H} NMR (376.5 MHz, CDCl_3_): δ −105.8 (d, ^4^ *J*_F–F_ = 10.2, F^10/10′^), −107.6 (d, ^4^ *J*_F–F_ = 10.2, F^10′/10^), −109.4 (d, ^4^ *J*_F–F_ = 10.4, F^8/8′^), −111.2 (d, ^4^ *J*_F–F_ = 10.3, F^8′/8^).

#### Synthesis of *N*,*N*-*cis*-**(*OC*-6–44)-1**_**Cl**_

This species was obtained by the reaction of carbazole (0.035 g, 0.206 mmol) with *n*-butyllithium 1.6 M (130 μL, 0.206 mmol) in THF at −30 °C, followed by the addition of isomer *N,N* **-** *trans* **-(** *OC* **-6–42)-1**_**Cl**_ (0.153 g, 0.206 mmol). After reaching room temperature, the reaction mixture was gradually heated to reflux and stirred under these conditions for 8 h. The solvent was removed under reduced pressure, and the resulting residue was treated with isopropanol (5 mL) to give isomer *N,N* **-** *cis* **-(** *OC* **-6–44)-1**_**Cl**_ as a pale solid (0.121 g, 79%). ESI (+): *m*/*z* (%) 704.1 [M–Cl]^+^ (100). ^1^H NMR (400 MHz, CDCl_3_): δ 9.94 (d, ^3^ *J*_H–H_ = 5.4, H^2/2′^), 8.39 (d, ^3^ *J*_H–H_ = 8.5, H^5/5′^), 8.23 (d, ^3^ *J*_H–H_ = 8.5, H^2′/2^), 8.00 (d, ^3^ *J*_H–H_ = 7.8, H^4/4′^), 7.66 (td, ^3^ *J*_H–H_ = 7.9, ^4^ *J*_H–H_ = 2.4, H^4′/4^), 7.55 (dd, ^3^ *J*_H–F_ = 8.6, ^4^ *J*_H–H_ = 2.3, H^11/11′^), 7.48 (d, ^3^ *J*_H–H_ = 6.5, H^3/3′^), 7.29 (d, ^3^ *J*_H–H_ = 5.7, H^5′/5^) 7.10 (t, ^3^ *J*_H–H_ = 7.4, H^*p*^), 7.01 (d, ^3^ *J*_H–H_ = 7.7, 2H, H^*m*^), 6.87 (td, ^3^ *J*_H–H_ = 6.7, H^3′/3^), 6.63 (td, ^3^ *J*_H–F_ = 10.9, ^4^ *J*_H–H_ = 2.3, H^9/9′^), 6.45 (td, ^3^ *J*_H–F_ = 10.8, ^4^ *J*_H–H_ = 2.5, H^9′/9^), 6.33 (dd, ^3^ *J*_H–F_ = 8.8, ^4^ *J*_H–H_ = 2.2, H^11′/11^), 2.15 (s, 6H, Me Xyl). ^19^F­{^1^H} NMR (376.5 MHz, CDCl_3_): δ −107.2 (d, ^4^ *J*_F–F_ = 10.1, F^10/10′^), −107.6 (d, ^4^ *J*_F–F_ = 10.4, F^10′/10^), −109.3 (d, ^4^ *J*_F–F_ = 10.1, F^8/8′^), −109.4 (d, ^4^ *J*_F–F_ = 10.4, F^8′/8^).

#### Synthesis of *N*,*N*-*cis* **-(*OC*-6–23)-1**_**Cl**_

A solution of the *N,N* **-** *trans* **-(** *OC* **-6–42)-1**_**Cl**_ isomer **(**2.5 mM in 500 μL of CD_3_CN) was irradiated with a Kessil lamp (370 nm, 40 W) located 5 cm apart from the tube for 5 min. This procedure resulted in the formation of isomer *N,N* **-** *cis* **-(** *OC* **-6–23)-1**_**Cl**_ in 83%. ESI (+): *m*/*z* (%) 704.1 [M–Cl]^+^ (100). ^1^H NMR (400 MHz, CD_3_CN): δ 9.30 (d, ^3^ *J*_H–H_ = 5.6, H^2/2′^), 9.15 (d, ^3^ *J*_H–H_ = 5.9, H^2′/2^), 8.40 (t, ^3^ *J*_H–H_ = 9.0, H^5,5′^), 8.16 (t, ^3^ *J*_H–H_ = 10.4, H^4,4′^), 7.53 (td, ^3^ *J*_H–H_ = 6.7, ^4^ *J*_H–H_ = 1.4, H^3′/3^), 7.47 (td, ^3^ *J*_H–H_ = 6.7, ^4^ *J*_H–H_ = 1.3, H^3/3′^), 7.26 (t, ^3^ *J*_H–F_ = 7.6, H^*p*^), 7.15 (d, ^3^ *J*_H–F_ = 7.6, 2H, H^*m*^), 6.66–6.63 (td, ^3^ *J*_H–F_ = 10.5, ^4^ *J*_H–H_ = 2.6, H^9,9′^), 5.74 (dd, ^3^ *J*_H–H_ = 8.7, ^4^ *J*_H–H_ = 2.5, H^11′/11^), 5.61 (dd, ^3^ *J*_H–H_ = 8.3, ^4^ *J*_H–H_ = 2.4, H^11/11′^), 2.13 (s, 6H, Me _Xyl_). ^19^F­{^1^H} NMR (376.5 MHz, CD_3_CN): δ −108.1 (d, ^4^ *J*_F–F_ = 10.4, F^10/10′^), −108.2 (d, ^4^ *J*_F–F_ = 10.1, F^10′/10^), −109.9 (d, ^4^ *J*_F–F_ = 10.4, F^8′/8^), −110.2 (d, ^4^ *J*_F–F_ = 10.4, F^8/8′^).

#### Synthesis of *N*,*N*-*trans*-[Ir­(pq)_2_(CNXyl)­Cl] (**3**_**Cl**_)

A mixture of [Ir­(pq)_2_(μ-Cl)]_2_ (0.102 g, 0.0799 mmol) and (2,6-dimethylphenyl)­isocyanide (0.024 g, 0.184 mmol) in toluene (25 mL) was refluxed for 2 h. The orange resulting solution was filtered, evaporated to dryness, and treated with *n*-hexane (2 × 10 mL) to obtain **3**_**Cl**_ as an orange solid (0.043 g, 70%). Elem. Anal. Calcd for C_39_H_29_ClIrN_3_ (767.35): C, 61.05; H, 3.81; N, 5.48. Found: C, 61.13; H, 4.30; N, 5.12. ESI (+): *m*/*z* (%) 732.215 [M–Cl]^+^ (100). IR (cm^–1^): ν­(C≡N) 2131 (vs). ^1^H NMR (400 MHz, CDCl_3_): δ 10.62 (d, ^3^ *J*_H–H_ = 8.7, H^8/8′^), 9.30 (d, ^3^ *J*_H–H_ = 8.6, H^8′/8^), 8.30 (d, ^3^ *J*_H–H_ = 8.6, H^4/4′^), 8.25 (d, ^3^ *J*_H–H_ = 8.5, H^4′/4^), 8.03 (d, ^3^ *J*_H–H_ = 8.7, 2H, H^3, 3′^), 7.87–7.83 (m, 4H, H^5, 5′, 7, 7′^), 7.69 (d, ^3^ *J*_H–H_ = 7.1, H^9/9′^), 7.65 (d, ^3^ *J*_H–H_ = 7.5, H^9′/9^), 7.62 (t, ^3^ *J*_H–H_ = 7.5, H^6/6′^), 7.55 (t, ^3^ *J*_H–H_ = 7.5, H^6′/6^), 6.97 (t, ^3^ *J*_H–H_ = 7.4, H^*p*^), 6.88–6.80 (m, 4H, H^10, 10′, *m*^), 6.75 (t, ^3^ *J*_H–H_ = 7.1, H^11/11′^), 6.66 (t, ^3^ *J*_H–H_ = 7.2, H^11′/11^), 6.20 (d, ^3^ *J*_H–H_ = 7.5, H^12, 12′^), 1.85 (s, 6H, Me _Xyl_). ^13^C­{^1^H} NMR (100.6 MHz, CDCl_3_): δ 171.6 (s, C^2/2′^), 170.3 (s, C^2′/2^), 166.5 (s, C^13′/13^), 150.0 (s, C^8a/8a′^), 148.2 (s, C^8a′/8a^), 147.1 (s, C^14/14′^), 145.7 (s, C^14′/14^), 145.4 (s, C^13′/13^), 139.2 (s, C^4, 4′^), 135.2 (s, C^*o*^), 132.3 (s, C^12/12′^), 132.0 (s, C^12′/12^), 131.9 (s, C^7/7′^), 131.6 (s, C^7′/7^), 130.9–130.8 (s, C^8, 8′^), 130.6 (s, C^11/11′^), 130.4 (s, C^11′/11^), 128.8 (s, C^ipso^), 128.4 (s, C^*p*^_CNXyl_), 127.3 (s, C^4*a*/4a′^), 128.0 (s, C^4a′/4a^), 127.7 (s, C^*m*^_Xyl_), 127.5 (s, C^5, 5′^), 127.2 (s, C^6/6′^), 126.4 (s, C^6′/6^), 126.1 (m, C^9/9′^), 125.9 (s, C^9′/9^), 123.0 (s, C^10/10′^), 121.8 (s, C^10′/10^), 118.2 (s, C^3/3′^), 116.9 (s, C^3′/3^), 18.4 (s, Me _Xyl_).

### Synthesis of the Isomers of [Ir­(dffpy)_2_(CNXyl)­(C≡C-Tol)] (**1a**)

#### Synthesis of *N,N*-*trans* **-(*OC*-6–13)-1a**

A suspension of *N,N* **-** *trans* **-(** *OC* **-6–42)-1**_**Cl**_ **(**0.156 g, 0.211 mmol) and 4-ethynyltoluene silver acetylide (0.052 g, 0.232 mmol) was refluxed in 1,2-dichloroethane (20 mL) for 15 h. The suspension was filtered through Celite. The filtrate was evaporated to dryness and treated with *n*-hexane (20 mL) to obtain *N,N* **-** *trans* **-(** *OC* **-6–13)-1a** as a yellow solid (0.116 g, 67%). Elem. Anal. Calcd for C_40_H_28_F_4_IrN_3_ (818.90): C, 58.67; H, 3.45; N, 5.15. Found: C, 59.14; H, 3.71; N, 4.66. ESI (+): *m*/*z* (%) 704.155 [M–C≡CR]^+^ (100), 820.220 [M + H]^+^ (8). IR (cm^–1^): ν­(C≡N) 2141 (vs), ν­(C≡C) 2095 (s). ^1^H NMR (400 MHz, CDCl_3_): δ 10.19 (d, ^3^ *J*_H–H_ = 5.9, H^2/2′^), 9.20 (d, ^3^ *J*_H–H_ = 6.1, H^2́/2^), 8.33 (d, ^3^ *J*_H–H_ = 8.1, H^5/5′^), 8.29 (d, ^3^ *J*_H–H_ = 8.2, H^5́/5^), 7.86 (t, ^3^ *J*_H–H_ = 7.7, H^4/4′^), 7.83 (t, ^3^ *J*_H–H_ = 7.9, H^4́/4^), 7.24 (t, ^3^ *J*_H–H_ = 6.9, H^3/3′^), 7.15–7.08 (m, 3H, H^3́/3, *p*, 2″^), 7.03 (d, ^3^ *J*_H–H_ = 7.4, 2H, H^*m*^), 6.92 (d, ^3^ *J*_H–H_ = 7.9, H^3″^), 6.38 (td, ^3^ *J*_H–F_ = 10.5, ^4^ *J*_H–H_ = 2.5, 2H, H^9,9́^), 5.89 (dd, ^3^ *J*_H–F_ = 7.8, ^4^ *J*_H–H_ = 2.1, H^11/11′^), 5.67 (dd, ^3^ *J*_H–F_ = 8.4, ^4^ *J*_H–H_ = 2.1, H^11́/11^), 2.25 (s, 3H, Me _Tol_), 2.19 (s, 6H, Me _Xyl_). ^13^C­{^1^H} NMR (100.6 MHz, CDCl_3_): δ 166.2 (d, ^3^ *J*_C–F_ = 7.2, C^6/6′^), 165.1 (d, ^3^ *J*_C–F_ = 7.1, C^6′/6^), 162.6 (d, ^3^ *J*_C–F_ = 10.7, C^10/10′^), 162.1 (d, ^3^ *J*_C–F_ = 10.9, C^10′/10^), 160.5 (d, ^3^ *J*_C–F_ = 11.8, C^8/8′^), 160.4 (d, ^3^ *J*_C–F_ = 11.8, C^8′/8^), 154.2 (s, C^2/2′^), 153.1 (s, C^2′/2^), 137.9 (s, C^4/4′^), 136.9 (s, C^4′/4^), 135.2 (s, C^*o*^_Xyl_) 134.2 (s, C^4″^), 131.7 (s, C^2″^), 128.9 (s, C^*p*^_Xyl_), 128.7 (s, C^3″^), 128.3 (s, C^7/7′^), 128.0 (s, C^*m*^_Xyl_), 127.4 (s, C^7′/7^), 126.4 (s, C^1″^), 123.8 (d, ^4^ *J*_C–F_ = 21.2, C^5/5′^), 123.1 (d, ^4^ *J*_C–F_ = 21.4, C^5′/5^), 122.8 (s, C^3, 3′^), 114.0–113.8 (dd, ^2^ *J*_C–F_ = 16.1, ^4^ *J*_C–F_ = 3.2, C^11/11′^), 112.1–112.0 (dd, ^2^ *J*_C–F_ = 16.3, ^4^ *J*_C–F_ = 3.2, C^11′/11^), 104.5 (s, C^β^), 100.3 (s, C^α^), 98.1 (t, ^2^ *J*_C–F_ = 26.8, C^9/9′^), 98.0 (t, ^2^ *J*_C–F_ = 27.2, C^9′/9^), 21.3 (s, Me_Tol_), 18.6 (s, 6C, Me_Xyl_). ^19^F­{^1^H} NMR (376.5 MHz, CDCl_3_): δ −108.7 (d, ^4^ *J*_F–F_ = 9.7, F^10/10′^), −108.8 (d, ^4^ *J*_F–F_ = 9.7, F^10′/10^), −110.4 (d, ^4^ *J*_F–F_ = 9.7, F^8′/8^), −110.7 (d, ^4^ *J*_F–F_ = 9.7, F^8/8′^).

#### Synthesis of *N*,*N*-*cis*-**(*OC*-6–32)-1a**

A suspension of *N,N* **-** *cis* **-(** *OC* **-6–44)-1**_**Cl**_ (0.051 g, 0.070 mmol) and 4-ethynyltoluene silver acetylide (0.019 g, 0.083 mmol) was refluxed in 1,2-dichloroethane (20 mL) for 15 h. The suspension was filtered through Celite, and the filtrate was evaporated to dryness and treated with *n*-hexane (10 mL) to obtain *N,N* **-** *cis* **-(** *OC* **-6–32)-1a** as a yellow solid (0.030 g, 52%). ESI (+): *m*/*z* (%) 704.1 [M–C≡CR]^+^ (100), 820.2 [M + H]^+^ (0.6%). ^1^H NMR (400 MHz, CDCl_3_): δ 10.07 (d, ^3^ *J*_H–H_ = 5.5, H^2/2′^), 8.39 (dd, ^3^ *J*_H–H_ = 8.3, ^4^ *J*_H–H_ = 1.8, H^5/5′^), 8.22 (d, ^3^ *J*_H–H_ = 5.5, H^2′/2^), 7.95 (t, ^3^ *J*_H–H_ = 8.1, H^4/4′^), 7.62 (t, ^3^ *J*_H–H_ = 8.1, H^3′/3^), 7.53 (dd, ^3^ *J*_H–F_ = 9.0, ^4^ *J*_H–H_ = 2.2, H^11/11′^), 7.39–7.36 (m, H^3/3′, 5′/5^), 7.12–7.06 (m, 3H, H^2″, *p*^), 7.01 (d, ^3^ *J*_H–F_ = 7.6, 2H, H^*m*^), 6.93 (d, ^3^ *J*_H–F_ = 7.9, 2H, H^3″^), 6.82 (t, ^3^ *J*_H–H_ = 6.7, H^4′/4^), 6.60 (td, ^3^ *J*_H–F_ = 11.1, ^4^ *J*_H–H_ = 2.1, H^9/9′^), 6.40 (td, ^3^ *J*_H–F_ = 11.0, ^4^ *J*_H–H_ = 2.4, H^9′/9^), 6.32 (dd, ^3^ *J*_H–F_ = 8.1, ^4^ *J*_H–H_ = 2.3, H^11′/11^), 2.25 (s, 3H, Me _Tol_), 2.20 (s, 6H, Me _Xyl_). ^19^F­{^1^H} NMR (376.5 MHz, CDCl_3_): δ −108.6 (d, ^4^ *J*_F–F_ = 9.0, F^10/10′^), −108.9 (d, ^4^ *J*_F–F_ = 9.4, F^10′/10^), −109.8 (d, ^4^ *J*_F–F_ = 9.5, F^8/8′^), −110.3 (d, ^4^ *J*_F–F_ = 9.5, F^8′/8^).

#### Synthesis of *N*,*N*-*cis* **-(*OC*-6–42)-1a**

A CD_3_CN solution of *N,N* **-** *trans* **-(** *OC* **-6–13)-1a (**2.4 mM in 500 μL of CD_3_CN) was photoirradiated with a Kessil lamp (370 nm, 40 W) located 5 cm apart from the tube for 5 min, resulting in the quantitative formation of isomer *N,N* **-** *cis* **-(** *OC* **-6–42)-1a**. ESI (+): *m*/*z* (%) 704.1 [M–C≡CR]^+^ (100), 820.2 [M + H]^+^ (40%). ^1^H NMR (400 MHz, CD_3_CN): δ 9.33 (dd, ^3^ *J*_H–H_ = 5.6, ^4^ *J*_H–H_ = 1.1, H^2/2′^), 8.43 (d, ^3^ *J*_H–H_ = 8.3, H^5′/5^), 8.25 (d, ^3^ *J*_H–H_ = 8.3, H^2′/2^), 8.14–8.07 (m, H^4/4′,11/11′^), 7.78 (t, ^3^ *J*_H–H_ = 7.8, H^4′/4^), 7.71 (d, ^3^ *J*_H–H_ = 5.5, H^5′/5^), 7.46 (td, ^3^ *J*_H–H_ = 6.6, ^4^ *J*_H–H_ = 1.2, H^3/3′^), 7.18 (t, ^3^ *J*_H–F_ = 7.5, H^*p*^), 7.10 (d, ^3^ *J*_H–F_ = 7.6, 2H, H^*m*^), 7.00–6.89 (m, 5H, H^3′/3,2″,3″^), 6.78 (td, ^3^ *J*_H–F_ = 11.0, ^4^ *J*_H–H_ = 2.4, H^9/9′^), 6.50 (td, ^3^ *J*_H–F_ = 11.1, ^4^ *J*_H–H_ = 2.5, H^9′/9^), 6.09 (dd, ^3^ *J*_H–H_ = 8.5, ^4^ *J*_H–H_ = 2.5, H^11′/11^), 2.22 (s, 3H, Me _Tol_), 2.18 (s, 6H, Me _Xyl_). ^19^F­{^1^H} NMR (376.5 MHz, CD_3_CN): δ −110.0 (d, ^4^ *J*_F–F_ = 9.8, F^10/10′^), −110.8 (d, ^4^ *J*_F–F_ = 9.8, F^10′/10^), −110.9 (d, ^4^ *J*_F–F_ = 9.7, F^8′/8^), −111.8 (d, ^4^ *J*_F–F_ = 9.8, F^8/8′^).

### Synthesis of the Isomers of [Ir­(dffpy)_2_(CNXyl)­(C≡C–C_6_H_2_(OMe)_3_)] **(1b)**

#### Synthesis of *N*,*N*-*trans* **-(*OC*-6–13)-1b**

A suspension of *N,N* **-** *trans* **-(** *OC* **-6–42)-1**_**Cl**_ (0.201 g, 0.271 mmol) and AgC≡C–C_6_H_2_(OMe)_3_ (0.114 g, 0.380 mmol) was refluxed in 1,2-dichloroethane (20 mL) for 12 h. The suspension was filtered through Celite. The filtrate was evaporated to dryness, treated with toluene (20 mL), and filtered again. The soluble fraction was evaporated to small volume (2 mL) and treated with *n*-hexane (10 mL) to obtain *N,N* **-** *trans* **-(** *OC* **-6–13)-1b** as a yellow solid (0.102 g, 42%). Elem. Anal. Calcd for C_42_H_32_F_4_IrN_3_O_3_ (894.95): C, 56.37; H, 3.60; N, 4.70. Found: C, 55.89; H, 3.78; N, 4.31. ESI (+): *m*/*z* (%) 704.153 [M–C≡CR]^+^ (100), 896.238 [M + H]^+^ (10). IR (cm^–1^): ν­(C≡N) 2138 (vs), ν­(C≡C) 2093 (s). ^1^H NMR (400 MHz, CDCl_3_): δ 10.14 (d, ^3^ *J*_H–H_ = 5.5, H^2/2′^), 9.20 (d, ^3^ *J*_H–H_ = 6.2, H^2́/2^), 8.34 (d, ^3^ *J*_H–H_ = 7.8, H^5/5′^), 8.31 (d, ^3^ *J*_H–H_ = 7.9, H^5́/5^), 7.87 (t, ^3^ *J*_H–H_ = 7.7, H^4/4′^), 7.84 (t, ^3^ *J*_H–H_ = 7.8, H^4́/4^), 7.25 (t, ^3^ *J*_H–H_ = 6.7, H^3/3′^), 7.16–7.09 (m, 2H, H^3́/3, *p*^), 7.04 (d, ^3^ *J*_H–H_ = 7.5, 2H, H^*m*^), 6.45 (s, 2H, H^2″^), 6.39 (td, ^3^ *J*_H–F_ = 10.6, ^4^ *J*_H–H_ = 2.0, 2H, H^9,9́^), 5.88 (dd, ^3^ *J*_H–F_ = 7.8, ^4^ *J*_H–H_ = 1.9, H^11/11′^), 5.69 (dd, ^3^ *J*_H–F_ = 8.2, ^4^ *J*_H–H_ = 2.1, H^11́/11^), 3.77 (s, 9H, Me _C_≡_C_–_C6H2(OMe)3_), 2.20 (s, 6H, Me _Xyl_). ^13^C­{^1^H} NMR (100.6 MHz, CDCl_3_): δ 166.2 (s, C^6, 6′^), 164.8 (s, C^10, 10′^), 159.9 (s, C^8, 8′^), 154.1 (s, C^2/2′^), 153.7 (s, C^2′/2^), 152.6 (s, C^3″^), 138.0 (s, C^4/4′^), 137.0 (s, C^4′/4^), 136.3 (s, C^4″^), 135.0 (s, C^*o*^), 128.8 (s, C^*p*^), 128.0 (s, C^*m*^), 127.6 (s, C^7/7′^), 127.4 (s, C^7′/7^), 124.7 (s, C^1″^), 123.8 (d, ^4^ *J*_C–F_ = 21.0, C^5/5′^), 123.2 (d, ^4^ *J*_C–F_ = 21.0, C^5′/5^), 122.9 (s, C^3/3′^), 122.8 (s, C^3′/3^), 113.8 (dd, ^2^ *J*_C–F_ = 16.8, ^4^ *J*_C–F_ = 3.0, C^11/11′^), 112.0 (dd, ^2^ *J*_C–F_ = 16.5, ^4^ *J*_C–F_ = 2.9, C^11′/11^), 108.9 (s, C^2″^), 104.5 (s, C^β^), 101.0 (s, C^α^), 98.2 (t, ^2^ *J*_C–F_ = 26.9, C^9/9′^), 98.0 (t, ^2^ *J*_C–F_ = 27.0, C^9′/9^), 61.0 (s, 3C, C^O^–^Me^), 56.1 (s, 6C, C^O^–^Me^), 18.6 (s, 6C, Me _Xyl_). ^19^F­{^1^H} NMR (376.5 MHz, CDCl_3_): δ −104.9 (d, ^4^ *J*_F–F_ = 9.1, F^10/10′^), −105.1 (d, ^4^ *J*_F–F_ = 9.1, F^10′/10^), −106.1 (d, ^4^ *J*_F–F_ = 9.1, F^8′/8^), −106.6 (d, ^4^ *J*_F–F_ = 9.1, F^8/8′^).

#### Synthesis of *N*,*N*-*cis* **-(*OC*-6–32)-1b**

This isomer was obtained as a yellow solid (0.015, 56%) following the procedure described for *N,N* **-** *cis* **-(** *OC* **-6–32)-1a** using *N,N* **-** *cis* **-(** *OC* **-6–44)-1**_**Cl**_ (0.022 g, 0.031 mmol) and AgC≡C–C_6_H_2_(OMe)_3_ (0.011 g, 0.037 mmol). ESI (+): *m*/*z* (%) 704.1 [M–C≡CR]^+^ (100), 896.2 [M + H]^+^ (5.1). ^1^H NMR (400 MHz, CDCl_3_): δ 10.03 (dd, ^3^ *J*_H–H_ = 5.6, ^4^ *J*_H–H_ = 1.3, H^2/2′^), 8.40 (dd, ^3^ *J*_H–H_ = 8.4, ^4^ *J*_H–H_ = 2.1, H^5/5′^), 8.24 (dd, ^3^ *J*_H–H_ = 8.5, ^4^ *J*_H–H_ = 2.1, H^2′/2^), 7.97 (td, ^3^ *J*_H–H_ = 7.9, ^4^ *J*_H–H_ = 1.6, H^4/4′^), 7.64 (td, ^3^ *J*_H–H_ = 7.9, ^4^ *J*_H–H_ = 1.5, H^3′/3^), 7.53 (dd, ^3^ *J*_H–F_ = 8.9, ^4^ *J*_H–H_ = 2.4, H^11/11′^), 7.41–7.37 (m, H^3/3′, 5′/5^), 7.09 (t, ^3^ *J*_H–F_ = 7.4, H^*p*^), 7.02 (d, ^3^ *J*_H–F_ = 7.6, 2H, H^*m*^), 6.84 (td, ^3^ *J*_H–H_ = 6.7, ^4^ *J*_H–H_ = 1.2, H^4′/4^), 6.61 (td, ^3^ *J*_H–F_ = 11.0, ^4^ *J*_H–H_ = 2.3, H^9/9′^), 6.45 (s, 2H, H^2″^), 6.40 (td, ^3^ *J*_H–F_ = 11.9, ^4^ *J*_H–H_ = 2.5, H^9′/9^), 6.31 (dd, ^3^ *J*_H–F_ = 8.1, ^4^ *J*_H–H_ = 2.5, H^11′/11^), 3.76 (s, 9H, Me _C_≡_C_–_C6H2(OMe)3_), 2.20 (s, 6H, Me _Xyl_). ^19^F­{^1^H} NMR (376.5 MHz, CDCl_3_): δ −108.4 (d, ^4^ *J*_F–F_ = 9.8, F^10/10′^), −108.8 (d, ^4^ *J*_F–F_ = 10.1, F^10′/10^), −109.7 (d, ^4^ *J*_F–F_ = 10.1, F^8/8′^), −110.2 (d, ^4^ *J*_F–F_ = 10.1, F^8′/8^).

#### Synthesis of *N*,*N*-*cis* **-(*OC*-6–42)-1b**

A CD_3_CN solution [2.2 mM of *N,N* **-** *trans* **-(** *OC* **-6–13)-1b** in 500 μL] was photoirradiated as in isomer *N,N* **-** *cis* **-(** *OC* **-6–42)-1a**, resulting in the quantitative formation of *N,N* **-** *cis* **- (** *OC* **-6–42)-1b**. ESI (+): *m*/*z* (%) 704.1 [M–C≡CR]^+^ (100), 896.2 [M + H]^+^ (1.1). ^1^H NMR (400 MHz, CD_3_CN): δ 9.34 (d, ^3^ *J*_H–H_ = 5.7, H^2/2′^), 8.43 (d, ^3^ *J*_H–H_ = 8.7, H^5/5′^), 8.25 (d, ^3^ *J*_H–H_ = 8.3, H^2′/2^), 8.14–8.07 (m, 2H, H^4/4′,11/11′^), 7.78 (t, ^3^ *J*_H–H_ = 8.0, H^4′/4^), 7.71 (d, ^3^ *J*_H–H_ = 5.5, H^5′/5^), 7.46 (td, ^3^ *J*_H–H_ = 6.4, H^3/3′^), 7.19 (t, ^3^ *J*_H–F_ = 7.2, H^*p*^), 7.11 (d, ^3^ *J*_H–F_ = 7.6, 2H, H^*m*^), 7.00 (t, ^3^ *J*_H–F_ = 6.6, H^3′/3^), 6.78 (td, ^3^ *J*_H–F_ = 11.0, ^4^ *J*_H–H_ = 2.4, H^9/9′^), 6.51 (td, ^3^ *J*_H–F_ = 10.6, ^4^ *J*_H–H_ = 2.5, H^9′/9^), 6.28 (s, 2H, H^2″^), 6.09 (dd, ^3^ *J*_H–H_ = 8.7, ^4^ *J*_H–H_ = 2.6, H^11′/11^), 3.69 (s, 6H, Me _C≡C_–_C6H2(OMe)3_), 3.63 (s, 3H, Me _C≡C_–_C6H2(OMe)3_), 2.20 (s, 6H, Me _Xyl_). ^19^F­{^1^H} NMR (376.5 MHz, CD_3_CN): δ −110.0 (d, ^4^ *J*_F–F_ = 9.8, F^10/10′^), −110.8 (d, ^4^ *J*_F–F_ = 9.4, F^10′/10^), −109.9 (d, ^4^ *J*_F–F_ = 9.4, F^8′/8^), −110.2 (d, ^4^ *J*_F–F_ = 9.8, F^8/8′^).

#### Synthesis of *N*,*N*-*trans*-[Ir­(dffpy)_2_(CNXyl)­(C≡C-pyrene)] **(1c)**

Complex *N,N* **-** *trans* **-1c** was obtained as a yellow solid (0.198 g, 77%) following the procedure described for *N,N* **-** *trans* **-1a** using *N,N* **-** *trans* **-(** *OC* **-6–42)-1**_**Cl**_ (0.204 g, 0.276 mmol) and pyrene silver acetylide (0.106 g, 0.331 mmol). Elem. Anal. Calcd for C_49_H_32_F_4_IrN_3_ (931.03): C, 63.21; H, 3.46; N, 4.51. Found: C, 63.33; H, 3.82; N, 4.13. ESI (+): *m*/*z* (%) 704.129 [M–C≡CR]^+^ (100), 930.209 [M + H]^+^ (19). IR (cm^–1^): ν­(C≡N) 2148 (vs), ν­(C≡C) 2083 (s). ^1^H NMR (400 MHz, CDCl_3_): δ 10.28 (d, ^3^ *J*_H–H_ = 5.9, H^2/2′^), 9.32 (d, ^3^ *J*_H–H_ = 5.5, H^2́/2^), 8.41 (d, ^3^ *J*_H–H_ = 9.1, H^5/5′^), 8.34 (d, ^3^ *J*_H–H_ = 9.1, H^4/4′^), 8.31 (d, ^3^ *J*_H–H_ = 9.1, H^4́/4^), 8.05 (d, ^3^ *J*_H–H_ = 8.0, 2H, H^pyrene^, 7.96–7.84 (m, 7H, H^pyrene^), 7.78 (d, ^3^ *J*_H–H_ = 9.1, H^5́/5^), 7.22 (t, ^3^ *J*_H–H_ = 6.5, H^3/3′^), 7.16 (t, ^3^ *J*_H–H_ = 6.8, H^3́/3^), 7.12 (d, ^3^ *J*_H–H_ = 7.5, H^*p*^), 7.03 (d, ^3^ *J*_H–H_ = 7.7, 2H, H^*m*^), 6.50 (td, ^3^ *J*_H–F_ = 10.0, ^4^ *J*_H–H_ = 2, H^9/9′^), 6.44 (dd, ^3^ *J*_H–F_ = 9.8, ^4^ *J*_H–H_ = 1.9, H^9́/9^), 6.04 (dd, ^3^ *J*_H–F_ = 8.1, ^4^ *J*_H–H_ = 1.9, H^11/11′^), 5.84 (td, ^3^ *J*_H–F_ = 8.3, ^4^ *J*_H–H_ = 2.1, H^11́/11^), 2.23 (s, 6H, Me _Xyl_). ^13^C­{^1^H} NMR (100.6 MHz, CDCl_3_): δ 166.1 (d, ^3^ *J*_C–F_ = 7.2, C^6/6′^), 165.0 (d, ^3^ *J*_C–F_ = 7.2, C^6′/6^), 162.5 (d, ^3^ *J*_C–F_ = 11.5, C^10/10′^), 162.3 (d, ^3^ *J*_C–F_ = 11.5, C^10′/10^), 160.6 (d, ^3^ *J*_C–F_ = 9.7, C^8/8′^), 160.4 (d, ^3^ *J*_C–F_ = 10.0, C^8′/8^), 154.4 (s, C^2/2′^), 153.2 (s, C^2′/2^), 138.0 (s, C^4/4′^), 137.2 (s, C^4′/4^), 135.2 (s, C^o^_Xyl_), 132.1–124.3 (m, C^pyrene, *o*^_Xyl_), 128.5 (s, C^7/7′^), 128.0 (s, C^*m* xyl^), 127.6 (s, C^7′/7^), 123. Nine (d, ^4^ *J*_C–F_ = 21.0, C^5/5′^), 123.2 (d, ^4^ *J*_C–F_ = 21.0, C^5′/5^), 122.9 (s, C^3/3′^), 114.2 (dd, ^2^ *J*_C–F_ = 16.1, ^4^ *J*_C–F_ = 3.0, C^11/11′^), 112.3 (dd, ^2^ *J*_C–F_ = 16.8, ^4^ *J*_C–F_ = 3.2, C^11′/11^), 110.5 (s, C^α^), 103.9 (s, C^β^), 98.3 (t, ^2^ *J*_C–F_ = 27.0, 2C, C^9, 9′^), 19.2 (s, 6C, Me _Xyl_). ^19^F­{^1^H} NMR (376.5 MHz, CDCl_3_): δ −108.3 (d, ^4^ *J*_F–F_ = 9.5, F^10/10′^), −108.6 (d, ^4^ *J*_F–F_ = 9.5, F^10′/10^), −110.1 (d, ^4^ *J*_F–F_ = 9.5, F^8/8′^), −110.3 (d, ^4^ *J*_F–F_ = 9.5, F^8′/8^).

#### Synthesis of *N*,*N*-*trans*-[Ir­(pbt)_2_(CNXyl)­(C≡C-tol)] **(2a)**

A mixture of **2**_**Cl**_ (0.269 g, 0.345 mmol) and silver acetylide 4-ethynyltoluene (0.085 g, 0.380 mmol) was refluxed in 1,2-dichloroethane (20 mL) for 24 h. The suspension was filtered through Celite. The filtrate was evaporated to dryness and treated with *n*-hexane (10 mL) to obtain *N,N* **-** *trans* **-2a** as a yellow solid (0.223 g, 75%). Elem. Anal. Calcd for C_44_H_32_IrN_3_S_2_ (859.10): C, 61.52; H, 3.75; N, 4.89; S, 7.46. Found: C, 61.05; H, 3.74; N, 4.60; S, 6.92. ESI (+): *m*/*z* (%) 744.137 [M–C≡CR]^+^ (100), 860.202 [M + H]^+^ (7). IR (cm^–1^): ν­(C≡N) 2121 (vs), ν­(C≡C) 2088 (s). ^1^H NMR (400 MHz, CDCl_3_): δ 10.35 (br, ^3^ *J*_H–H_ = 6.5, H^7/7′^), 8.60 (d, ^3^ *J*_H–H_ = 7.7, H^7́/7^), 7.94 (d, ^3^ *J*_H–H_ = 7.8, H^4/4′^), 7.89 (d, ^3^ *J*_H–H_ = 7.9, H^4′/4^), 7.69 (d, ^3^ *J*_H–H_ = 8.0, 2H, H^8, 8′^), 7.59 (t, ^3^ *J*_H–H_ = 7.5, H^6/6′^), 7.53–7.46 (m, 3H, H^5, 5′, 6′/6^), 7.05 (t, ^3^ *J*_H–H_ = 7.5, H^*p*^), 7.02 (d, ^3^ *J*_H–H_ = 8.0, 2H, H^2″^), 6.96 (d, ^3^ *J*_H–H_ = 8.0, 2H, H^*m*^), 6.92–6.87 (m, 4H, H^9, 9′, 3″^), 6.78 (dd, ^3^ *J*_H–H_ = 7.2, ^3^ *J*_H–H_ = 6.9, H^10, 10′^), 6.52 (d, ^3^ *J*_H–H_ = 7.3, H^11/11′^), 6.43 (d, ^3^ *J*_H–H_ = 7.5, H^11′/11^), 2.22 (s, 3H, Me _Tol_), 2.10 (s, 6H, Me _Xyl_). ^13^C­{^1^H} NMR (100.6 MHz, CDCl_3_): δ 182.0 (s, C^2, 2′^), 152.2 (s, C^7a/7a′^), 150.7 (s, C^7a′/7a^), 141.4 (s, C^13, 13′^), 139.9 (s, C^12, 12′^), 135.2 (s, C^*o*^), 133.8 (s, C^1″^), 131.5–131.4 (s, C^11, 11′^), 131.0 (s, C^10, 10′^), 130.8 (s, C^3a, 3a′^), 128.2 (s, C^*p*^), 127.8 (s, C^*m*^), 127.6 (s, C^2″^), 125.5 (s, C^5, 5′^), 125.4 (s, C^8, 8′^), 122.8 (s, C^4, 4′^), 121.8 (s, C^7, 7′^), 21.3 (s, Me _Tol_), 18.6 (s, Me _Xyl_).

#### Synthesis of *N*,*N*-*trans*-[Ir­(pbt)_2_(CNXyl)­(C≡C–C_6_H_2_(OMe)_3_)] **(2b)**

A mixture of **2**_**Cl**_ (0.205 g, 0.263 mmol) and AgC≡C–C_6_H_2_(OMe)_3_ (0.086 g, 0.289 mmol) was refluxed in 1,2-dichloroethane (20 mL) for 4 h. After filtration through Celite, the filtrate was evaporated to dryness and treated with toluene (20 mL) and filtered again. The soluble fraction was evaporated to small volume (2 mL) and treated with *n*-hexane (10 mL) to obtain *N,N* **-** *trans* **-2b** as a yellow solid (0.223 g, 75%). Elem. Anal. Calcd for C_47_H_40_IrN_3_O_3_S_2_ (951.19): C, 59.35; H, 4.24; N, 4.42; S, 6.74. Found: C, 59.20; H, 4.28; N, 3.96; S, 6.77. ESI (+): *m*/*z* (%) 744.144 [M–C≡CR]^+^ (100), 936.226 [M + H]^+^ (23). IR (cm^–1^): ν­(C≡N) 2126 (vs), ν­(C≡C) 2089 (s). ^1^H NMR (400 MHz, CDCl_3_): δ 10.30 (d, ^3^ *J*_H–H_ = 8.6, H^7/7′^), 8.61 (d, ^3^ *J*_H–H_ = 8.1, H^7′/7^), 7.94 (d, ^3^ *J*_H–H_ = 7.7, H^4/4′^), 7.90 (d, ^3^ *J*_H–H_ = 7.9, H^4′**/4**^), 7.70 (d, ^3^ *J*_H–H_ = 6.2, H^8/8′^), 7.68 (d, ^3^ *J*_H–H_ = 6.2, H^8′/8^), 7.59 (t, ^3^ *J*_H–H_ = 7.6, H^6/6′^), 7.54–7.46 (m, 3H, H^5, 5′, 6′/6^), 7.06 (t, ^3^ *J*_H–H_ = 7.6, H^*p*^), 6.96 (d, ^3^ *J*_H–H_ = 7.7, 2H, H^*m*^), 6.89 (t, ^3^ *J*_H–H_ = 7.3, 2H, H^9, 9′^), 6.80–6.73 (m, 2H, H^10, 10′^), 6.52 (d, ^3^ *J*_H–H_ = 7.5, H^11/11′^), 6.43 (d, ^3^ *J*_H–H_ = 7.5, H^11′/11^), 6.35 (s, 2H, H^2″^), 3.73 (s, 3H, Me _C≡C_–_C6H2(OMe)3_), 3.71 (s, 6H, Me _C≡C_–_C6H2(OMe)3_), 2.10 (s, 6H, Me _Xyl_). ^13^C­{^1^H} NMR (100.6 MHz, CDCl_3_): δ 182.1 (s, C^2/2′^), 179.7 (s, C^2′/2^), 164.5 (s, C^7a/7a′^), 162.1 (s, C^7a′/7a^), 153.3 (s, C^1″^), 152.4 (s, C^O^–^Me^), 150.9 (s, C^3′′^),141.1 (s, C^12/12′^), 140.0 (s, C^12′/12^), 135.2 (s, C^13, 13′^), 133.9 (s, C^11/11′^), 131.6 (s, C^11′/11^), 131.5 (s, C^3a/3a′^), 131.4 (s, C^3a′/3a^), 130.9 (m, C^10, 10′^), 128.3 (s, C^*p*^_CNXyl_), 127.9 (s, C^*m*^_CNxyl_), 127.6 (s, C^6/6′^), 126.9–125.5 (m, C^5, 5′, 6′/6^), 125.4 (m, C^8, 8′^), 125.1 (s, C^7/7′^), 122.9 (s, C^4/4′^), 122.2 (s, C^4′/4^), 122.0–121.9 (C^7′/7, 9, 9′^), 110.0 (C^β^, C^α^)­109.0 (s, C^2″^_C≡C_–_C6H2(OMe)3_), 60.6 (s, Me _C≡C_–_C6H2(OMe)3_), 56.0 (s, Me _C≡C_–_C6H2(OMe)3_), 18.3 (s, Me _Xyl_).

#### Synthesis of *N*,*N* *-trans*-[Ir­(pbt)_2_(CNXyl)­(C≡C-pyrene)] **(2c)**

This complex was synthesized as a yellow solid (0.081 g, 73%) in a similar way to *N,N* **-** *trans* **-2a**, using **2**_**Cl**_ (0.090 g, 0.115 mmol) and AgC≡C-pyrene (0.0386 g, 0.152 mmol). Elem. Anal. Calcd for C_53_H_34_IrN_3_S_2_ (969.21): C, 65.68; H, 3.54; N, 4.34; S, 6.62. Found: C, 65.14; H, 3.44; N, 4.03; S, 6.22. ESI (+): *m*/*z* (%) 744.131 [M–C≡CR]^+^ (100). IR (cm^–1^): ν­(C≡N) 2130 (vs), ν­(C≡C) 2078 (s). ^1^H NMR (400 MHz, CDCl_3_): δ 10.45 (d, ^3^ *J*_H–H_ = 8.5, H^7/7′^), 8.72 (d, ^3^ *J*_H–H_ = 8.1, H^7′/7^), 8.11 (d, ^3^ *J*_H–H_ = 8.9, H^pyrene^), 8.00–7.97 (m, 4H, H^4,4′,8,8′^), 7.91–7.82 (m, 6H H^pyrene^), 7.80–7.74 (m, 2H, H^pyrene^), 7.57–7.44 (m, 4H, H^5, 5′, 6, 6′^), 7.05 (t, ^3^ *J*_H–H_ = 7.6, H^*p*^), 7.00–6.93 (m, 4H, H^9, 9′, *m*^), 6.86 (t, ^3^ *J*_H–H_ = 8.4, H^10/10′^), 6.83 (t, ^3^ *J*_H–H_ = 8.3, H^10′/10^), 6.67 (d, ^3^ *J*_H–H_ = 8.0, H^11/11′^), 6.53 (d, ^3^ *J*_H–H_ = 7.5, H^11′/11^), 2.11 (s, 6H, Me _CNXyl_). ^13^C­{^1^H} NMR (100.6 MHz, CDCl_3_): δ 181.1 (s, C^2/2′^), 180.2 (s, C^2′/2^), 164.2 (s, C^12/12′^), 162.0 (s, C^12′/12^), 152.2 (s, C^7a/7a′^), 151.0 (s, C^7a′/7a^), 141.5 (s, C^13/13′^), 140.1 (s, C^13′/13^), 135.4 (C^*o*^_Xyl_), 134.3 (s, C^11/11′^), 131.7–130.9 (C^11′/11, 3a, 3a′,10, 10′^), 19.3 (C^pyrene^), 128.4 (s, C^*p*^_Xyl_), 127.9 (s, C^*m*^_Xyl_), 127.8–127.0 (C^pyrene^), 126.2–125.5 (m, C^5, 5′, 6, 6′, pyrene^), 124.2–121.8 (s, C^4, 4′, 8, 8′, 7, 7′, 9, 9′^), 19.1 (s, Me _Xyl_).

#### Synthesis of *N*,*N*-trans-[Ir­(pq)_2_(CNXyl)­(C≡C-tol)] **(3a)**

This complex was obtained as an orange solid (0.040 g, 36%) following the procedure described for *N,N* **-** *trans* **-2b** using **3**_**Cl**_ (0.103 g, 0.134 mmol) and silver acetylide 4-ethynyltoluene (0.033 g, 0.147 mmol) in 1,2-dichloroethane (20 mL) at room temperature for 3 h. Elem. Anal. Calcd for C_48_H_36_IrN_3_ (847.05): C, 68.06; H, 4.28; N, 4.96. Found: C, 67.82; H, 4.69; N, 4.59. ESI (+): *m*/*z* (%) 732.222 [M–C≡CR]^+^ (100), 848.289 [M + H]^+^ (53). IR (cm^–1^): ν­(C≡N) 2122 (vs), ν­(C≡C) 2101 (s). ^1^H NMR (400 MHz, CDCl_3_): δ 11.22 (d, ^3^ *J*_H–H_ = 8.7, H^8/8′^), 9.47 (d, ^3^ *J*_H–H_ = 8.7, H^8′/8^), 8.25 (d, ^3^ *J*_H–H_ = 8.7, H^4/4′^), 8.20 (d, ^3^ *J*_H–H_ = 8.7, H^4′/4^), 8.04 (d, ^3^ *J*_H–H_ = 7.2, H^3/3′^), 8.02 (d, ^3^ *J*_H–H_ = 7.4, H^3′/3^), 7.83 (m, 4H, H^5, 5′,7, 7′^), 7.70–7.53 (m, 4H, H^6, 6′, 9, 9′^), 6.92 (m, 4H, H^10, 10′, 2″^), 6.85 (m, 5H, H^3″, *m, p*^), 6.70 (m, 2H, H^11, 11′^), 6.27 (d, ^3^ *J*_H–H_ = 7.6, H^12/12′^), 6.22 (d, ^3^ *J*_H–H_ = 7.6, H^12′/12^), 2.19 (s, 3H, Me _Tol_), 1.58 (s, 6H, Me _Xyl_). ^13^C­{^1^H} NMR (100.6 MHz, CDCl_3_): δ 172.5 (s, C^2/2′^), 171.2 (s, C^2′/2^), 167.0 (s, C^13/13′^), 164.4 (s, C^13′/13^), 150.1 (s, C^8a/8a′^), 148.8 (s, C^8a′/8a^), 147.3 (s, C^14/14′^), 145.9 (s, C^14′/14^), 138.6 (s, C^4/4′^), 137.7 (s, C^4′/4^), 134.9 (s, C^*o*^), 134.0 (s, C^8/8′^), 133.2 (s, C^12/12′^), 133.1 (s, C^4″^), 131.7 (s, C^12′/12^), 131.2 (s, C^2″^), 131.0 (s, C^8′/8^), 130.1 (s, C^11, 11′^), 130.0 (s, C^*p*^), 129.0 (s, C^4a, 4a′^), 128.2–127.7 8 (s, C^5, 5′, 7, 7′, 10, 10′^), 128.0 (s, C^3″^), 127.5 (s, C^*m*^), 127.3 (s, C^1″^), 127.0 (s, C^ipso^), 126.8 (s, C^6/6′^), 126.0 (s, C^6′/6^), 125.9 (s, C^9/9′^), 125.6 (s, C^9′/9^), 121.5 (s, C^3″^), 118.0 (s, C^3/3′^), 117.5 (s, C^3′/3^), 108.4 (s, C^β^), 102.3 (s, C^α^), 21.1 (s, C^Me^_Tol_), 18.2 (s, C^Me^_Xyl_).

#### Synthesis of *N,N*-*trans*-[Ir­(pq)_2_(CNXyl)­(C≡C–C_6_H_2_(OMe)_3_)] **(3b)**

This complex was obtained as an orange solid (0.099 g, 86%) following a procedure similar to that for *N,N* **-** *trans* **-1a**, using **3**_**Cl**_ (0.115 g, 0.150 mmol) and AgC≡C–C_6_H_2_(OMe)_3_ (0.058 g, 0.194 mmol) in 1,2-dichloroethane (20 mL) at room temperature for 6 h. Elem. Anal. Calcd for C_50_H_40_IrN_3_O_3_ (923.10): C, 65.06; H, 4.37; N, 4.55. Found: C, 65.01; H, 4.83; N, 4.19. ESI (+): *m*/*z* (%) 732.200 [M–C≡CR]^+^ (100), 924.280 [M + H]^+^ (27). IR (cm^–1^): ν­(C≡N) 2114 (vs), ν­(C≡C) 2086 (s). ^1^H NMR (400 MHz, CDCl_3_): δ 11.15 (d, ^3^ *J*_H–H_ = 8.9, H^8/8′^), 9.54 (d, ^3^ *J*_H–H_ = 8.9, H^8′/8^), 8.25 (d, ^3^ *J*_H–H_ = 8.6, H^4/4′^), 8.16 (d, ^3^ *J*_H–H_ = 8.5, H^4′/4^), 8.01 (d, ^3^ *J*_H–H_ = 8.6, H^3/3′^), 7.96 (d, ^3^ *J*_H–H_ = 8.6, H^3′/3^), 7.88–7.80 (m, 3H, H^5/5′, 7, 7′^), 7.66 (d, ^3^ *J*_H–H_ = 7.7, H^5′/5^), 7.61 (d, ^3^ *J*_H–H_ = 7.4, 2H, H^9, 9′^), 7.52 (t, ^3^ *J*_H–H_ = 7.5, H^6/6′^), 7.00 (t, ^3^ *J*_H–H_ = 7.5, H^*p*^), 7.92–6.86 (m, 4H, H^6′/6, 10, 10′, *m*^), 6.72 (t, ^3^ *J*_H–H_ = 7.6, 2H, H^11, 11′^), 6.28 (s, H^2″^) 6.20 (d, ^3^ *J*_H–H_ = 8.0, 2H, H^12, 12′^), 3.71 (s, 3H, Me _Tol_), 3.65 (s, 6H, Me _Tol_), 1.85 (s, 6H, Me _xyl_). ^13^C­{^1^H} NMR (100.6 MHz, CDCl_3_): δ 172.5 (s, C^2/2′^), 171.2 (s, C^2′/2^), 166.9 (s, C^13/13′^), 164.7 (s, C^13′/13^), 152.4 (s, C^3″^), 150.2 (s, C^8a/8a′^), 148.8 (s, C^8a′/8a^), 147.5 (s, C^14/14′^), 146.0 (s, C^14′/14^), 144.3 (s, C^ipso^), 138.8 (s, C^4/4′^), 137.9 (s, C^4′/4^), 135.7 (s, C^4″^), 134.9 (s, C^*o*^), 133.9 (s, C^8/8′^), 133.1 (s, C^12/12′^), 131.9 (s, C^12′/12^), 131.1 (s, C^8′/8^), 130.9 (s, C^7/7′^), 130.2 (s, C^11, 11′^), 128.4–128.0 (s, C^5, 5, 7′/7^), 127.9 (s, C^*p*^), 127.6 (s, C^*m*^), 126.9 (s, C^6/6′^), 126.1–125.5 (C^6′/6, 9, 9′, 1″^), 121.8 (s, C^10/10′^), 121.6 (s, C^10′/10^), 118.2 (s, C^3/3′^), 117.8 (s, C^3′/3^), 108.7 (s, C^α^), 108.6 (s, C^2″^), 103.5 (s, C^β^), 61.0 (s, Me _C≡C_–_C6H2(OMe)3_), 55.9 (s, Me _C≡C(–C6H2(OMe)3_), 18.4 (s, Me _Xyl_).

#### Synthesis of *N*,*N*-*trans*-[Ir­(pq)_2_(CNXyl)­(C≡C-pyrene)] **(3c)**

This complex was obtained as an orange solid (0.076 g, 60%) following a procedure similar to that for *N,N* **-** *trans* **-1a**, using **3**_**Cl**_ (0.211 g, 0.275 mmol) and 1-pyrene silver acetylide (0.092 g, 0.275 mmol) in 1,2-dichloroethane (20 mL) at room temperature for 3 h. Elem. Anal. Calcd for C_57_H_40_IrN_3_ (959.18): C, 71.38; H, 4.20; N, 4.38. Found: C, 71.74; H, 4.50; N, 4.52. ESI (+): *m*/*z* (%) 732.198 [M–C≡CR]^+^ (100), 958.278 [M + H]^+^ (14). IR (cm^–1^): ν­(C≡N) 2121 (vs), ν­(C≡C) 2074 (s). ^1^H NMR (400 MHz, CDCl_3_): δ 11.32 (d, ^3^ *J*_H–H_ = 8.9, H^8/8′^), 9.54 (d, ^3^ *J*_H–H_ = 8.9, H^8′/8^), 8.29 (d, ^3^ *J*_H–H_ = 8.3, H^4/4′^), 8.27 (d, ^3^ *J*_H–H_ = 8.3, H^4′/4^), 8.12–8.08 (m, H^3, 3′^), 7.98 (d, 2H, ^3^ *J*_H–H_ = 8.3, H^5, 5′^), 7.84 (m, H^7, 7′, pyrene^), 7.76 (d, 2H, ^3^ *J*_H–H_ = 8.1, H^9, 9′^), 7.69 (t, ^3^ *J*_H–H_ = 8.0, H^6/6′^), 7.61–7.55 (m, H^6′/6, *o*, pyrene^), 6.92 (m, H^10,10′,*m*^), 6.84 (m, H^pyrene^), 6.78 (dd, 2H, ^3^ *J*_H–H_ = 7.3, ^3^ *J*_H–H_ = 6.7, H^11, 11′^), 6.45 (d, ^3^ *J*_H–H_ = 7.5, H^12/12′^), 6.32 (d, ^3^ *J*_H–H_ = 7.5, H^12′/12^), 1.88 (s, 6H, Me _Xyl_). ^13^C­{^1^H} NMR (100.6 MHz, CDCl_3_): δ 172.7 (s, C^2/2′^), 171.6 (s, C^2′/2^), 166.4 (s, C^13/13′^), 164.6 (s, C^13′/13^), 150.2 (s, C^8a/8a′^), 149.3 (s, C^8a′/8a^), 147.6 (s, C^14/14′^), 146.3 (s, C^14′/14^),138.9 (s, C^4/4′^), 138.1 (s, C^4′/4^), 135.1 (s, C^*o*^), 134.1 (s, C^8/8′^), 133.5 (s, C^12/12′^), 131.9 (s, C^8′/8^), 131.7–131.6 (s, C^pyrene^), 131.3 (s, C^12′/12^), 130.6 (s, C^11/11′^), 130.4 (s, C^11′/11^), 130.3 (s, C^6/6′^), 129.6 (s, C^*p*^), 128.6 (s, C^pyrene^), 128.1 (s, C^pyrene^), 127.9–127.6 (s, C^7, 7′^), 127.7 (s, C^*m*^), 126.3 (s, C^6′/6^), 126.2 (s, C^9/9′^), 126.0 (s, C^9′/9^), 125.8–125.5 (s, C^pyrene^), 125.0–124.8 (s, C^pyrene^), 124.0–123.9 (s, C^5, 5′^), 121.8 (s, C^10, 10′^), 118.3 (s, C^3/3′^), 117.8 (s, C^3′/3^), 18.4 (s, Me _Xyl_).

### X-ray Structure Determinations

Yellow [*N,N-trans* **-1a** and *N,N-trans-* **1c**] and orange [*N,N-trans-* **2c** and *N,N-trans-* **3**_**Cl**_] crystals were obtained by slow diffusion of *n*-hexane into a solution of the corresponding compounds in CHCl_3_ at room temperature. Yellow [*N,N-trans-* **1**_**Cl**_, *N,N-cis-* **(** *OC* **-6–24)-1**_**Cl**_, *N,N-cis-* **(** *OC* **-6–44)-1**_**Cl**_, *N,N-cis-* **(** *OC* **-6–32)-1a**, and *N,N-cis-* **(** *OC* **-6–32)-1b**] crystals were obtained by slow diffusion of *n*-hexane into a solution of the corresponding compounds in CH_2_Cl_2_ at room temperature. Orange crystals of complex *N,N-trans-* **2a** were obtained by slow diffusion of *n*-hexane in acetone at room temperature. X-ray intensity data were collected using molybdenum graphite monochromatic (Mo–K_α_) radiation with a Bruker APEX-II diffractometer at 100 K using APEX-II software. Structures were solved by Intrinsic Phasing using SHELXT^27^ with the WinGX graphical user interface.^28^ Multiscan absorption corrections were applied to all the data sets and refined by full-matrix least-squares on *F* ^2^ with SHELXL.^29^ Hydrogen atoms were positioned geometrically, with isotropic parameters *U*_iso_ = 1.2 *U*_eq_ (parent atom) for aromatic hydrogens. These complexes crystallized in a centrosymmetric group, so must be aquiral. Thus, both configurations (Λ and Δ) in a 50:50 ratio were found in the unit cell. *N,N* **-** *trans* **-1**_**Cl**_·CH_2_Cl_2_ crystallized with one molecule of solvent, identified as CH_2_Cl_2_, which could be modeled. For *N,N* **-** *trans* **-3**_**Cl**_, *N,N* **-** *trans* **-1c**, *N,N* **-cis-(** *OC* **-6–32)-1a**, and *N,N* **-cis-(** *OC* **-6–32)-1b**, disordered crystallization molecules of solvents were observed, but could not be properly modeled. Examination with PLATON^30^ and SQUEEZE^30,31^ revealed for *N,N* **-** *trans* **-3**_**Cl**_ the presence of two voids of 324 Å^3^ in the unit cell, each of them containing 123 e^–^, which are attributed to the presence of one molecule of CHCl_3_ in the unit cell (*N,N* **-** *trans* **-3**_**Cl**_ **·**CHCl_3_), in *N,N* **-** *trans* **-1c** the presence of two voids of 281 Å^3^ in the unit cell, each of them containing 68 e^–^, which are attributed to the presence of one molecule of CHCl_3_ in the unit cell (*N,N* **-** *trans* **-1c·**0.6CHCl_3_), in *N,N* **-cis-(** *OC* **-6–32)-1a** the presence of one void of 239 Å^3^ in the unit cell containing 73 e^–^, which fit well with the presence of one molecule of CH_2_Cl_2_ in the unit cell (*N,N* **-cis-(** *OC* **-6–32)-1a**·1.75CH_2_Cl_2_), and in *N,N* **-cis-(** *OC* **-6–32)-1b** the presence of two voids of 218 Å^3^ in the unit cell containing 39 e^–^, which fit well with the presence of one molecule of CH_2_Cl_2_ in the unit cell (*N,N* **-cis-(** *OC* **-6–32)-1b**·0.9CH_2_Cl_2_).

### Theoretical Calculations

Calculations were carried out with the Gaussian 16 package^32^ for *N,N-trans-* **1**_**Cl**_, *N,N-cis-* **(** *OC* **-6–24)-1**_**Cl**_, *N,N-cis-* **(** *OC* **-6–44)-1**_**Cl**_, *C,C-trans-* **1**_**Cl**_, *N,N-trans-* **1a**, *N,N-cis-* **(** *OC* **-6–32)-1a**, and *N,N-trans-* **1c**, **2a**, **2b**, **2c, 3a,** and **3c** using Becké’s three-parameter functional combined with the Lee–Yang–Parŕs correlation functional (B3LYP).^33^ Optimizations on the singlet state (S_0_) were performed using as a starting point the molecular geometry obtained through X-ray diffraction analysis for complexes *N,N-cis-* **(** *OC* **-6–24)-1**_**Cl**_, *N,N-cis-* **(** *OC* **-6–44)-1**_**Cl**_, *N,N-trans-* **1a**, and *N,N-trans-* **1c**, **2a**, and **2c** and simulated structures for *N,N-trans* **-1**_**Cl**_, *C,C-trans-* **1**_**Cl**_, and *N,N-cis-* **(** *OC* **-6–32)-1a**, **2b, 3a**, and **3c**. No negative frequency was found in the vibrational frequency analysis of the final equilibrium geometries. The basis set used was the LanL2DZ effective core potential for Ir and 6–31G­(d,p) for the ligand atoms.^34^ DFT and TD-DFT calculations were carried out using the polarized continuum model approach^35^ (PCM) implemented in Gaussian 16 software, in the presence of CH_2_Cl_2_. The predicted emission wavelengths were obtained by energy difference between the triplet state at its optimized geometry and the singlet state at the triplet geometry. The results were visualized with GaussView 6. Overlap populations between molecular fragments were calculated using GaussSum 3.0 software.^36^

## Supplementary Material



## Notes

Characterization of complexes (NMR spectra and crystal data), photophysical and electrochemical properties, and computational details (PDF)
