# Supplementary material for: Ligand-Tuned Photophysical Properties and Geometrical Isomerism of Bis-Cyclometalated [Ir(C^N)2(CNXyl)X] (X = Cl, C≡CR) Complexes
Source: Inorg Chem. 2026 Jul 29;65(31):17926–45. doi: 10.1021/acs.inorgchem.6c01800 (PMC13463687; doi:10.1021/acs.inorgchem.6c01800)
Supplement: Supplementary file 1 [file ic6c01800_si_001.pdf]

Supporting Information

Ligand-Tuned Photophysical Properties and Geometrical Isomerism of Bis-Cyclometalated [Ir(C^N)$_2$(CNXyl)X] (X = Cl, C≡CR) Complexes

Andrea Corral-Zorzano, Mónica Martínez-Junquera, Elena Lalinde* and M. Teresa Moreno*

Departamento de Química, Instituto de Investigación en Química (IQUR), Complejo Científico Tecnológico, Universidad de La Rioja, Madre de Dios 53, 26006 Logroño, Spain. E-mail: teresa.moreno@unirioja.es

Contents: Page

1.- Computational data relative to stability of the different isomers ……………….. S2
2.- NMR Spectra……………………………………………………………………….. S3
3.- X-Ray Diffraction Studies……………………………………………………….. S29
4.- Photophysical Properties and Theoretical Calculations…………………………. S40
5.- Cyclic Voltammograms……………………………………………………………. S73

1. Computational data relative to stability of the different isomers

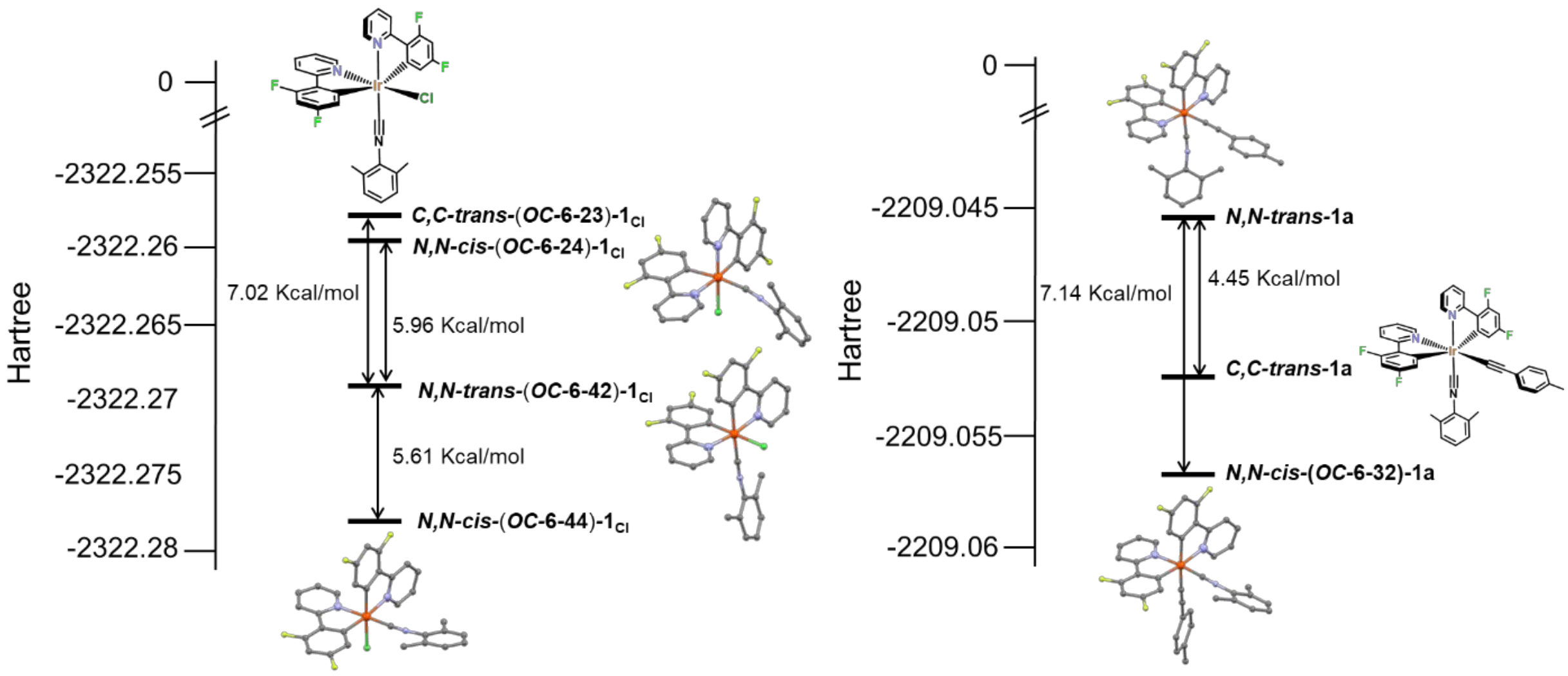

Figure S1. Optimized geometries by DFT calculations for compounds ***N,N*-*trans*-1**$_{\mathbf{Cl}}$, ***N,N*-*cis*-(*OC*-6-24)-1**$_{\mathbf{Cl}}$, ***N,N*-*cis*-(*OC*-6-44)-1**$_{\mathbf{Cl}}$, ***C,C*-*trans*-1**$_{\mathbf{Cl}}$ (left) and ***N,N*-*trans*-1a**, ***N,N*-*cis*-(*OC*-6-32)-1a** and ***C,C*-*trans*-1a** (right) with the differences in the ground state energies between the *cis* and the *trans* isomers.

2. NMR Spectra

a)

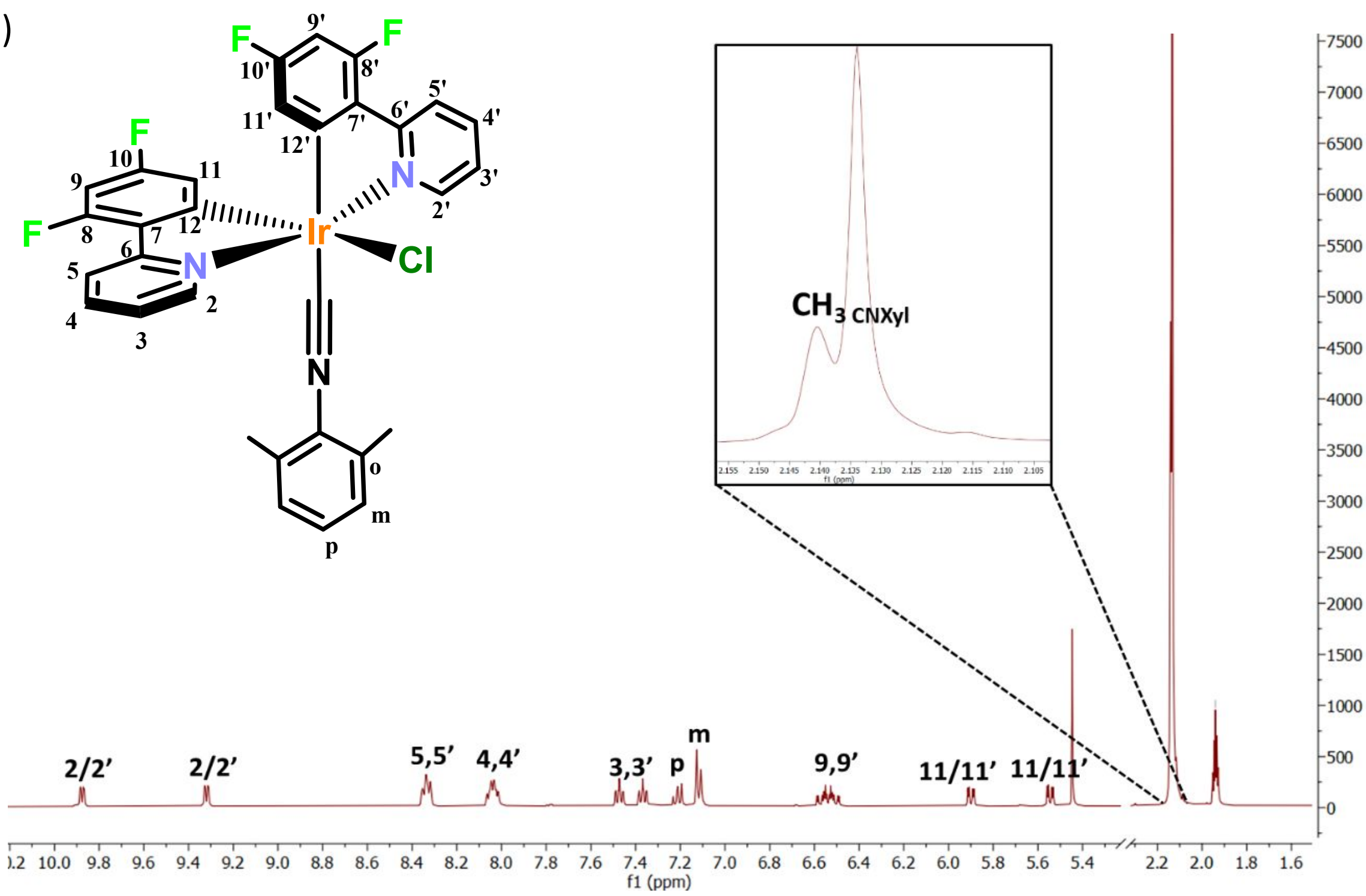

b)

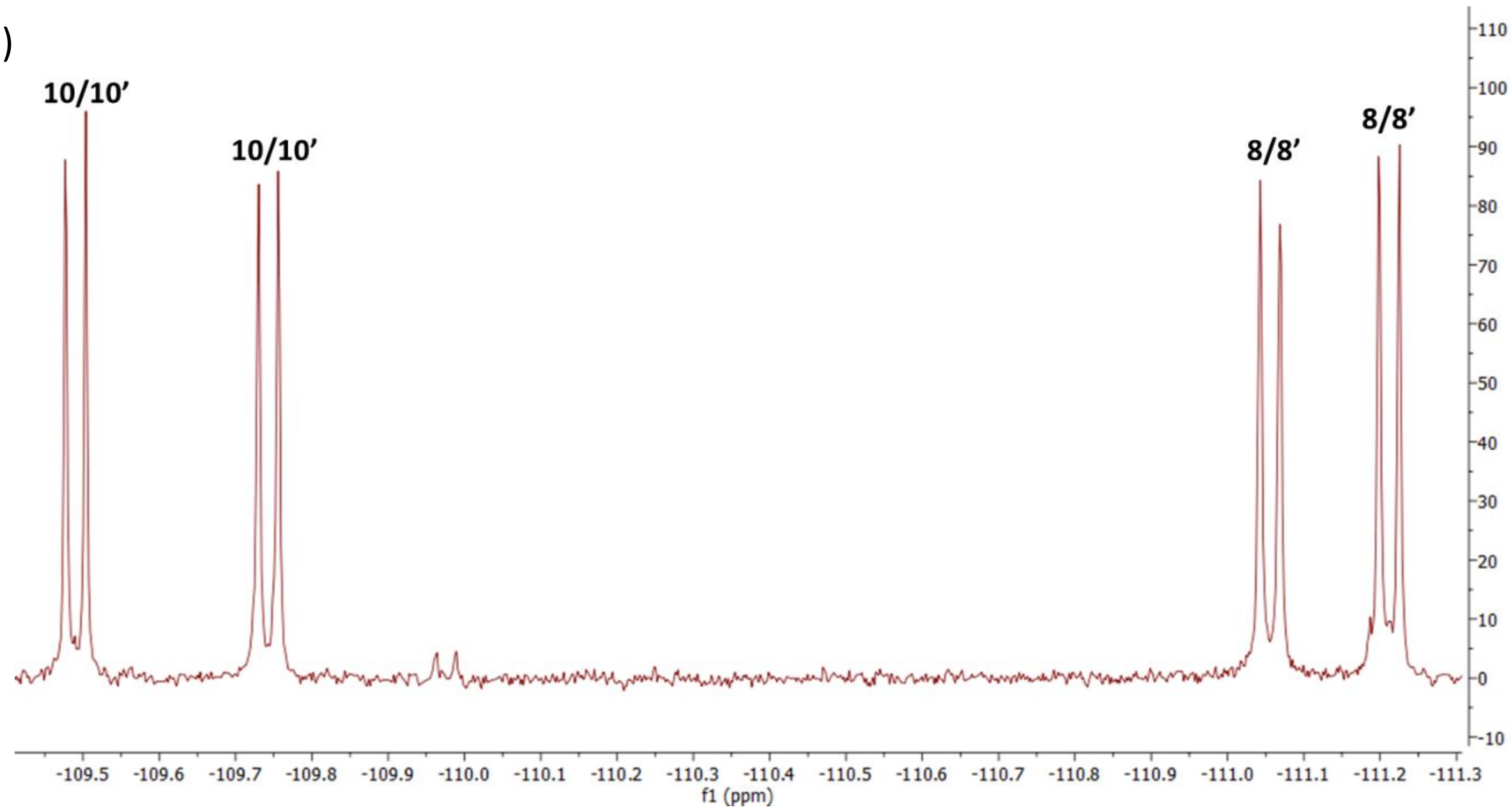

Figure S2. NMR spectra of ***N,N-trans*-1$_{Cl}$** in CD_3CN at 298 K a) ^1H and b) $^{19}F\{^1H\}$

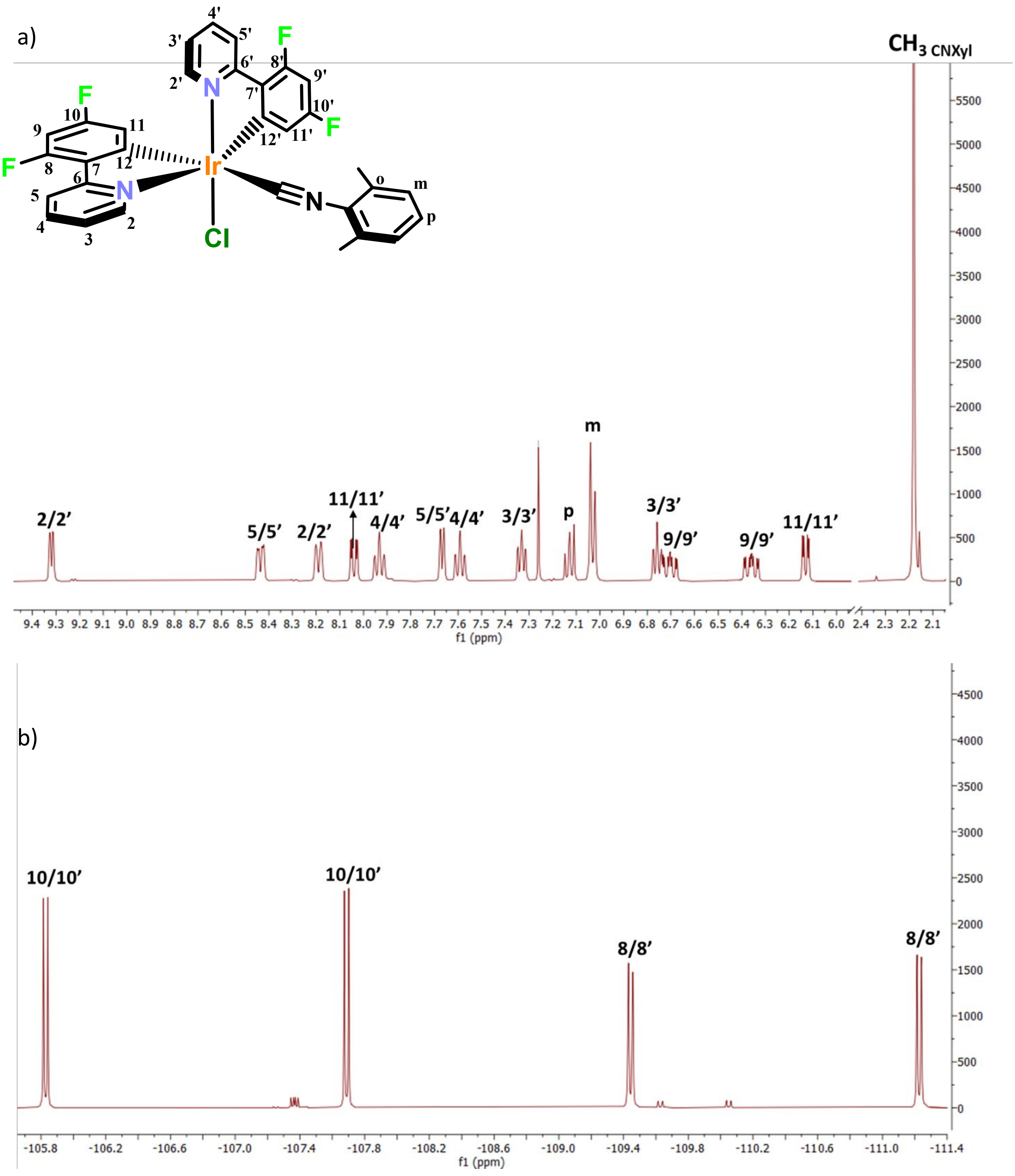

Figure S3. NMR spectra of ***N,N-cis*-(*OC*-6-24)-1**$_{\mathbf{Cl}}$ in $CDCl_3$ at 298 K a) ^{1}H and b) $^{19}F\{^{1}H\}$

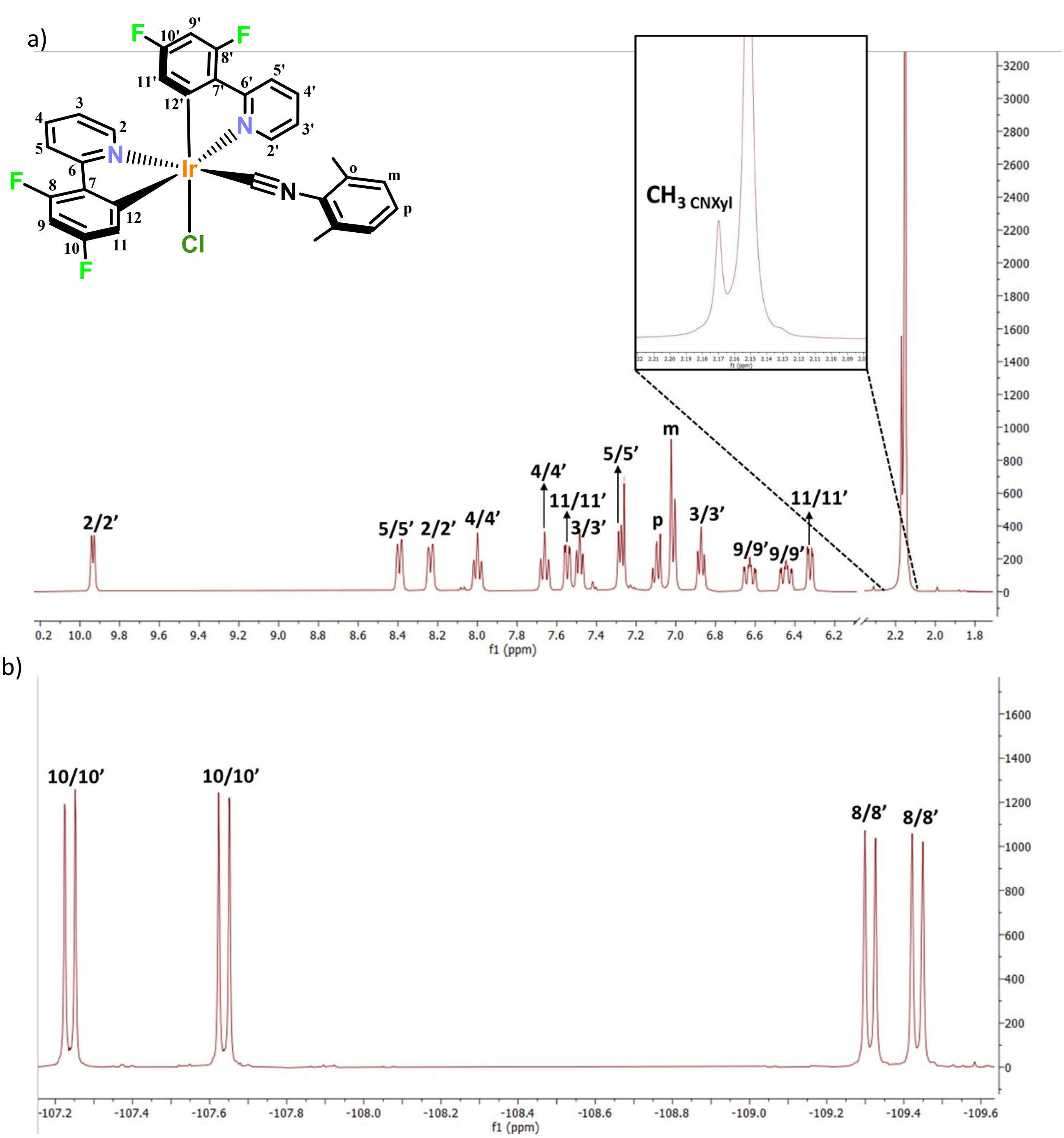

Figure S4. NMR spectra of ***N,N-cis*-(*OC*-6-44)-1$_{Cl}$** in $CDCl_3$ at 298 K a) ^1H and b) $^{19}F\{^1H\}$

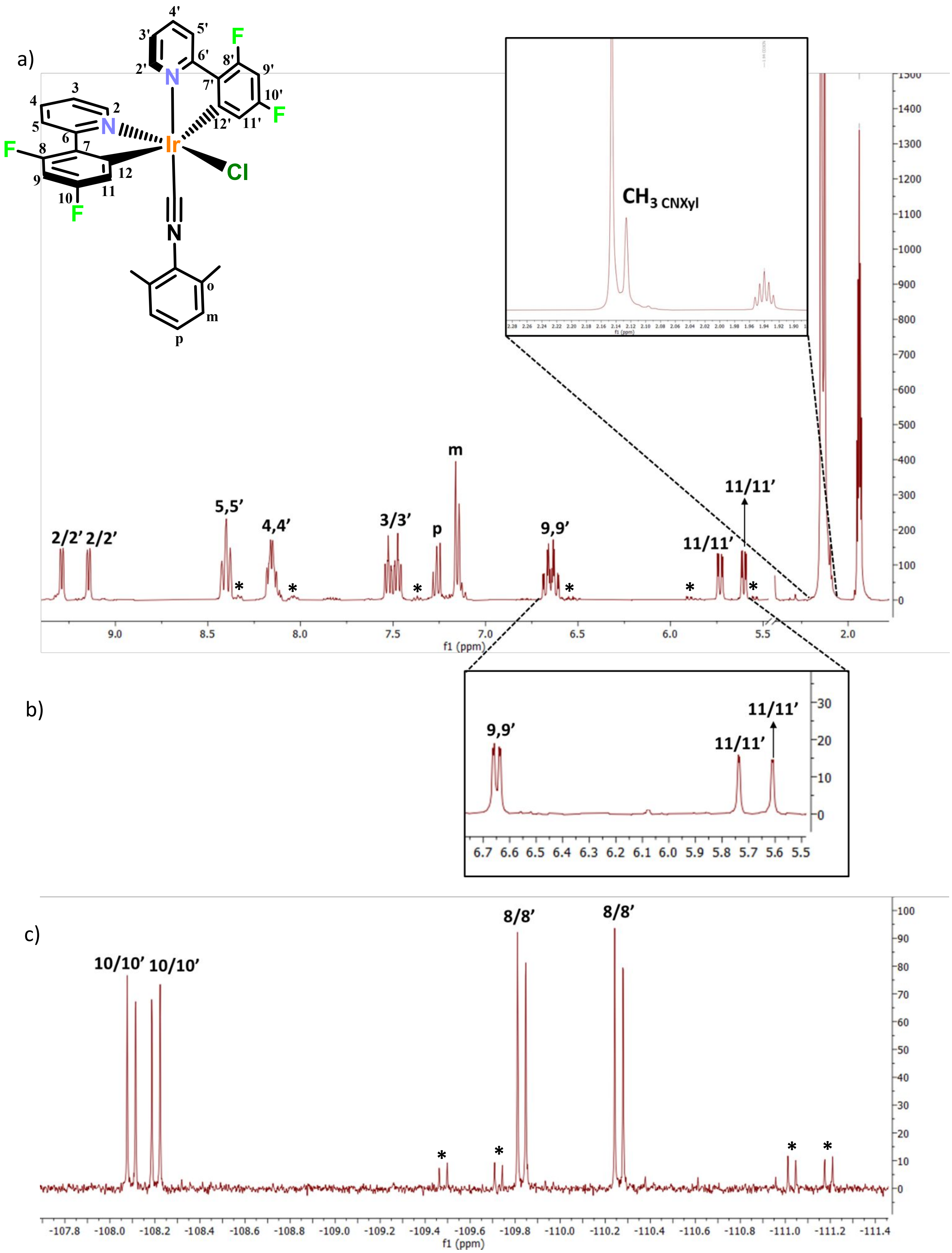

Figure S5. NMR spectra of ***C,C-trans*-1**$_{Cl}$ in CD_3CN at 298 K a) ^1H, b) ^1H {^{19}F} (region from 6.7-5.6 ppm corresponding to $H^{9,9'}$ and $H^{11,11'}$) and c) ^{19}F{^1H}. Signals marked with * correspond to ***N,N-trans*-1**$_{Cl}$

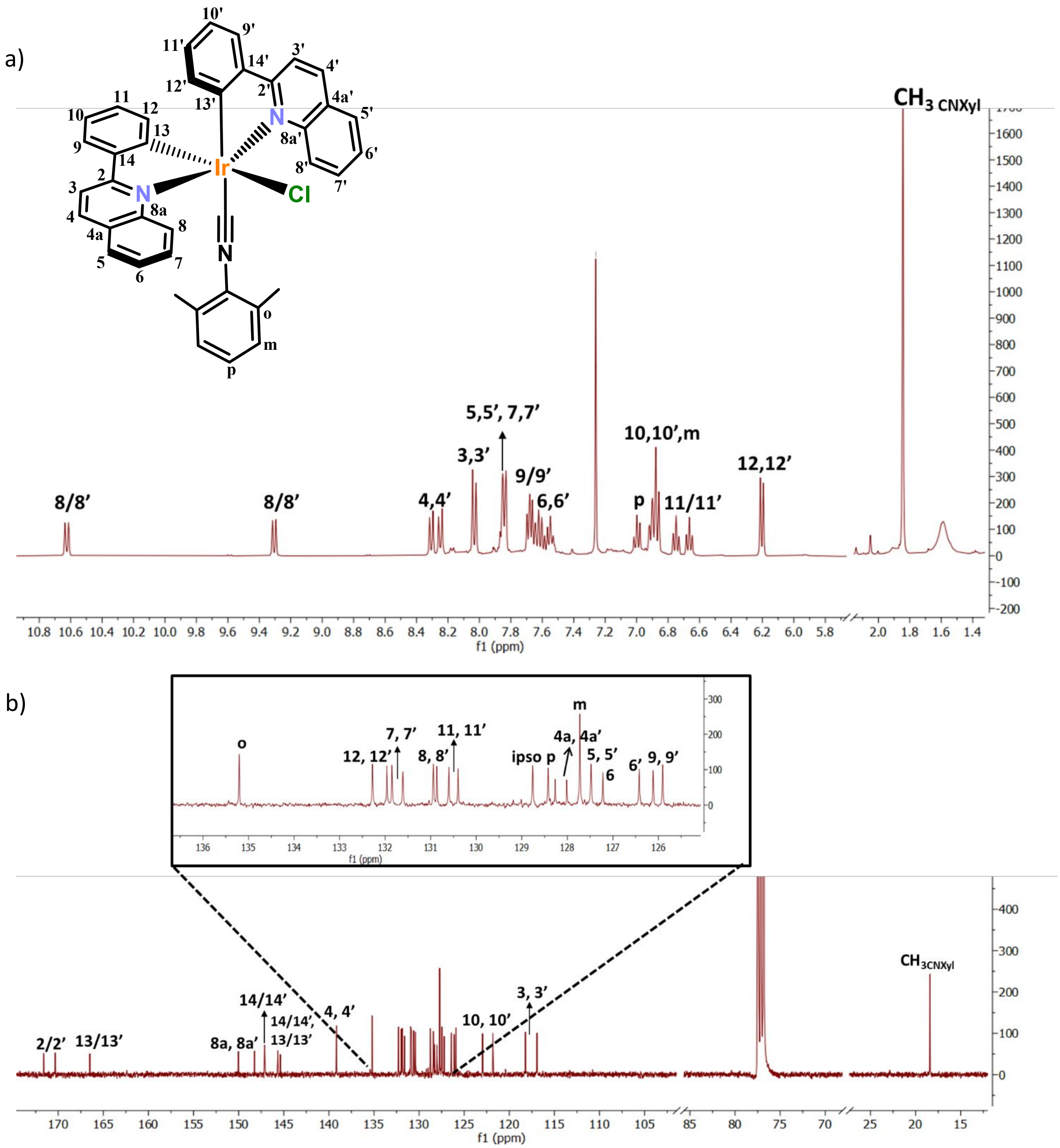

Figure S6. NMR spectra of ***N,N-trans*-3**$_{Cl}$ in $CDCl_3$ at 298 K a) ^1H and b) $^{13}C\{^1H\}$

a)

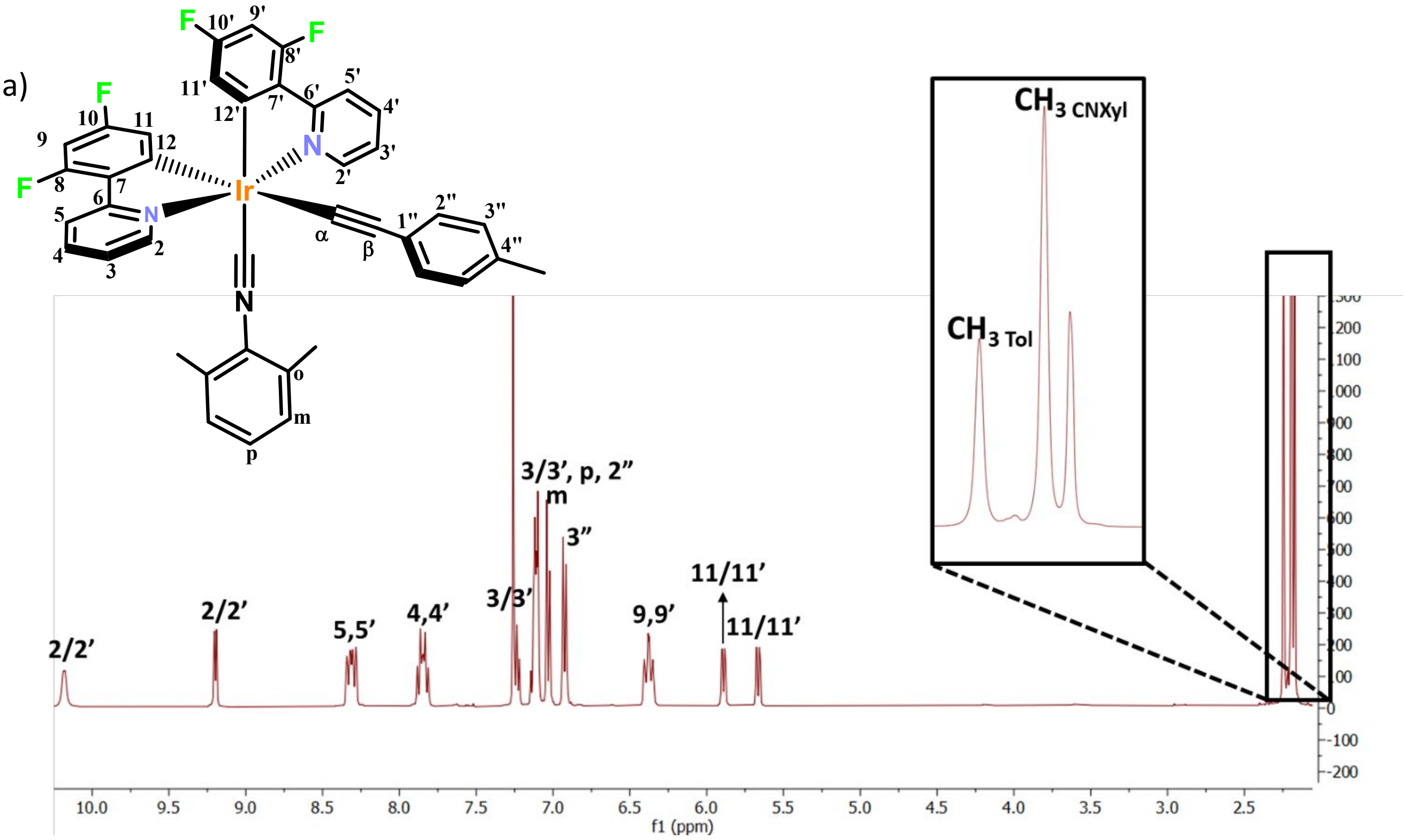

CH3 CNXyl
CH3 Tol
2/2'
2/2'
5,5'
4,4'
3/3'
3/3', p, 2"
m
3"
9,9'
11/11'
11/11'
f1 (ppm)

b)

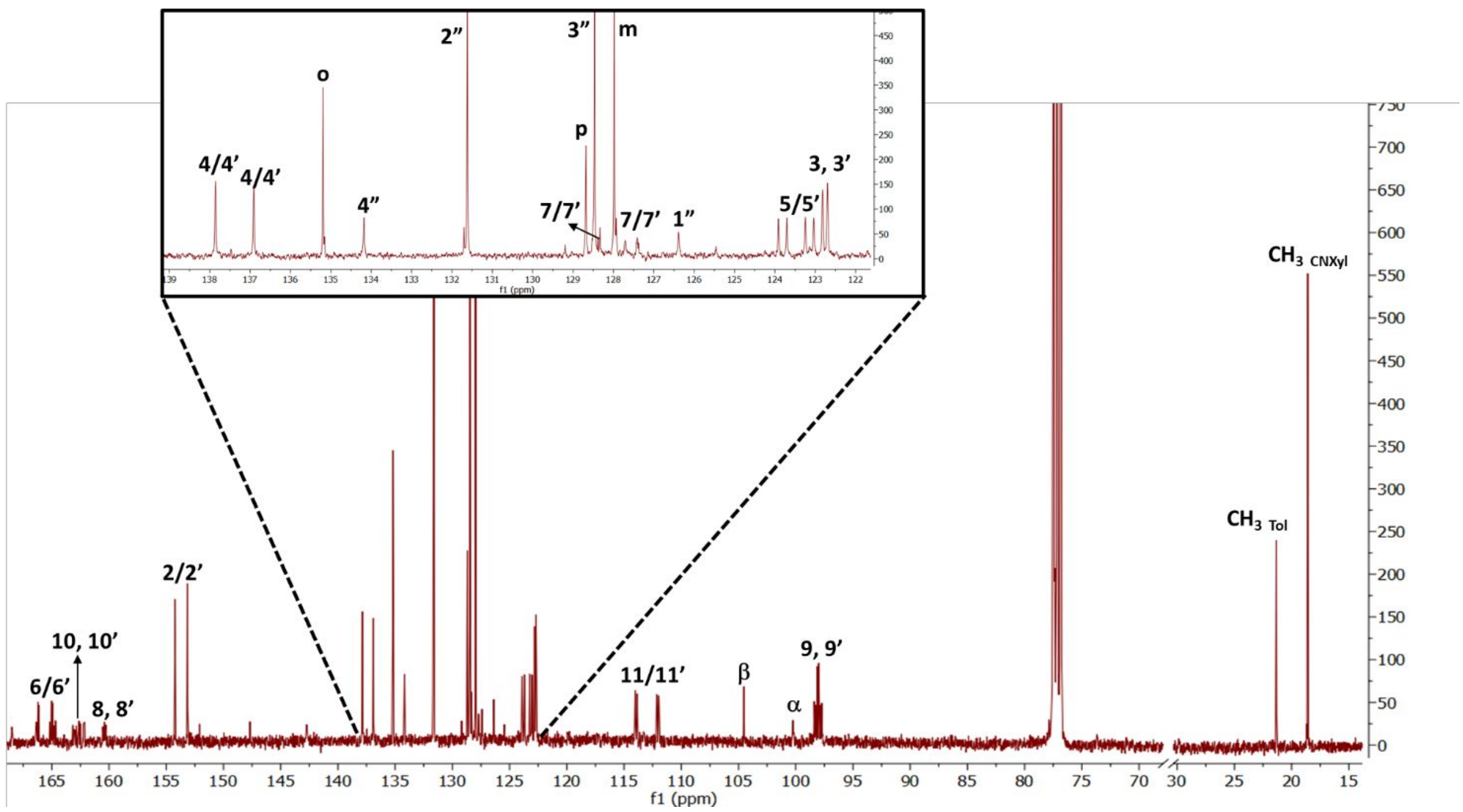

o
2"
3"
m
p
4/4'
4/4'
4"
7/7'
7/7'
1"
5/5'
3, 3'
f1 (ppm)
6/6'
10, 10'
8, 8'
2/2'
11/11'
β
α
9, 9'
CH3 Tol
CH3 CNXyl
f1 (ppm)

c)

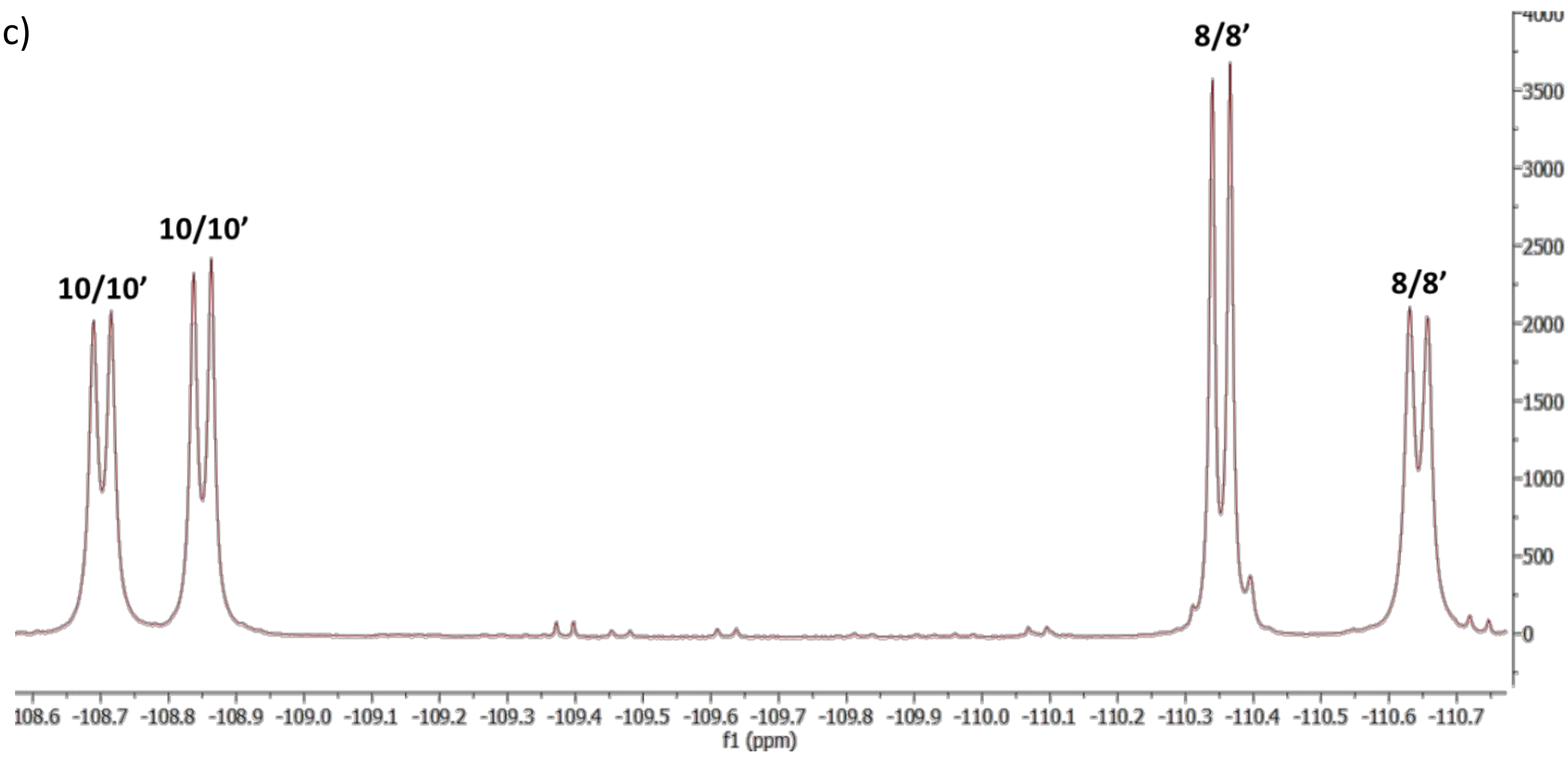

Figure S7. NMR spectra of ***N,N-trans*-1a** in $CDCl_3$ at 298 K a) ^{1}H; b) $^{13}C\{^{1}H\}$ and c) $^{19}F\{^{1}H\}$

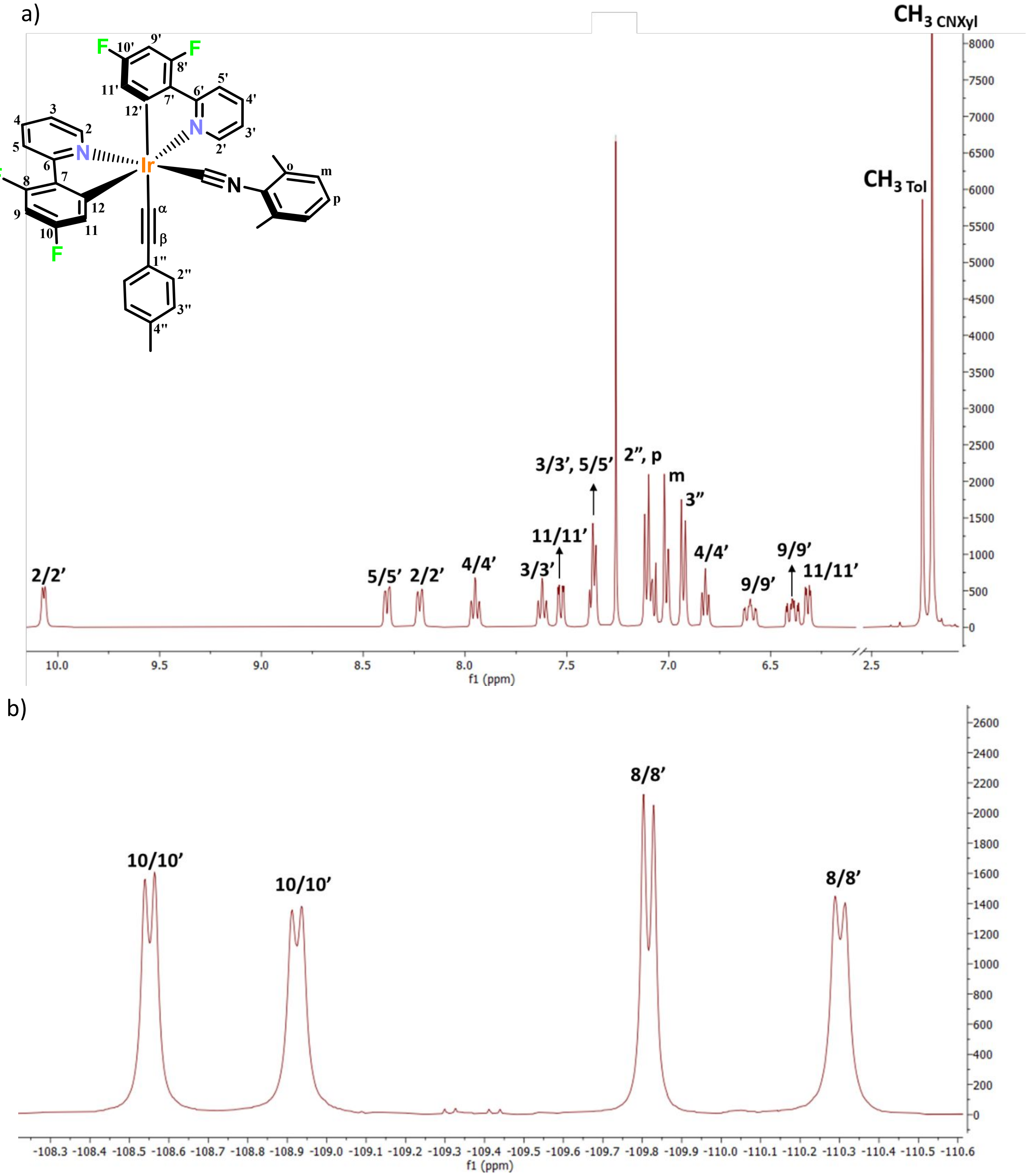

Figure S8. NMR spectra of ***N,N-cis*-(*OC*-6-32)-1a** in $CDCl_3$ at 298 K a) ^{1}H and b) $^{19}F\{^{1}H\}$

a)

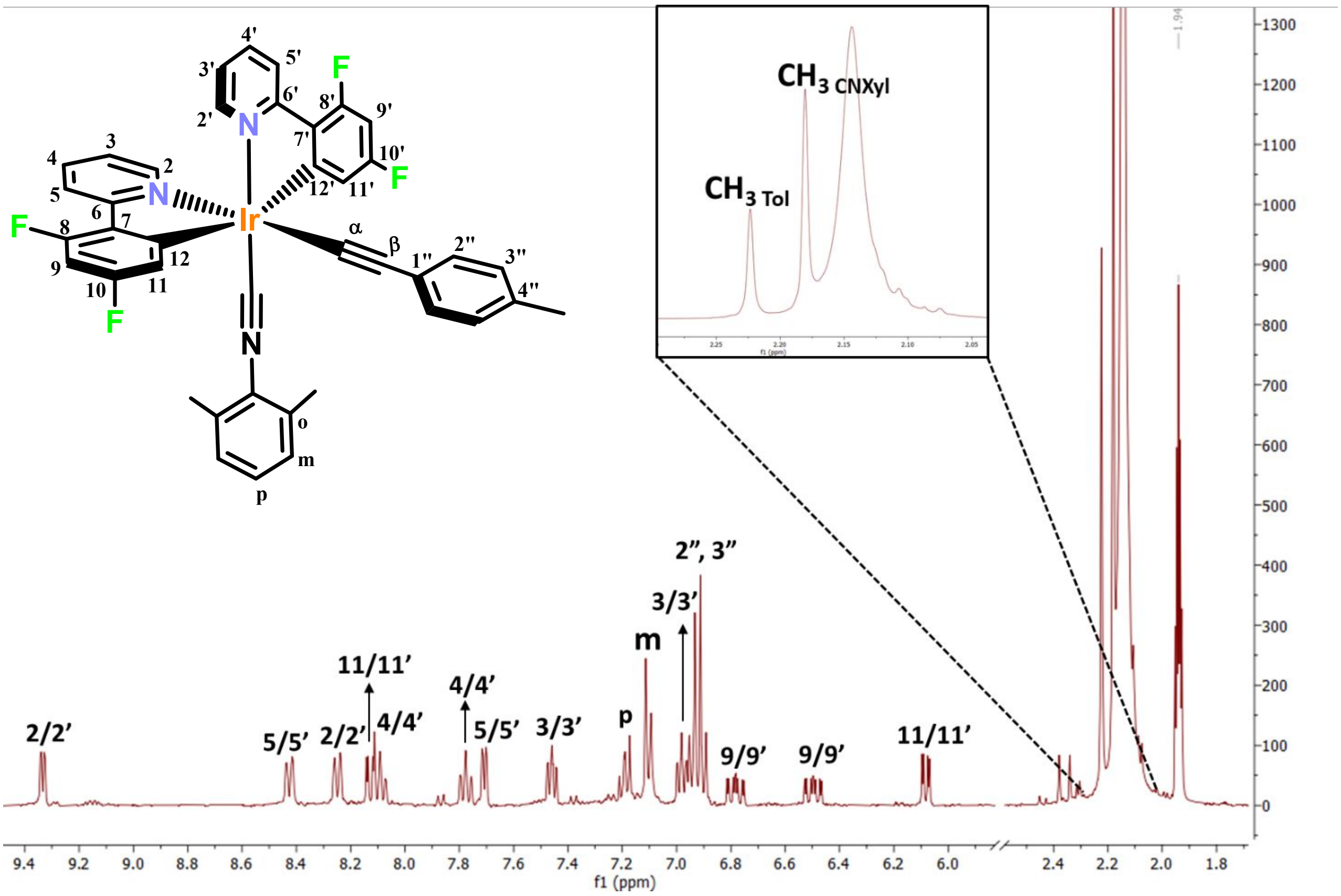

CH3 CNXyl
CH3 Tol
2/2'
5/5'
2/2'
11/11'
4/4'
4/4'
5/5'
3/3'
p
m
3/3'
2", 3"
9/9'
9/9'
11/11'
f1 (ppm)

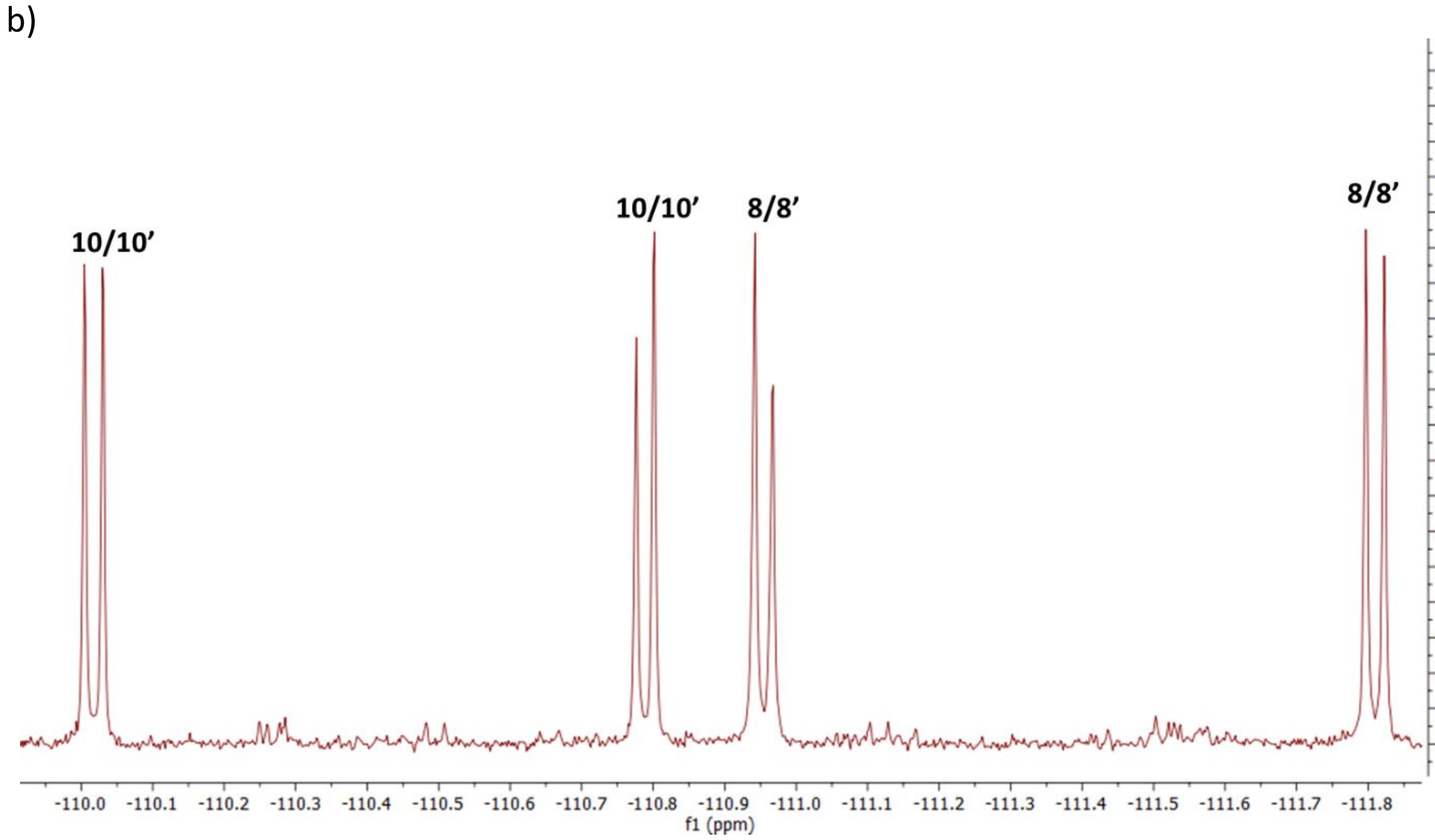

Figure S9. NMR spectra of ***C,C-trans*-1a** in CD_3CN at 298 K a) ^1H and b) $^{19}F\{^1H\}$

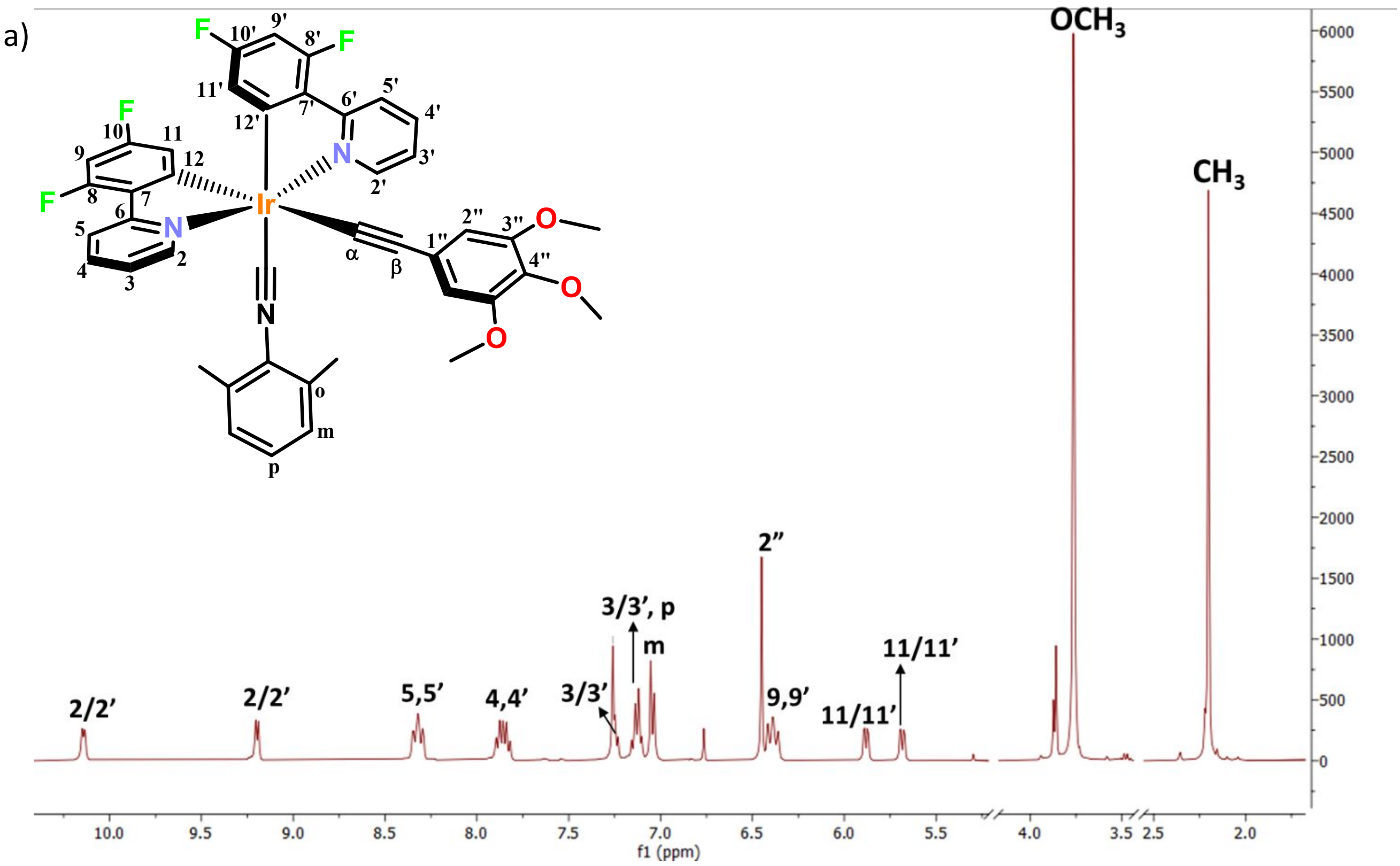

b)

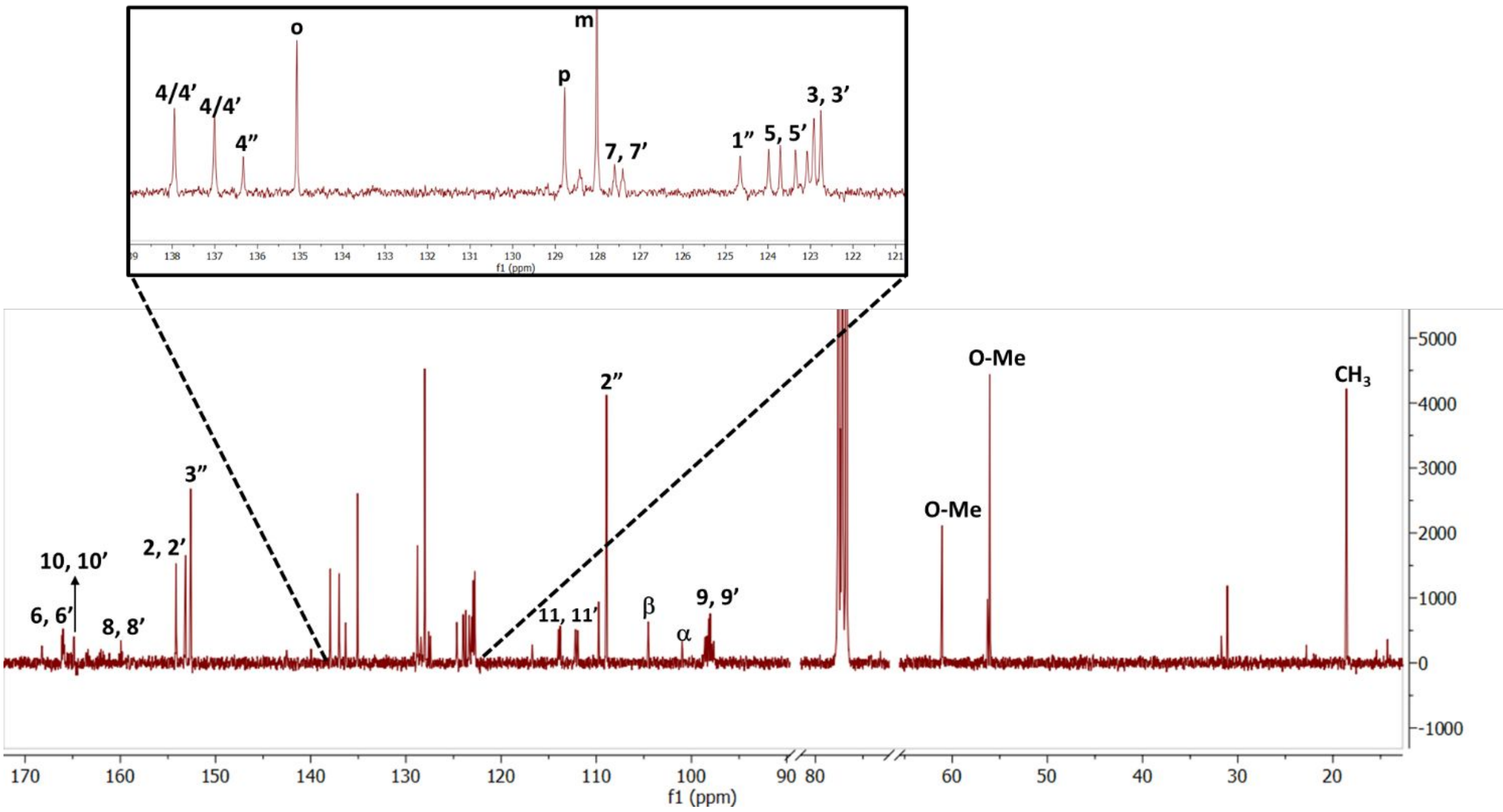

c)

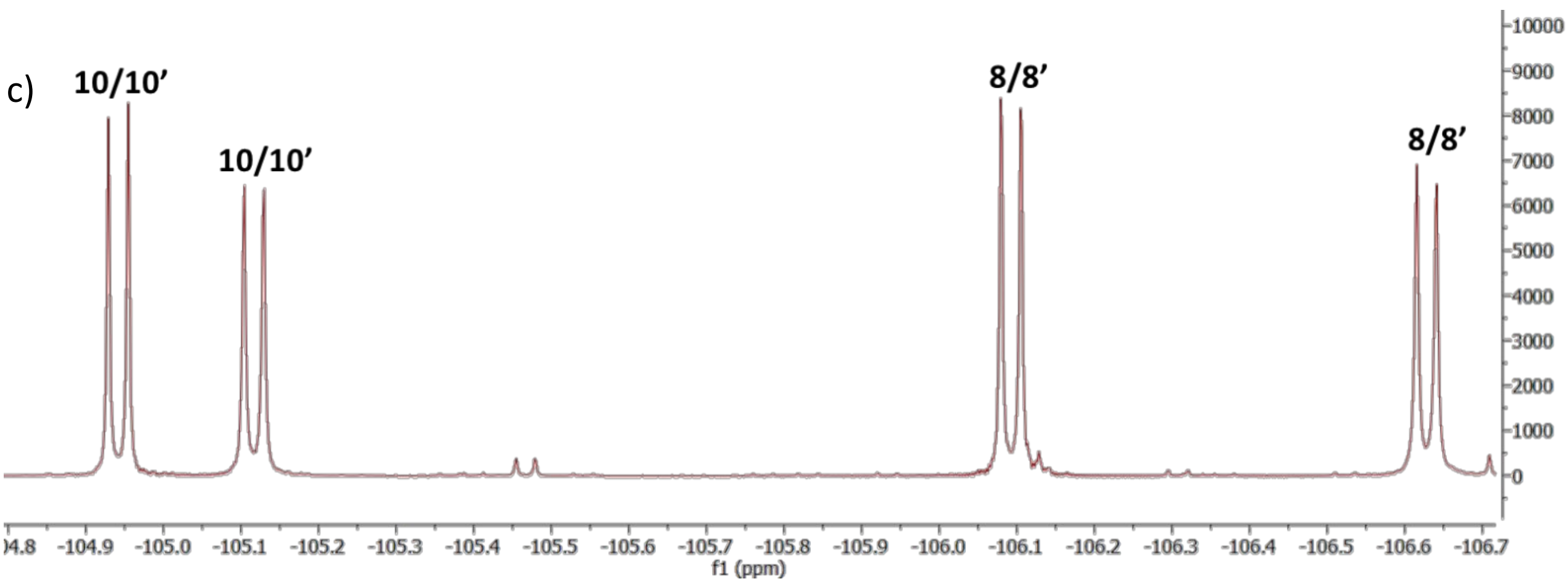

Figure S10. NMR spectra of ***N,N-trans*-1b** in $CDCl_3$ at 298 K a) ^1H; b) $^{13}C\{^1H\}$ and c) $^{19}F\{^1H\}$

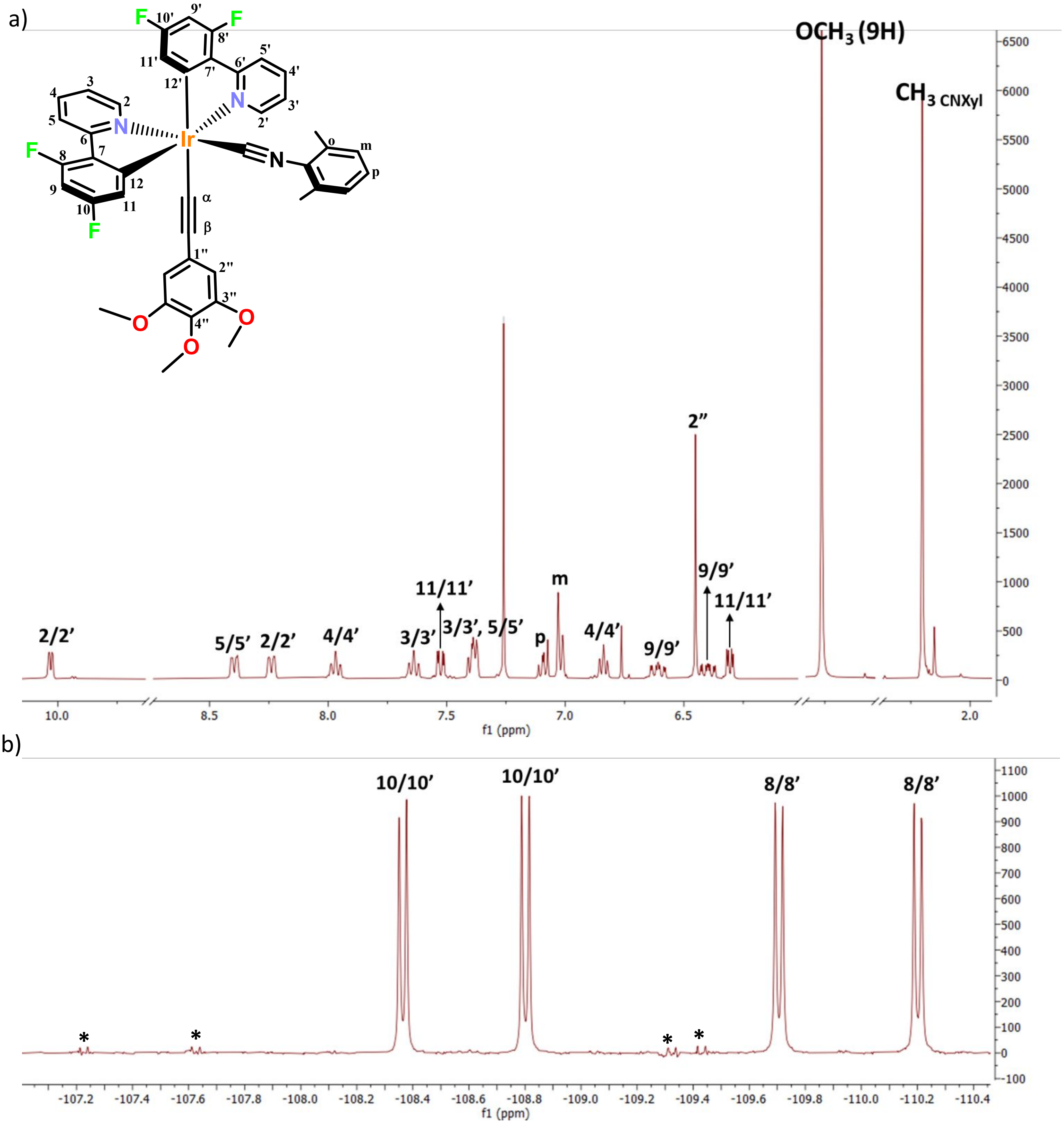

Figure S11. NMR spectra of ***N,N-cis*-(*OC*-6-32)-1b** in CD_3CN at 298 K a) ^{1}H and b) $^{19}F\{^{1}H\}$. Signals marked with* correspond to the isomer ***N,N-cis*-(*OC*-6-44)-1$_{Cl}$**

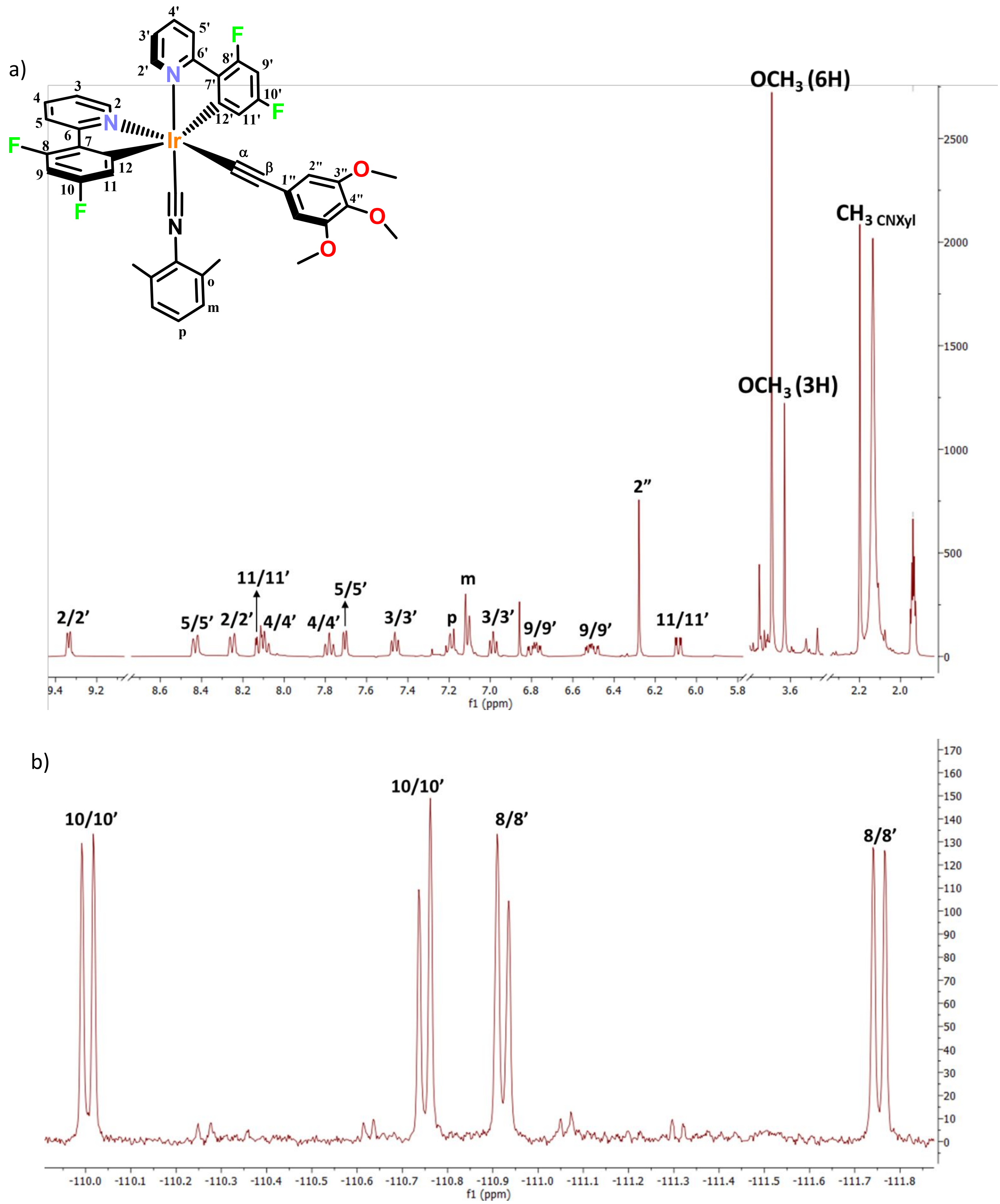

Figure S12. NMR spectra of ***C,C-trans*-1b** in CD_3CN at 298 K a) ^{1}H and b) $^{19}F\{^{1}H\}$

a)

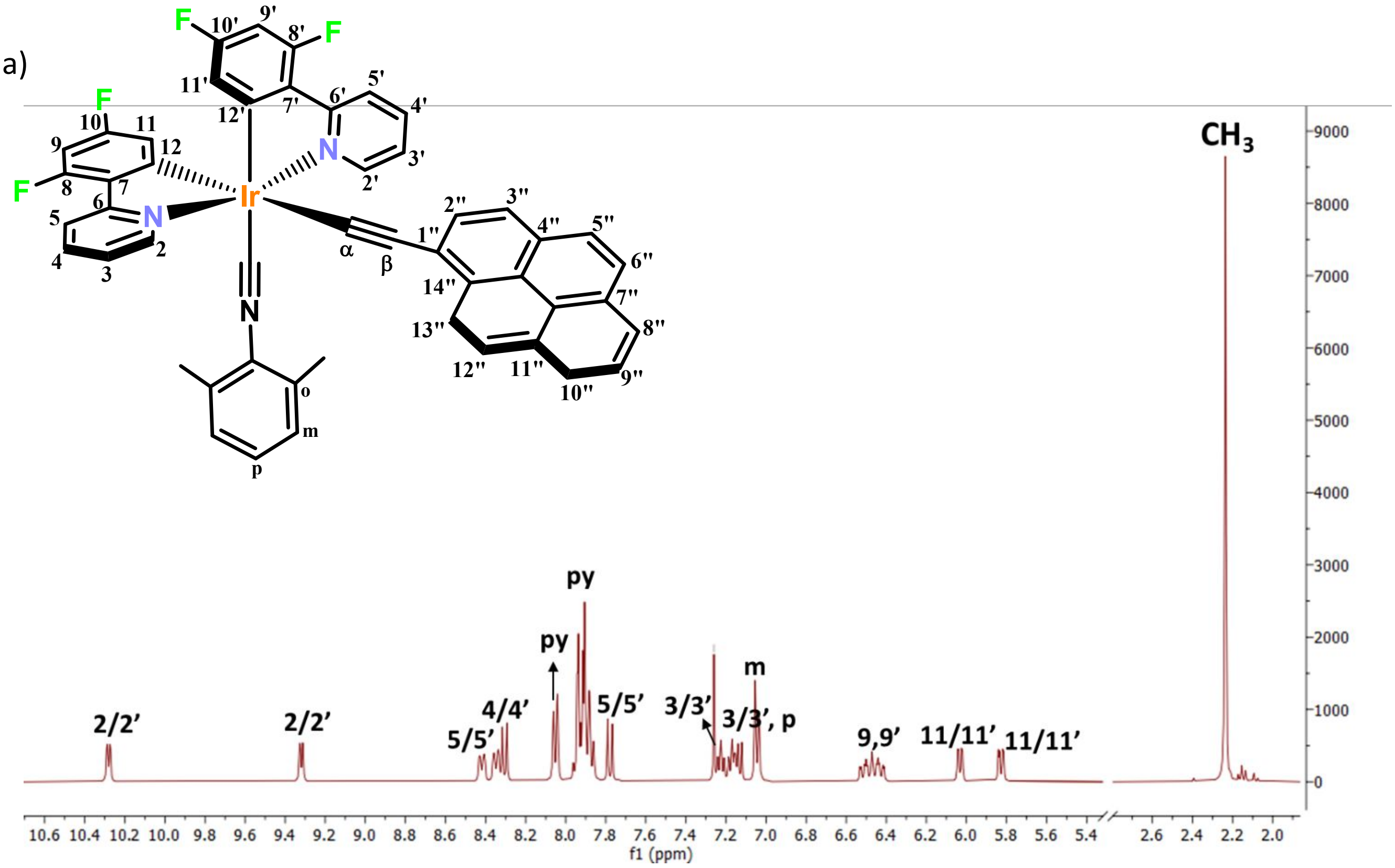
CH3
py
py
4/4'
5/5'
5/5'
3/3'
m
3/3', p
9,9'
11/11'
11/11'
2/2'
2/2'
f1 (ppm)

b)

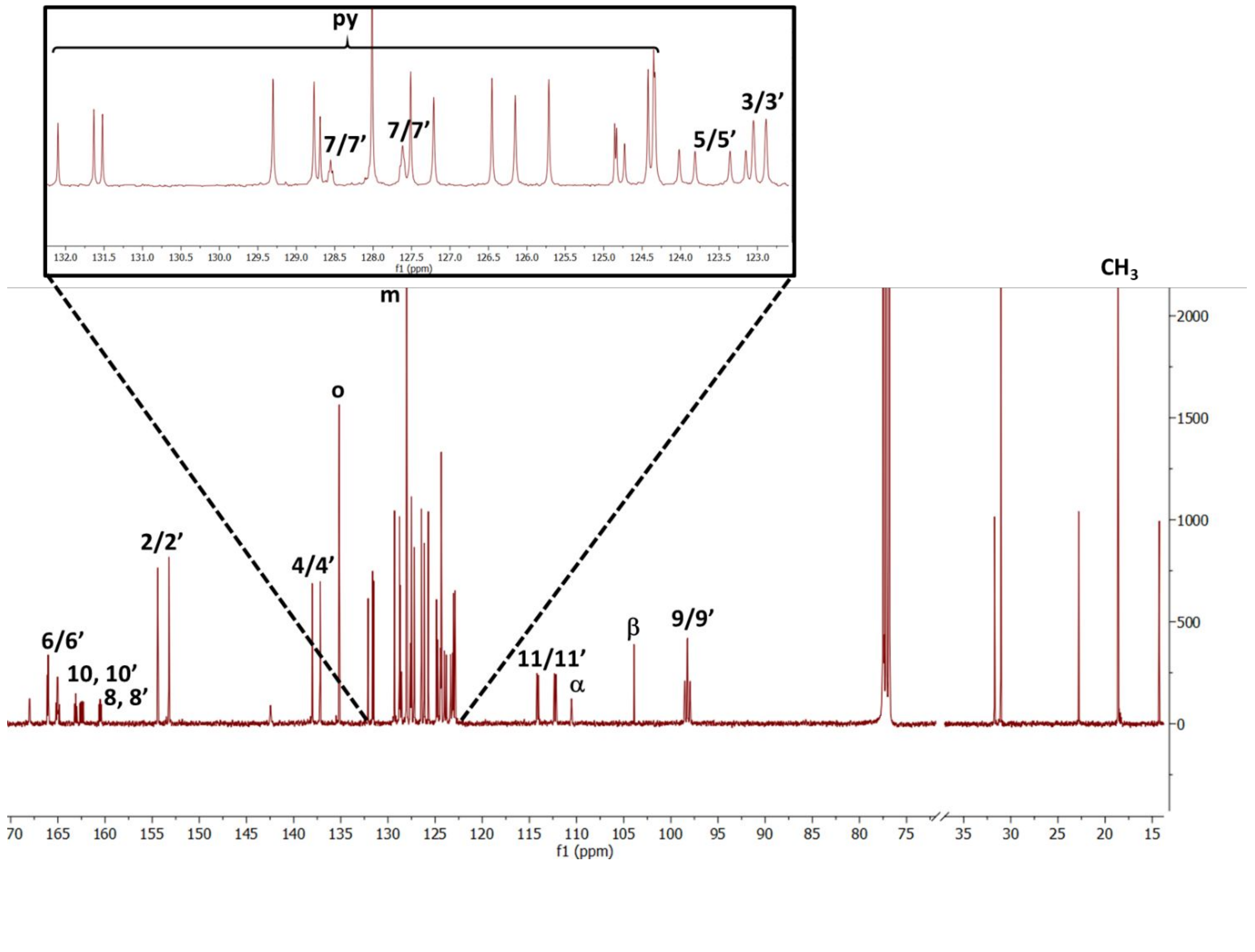
py
7/7'
7/7'
5/5'
3/3'
m
o
CH3
2/2'
4/4'
6/6'
10, 10'
8, 8'
11/11'
α
β
9/9'
f1 (ppm)

c)

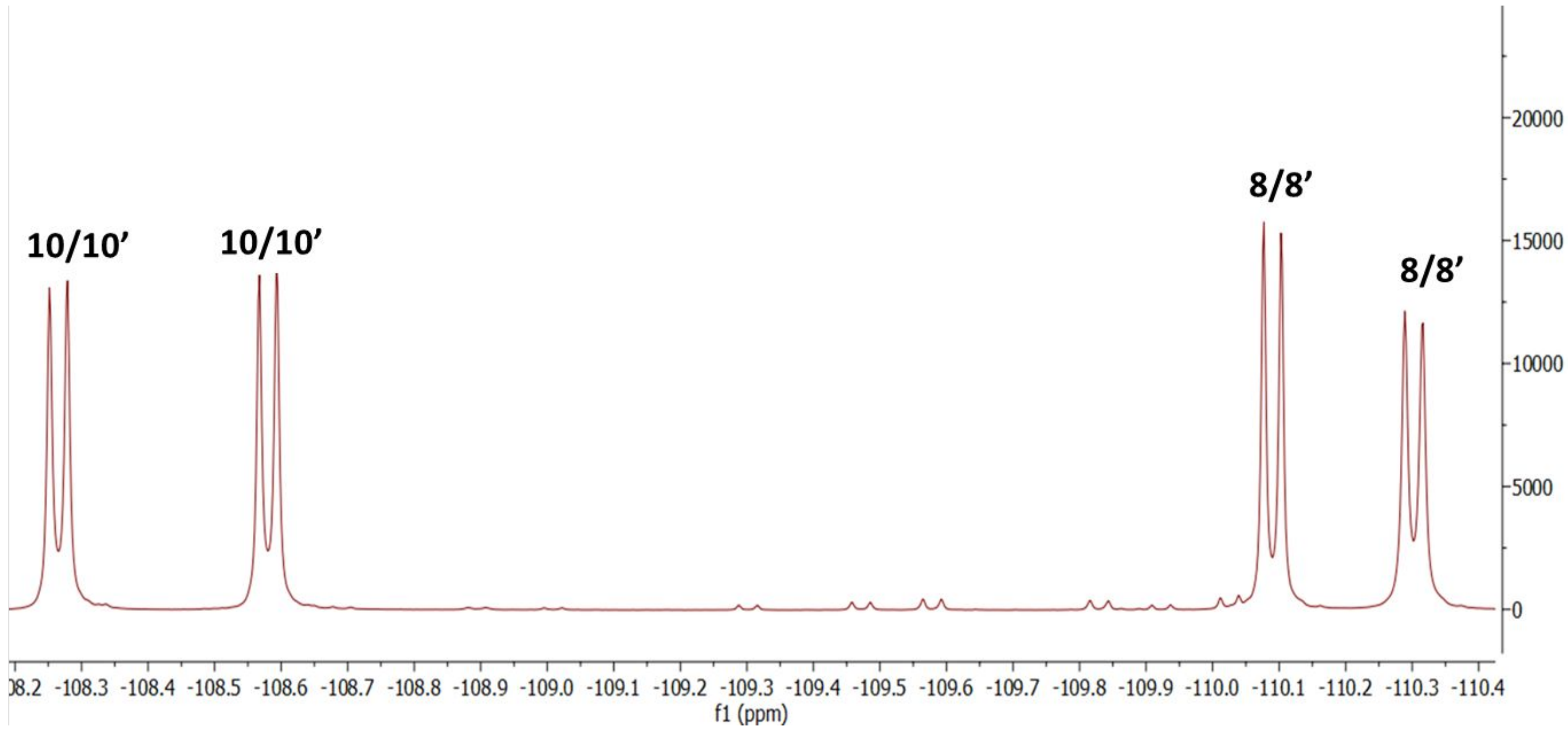

Figure S13. NMR spectra of ***N,N-trans*-1c** in $CDCl_3$ at 298 K a) ^1H; b) $^{13}C\{^1H\}$ and c) $^{19}F\{^1H\}$

a)

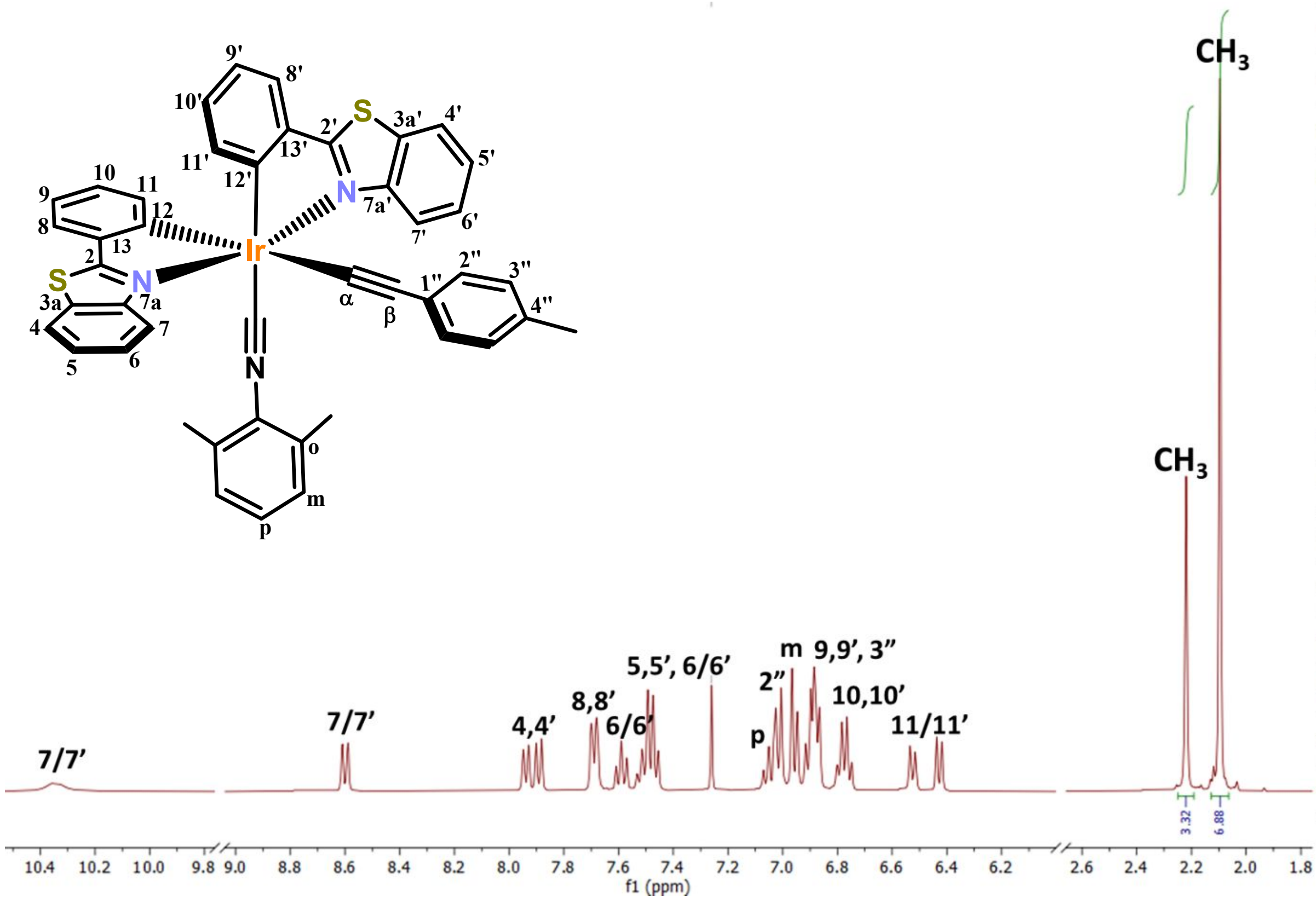

b)

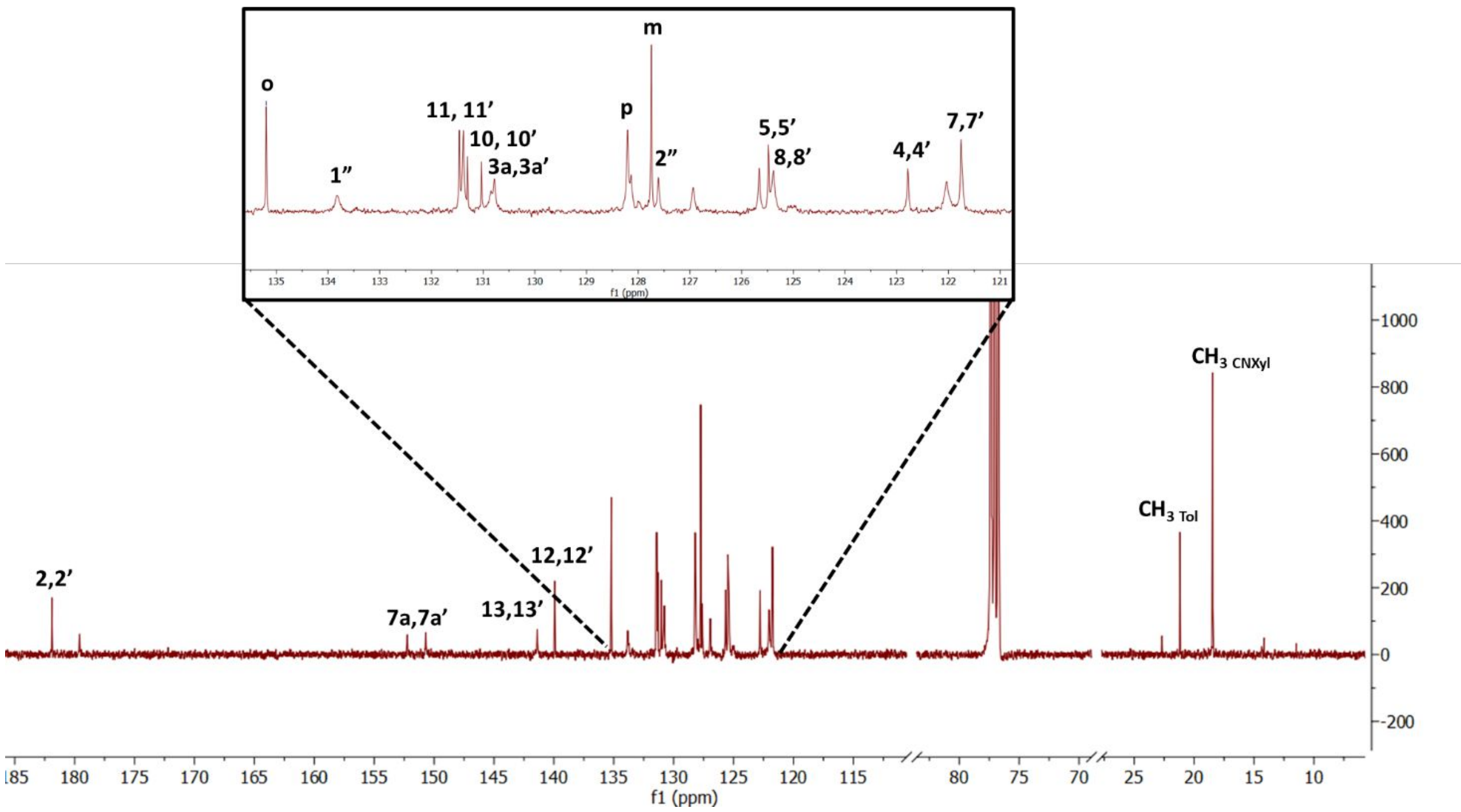

Figure S14. NMR spectra of ***N,N-trans*-2a** in $CDCl_3$ at 298 K a) ^1H; b) $^{13}C\{^1H\}$

a)

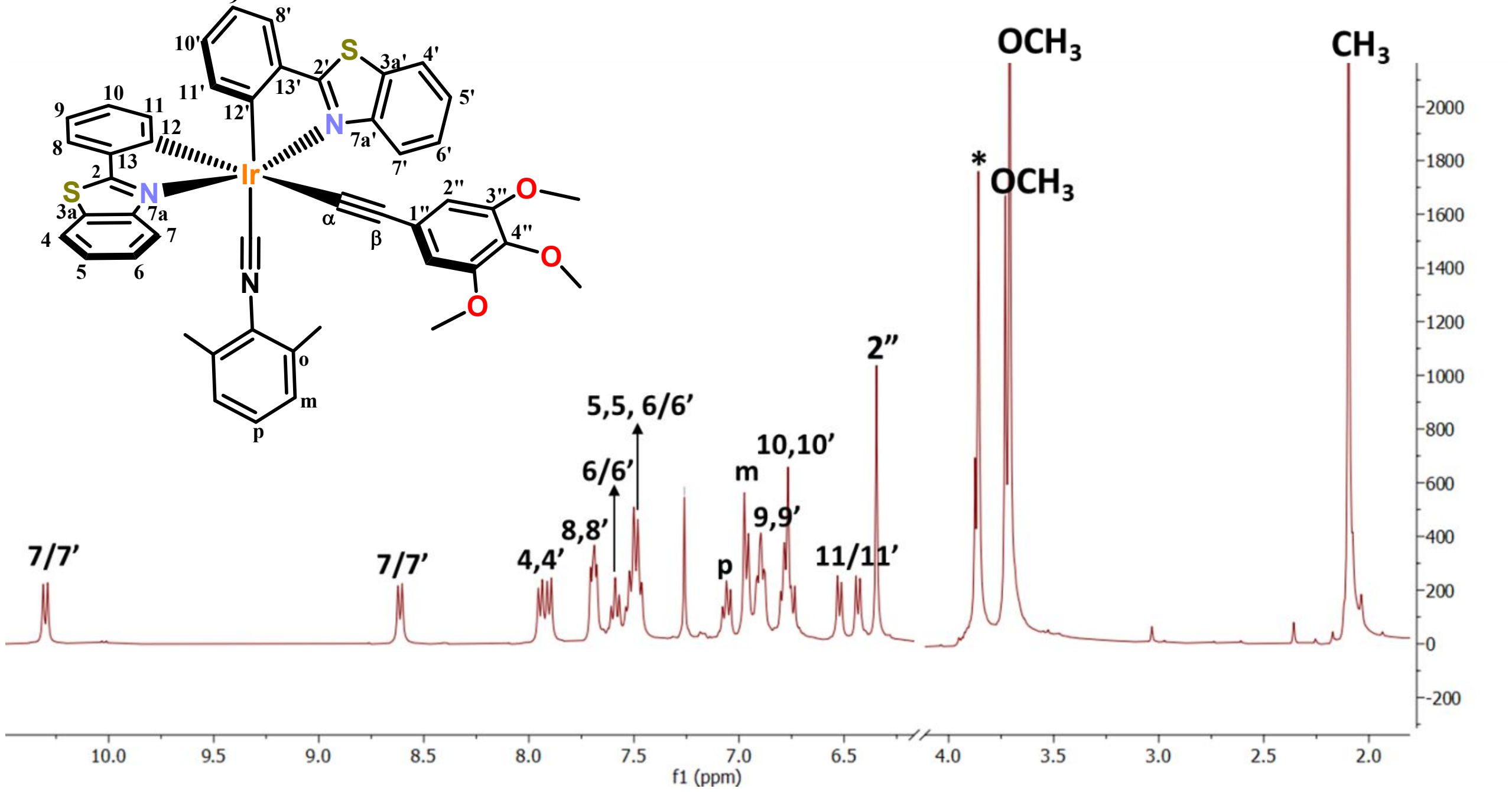

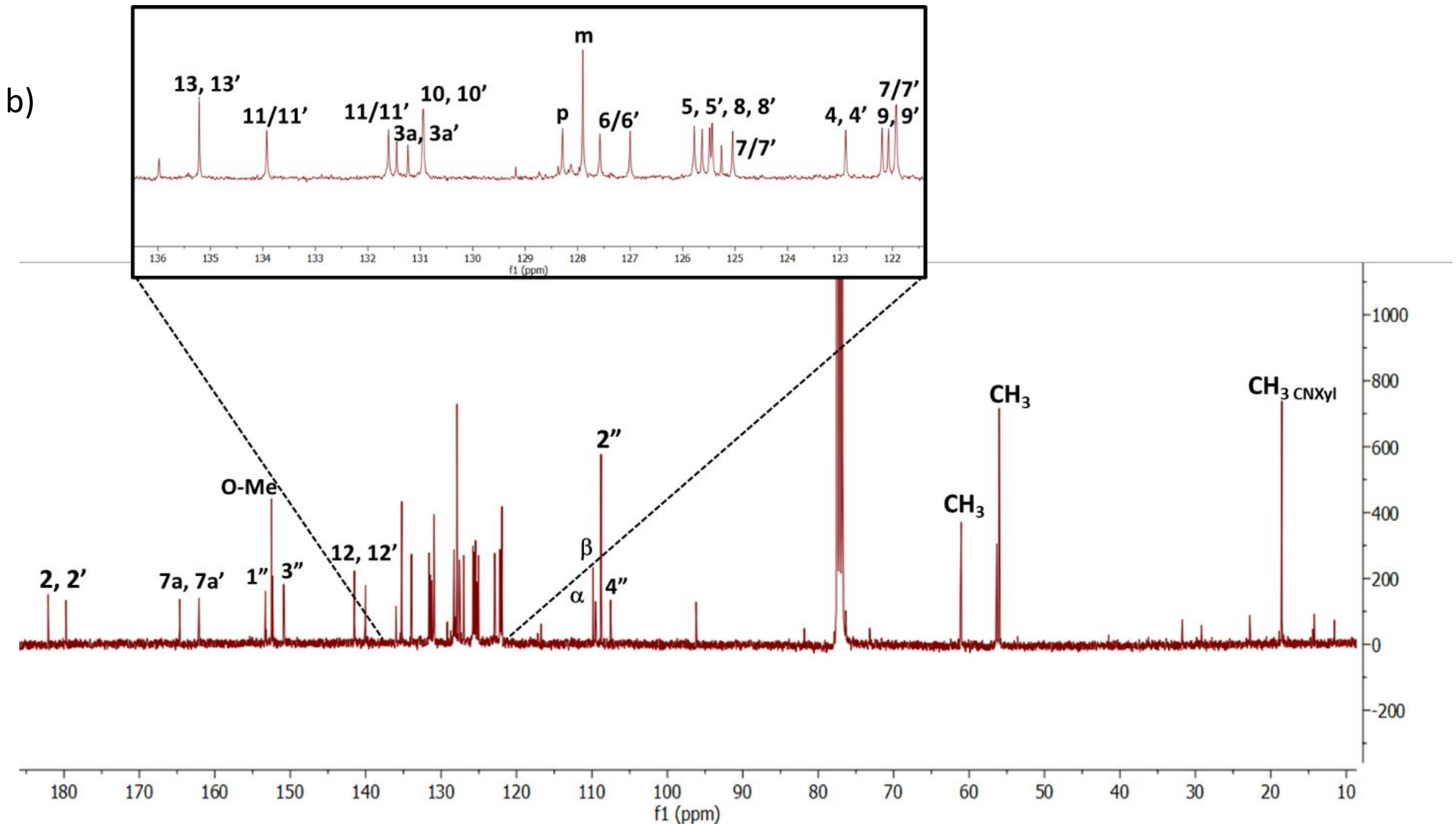

Figure S15. NMR spectra of ***N,N-trans*-2b** in $CDCl_3$ at 298 K a) ^{1}H (Signal marked with* corresponds to residual 1,2-dichloroethane); b) $^{13}C\{^{1}H\}$

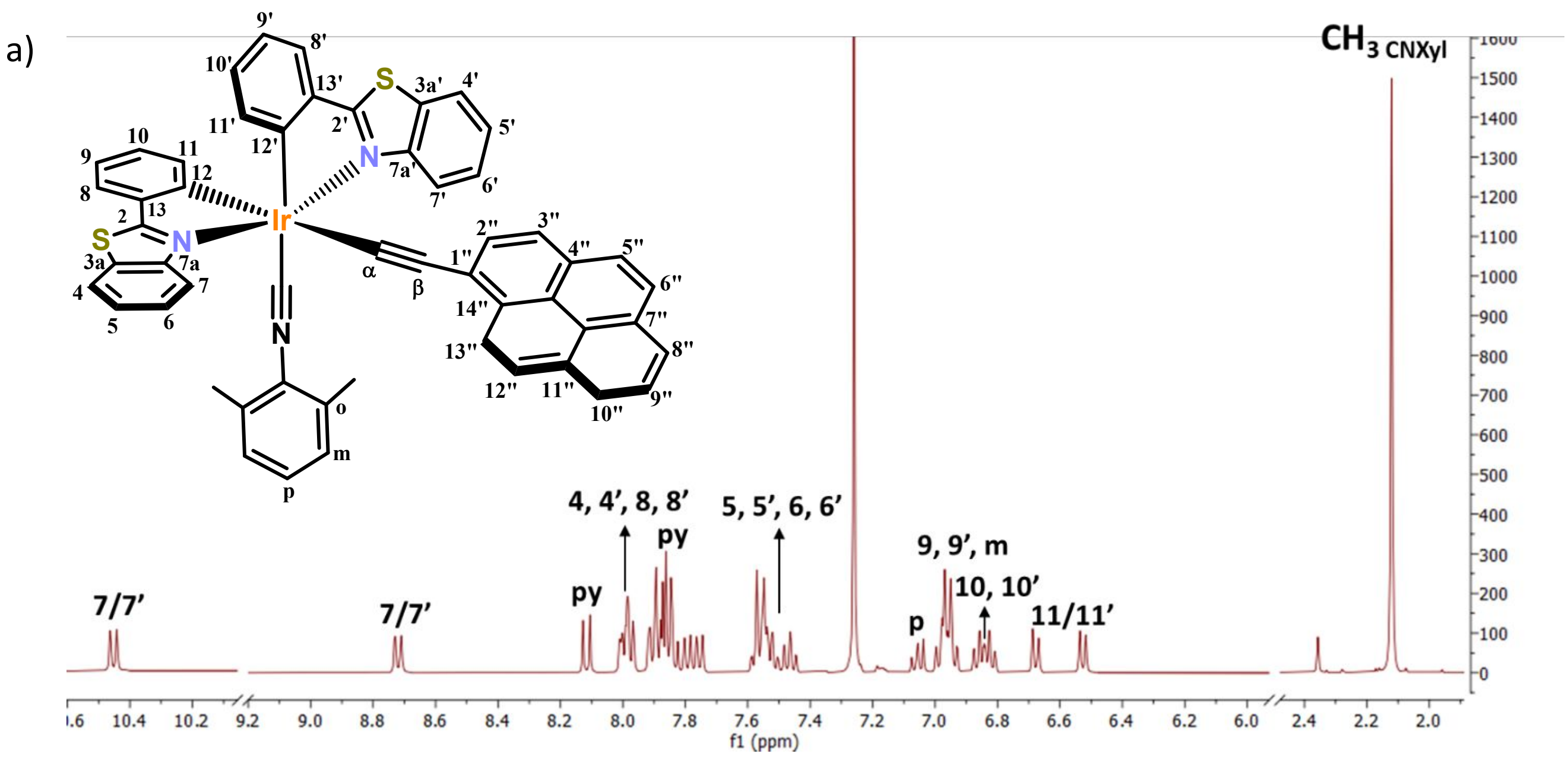

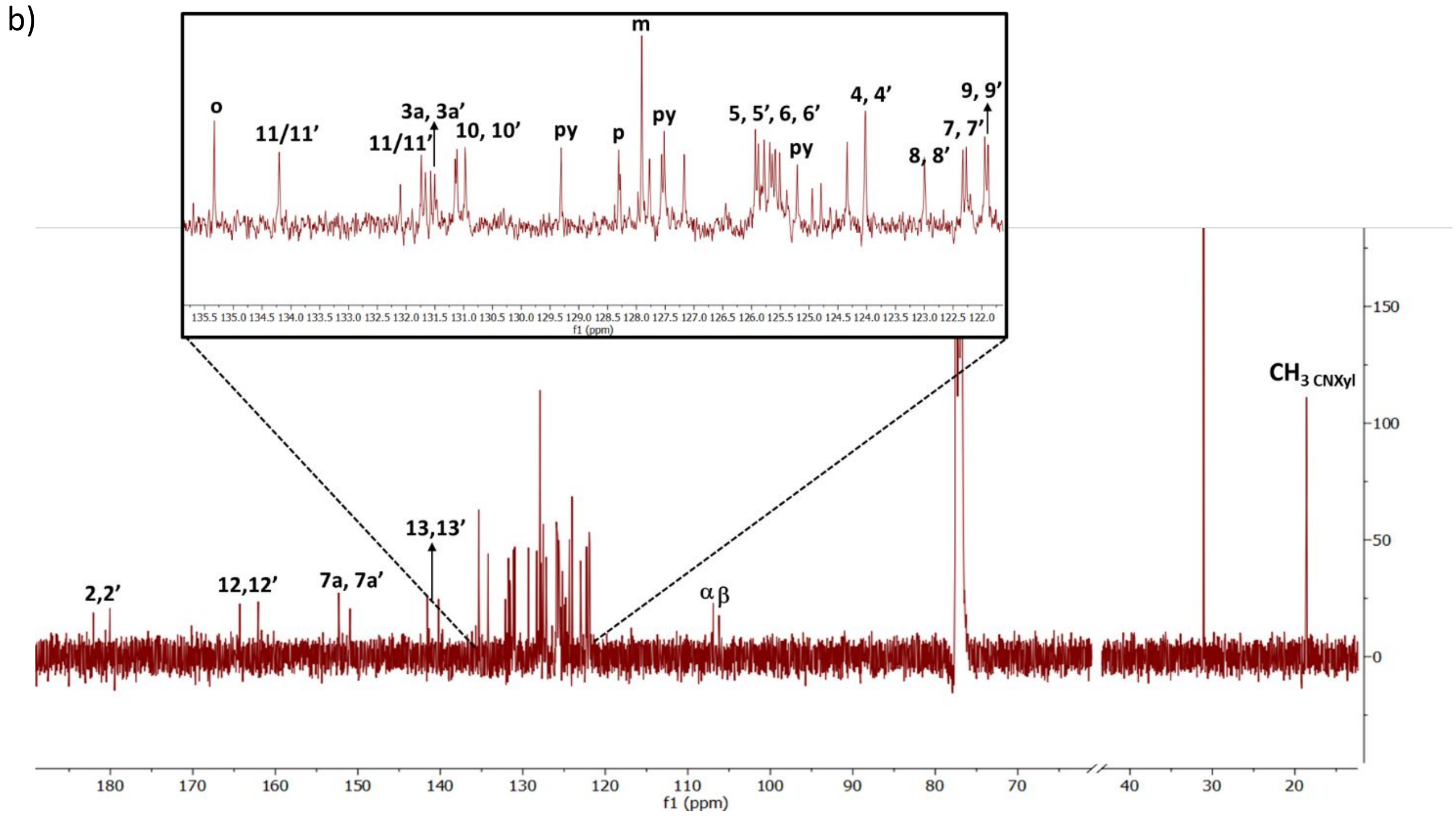

Figure S16. NMR spectra of ***N,N-trans*-2c** in $CDCl_3$ at 298 K a) ^{1}H; b) $^{13}C\{^{1}H\}$

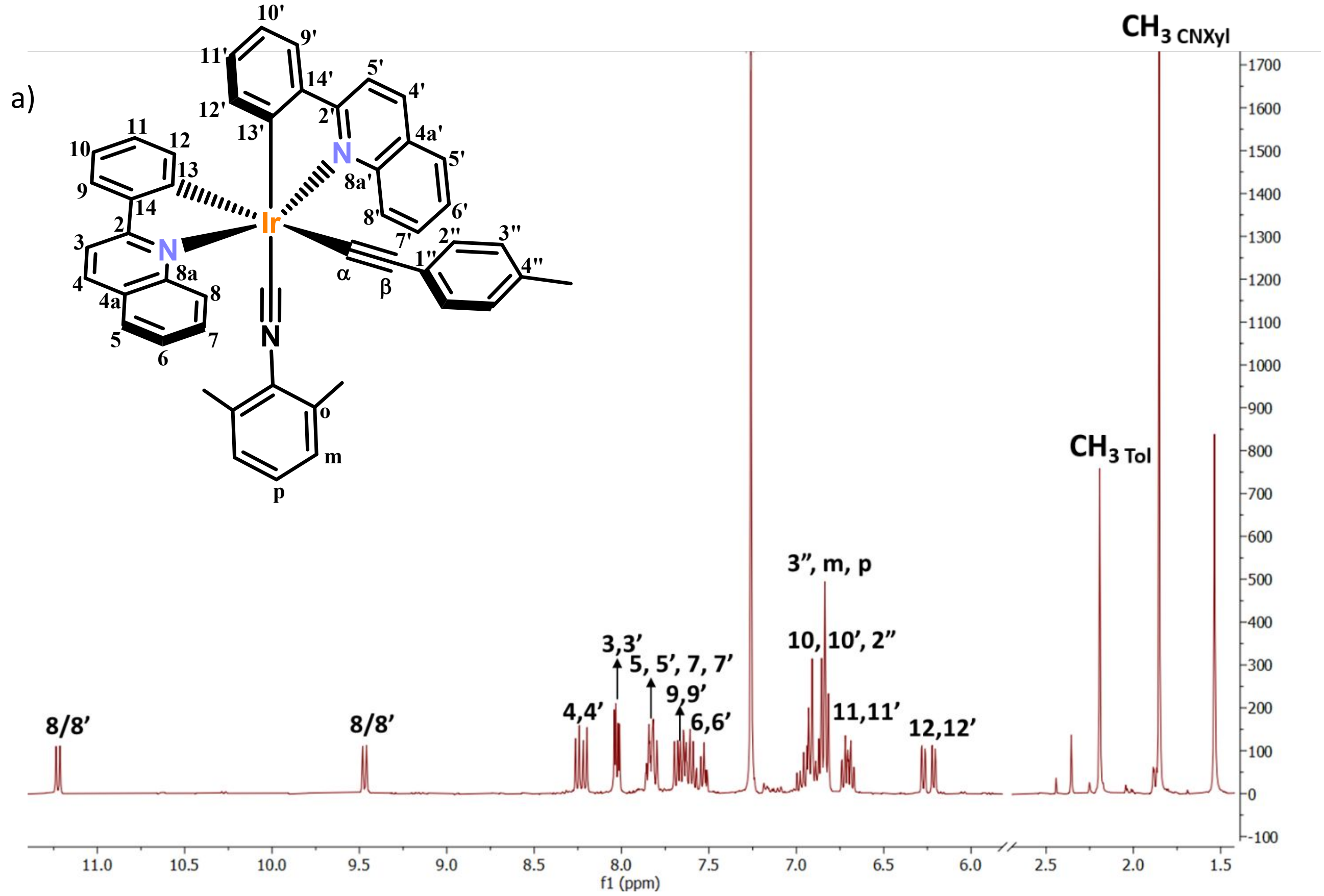

b)

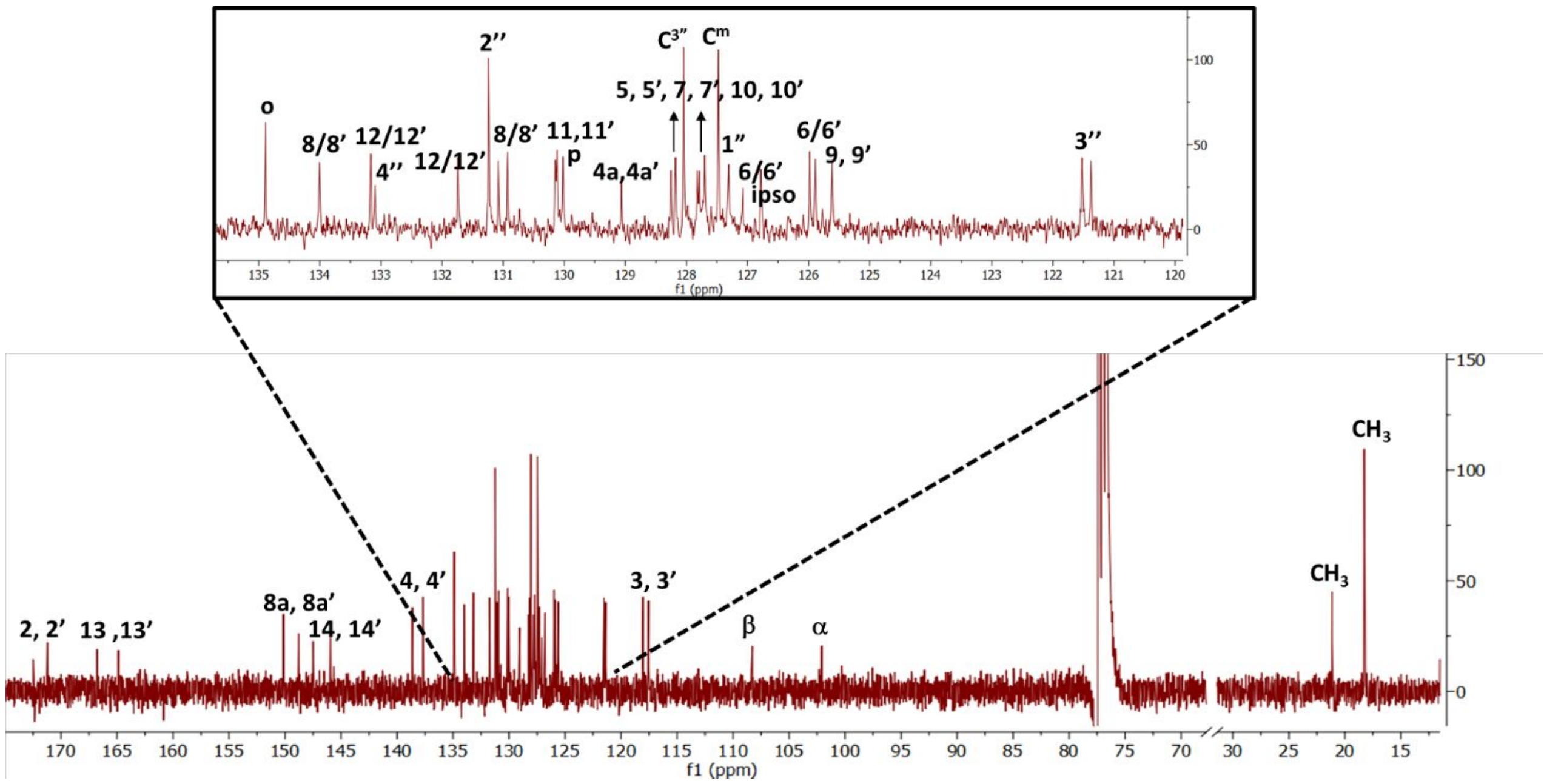

Figure S17. NMR spectra of ***N,N-trans*-3a** in $CDCl_3$ at 298 K a) ^1H; b) $^{13}C\{^1H\}$

a)

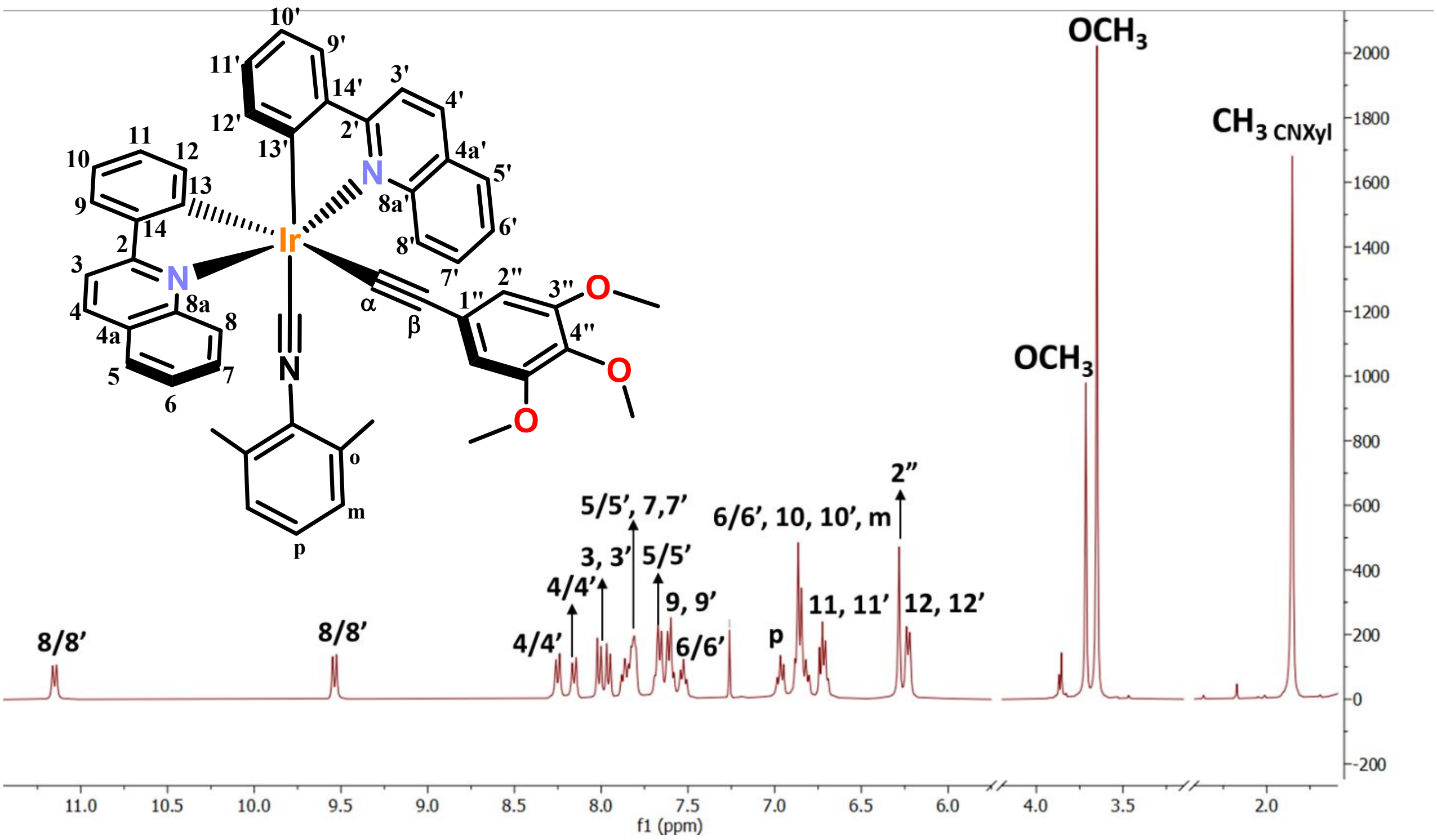

b)

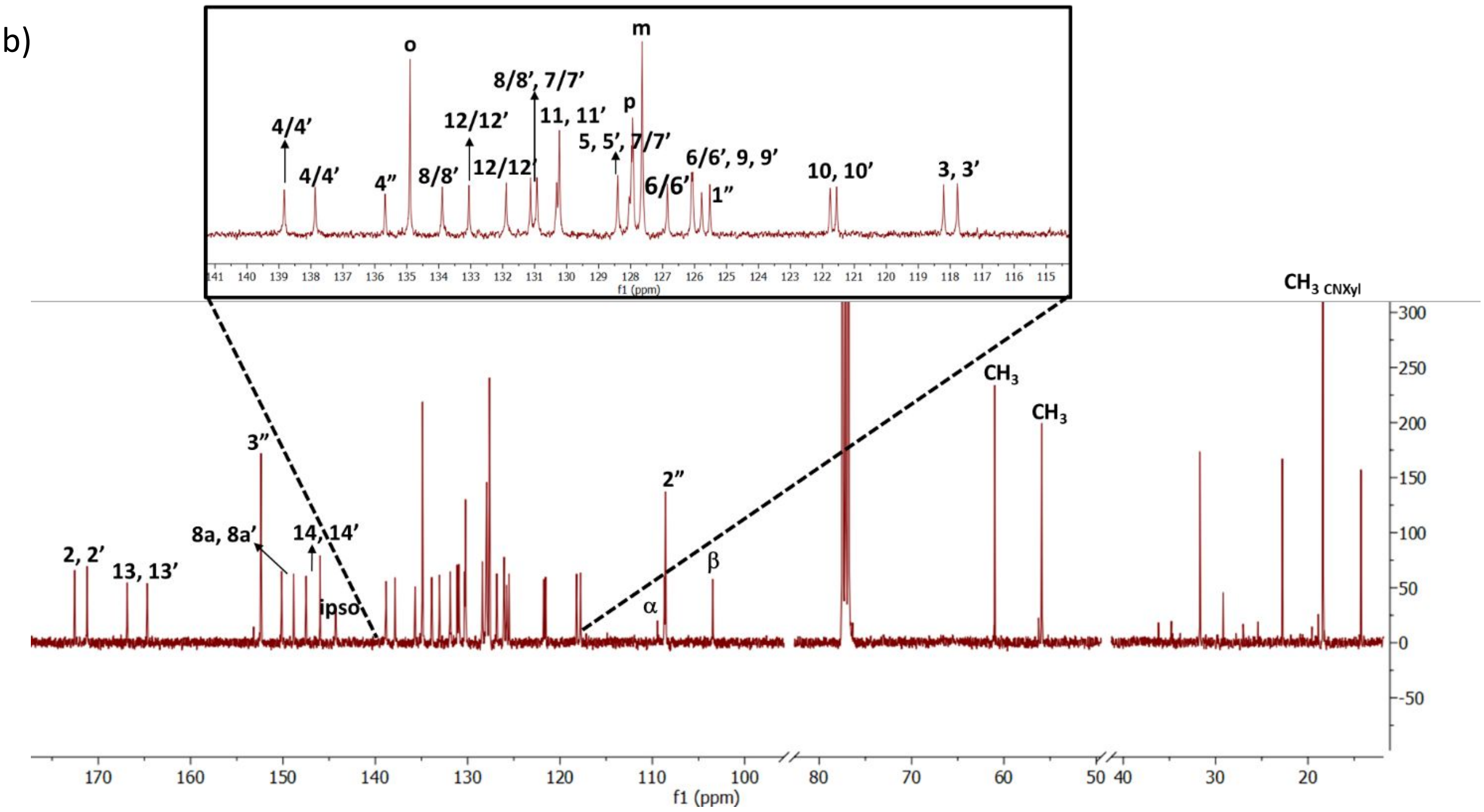

Figure S18. NMR spectra of ***N,N-trans*-3b** in $CDCl_3$ at 298 K a) ^1H; b) $^{13}C\{^1H\}$

a)

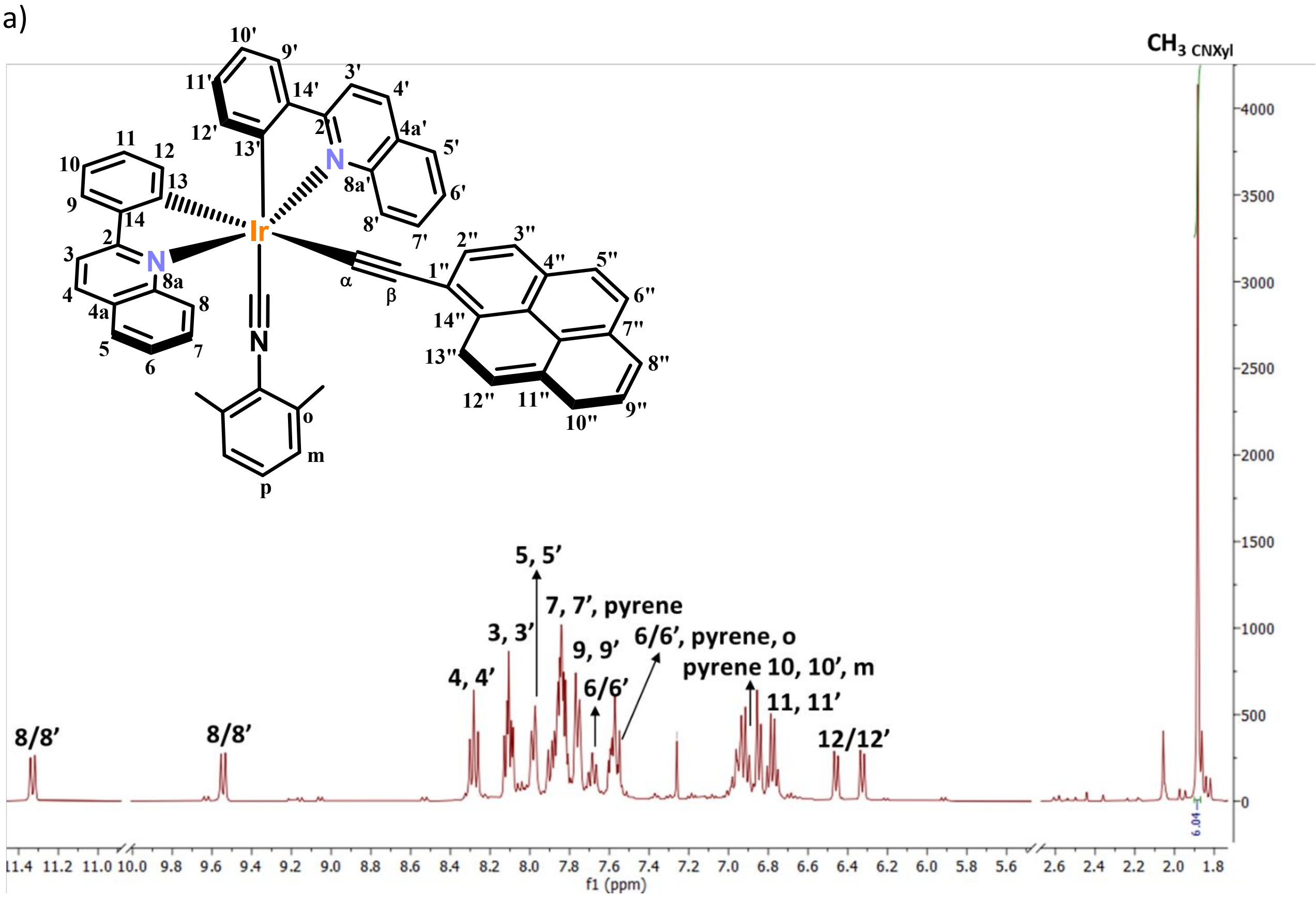

b)

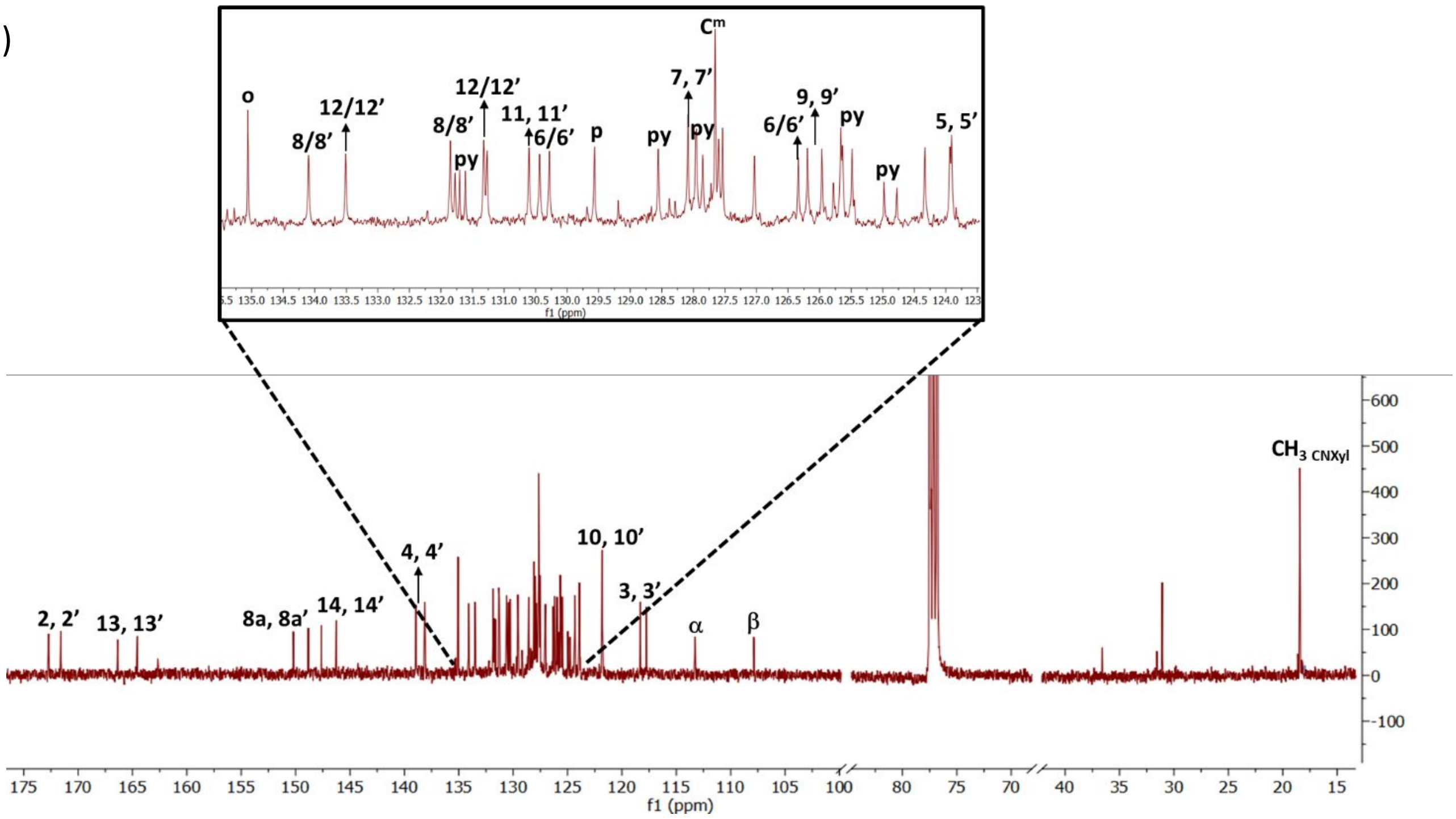

Figure S19. NMR spectra of ***N,N-trans*-3c** in $CDCl_3$ at 298 K a) ^1H; b) $^{13}C\{^1H\}$

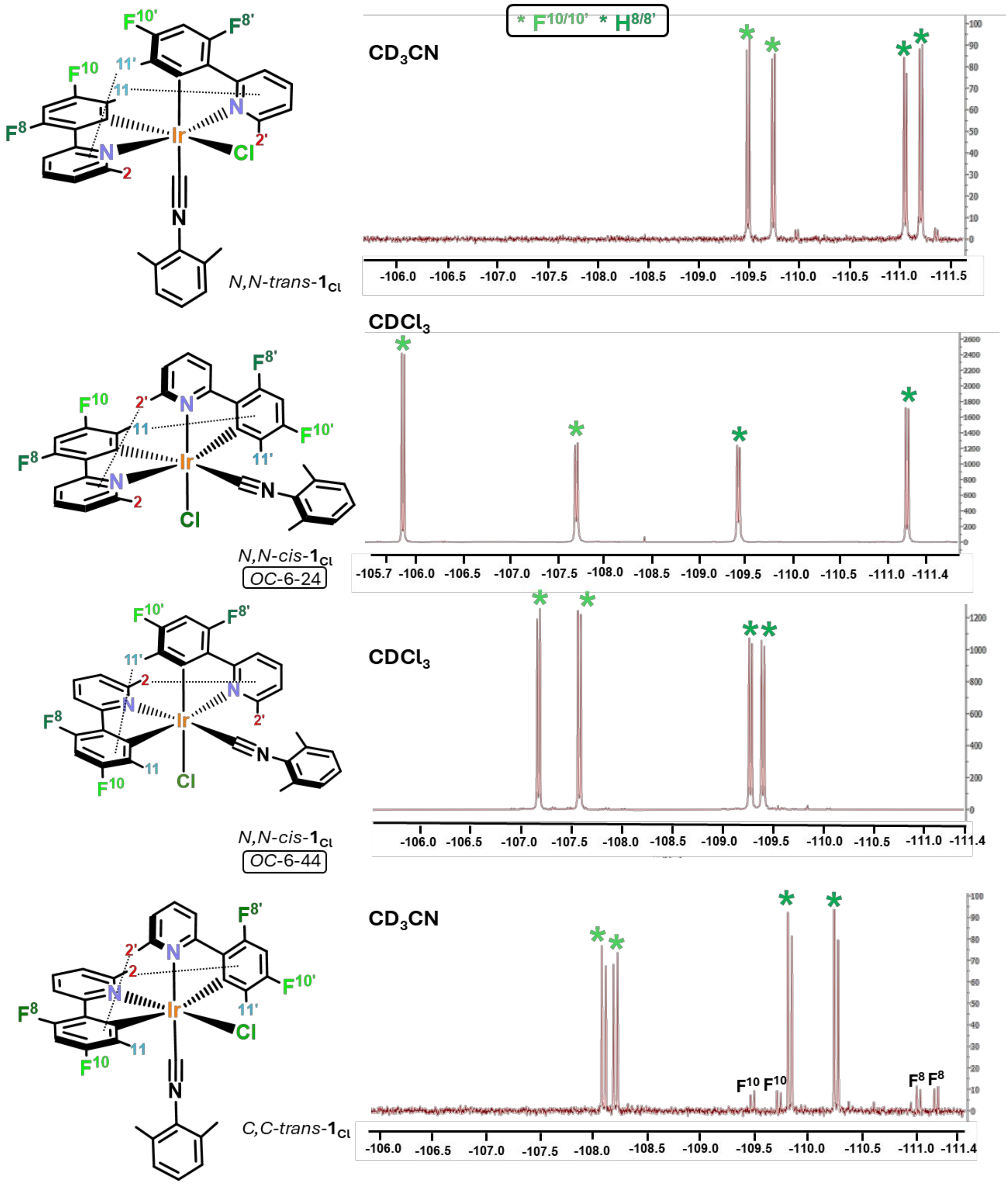

Figure S20. $^{19}F\{^{1}H\}$ NMR spectra of the isomers, ***N,N-trans*-1**$_{Cl}$, ***N,N-cis*-(*OC*-6-24)-1**$_{Cl}$, ***N,N-cis*-(*OC*-6-44)-1**$_{Cl}$ and ***C,C-trans*-1**$_{Cl}$. The superimposed structures on the spectra represent the location of fluorine atoms $F^{10/10'}$ (in light green) and $F^{8/8'}$ (in dark green). In the spectrum of the photoisomer ***C,C-trans*-1**$_{Cl}$, the additional signals corresponds to ***N,N-trans*-1**$_{Cl}$.

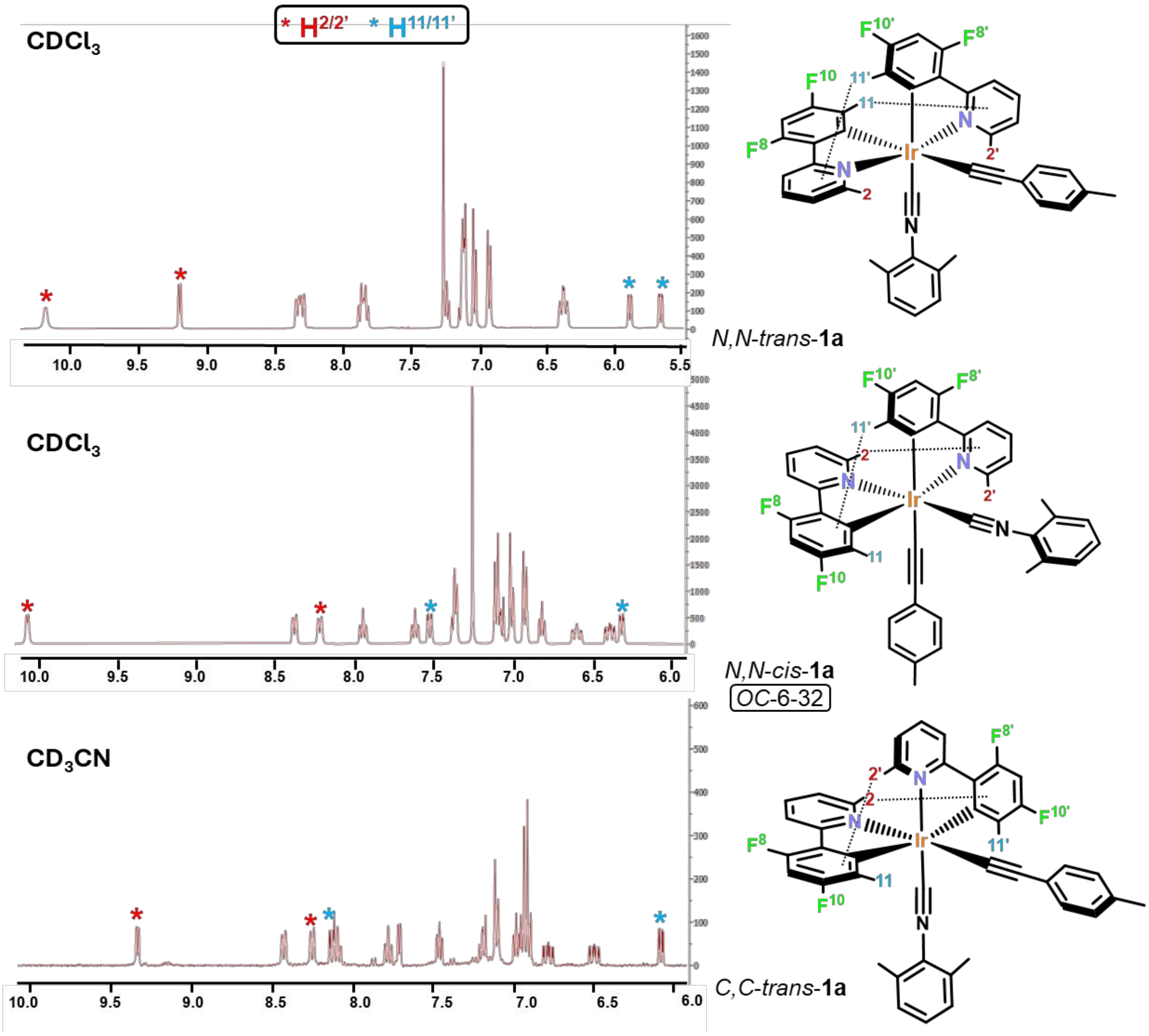

Figure S21. ^{1}H NMR spectra at 298 K of ***N,N-trans*-1a**, ***N,N-cis*-(*OC*-6-32)-1a**, and ***C,C-trans*-1a**.

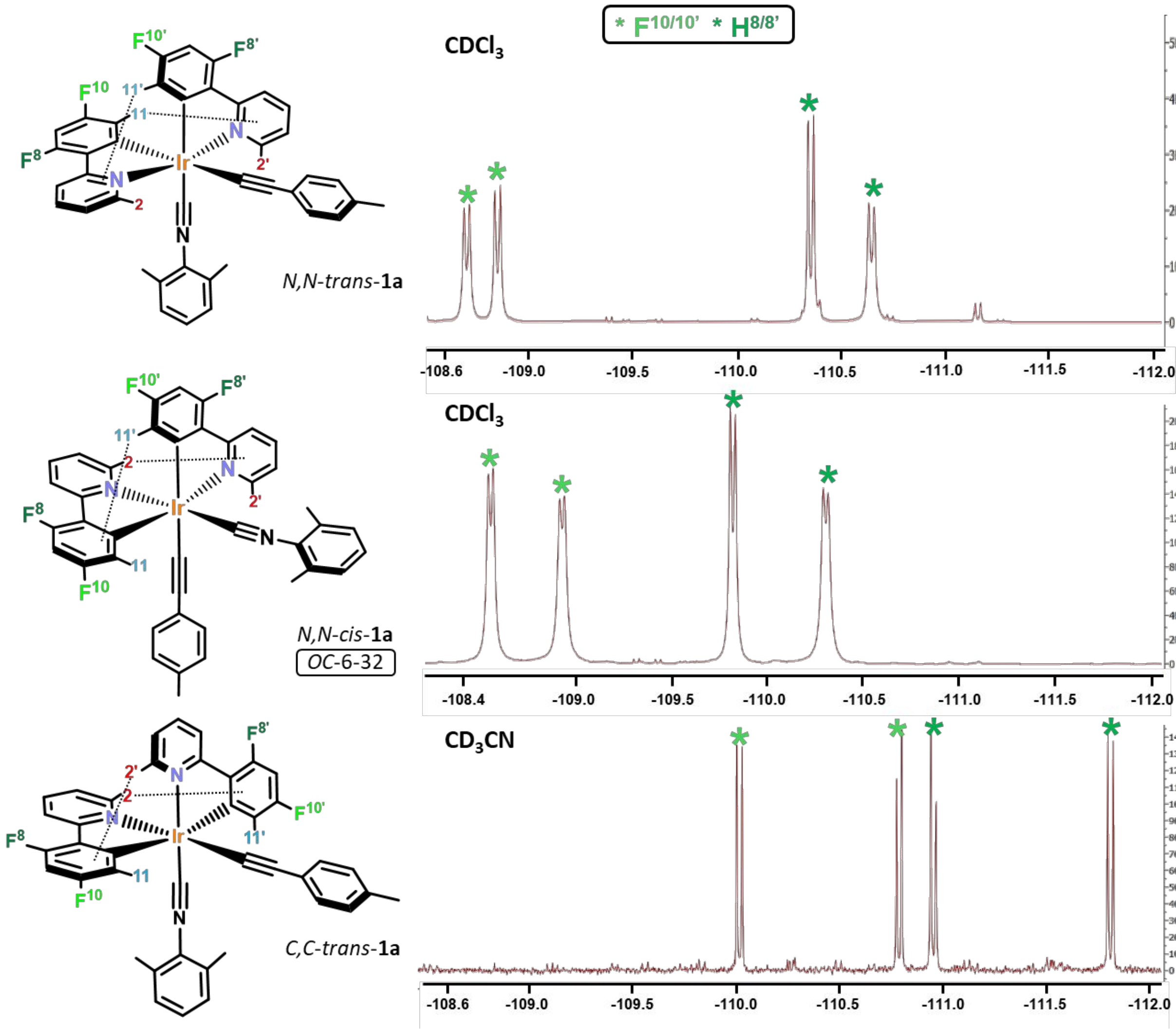

Figure S22. $^{19}F\{^1H\}$ NMR spectra of the isomers, ***N,N-trans*-1a**, ***N,N-cis*-(*OC*-6-32)-1a** in $CDCl_3$ and ***C,C-trans*-1a** in CD_3CN**.** The superimposed structures on the spectra represent the location of fluorine atoms $F^{10/10'}$ (in light green) and $F^{8/8'}$ (in dark green).

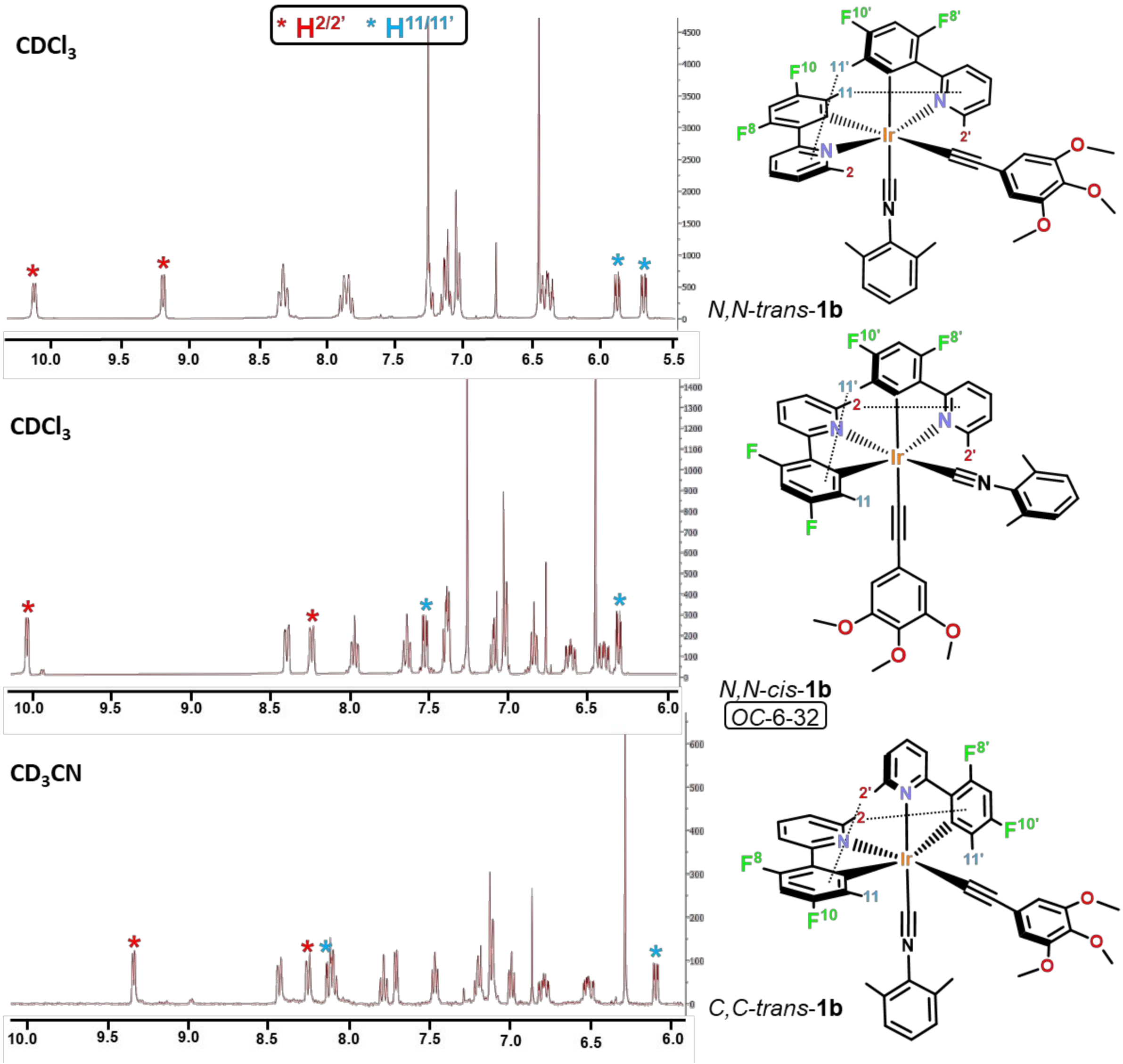

Figure S23. ^{1}H NMR spectra of the isomers, ***N,N-trans*-1b**, ***N,N-cis*-(*OC*-6-32)-1b** and ***C,C-trans*-1b.** The superimposed structures on the spectra represent the location of hydrogen atoms $H^{2/2'}$ (in red) and $H^{11/11'}$ (in blue).

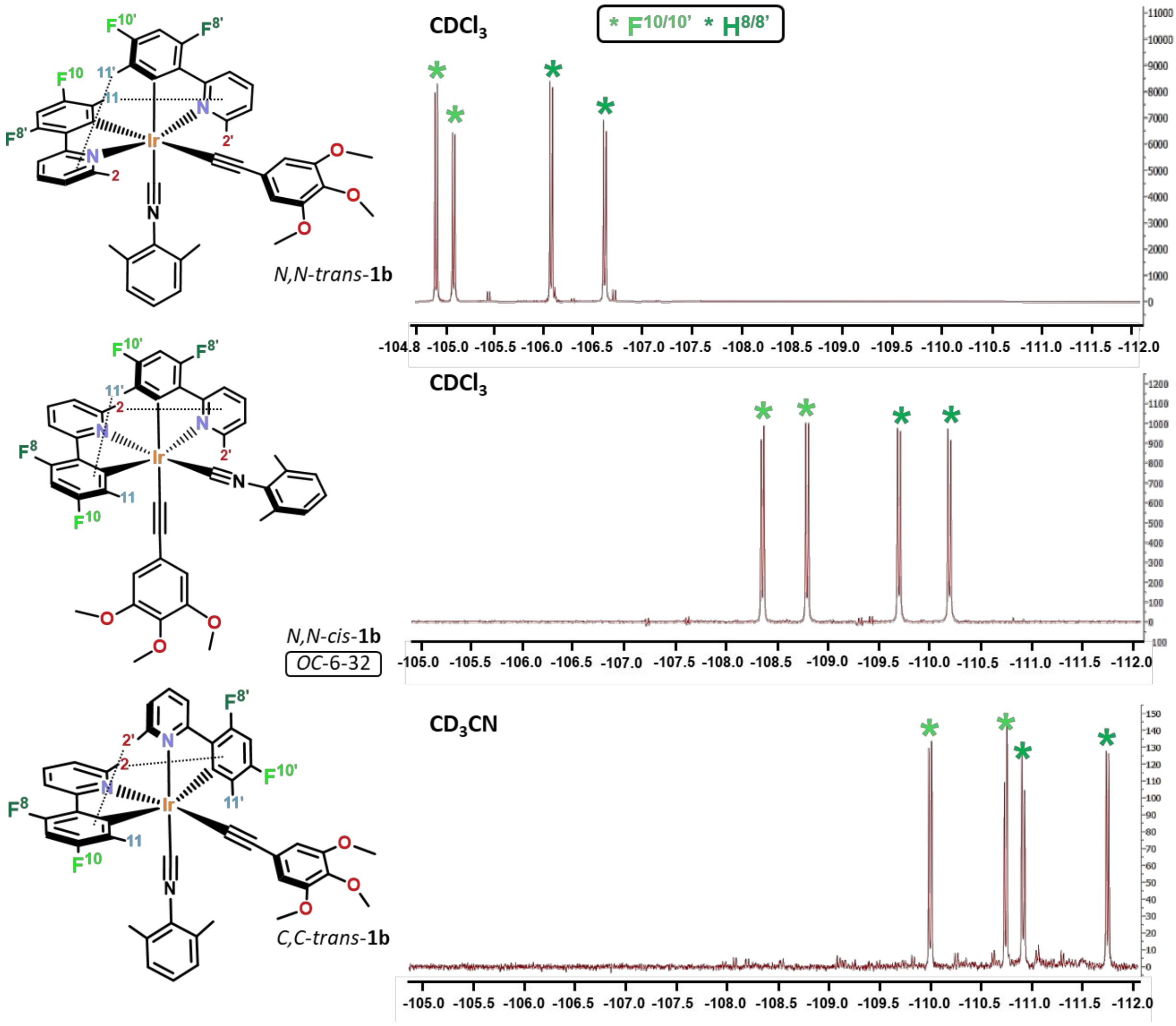

Figure S24. $^{19}F\{^1H\}$ NMR spectra of the isomers, ***N,N-trans*-1b**, ***N,N-cis*-(*OC*-6-32)-1b** and ***C,C-trans*-1b.** The superimposed structures on the spectra represent the location of fluorine atoms $F^{10/10'}$ (in light green) and $F^{8/8'}$ (in dark green).

3. X-Ray Diffraction Studies

Table S1. X-ray Crystallographic data for ***N,N-trans*-1$_{Cl}$·CH$_2$Cl$_2$**, ***N,N-cis*-(*OC*-6-24)-1$_{Cl}$**, ***N,N-cis*-(*OC*-6-44)-1$_{Cl}$** and ***N,N-trans*-3$_{Cl}$·CHCl$_3$**

	***N,N-trans*-1$_{Cl}$·CH$_2$Cl$_2$**	***N,N-cis-(OC*-6-24)-1$_{Cl}$**	***N,N-cis-(OC*-6-44)-1$_{Cl}$**	***N,N-trans*-3$_{Cl}$·CHCl$_3$**
**Empirical formula**	$C_{32}H_{23}Cl_3F_4IrN_3$	$C_{31}H_{21}ClF_4IrN_3$	$C_{31}H_{21}ClF_4IrN_3$	$C_{39}H_{29}ClIrN_3$
**Molecular weight**	824.08	739.16	739.16	767.30
**T (K)**	120(2) K	124(2)	120(2)	100(2) K
**Wavelength (Å)**	0.71076	0.71073 Å	0.71073	0.71076
**Crystal system**	Monoclinic	Triclinic	Monoclinic	Monoclinic
**Space group**	P 21/c	P -1	P 21/n	P 2_1/n
**Crystal size (mm)**	0.491 x 0.232 x 0.080	0.672 x 0.210 x 0.083	0.257 x 0.157 x 0.104	0.144 x 0.129 x 0.031
**a (Å)**	17.3020(19)	8.2250(5)	10.8107(11)	12.0721(5)
**b (Å)**	8.6908(9)	9.7771(6)	14.9098(13)	11.5078(5)
**c (Å)**	21.232(2)	18.1896(11)	16.9070(16)	25.2015(9)
**α (°)**	90°	88.331(2)	90	90
**β (°)**	113.209(3)	85.022(2)	103.421(3)	91.0390(10)
**γ (°)**	90°	66.420(2)	90	90
**V (Å^3)**	2934.2(5)	1335.53(14)	2650.7(4)	3500.5(2)
**Z**	4	2	4	4
**Density (calculated) (Mg/cm^3)**	1.865	1.838	1.852	1.456
**Absorption coefficient (mm^{-1})**	4.878	5.153	5.193	3.920
**F(000)**	1600	716	1432	1512
**θ range for data collection (°)**	2.562 to 26.373	2.530 to 25.681°	2.370 to 27.939	2.397 to 27.877
**Index ranges**	-22<=h<=20, 0<=k<=11, 0<=l<=27	-10<=h<=10, -11<=k<=11, -22<=l<=22	-13<=h<=13, -18<=k<=18, -20<=l<=20	-15<=h<=15, -15<=k<=15, -33<=l<=33
**Reflections collected**	5564	67890	127649	156463
**Independent reflections**	5564 [R(int) = 0.0189]	5042 [R(int) = 0.0443]	5035 [R(int) = 0.0345]	8328 [R(int) = 0.1018]
**Data / restraints / parameters**	5564 / 0 / 373	5042 / 0 / 361	5035 / 0 / 363	8328 / 0 / 397
**Goodness-of-fit on F2**	1.071	1.065	1.322	1.154
**Final R indices [I>2σ(I)][a]**	R1 = 0.0189, wR2 = 0.0473	R1 = 0.0181, wR2 = 0.0504	R1 = 0.0180, wR2 = 0.0394	R_1 = 0.0444, wR_2 = 0.0849
**R indices (all data)[a]**	R1 = 0.0195, wR2 = 0.0476	R1 = 0.0184, wR2 = 0.0507	R1 = 0.0192, wR2 = 0.0398	R_1 = 0.0647, wR_2 = 0.0914
**Largest diff. peak and hole (e Å^{-3}) (dmin/dmax)**	1.116 and -1.368	0.737 and -1.182	1.014 and -0.778	1.059 and -2.363

[a] $R_1 = \Sigma(|F_o| - |F_c|)/\Sigma|F_o|$; $wR_2 = [\Sigma w(F_o^2 - F_c^2)^2/\Sigma wF_o^2]^{1/2}$; goodness of fit = $\{\Sigma[w(F_o^2 - F_c^2)^2]/(N_{obs} - N_{param})\}^{1/2}$; $w = [\sigma^2(F_o) + (g_1P)^2 + g_2P]^{-1}$; $P = [\max(F_o^2;0 + 2F_c^2]/3$.

Table S2. X-ray Crystallographic data for ***N,N-trans*-1a, *N,N-trans*-1c·0.6 $CHCl_3$**, ***N,N-trans*-2a**, ***N,N-trans*-2c**, ***N,N-cis*-(*OC*-6-32)-1a** and ***N,N*-cis-(*OC*-6-32)-1b.**

	***N,N-trans*-1a**	***N,N-trans*-1c·0.6 $CHCl_3$**	***N,N-trans*-2a**	***N,N-trans*-2c**	***N,N*-cis-(*OC*-6-32)-1a·1.75 CH_2Cl_2**	***N,N*-cis-(*OC*-6-32)-1b·0.9 CH_2Cl_2**
**Empirical formula**	$C_{40}H_{28}F_4IrN_3$	$C_{49}H_{30}F_4IrN_3$	$C_{44}H_{32}IrN_3S_2$	$C_{54}H_{35}Cl_3IrN_3S_2$	$C_{40}H_{28}F_4IrN_3$	$C_{42}H_{32}F_4IrN_3O_3$
**Molecular weight**	818.85	928.96	859.04	1088.52	818.85	894.90
**T (K)**	100(2)	100(2)	150(2)	150(2)	125(2)	146(2)
**Wavelength (Å)**	0.71076	0.71076	0.71073	0.71076	0.71073	0.71076
**Crystal system**	Triclinic	Monoclinic	Monoclinic	Monoclinic	Triclinic	Monoclinic
**Space group**	P $_{-1}$	P 2_1/n	P 2_1/n	P 2_1/n	P -1	P 21/n
**Crystal size (mm)**	0.260 x 0.152 x 0.141	0.200 x 0.197 x 0.152	0.333 x 0.202 x 0.079	0.260 x 0.112 x 0.090	0.707 x 0.270 x 0.087	0.172 x 0.134 x 0.116
**a (Å)**	7.8956(18)	13.723(3)	14.8022(14)	16.4656(17)	10.8163(12)	14.196(2)
**b (Å)**	12.985(3)	12.653(3)	15.5912(14)	12.8790(11)	11.0100(14)	14.916(2)
**c (Å)**	17.574(4)	23.878(5)	15.7437(15)	21.455(2)	16.6649(19)	18.781(3)
**α (°)**	105.328(8)	90	90	90	89.367(4)	90
**β (°)**	95.165(8)	99.298(8)	100.666(3)	98.695(4)	71.558(4)	97.107(6)
**γ (°)**	106.251(8)	90	90°	90	71.284(4)	90
**V (Å^3)**	1641.9(6)	4091.5(17)	3570.6(6)	4497.5(7)	1774.0(4)	3946.2(11)
**Z**	2	4	4	4	2	4
**Density (calculated) (Mg/cm^3)**	1.656	1.508	1.598	1.608	1.533	1.506
**Absorption coefficient (mm^{-1})**	4.122	3.319	3.893	3.282	3.815	3.443
**F(000)**	804	1832	1704	2160	804	1768
**θ range for data collection (°)**	2.727 to 25.683	2.203 to 25.683	2.466 to 25.681	2.314 to 27.876	2.076 to 25.681	2.181 to 25.678
**Index ranges**	-9<=h<=9 -15<=k<=15 -21<=l<=21	-16<=h<=16 -15<=k<=15 -29<=l<=29	-19<=h<=19 -20<=k<=20 -20<=l<=20	-21<=h<=21, -16<=k<=16, -28<=l<=28	-13<=h<=13, -13<=k<=13, -20<=l<=20	-17<=h<=17, -18<=k<=-17, -22<=l<=22
**Reflections collected**	76821	246171	6766	208265	96284	196470
**Independent reflections**	6236 [R(int) = 0.0620]	7748 [R(int) = 0.0552]	6766 [R(int) = 0.0889]	10691 [R(int) = 0.0315]	6758 [R(int) = 0.0473]	7485 [R(int) = 0.0670]

Data / restraints / parameters	6236 / 1 / 427	7748 / 0 / 514	6766 / 0 / 451	10691 / 13 / 478	6758 / 0 / 433	7485 / 0 / 479
Goodness-of-fit on F2	1.119	1.156	1.075	1.078	1.323	0.910
Final R indices [I>2σ(I)][a]	R_1 = 0.0413, wR_2 = 0.1091	R_1 = 0.0409, wR_2 = 0.1123	R_1 = 0.0298, wR_2 = 0.0687	R_1 = 0.0494, wR_2 = 0.1269	R1 = 0.0345, wR2 = 0.0981	R1 = 0.0576, wR2 = 0.1272
R indices (all data)[a]	R_1 = 0.0418, wR_2 = 0.1094	R_1 = 0.0414, wR_2 = 0.1127	R_1 = 0.0365, wR_2 = 0.0707	R_1 = 0.0591, wR_2 = 0.1369	R1 = 0.0349, wR2 = 0.0983	R1 = 0.0770, wR2 = 0.1577
Largest diff. peak and hole (e Å^{-3}) (dmin/dmax)	5.730 and -1.820	3.498 and -2.310	2.966 and -0.842	2.121 and -1.595	2.493 and -2.354	4.707 and -2.587 e.Å^{-3}

[a] $R_1 = \Sigma(|F_o| - |F_c|)/\Sigma|F_o|$; $wR_2 = [\Sigma w(F_o^2 - F_c^2)^2/\Sigma wF_o^2]^{1/2}$; goodness of fit = $\{\Sigma[w(F_o^2 - F_c^2)^2]/(N_{obs} - N_{param})\}^{1/2}$; $w = [\sigma^2(F_o) + (g_1P)^2 + g_2P]^{-1}$; $P = [\max(F_o^2;0 + 2F_c^2]/3$.

Table S3. Selected distances (Å) and angles (º) for complexes ***N,N-trans*-1$_{Cl}$·CH_2Cl_2**, ***N,N-cis-(OC*-6-24)-1$_{Cl}$**, ***N,N-cis-(OC*-6-44)-1$_{Cl}$** and ***N,N-trans*-3$_{Cl}$·$CHCl_3$**

Complexes	*N,N-trans*-1$_{Cl}$·CH_2Cl_2	*N,N-cis-(OC*-6-24)-1$_{Cl}$	*N,N-cis-(OC*-6-44)-1$_{Cl}$	*N,N-trans*-3$_{Cl}$·$CHCl_3$
**Distances (Å)**				
N(1)-Ir(1)	2.052(2)	2.046(2)	2.147(2)	2.112(4)
N(2)-Ir(1)	2.055(2)	2.146(2)	2.085(2)	2.104(4)
C(1)-Ir(1)	2.013(3)	2.019(3)	2.0121(3)	2.031(5)
C(2)-Ir(1)	2.045(3)	2.062(3)	2.015(3)	2.010(5)
$C_{(C≡NR)}$-Ir(1)	1.998(3)	1.990(3)	1.910(3)	2.015(5)
C≡N	1.162(3)	1.155(4)	1.156(4)	1.146(6)
Cl(1)-Ir(1)	2.480(7)	2.384(7)	2.446(7)	2.513(16)
**Angles (º)**				
C(1)-Ir(1)-N(2)	95.1(10)	173.7(9)	89.4(10)	90.4(19)
C(1)-Ir(1)-N(1)	80.4(10)	80.2(10)	79.7(11)	80.3(2)
C(1)-Ir(1)-C(2)	87.4(10)	95.1(11)	96.1(11)	90.7(2)
C(2)-Ir(1)-N(2)	80.0(10)	78.7(10)	80.0(11)	80.3(2)
C(2)-Ir(1)-N(1)	91.7(10)	88.2(10)	173.4(10)	93.0(2)
C(2)-Ir(1)-$C_{(C≡NR)}$	176.4(10)	172.1(10)	95.3(12)	90.4(2)
N(2)-Ir(1)-$C_{(C≡NR)}$	96.4(10)	94.3(10)	174.8(11)	83.1(19)
N(1)-Ir(1)-$C_{(C≡NR)}$	91.9(9)	96.5(10)	89.7(10)	106.5(2)
Ir(1)- $C_{(C≡NR)}$-$N_{(C≡NR)}$	178.6(2)	171.5(2)	176.0(3)	168.2(5)
$C_{(C≡NR)}$-$N_{(C≡NR)}$-C_{ipso}	173.1(3)	174.0(3)	171.4(3)	177.0(6)
N(1)-Ir-N(2)	170.7(9)	98.4(9)	94.8(9)	168.4(17)
C(1)-Ir- $C_{(C≡NR)}$	92.7(10)	92.0(11)	88.9(11)	173.1(2)
C(1)-Ir- $C_{(Cl)}$	175.3(7)	94.9(8)	172.6(9)	91.8(15)
C(2)-Ir- $C_{(Cl)}$	89.8(7)	89.2(7)	90.2(8)	170.8(17)

Table S4. Selected distances (Å) and angles (º) for complexes ***N,N-trans*-1a, *N,N-trans*-1c, *N,N-trans*-2a** and ***N,N-trans*-2c.**

Complexes	***N,N-trans*-1a**	***N,N-trans*-1c·0.6 $CHCl_3$**	***N,N-trans*-2a**	***N,N-trans*-2c**
**Distances (Å)**				
N(1)-Ir(1)	2.081(6)	2.048(3)	2.064(4)	2.060(5)
N(2)-Ir(1)	2.048(6)	2.063(3)	2.063(4)	2.078(4)
C(1)-Ir(1)	2.053(6)	2.045(3)	2.076(4)	2.056(6)
C(2)-Ir(1)	2.082(6)	2.059(3)	2.069(5)	2.055(5)
$C_{(C\equiv CR)}$-Ir(1)	2.058(6)	2.062(3)	2.079(5)	2.110(5)
$C_{(C\equiv NR)}$-Ir(1)	1.985(6)	1.983(3)	1.986(5)	1.983(5)
$C_\alpha \equiv C_\beta$	1.194(9)	1.213(5)	1.163(7)	1.147(8)
C≡N	1.163(8)	1.154(4)	1.169(6)	1.158(7)
**Angles (º)**				
C(1)-Ir(1)-N(2)	90.7(2)	92.7(12)	90.6(16)	89.7(2)
C(1)-Ir(1)-N(1)	79.6(2)	80.5(12)	79.2(16)	79.2(2)
C(1)-Ir(1)-C(2)	88.0(2)	90.0(12)	91.1(17)	87.4(2)
C(2)-Ir(1)-N(2)	80.3(2)	79.8(12)	79.2(18)	79.4(2)
C(2)-Ir(1)-N(1)	93.2(2)	95.8(12)	94.2(17)	92.5(19)
C(2)-Ir(1)-$C_{(C\equiv NR)}$	94.9(2)	94.5(12)	97.6(18)	90.7(2)
N(2)-Ir(1)-$C_{(C\equiv NR)}$	90.6(2)	87.4(12)	91.6(16)	89.0(18)
N(1)-Ir(1)-$C_{(C\equiv NR)}$	99.4(2)	99.7(12)	99.5(16)	101.9(19)
C(1)-Ir(1)-$C_{(C\equiv CR)}$	90.1(2)	84.7(12)	86.0(17)	91.6(2)
N(2)-Ir(1)-$C_{(C\equiv CR)}$	96.8(2)	96.6(12)	102.5(16)	100.4(18)
N(1)-Ir(1)-$C_{(C\equiv CR)}$	89.4(2)	87.2(12)	83.6(16)	87.5(18)
$C_{(C\equiv NR)}$-Ir(1)-$C_{(C\equiv CR)}$	87.0(2)	90.8(2)	85.3(18)	90.2(2)
Ir(1)- $C_{(C\equiv NR)}$-$N_{(C\equiv NR)}$	174.0(5)	169.5(3)	171.6(4)	174.6(4)
Ir(1)- $C_{(C\equiv CR)}$-$C_{(C\equiv CR)}$	174.2(5)	172.5(5)	166.6(4)	177.1(5)
$C_{(C\equiv NR)}$-$N_{(C\equiv NR)}$-C_{ipso}	170.9(7)	168.6(6)	170.5(5)	170.2(5)
$C_{(C\equiv CR)}$-$C_{(C\equiv CR)}$-C_{ipso}	177.7(7)	172.6(3)	175.1(5)	174.3(8)
N(1)-Ir-N(2)	168.6(2)	171.9(11)	167.8(14)	166.6(19)
C(1)-Ir- $C_{(C\equiv NR)}$	177.0(2)	175.4(12)	171.3(18)	177.9(19)
C(2)-Ir- $C_{(C\equiv CR)}$	176.5(2)	173.4(13)	176.7(17)	179.0(2)

Table S5. Selected distances (Å) and angles (º) for complexes ***N,N-cis-*(*OC*-6-32)-1a** and ***N,N-cis-*(*OC*-6-32)-1b.**

**Complexes**	***N,N-cis-*(*OC*-6-32)-1a·1.75 CH_2Cl_2**	***N,N-cis-*(*OC*-6-32)-1b·0.9 CH_2Cl_2**
**Distances (Å)**		
N(1)-Ir(1)	2.156(5)	2.149(7)
N(2)-Ir(1)	2.090(5)	2.098(7)
C(1)-Ir(1)	2.064 (6)	2.079(8)
C(2)-Ir(1)	2.013(6)	2.007(9)
$C_{(C\equiv CR)}$-Ir(1)	2.061(6)	2.107(8)
$C_{(C\equiv NR)}$-Ir(1)	1.903(6)	1.900(10)
$C_{\alpha}\equiv C_{\beta}$	1.209(9)	1.110(12)
C≡N	1.163(8)	1.150(12)
**Angles (°)**		
C(1)-Ir(1)-N(2)	89.4(2)	88.7(3)
C(1)-Ir(1)-N(1)	78.7(2)	79.3(3)
C(1)-Ir(1)-C(12)	95.9(2)	95.5(3)
C(12)-Ir(1)-N(2)	79.8(2)	79.5(3)
C(12)-Ir(1)-N(1)	173.7(2)	172.0(3)
C(12)-Ir(1)-$C_{(C\equiv NR)}$	94.8(2)	96.4(3)
N(2)-Ir(1)-$C_{(C\equiv NR)}$	174.4(2)	175.5(3)
N(1)-Ir(1)-$C_{(C\equiv NR)}$	88.7(2)	89.7(3)
C(1)-Ir(1)-$C_{(C\equiv CR)}$	170.1(2)	172.6(3)
N(2)-Ir(1)-$C_{(C\equiv CR)}$	86.9(2)	89.0(3)
N(1)-Ir(1)-$C_{(C\equiv CR)}$	92.6(2)	93.9(3)
$C_{(C\equiv NR)}$-Ir(1)-$C_{(C\equiv CR)}$	91.7(2)	93.1(3)
Ir(1)- $C_{(C\equiv NR)}$-$N_{(C\equiv NR)}$	179.1(5)	176.9(8)
Ir(1)- $C_{(C\equiv CR)}$-$C_{(C\equiv CR)}$	173.0(5)	177.2(7)
$C_{(C\equiv NR)}$-$N_{(C\equiv NR)}$- C_{ipso}	166.8(6)	171.3(10)
$C_{(C\equiv CR)}$-$C_{(C\equiv CR)}$- C_{ipso}	177.7(7)	178.0(10)
N(1)-Ir-N(2)	96.8(19)	94.2(3)
C(1)-Ir- $C_{(C\equiv NR)}$	92.8(2)	89.7(3)
C(12)-Ir- $C_{(C\equiv CR)}$	92.5(2)	91.0(3)

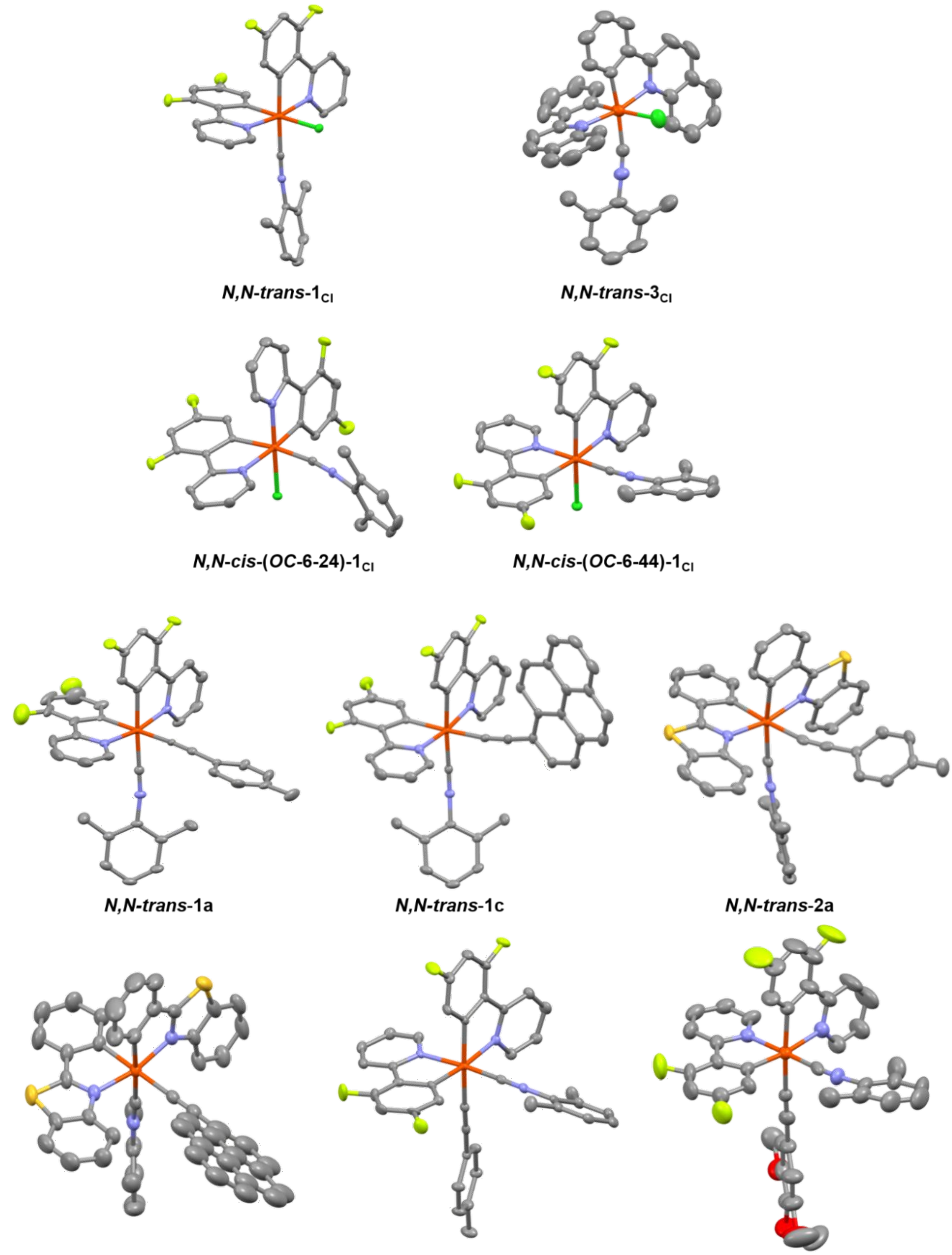

Figure S25. Crystal structures of ***N,N-trans*-1**$_{Cl}$·CH_2Cl_2, ***N,N-trans*-3**$_{Cl}$·$CHCl_3$, ***N,N-cis*-(*OC*-6-24)-1**$_{Cl}$, ***N,N-cis*-(*OC*-6-44)-1**$_{Cl}$, ***N,N-trans*-1a**, ***N,N-trans*-1c**·0.6$CHCl_3$, ***N,N-trans*-2a**, ***N,N-trans*-2c**, ***N,N-cis*-1a**·1.75CH_2Cl_2 and ***N,N-cis*-1b**·0.9CH_2Cl_2 with thermal ellipsoids drawn at 50% probability level.

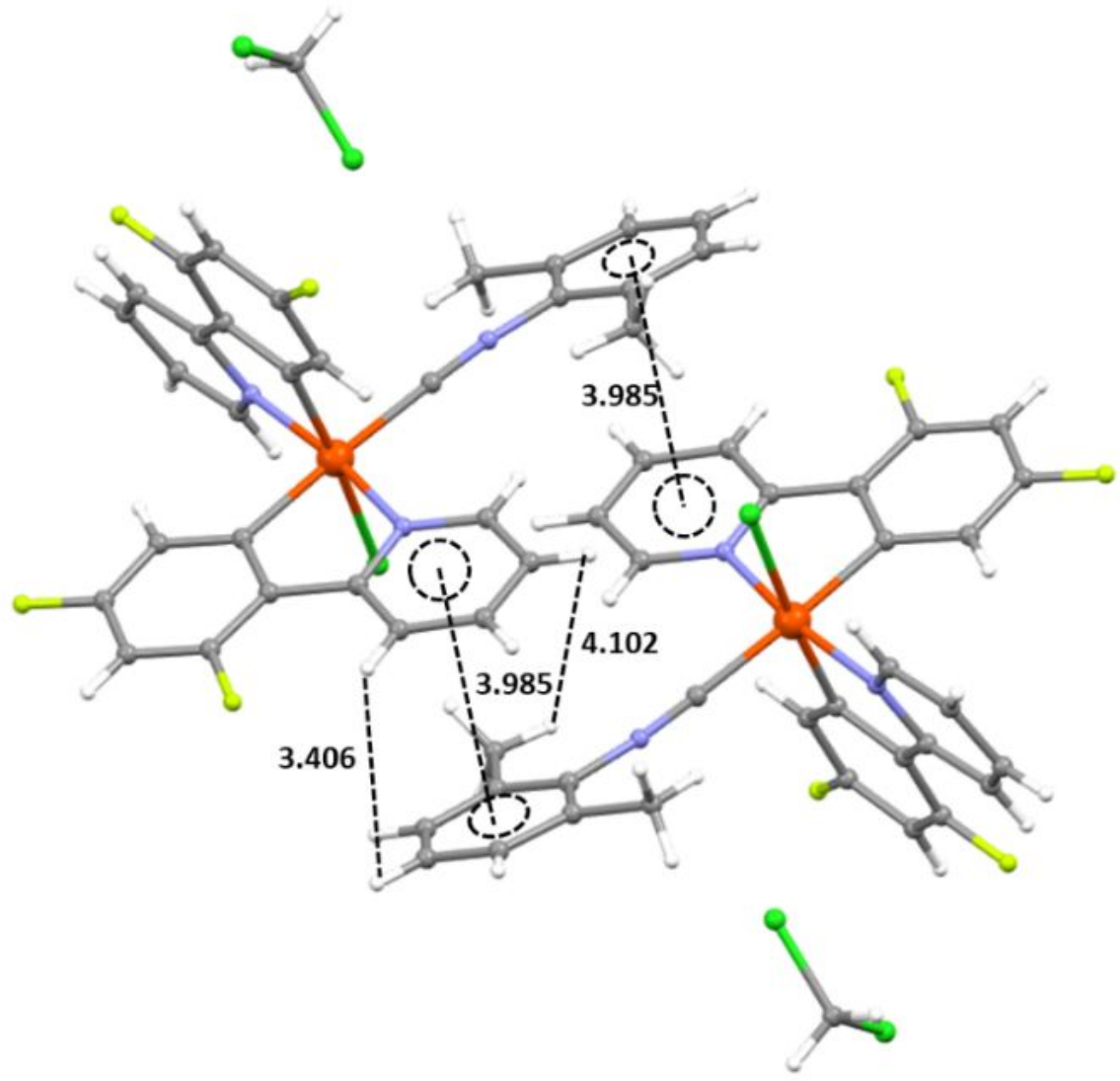

Figure S26. Crystal packing of complex ***N,N-trans*-1**$_{\mathbf{Cl}}$ with intermolecular contacts

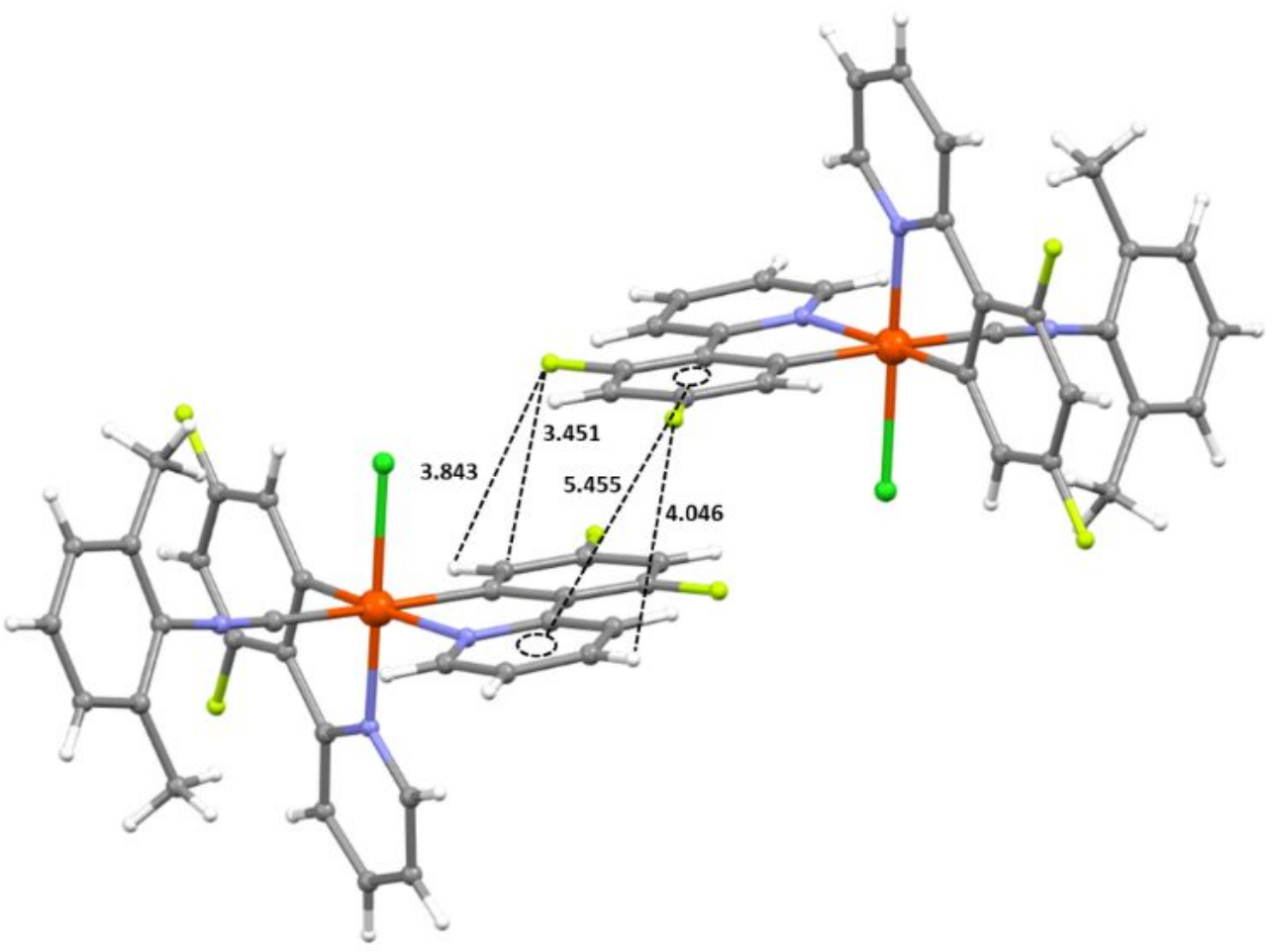

Figure S27. Crystal packing of complex ***N,N-cis*-(*OC*-6-24)-1**$_{\mathbf{Cl}}$ with intermolecular contacts

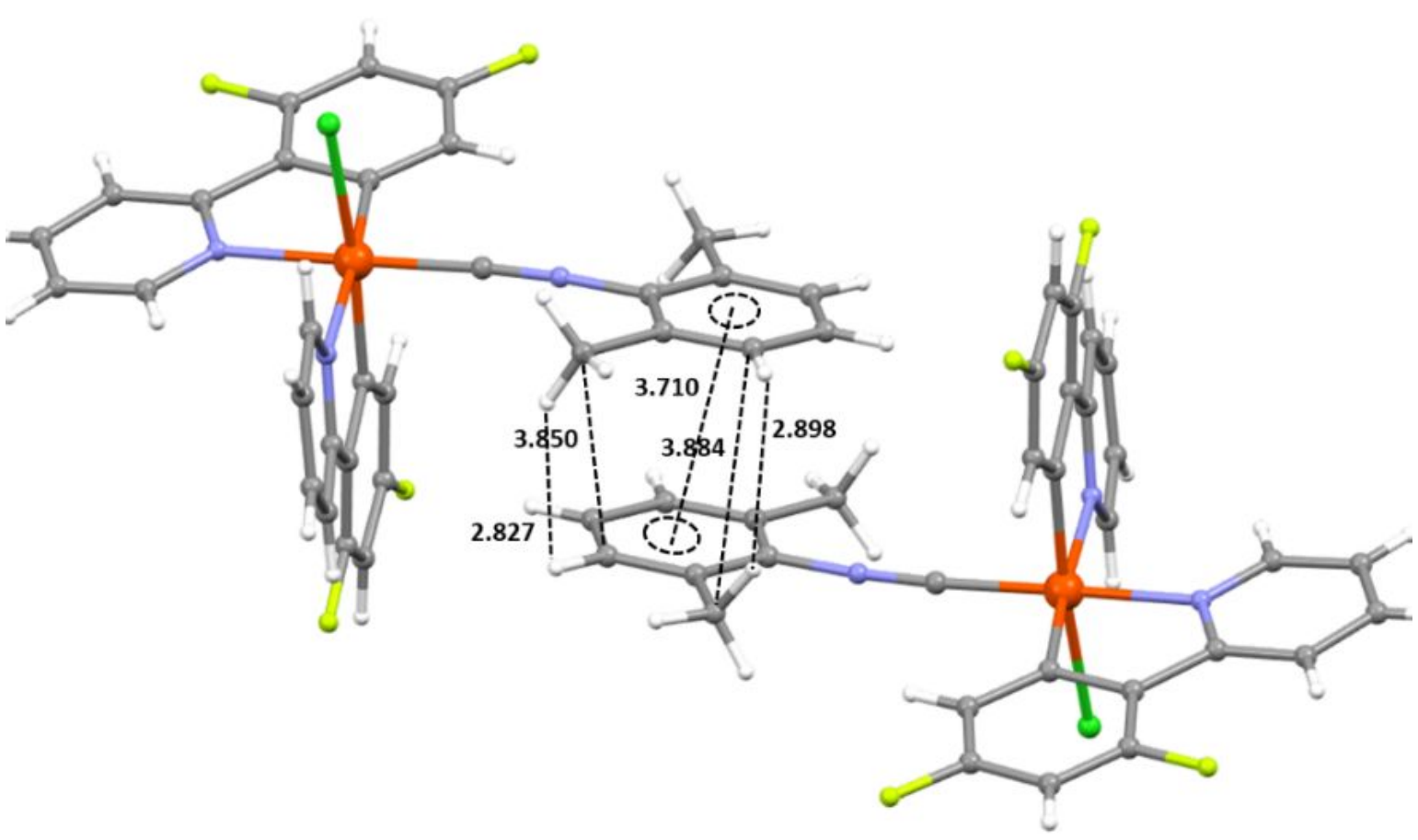

Figure S28. Crystal packing of complex ***N,N-cis*-(*OC*-6-44)-1**$_{\mathbf{Cl}}$ with intermolecular contacts

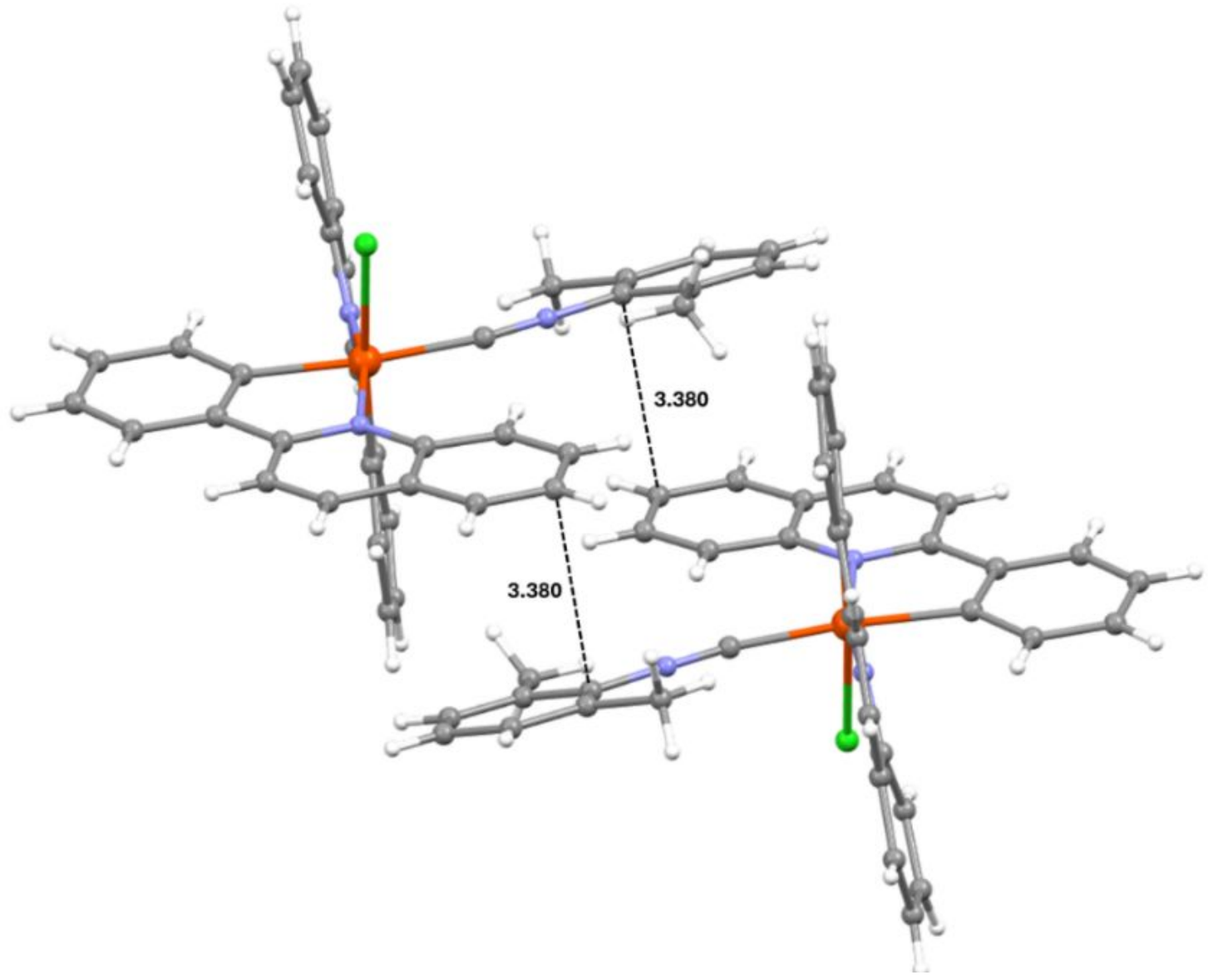

Figure S29. Crystal packing of complex ***N,N-trans*-3**$_{\mathbf{Cl}}$ with intermolecular contacts

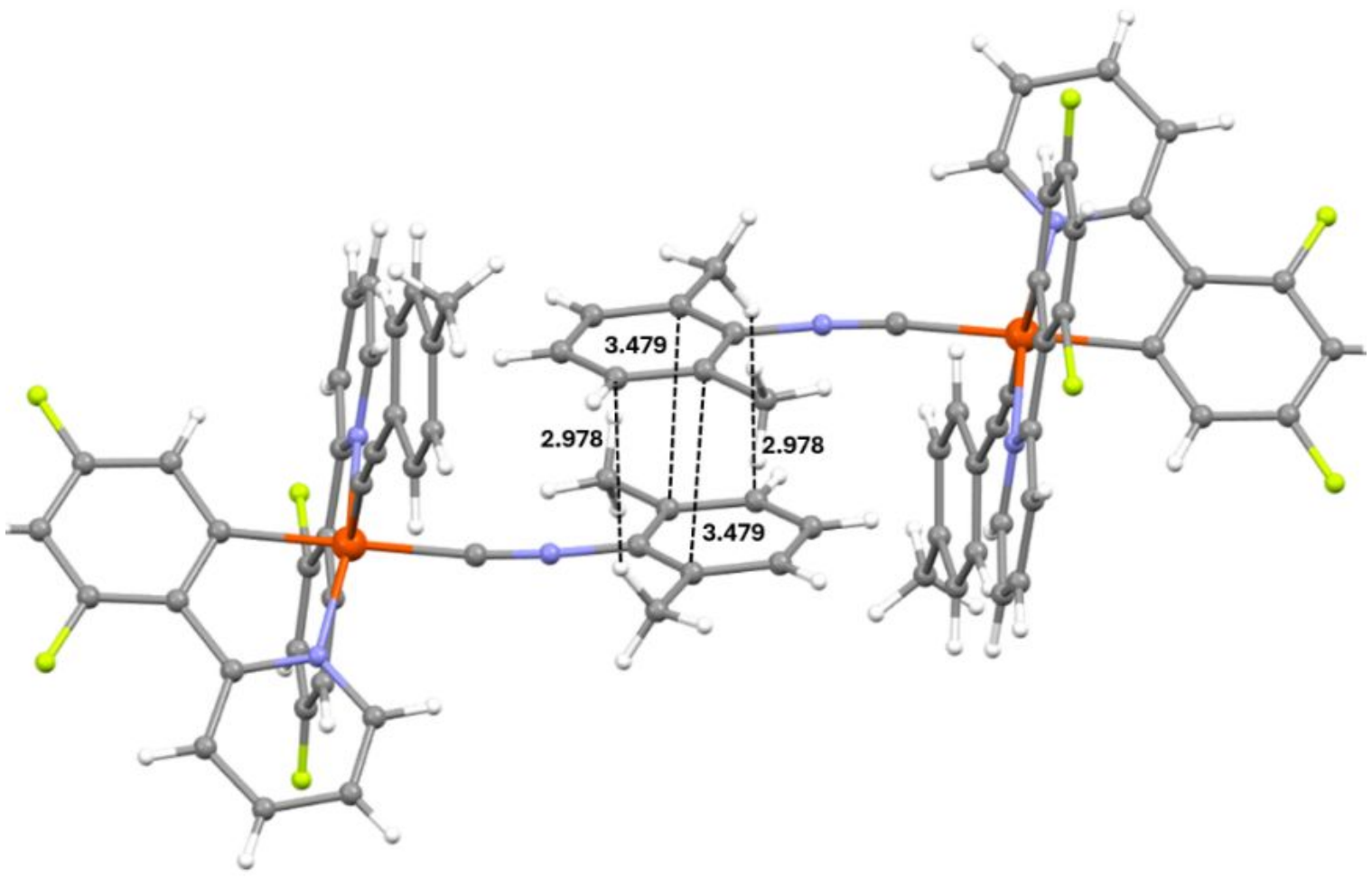

Figure S30. Crystal packing of complex ***N,N-trans*-1a** with intermolecular contacts

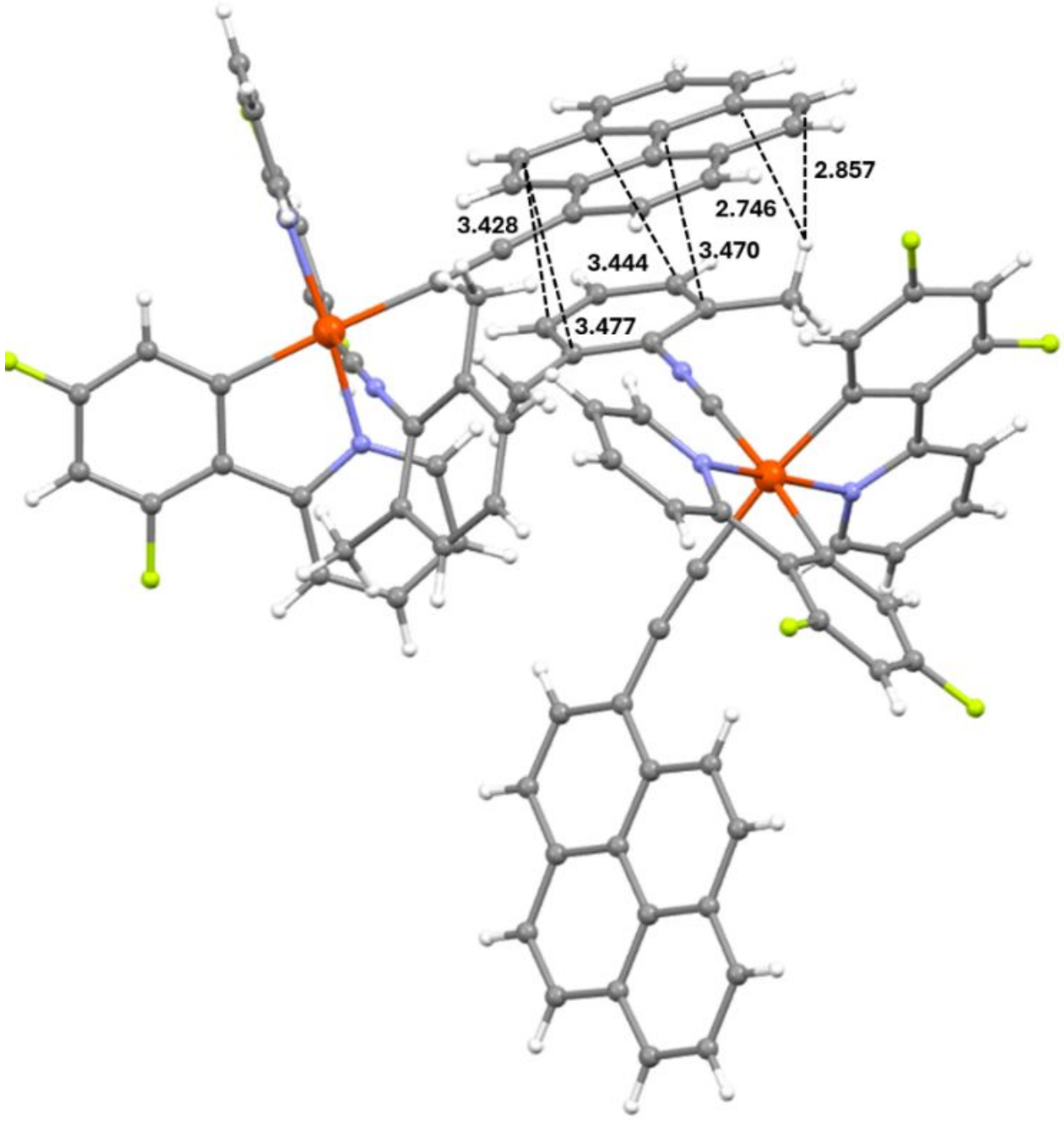

Figure S31. Crystal packing of complex ***N,N-trans*-1c** with intermolecular contacts

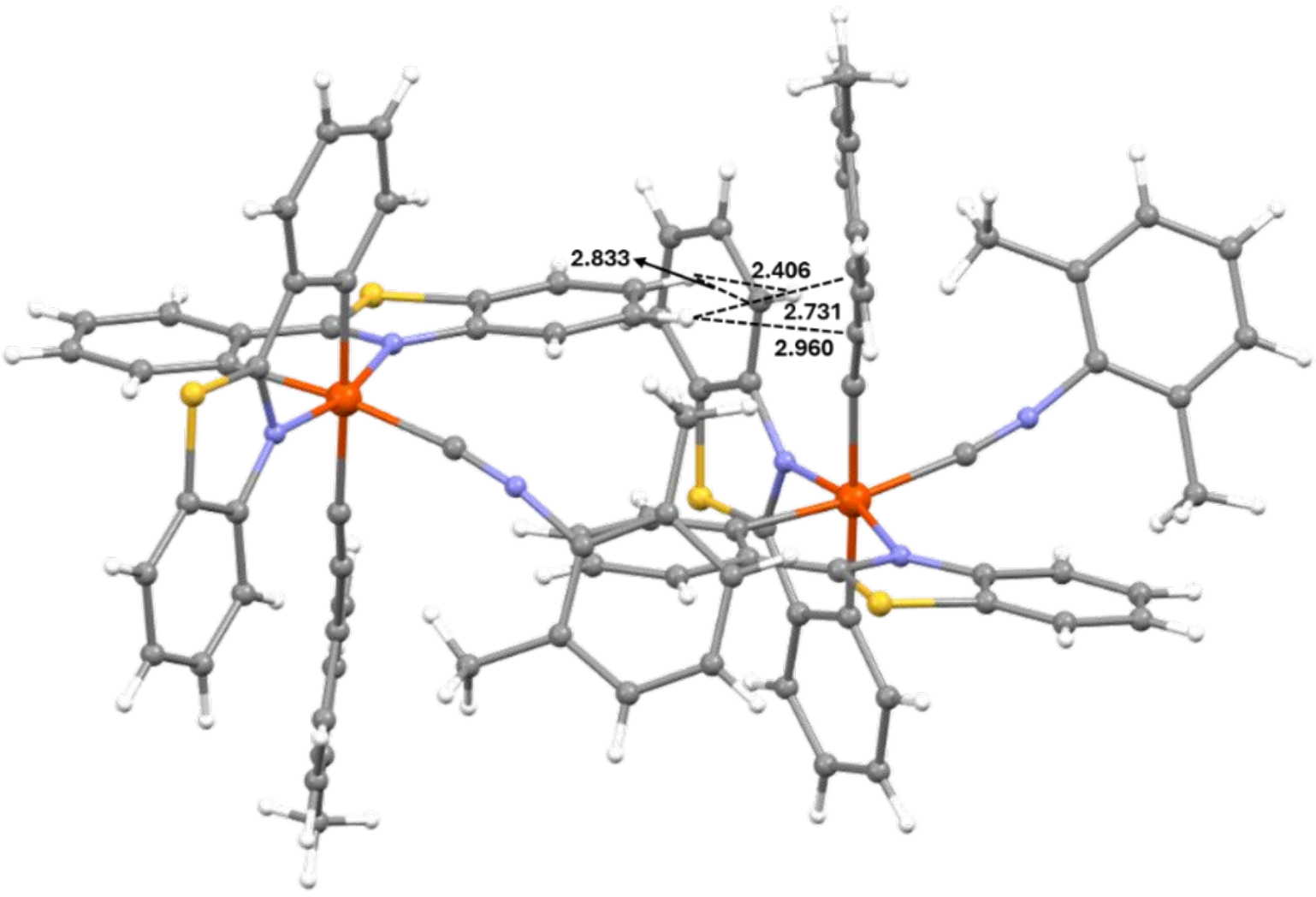

Figure S32. Crystal packing of complex ***N,N-trans*-2a** with intermolecular contacts

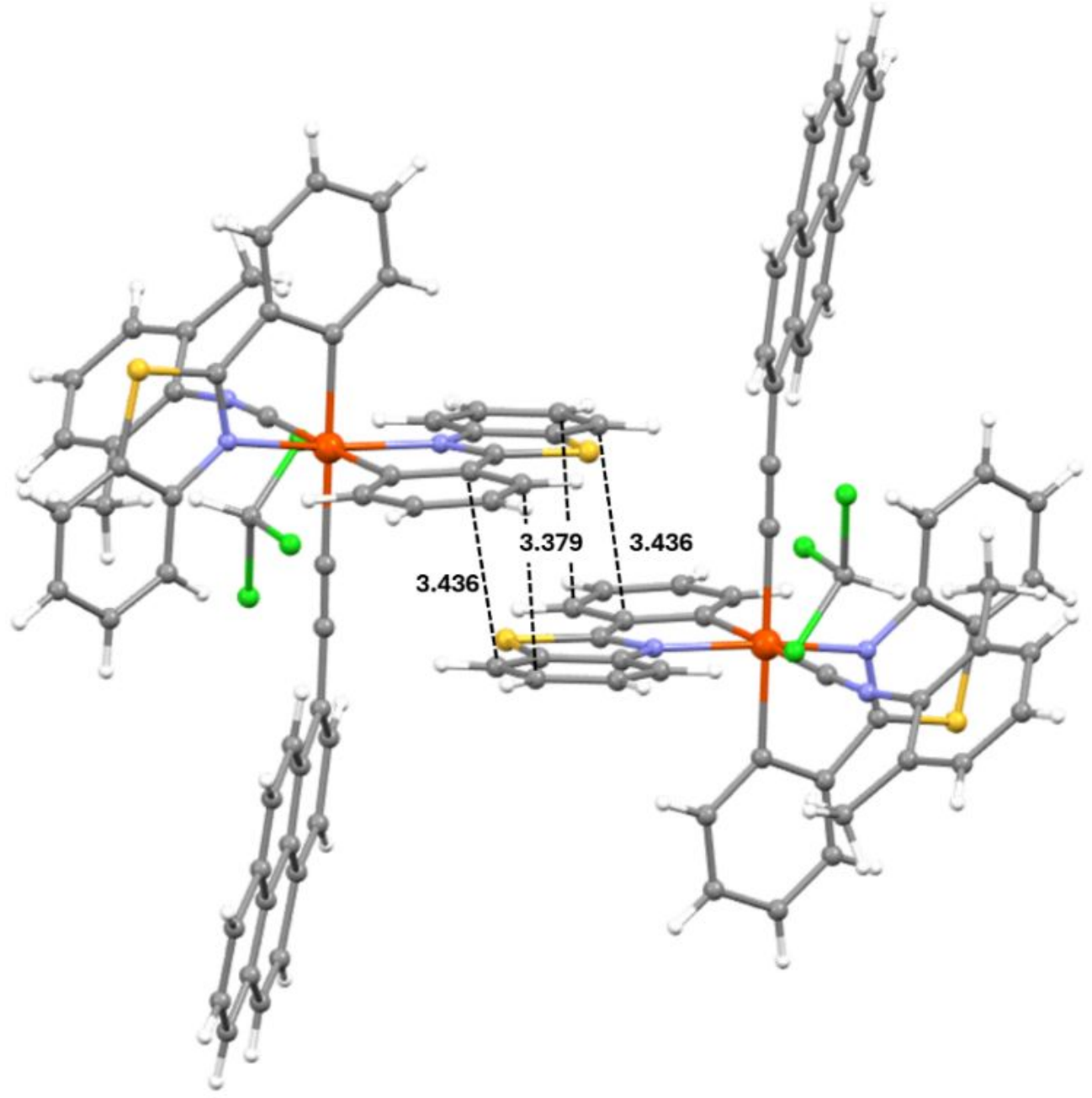

Figure S33. Crystal packing of complex ***N,N-trans*-2c** with intermolecular contacts

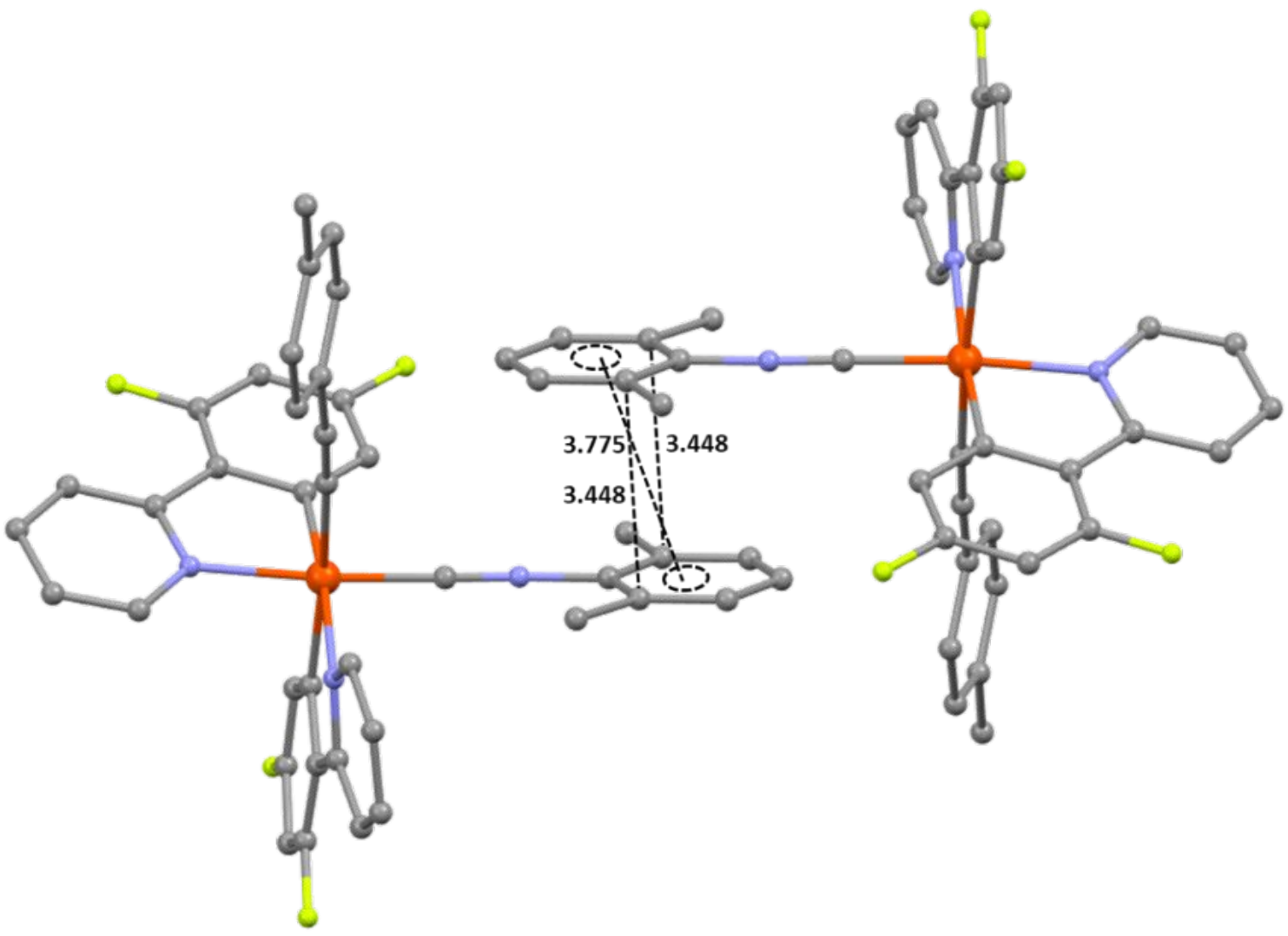

Figure S34. Crystal packing of complex ***N,N-cis*-(*OC*-6-32)-1a** with intermolecular contacts

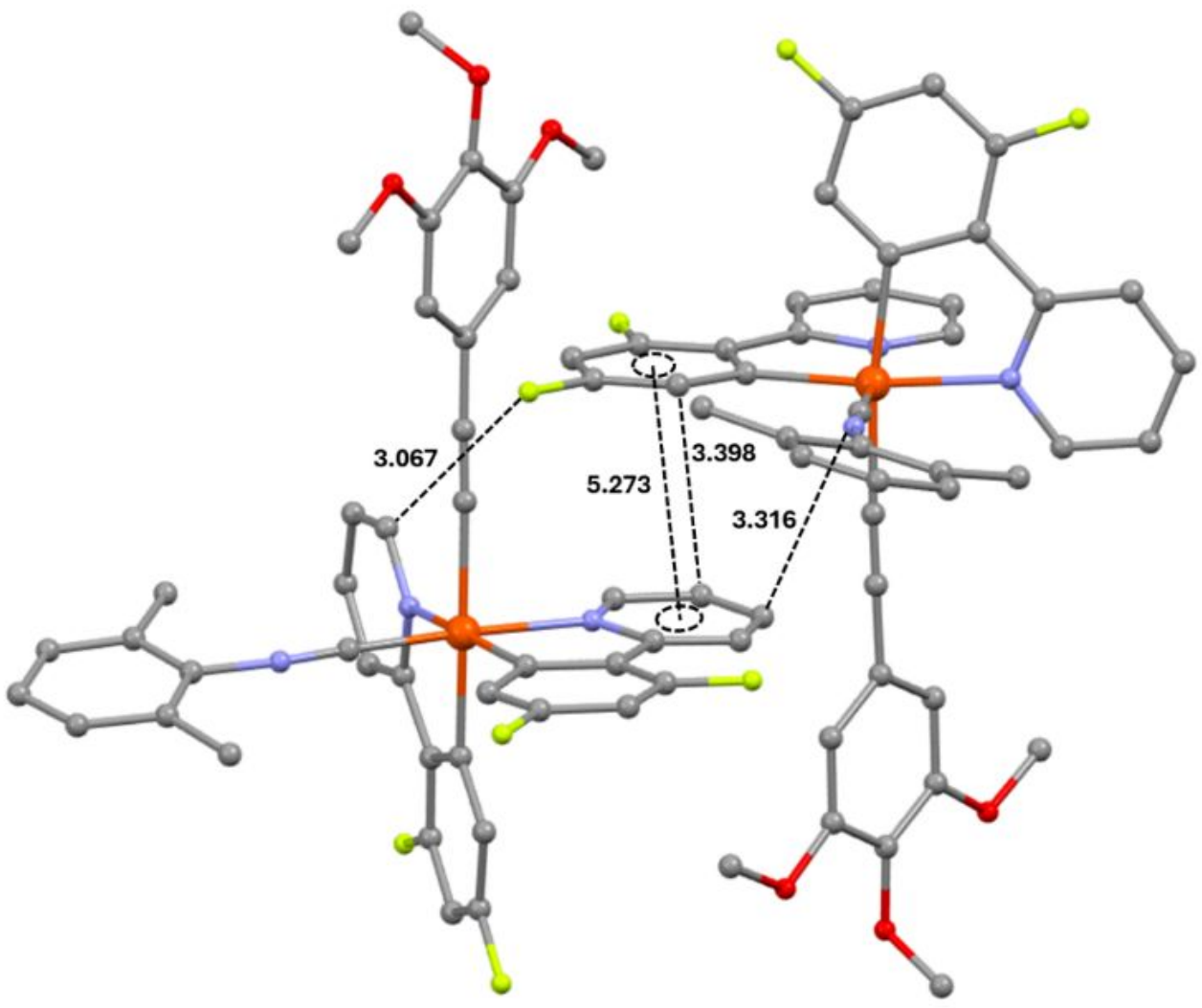

Figure S35. Crystal packing of complex ***N,N-cis*-(*OC*-6-32)-1b** with intermolecular contacts

4. Photophysical Properties and Theoretical calculations

Table S6. UV-Vis absorption data in CH_2Cl_2 solution (5 x 10^{-5} M)

Complex	λ /nm (ε x 10^{-3} / $mol^{-1}\cdot L\cdot cm^{-1}$)
***N,N-trans*-1$_{Cl}$** [Ir(dffpy)$_2$(CNXyl)Cl]	260 (46.7), 310 (19.3), 360 (5.5), 380$_{sh}$ tail to 420
***N,N-cis*-(*OC*-6-24)-1$_{Cl}$** [Ir(dffpy)$_2$(CNXyl)Cl]	260 (20.8), 276 (19.2), 310 (10.5), 360 (2.6), tail to 400
***N,N-cis*-(*OC*-6-44)-1$_{Cl}$** [Ir(dffpy)$_2$(CNXyl)Cl]	260 (24.5), 291 (13.4), 310 (10.4), 360 (3.5), 380$_{sh}$ tail to 420
***C,C-trans*-1$_{Cl}$** [Ir(dffpy)$_2$(CNXyl)Cl] a)	260 (22.3), 274 (8.3), 310 (10.2), 340 (4.9) tail to 400
***N,N-trans*-1a** [Ir(dffpy)$_2$(CNXyl)(C≡C-Tol)]	261(62.2), 278 (48.8), 312 (20.2), 375 (3.7), 385 (2.4), tail to 420
***N,N-cis*-(*OC*-6-32)-1a** [Ir(dffpy)$_2$(CNXyl)(C≡CTol)]	261 (43.4), 278 (37.2), 312 (14.3), 375 (2.9), 380 (2.5), tail to 420
***C,C-trans*-1a** [Ir(dffpy)$_2$(CNXyl)(C≡C-Tol)] a)	260 (20.8), 274 (20.4), 310 (11.1), 365 (2.7) tail to 420
***N,N-trans*-1b** [Ir(dffpy)$_2$(CNXyl)(C≡C-$C_6H_2(OMe)_3$)]	261 (53.6), 286 (42.0), 324 (14.7), 347 (9.5), 375 (3.1), tail to 420
***N,N-cis*-(*OC*-6-32)-1b** [Ir(dffpy)$_2$(CNXyl)(C≡C-$C_6H_2(OMe)_3$)]	261 (18.3), 286 (15.0), 310 (7.9), 347 (2.8), 375 (1.2), tail to 400
***C,C-trans*-1b** [Ir(dffpy)$_2$(CNXyl)(C≡C-$C_6H_2(OMe)_3$)] a)	260 (31.9), 266 (34.8), 275 (30.8), 320 (13.7), 343 (8.2), 365 (2.9) tail to 420
***N,N-trans*-1c** [Ir(dffpy)$_2$(CNXyl)(C≡C-pyrene)]	262 (67.4), 292 (55.3), 349 (20.9), 368 (34.8), 387 (41.7), 400 (33.5), tail to 425
***N,N-trans*-2$_{Cl}$** [Ir(pbt)$_2$(CNXyl)Cl]	275 (38.0), 318 (41.2), 371 (11.3), 400 (8.4), 420 (6.8), tail to 460
***N,N-trans*-2a** [Ir(pbt)$_2$(CNXyl)(C≡C-Tol)]	275 (51.7), 316 (28.7), 378 (6.5), 420 (2.5), tail to 460
***N,N-trans*-2b** [Ir(pbt)$_2$(CNXyl)(C≡C-$C_6H_2(OMe)_3$)]	275 (80.0), 287 (65.2), 318 (45.1), 347 (23.4), 380 (9.9), 425 (4.9), tail to 460
***N,N-trans*-2c** [Ir(pbt)$_2$(CNXyl)(C≡C-pyrene)]	295 (54.9), 318 (40.7), 368 (35.8), 387 (44.8), 401 (34.8), tail to 460
***N,N-trans*-3$_{Cl}$** [Ir(pq)$_2$(CNXyl)Cl]	278 (40.9), 327 (15.1), 347 (12.6), 430 (3.2), tail to 480
***N,N-trans*-3a** [Ir(pq)$_2$(CNXyl)(C≡C-Tol)]	277 (72.2), 286 (58.9), 331 (21.9), 355 (16.9), 412 (5.3), 430 (4.8), tail to 500
***N,N-trans*-3b** [Ir(pq)$_2$(CNXyl)(C≡C-$C_6H_2(OMe)_3$)]	277 (73.1), 286 (59.5), 330 (22.2), 354 (17.1), 412 (5.2), 450 (3.1), tail to 500
***N,N-trans*-3c** [Ir(pq)$_2$(CNXyl)(C≡C-pyrene)]	278 (67.3), 296 (45.0), 351 (26.6), 370 (31.4), 387 (35.4), 403 (29.8), tail to 500

a) Data in CH_3CN solution (5 x 10^{-5} M)

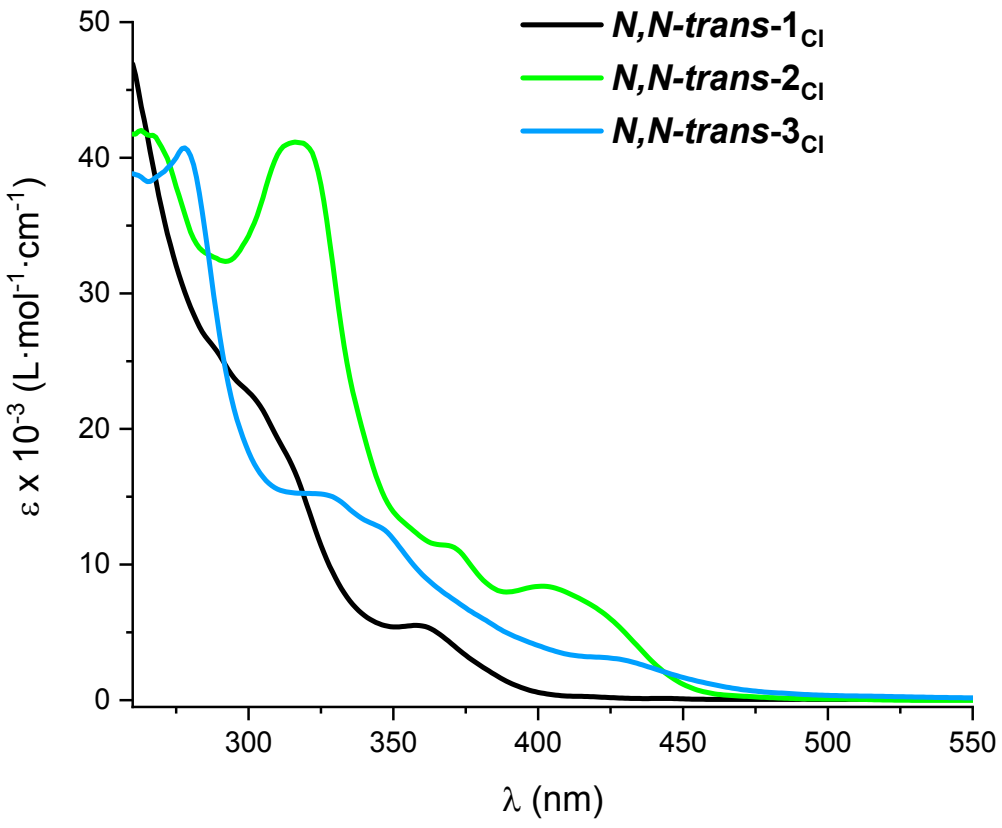

Figure S36. UV-Vis absorption spectra for the series a) ***N,N-trans*-1$_{Cl}$, 2$_{Cl}$-3$_{Cl}$**

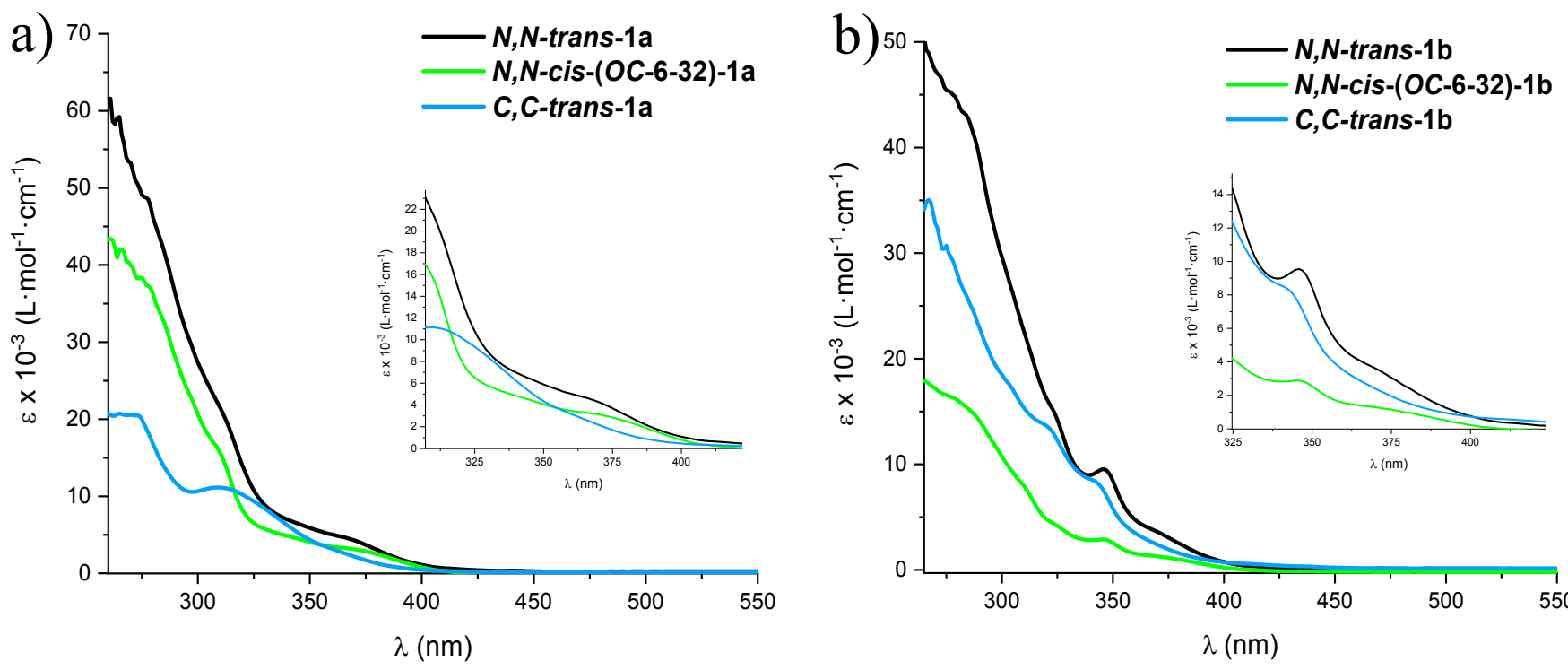

Figure S37. UV-Vis absorption spectra in CH_2Cl_2 solution (5 x 10^{-5} M) for a) ***N,N-trans*-1a**, ***N,N-cis*-(*OC-6*-43)-1a**, ***N,N-cis*-(*OC-6*-32)-1a** and ***C,C-trans*-1a** and b) ***N,N-trans*-1b, *N,N-cis*-(*OC-6*-32)-1b** and ***C,C-trans*-1b** (The spectra represented in blue were measured in CH_3CN 5 x 10^{-5} M solution).

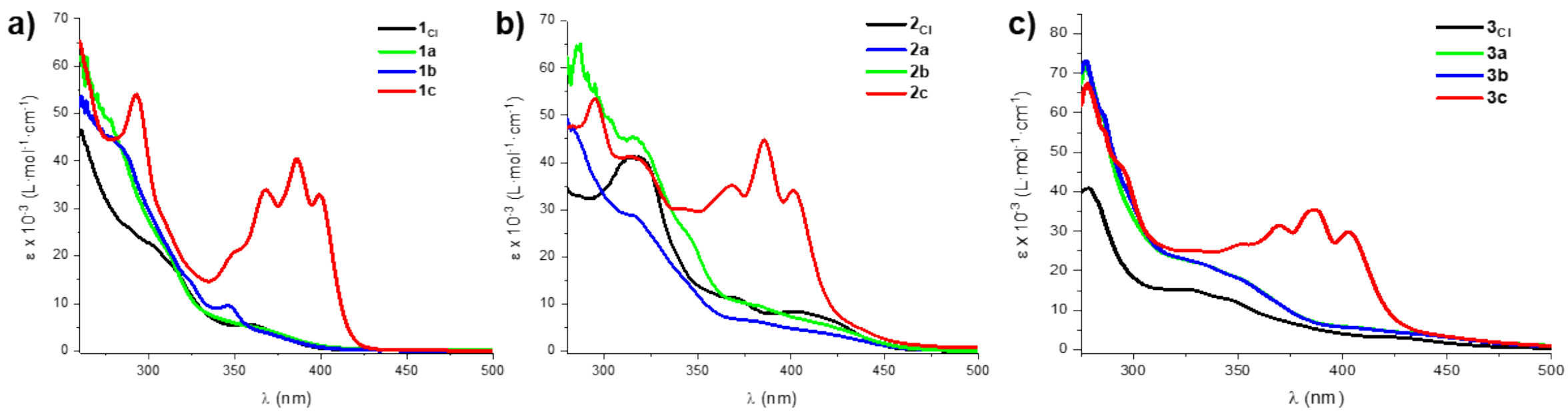

Figure S38. UV-Vis absorption spectra for series of complex a) **1$_{Cl}$, 1a-1c**, b) **2$_{Cl}$, 2a-2c** and c) **3$_{Cl}$, 3a-3c** in CH_2Cl_2 solution (5 x 10^{-5} M) at 298 K.

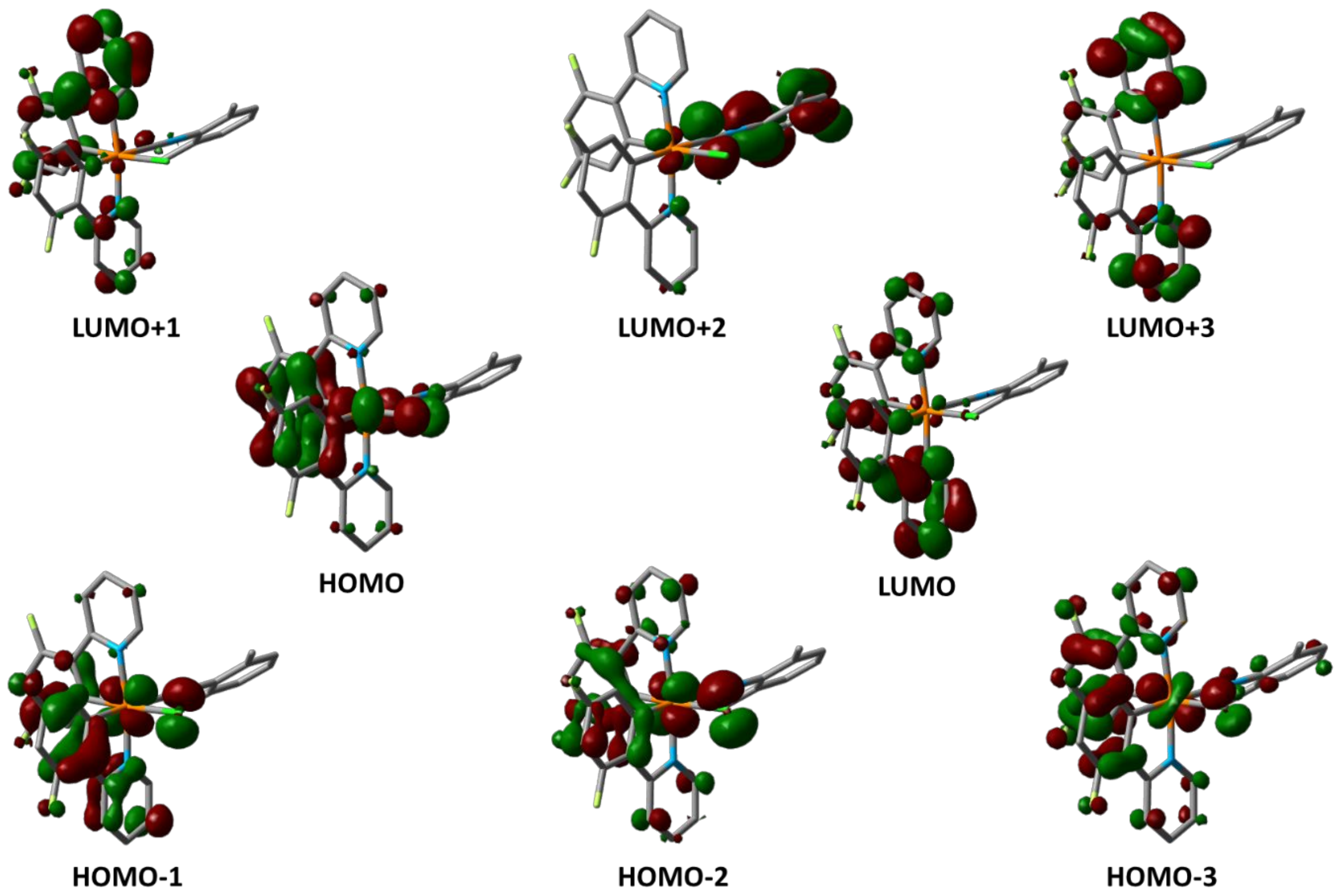

Figure S39. Selected frontier Molecular Orbitals for ***N,N-trans*-1**$_{Cl}$ in CH_2Cl_2 in the ground state.

Table S7. Composition (%) of frontier MOs in terms of ligands and metals in the ground state for ***N,N-trans*-1**$_{Cl}$ in CH_2Cl_2.

***N,N-trans*-1**$_{Cl}$						
**Orbital**	**Energy (eV)**	**Ir**	**dfppy(1)**	**dfppy(2)**	**CNXyl**	**Cl**
**LUMO+5**	-0.40	24	8	36	29	4
**LUMO+4**	-0.97	2	37	59	2	0
**LUMO+3**	-1.07	2	60	37	1	0
**LUMO+2**	-1.28	5	2	6	87	0
**LUMO+1**	-1.65	3	71	21	5	0
**LUMO**	-1.69	4	24	69	2	1
**HOMO**	-5.87	34	29	24	4	9
**HOMO-1**	-6.21	22	15	49	0	14
**HOMO-2**	-6.36	25	28	24	2	22
**HOMO-3**	-6.41	15	39	29	9	8
**HOMO-4**	-6.58	29	19	9	39	4
**HOMO-5**	-6.62	1	19	54	1	25

Table S8. Selected vertical excitation energies singlets (S_0) and first triplets computed by TD-DFT/SCRF (CH_2Cl_2) with the orbitals involved of complex ***N,N-trans*-1**$_{Cl}$.

Complex	State	λ/nm	f	Transition (% Contribution)	
***N,N-trans*-1**$_{Cl}$	**T$_1$**	425.1	-	H-1→LUMO (31%), HOMO→LUMO (29%)	ILCT/ MLCT/XLCT
	**T$_2$**	417.7	-	H-3→L+1 (13%), H-2→L+1 (11%), HOMO→L+1 (29%)	ILCT/ MLCT/XLCT
	**S$_1$**	360.7	0.0338	HOMO→LUMO (93%)	ILCT/ MLCT/XLCT
	**S$_2$**	353.9	0.0189	HOMO→L+1 (94%)	ILCT/ MLCT/XLCT
	**S$_3$**	326.2	0.0470	H-2→LUMO (20%), H-1→LUMO (70%)	ILCT/ MLCT/XLCT
	**S$_4$**	324.0	0.0237	H-2→L+1 (18%), H-1→L+1 (72%)	ILCT/ MLCT/XLCT
	**S$_5$**	317.0	0.0273	HOMO→L+2 (94%)	ML”CT/LL”CT/

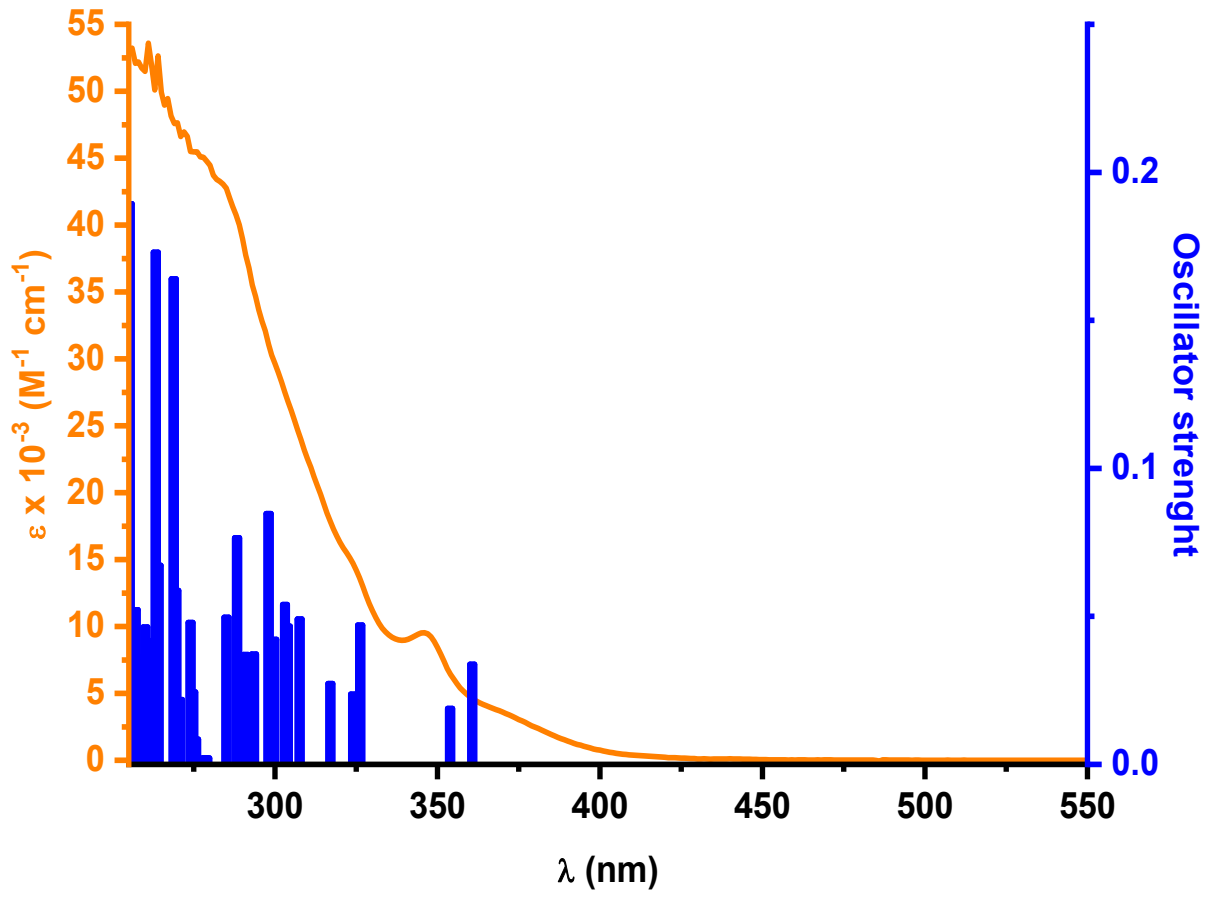

Figure S40. UV-Vis absorption experimental spectra (orange) and calculated transitions (blue) in CH_2Cl_2 ***N,N-trans*-1**$_{Cl}$.

Table S9. Plots and composition (%) of the frontier MOs and spin density of the first triplet state in CH_2Cl_2 of complex ***N,N-trans*-1**$_{Cl}$.

***N,N-trans*-1**$_{Cl}$		
SOMO	SOMO-1	Spin density
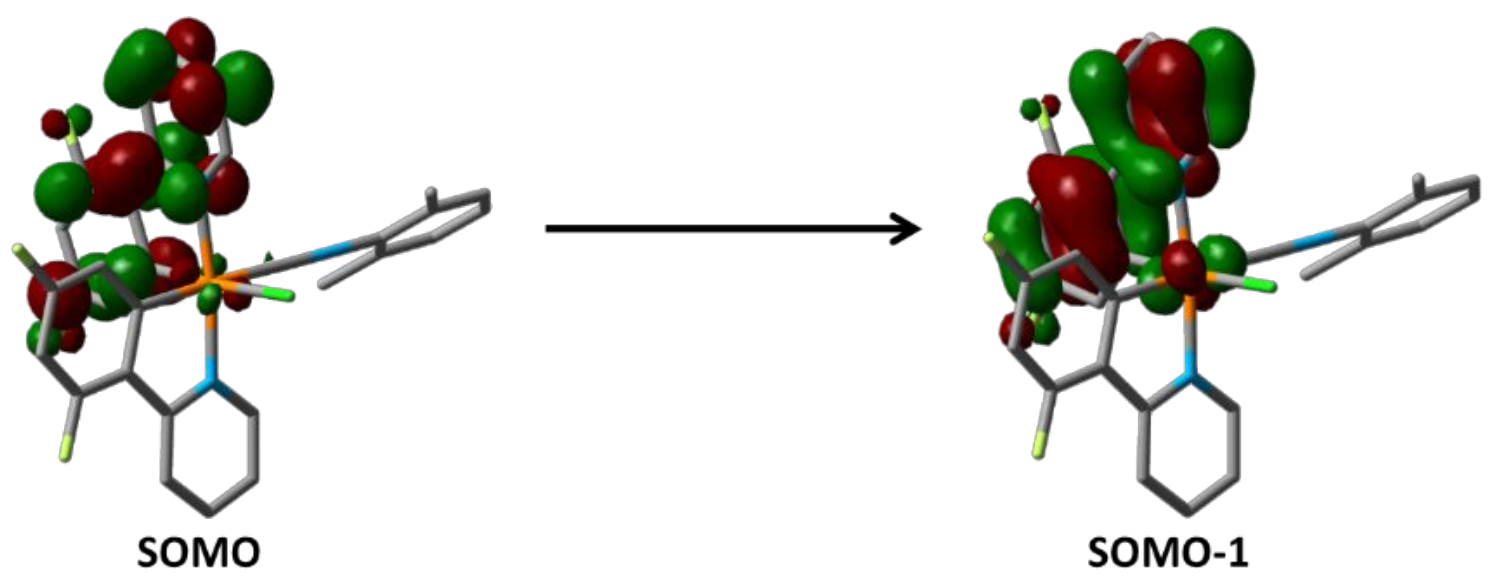		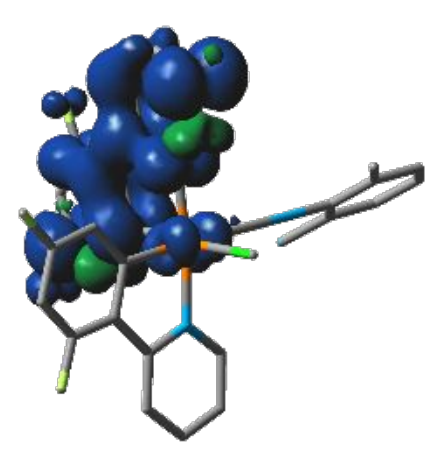
-3.34 eV<br>Ir 3 %, dfppy(1) 96 %, dfppy(2) 1 %, CNXyl 1 %, Cl 0 %	-4.47 eV<br>Ir 9 %, dfppy (1) 88 %, dfppy (2) 1 %, CNXyl 1 %, Cl 1 %	0.0556 on Ir

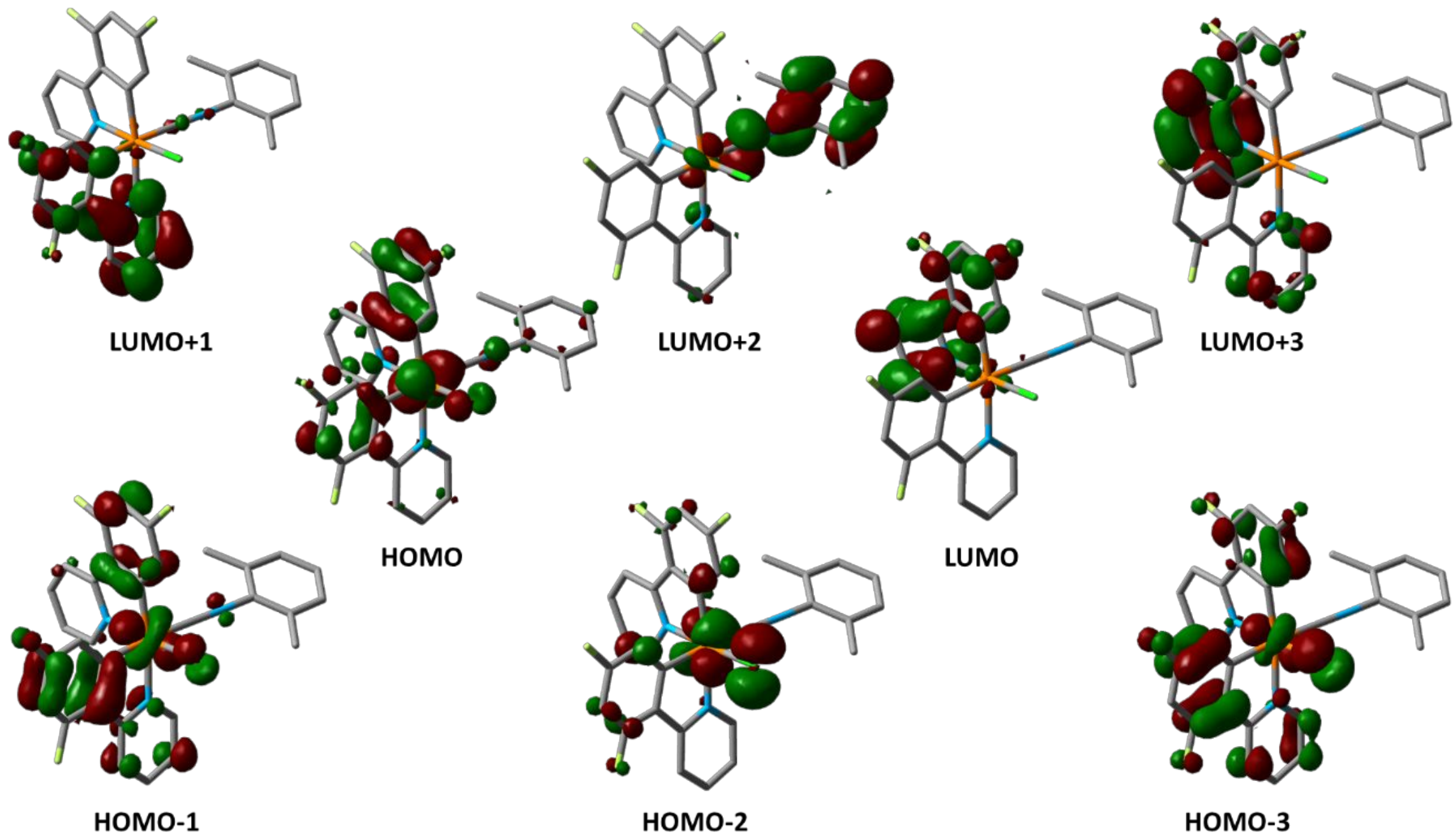

Figure S41. Selected frontier Molecular Orbitals for ***N,N-cis*-(*OC*-6-24)-1**$_{Cl}$ in the ground state in CH_2Cl_2.

Table S10. Composition (%) of frontier MOs in terms of ligands and metals in the ground state for ***N,N-cis*-(*OC*-6-24)-1**$_{Cl}$ in CH_2Cl_2.

***N,N-cis*-(*OC*-6-24)-1**$_{Cl}$						
**Orbital**	**Energy (eV)**	**Ir**	**dfppy(1)**	**dfppy(2)**	**CNXyl**	**Cl**
**LUMO+5**	-0.41	50	19	10	7	14
**LUMO+4**	-0.90	2	22	76	0	0
**LUMO+3**	-1.13	2	75	21	2	0
**LUMO+2**	-1.25	5	1	7	86	0
**LUMO+1**	-1.58	3	1	91	5	0
**LUMO**	-1.68	3	95	1	1	0
**HOMO**	-5.92	34	31	21	10	5
**HOMO-1**	-6.10	26	26	39	3	6
**HOMO-2**	-6.25	45	12	16	1	27
**HOMO-3**	-6.31	15	20	49	1	15
**HOMO-4**	-6.43	6	69	16	5	3
**HOMO-5**	-6.68	4	2	46	3	45

Table S11. Selected vertical excitation energies singlets (S_0) and first triplets computed by TD-DFT/SCRF (CH_2Cl_2) with the orbitals involved of complex ***N,N-cis-*(*OC*-6-24)-1**$_{Cl}$.

Complex	State	λ/nm	f	Transition (% Contribution)	
***N,N-cis-*(*OC*-6-24)-1**$_{Cl}$	**T_1**	422.3	-	H-4→LUMO (18%), HOMO→LUMO (51%)	ILCT/MLCT/ L”LCT
	**T_2**	415.5	-	H-3→L+1 (17%), H-1→L+1 (37%), HOMO→L+1 (21%)	ILCT/MLCT/ XLCT/ L”LCT
	**S_1**	354.2	0.0183	HOMO→LUMO (96%)	ILCT/MLCT/ L”LCT
	**S_2**	340.9	0.0247	HOMO→L+1 (91%)	ILCT/MLCT/ L”LCT
	**S_3**	333.5	0.0189	H-2→LUMO (25%), H-1→LUMO (62%)	ILCT/MLCT/ XLCT
	**S_4**	324.9	0.0352	H-1→L+1 (75%)	ILCT/MLCT
	**S_5**	323.9	0.0592	H-2→LUMO (67%), H-1→LUMO (26%)	ILCT/MLCT/ XLCT
	**S_6**	314.8	0.0685	H-2→L+1 (76%), H-1→L+1 (11%)	ILCT/MLCT /XLCT

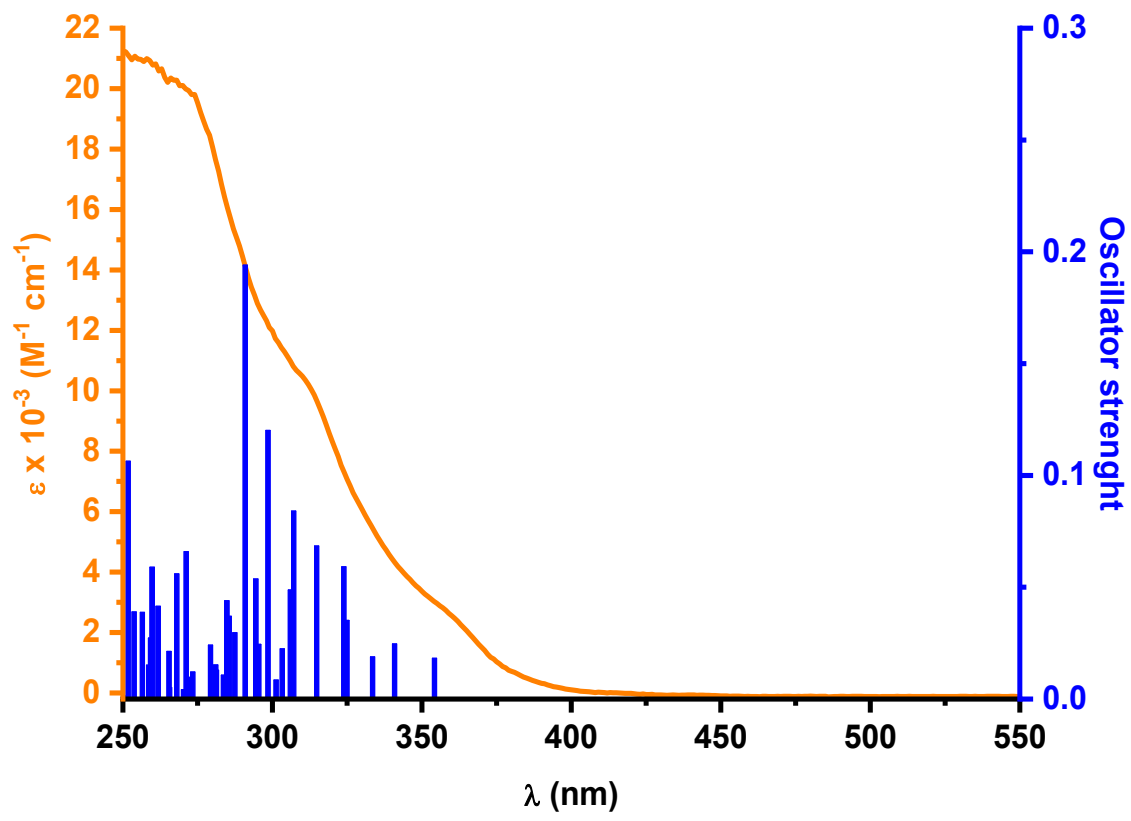

Figure S42. UV-Vis absorption experimental spectra (orange) and calculated transitions (blue) in CH_2Cl_2 ***N,N-cis-*(*OC*-6-24)-1**$_{Cl}$.

Table S12. Plots and composition (%) of the frontier MOs and spin density of the first triplet state in CH_2Cl_2 of complex ***N,N-cis-*(*OC*-6-24)-1**$_{Cl}$.

***N,N-cis-*(*OC*-6-24)-1**$_{Cl}$		
SOMO	SOMO-1	Spin density
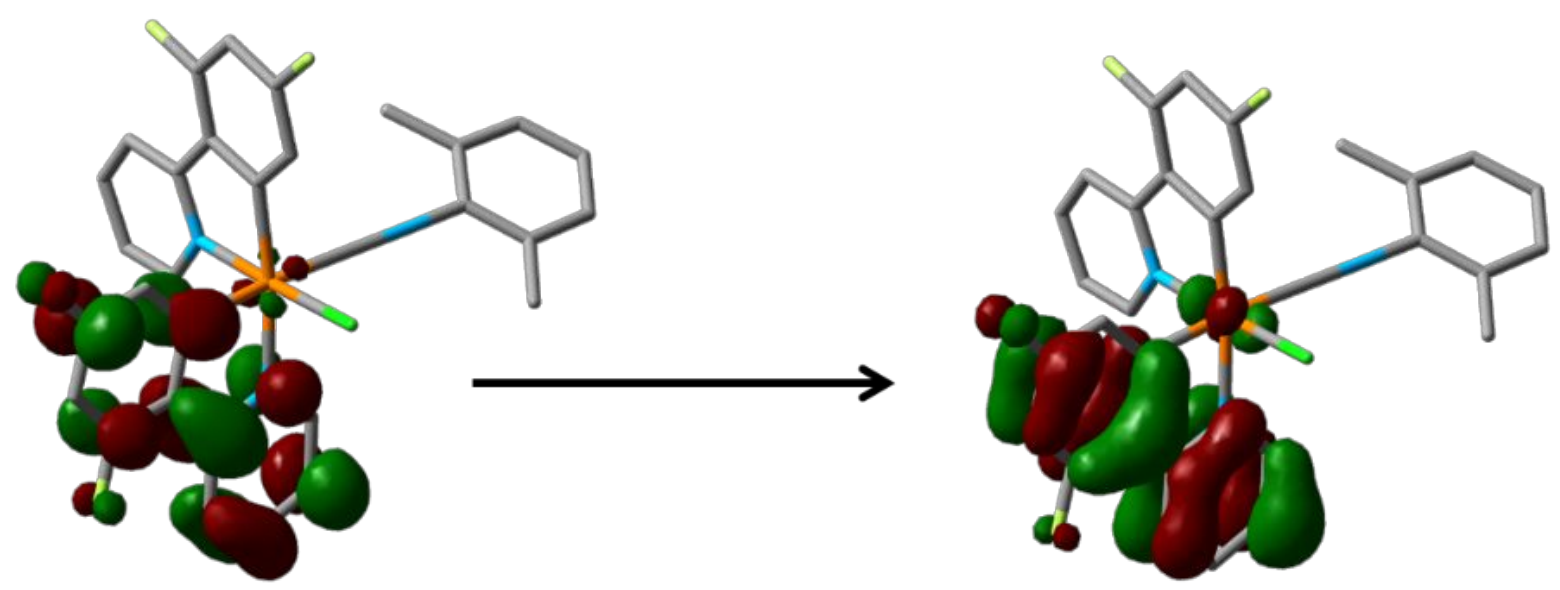		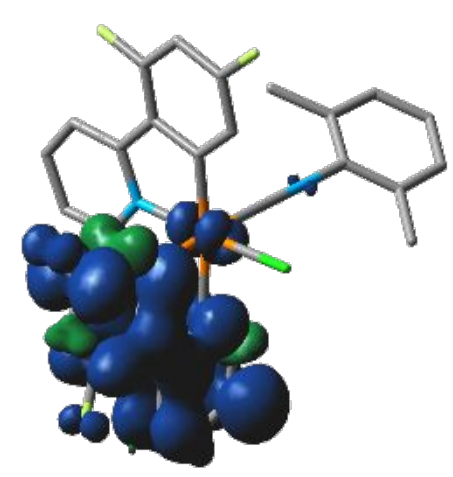
-3.32 eV<br>Ir 3 %, dfppy(1) 0 %, dfppy(2) 96 %, CNXyl 1 %, Cl 0%	-4.40 eV<br>Ir 5 %, dfppy (1) 0 %, dfppy (2) 94 %, CNXyl 1 %, Cl 0 %	0.0302 on Ir

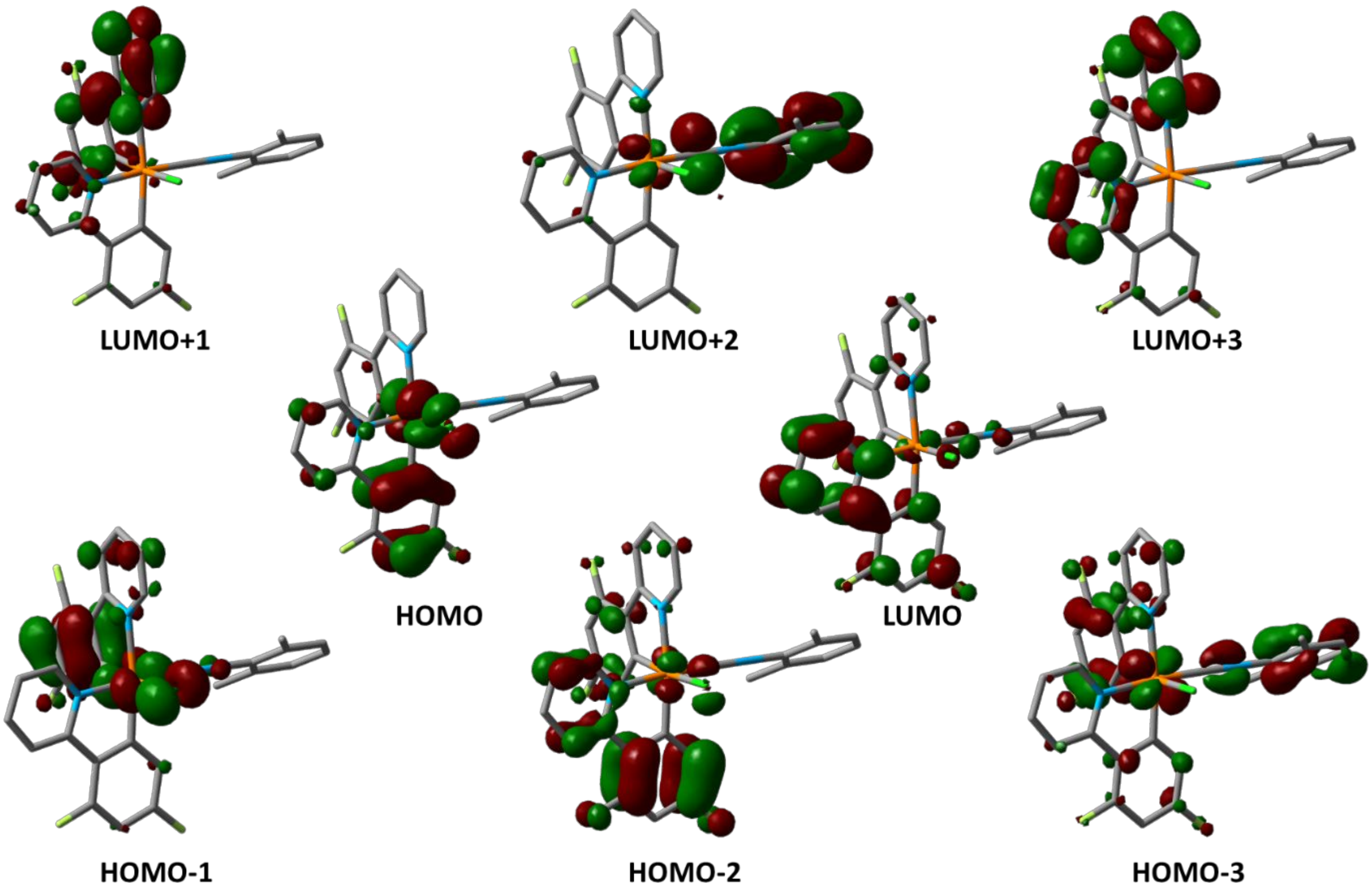

Figure S43. Selected frontier Molecular Orbitals for ***N,N-cis*-(*OC*-6-44)-1**$_{Cl}$ in the ground state in CH_2Cl_2.

Table S13. Composition (%) of frontier MOs in terms of ligands and metals in the ground state for ***N,N-cis*-(*OC*-6-44)-1**$_{Cl}$ in CH_2Cl_2.

***N,N-cis*-(*OC*-6-44)-1**$_{Cl}$						
**Orbital**	**Energy (eV)**	**Ir**	**dfppy(1)**	**dfppy(2)**	**CNXyl**	**Cl**
**LUMO+5**	-0.23	4	3	6	86	1
**LUMO+4**	-0.91	1	42	55	1	0
**LUMO+3**	-1.10	1	57	40	2	0
**LUMO+2**	-1.18	7	2	7	84	0
**LUMO+1**	-1.67	2	86	11	0	0
**LUMO**	-1.69	5	9	78	7	2
**HOMO**	-5.90	37	6	47	1	10
**HOMO-1**	-6.10	28	47	6	4	15
**HOMO-2**	-6.36	6	18	70	1	5
**HOMO-3**	-6.44	18	34	13	33	2
**HOMO-4**	-6.55	16	40	5	29	10
**HOMO-5**	-6.77	3	3	35	1	58

Table S14. Selected vertical excitation energies singlets (S_0) and first triplets computed by TD-DFT/SCRF (CH_2Cl_2) with the orbitals involved of complex ***N,N-cis-*(*OC*-6-44)-1**$_{Cl}$.

Complex	State	λ/nm	f	Transition (% Contribution)	
***N,N-cis-*(*OC*-6-44)-1**$_{Cl}$	**T$_1$**	424.2	-	H-2→LUMO (17%), HOMO→LUMO (52%)	ILCT/MLCT /XLCT
	**T$_2$**	413.7	-	H-3→L+1 (15%), H-2→L+1 (10%), H-1→L+1 (38%)	MLCT/L"LC T/ILCT/ XLCT
	**S$_1$**	359.6	0.0263	HOMO→LUMO (94%)	ILCT/MLCT /XLCT
	**S$_2$**	350.7	0.0074	HOMO→L+1 (94%)	ILCT/MLCT /XLCT
	**S$_3$**	333.7	0.0558	H-1→LUMO (92%)	ILCT/MLCT /XLCT
	**S$_4$**	332.2	0.0703	H-1→L+1 (94%)	ILCT/MLCT /XLCT
	**S$_5$**	310.6	0.0224	HOMO→L+2 (90%)	ML"CT/LL" CT/XL"CT
	**S$_6$**	303.6	0.0712	H-2→LUMO (69%), HOMO→L+3 (16%)	ILCT/MLCT /XLCT

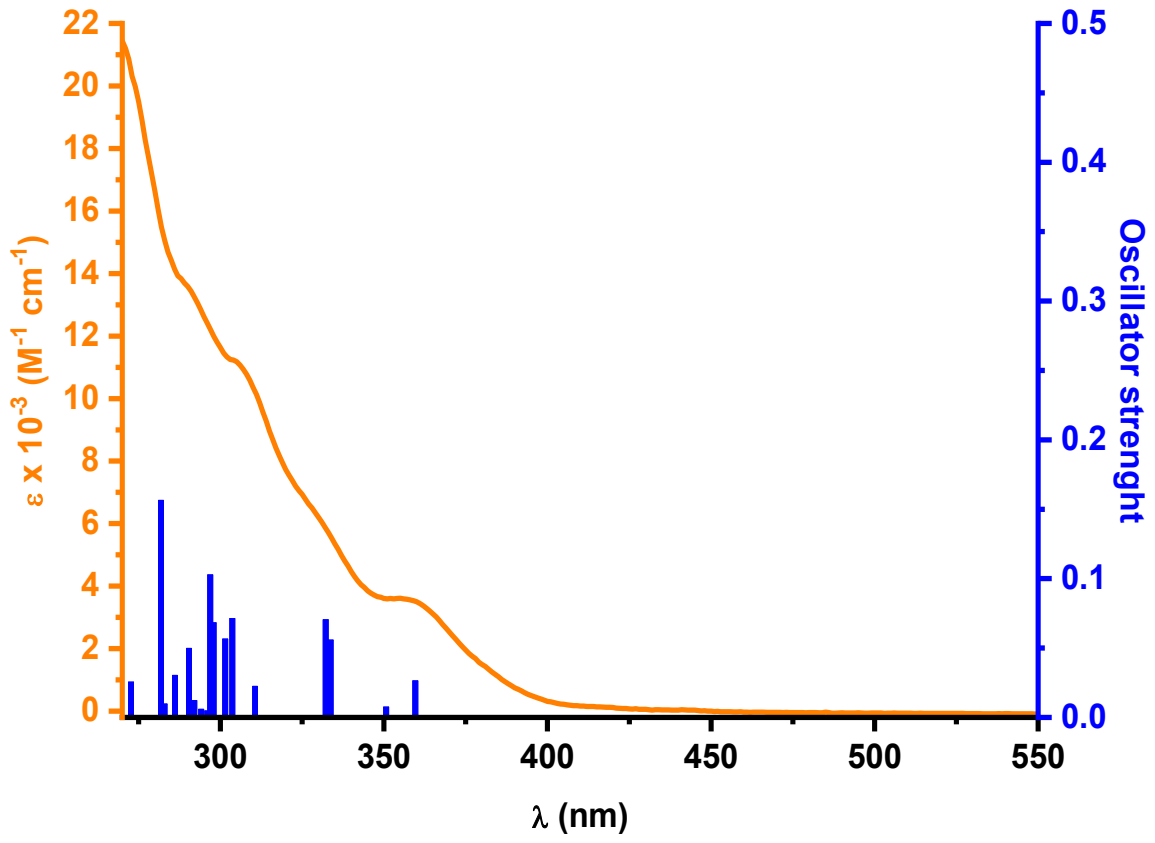

Figure S44. UV-Vis absorption experimental spectra (orange) and calculated transitions (blue) in CH_2Cl_2 ***N,N-cis-*(*OC*-6-44)-1**$_{Cl}$.

Table S15. Plots and composition (%) of the frontier MOs and spin density of the first triplet state in CH_2Cl_2 of complex ***N,N-cis-*(*OC*-6-44)-1**$_{Cl}$.

***N,N-cis-*(*OC*-6-44)-1**$_{Cl}$		
SOMO	SOMO-1	Spin density
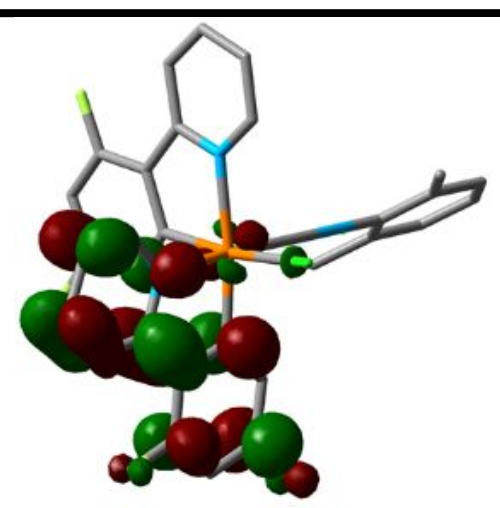 →	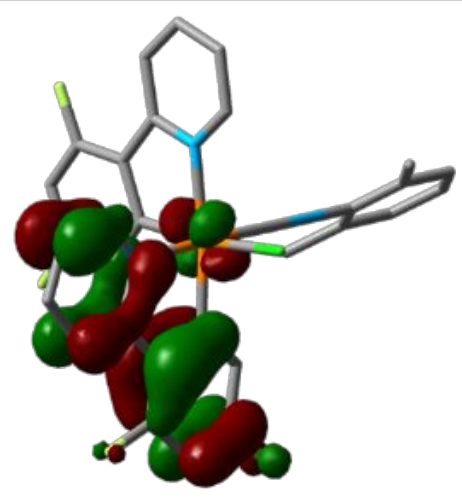	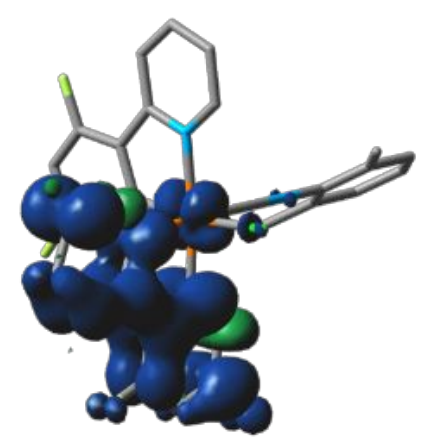
-3.31 eV<br>Ir 4 %, dfppy(1) 0 %, dfppy(2) 93 %, CNXyl 1 %, Cl 1%	-4.44 eV<br>Ir 10 %, dfppy (1) 1 %, dfppy (2) 88 %, CNXyl 1 %, Cl 1 %	0.0646 on Ir

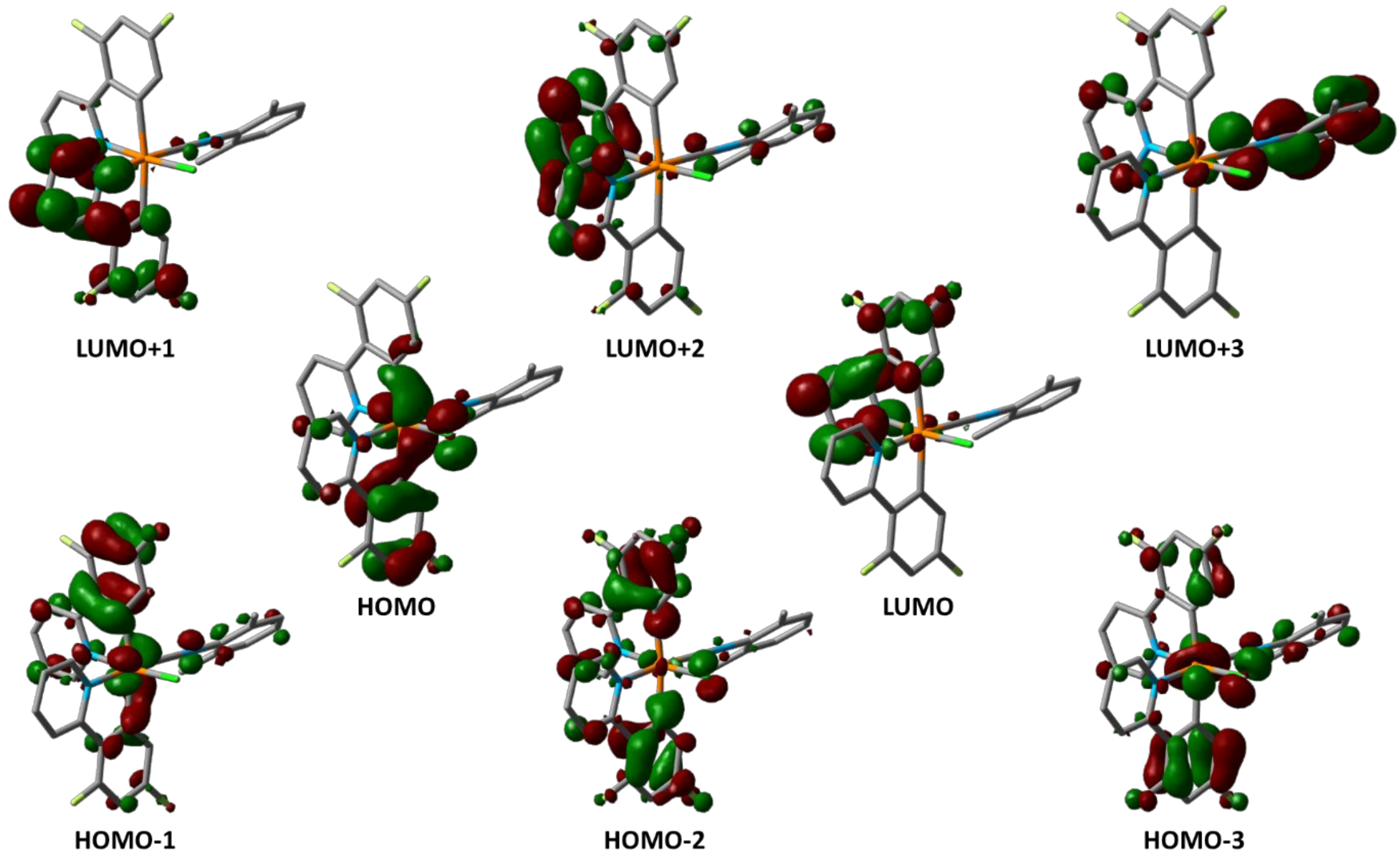

Figure S45. Selected frontier Molecular Orbitals for ***C,C-trans*-1**$_{Cl}$ in the ground state.

Table S16. Composition (%) of Frontier MOs in terms of ligands and metals in the ground state for ***C,C-trans*-1**$_{Cl}$ in CH_3CN.

***C,C-trans*-1**$_{Cl}$						
**Orbital**	**Energy (eV)**	**Ir**	**dfppy(1)**	**dfppy(2)**	**CNXyl**	**Cl**
**LUMO+5**	-0.65	56	19	4	6	14
**LUMO+4**	-0.90	1	20	76	3	0
**LUMO+3**	-1.10	5	6	5	83	0
**LUMO+2**	-1.16	1	74	22	2	0
**LUMO+1**	-1.65	3	3	89	5	0
**LUMO**	-1.78	3	92	2	2	0
**HOMO**	-5.95	33	9	39	4	14
**HOMO-1**	-6.14	20	46	20	12	1
**HOMO-2**	-6.26	3	40	49	2	7
**HOMO-3**	-6.32	25	21	25	16	13
**HOMO-4**	-6.52	7	32	53	1	6
**HOMO-5**	-6.59	21	46	7	5	21

Table S17. Selected vertical excitation energies singlets (S_0) and first triplets computed by TD-DFT/SCRF (CH_3CN) with the orbitals involved for complex ***C,C-trans*-1**$_{Cl}$.

Complex	State	λ/nm	*f*	Transition (% Contribution)	
***C,C-trans*-1**$_{Cl}$	T_1	419.3	-	H-2→LUMO (26%), H-1→LUMO (41%)	LLCT/MLCT/ L”LCT
	T_2	417.3	-	H-3→L+1 (11%), H-2→L+1 (17%), HOMO→L+1 (38%)	MLCT/LLCT/ L”LCT/L’LCT
	T_3	377.4	-	HOMO→LUMO (23%), HOMO→L+5 (37%)	MLCT/LLCT/ L’LCT/LMCT
	$\mathbf{S_1}$	354.2	0.0019	HOMO→LUMO (93%)	MLCT/L’LCT/LLCT
	$\mathbf{S_2}$	344.9	0.0237	HOMO→L+1 (93%)	MLCT/L’LCT
	$\mathbf{S_3}$	335.9	0.0346	H-1→LUMO (93%)	MLCT/LLCT/ L”LCT
	$\mathbf{S_5}$	318.9	0.0181	H-2→LUMO (42%), HOMO→L+5 (29%)	LLCT/LMCT
	$\mathbf{S_6}$	316.7	0.0327	H-2→LUMO (36%), HOMO→L+5 (34%)	LLCT/LMCT
	$\mathbf{S_9}$	305.8	0.0904	H-3→L+1 (85%)	MLCT/LLCT/ L”LCT
	$\mathbf{S_{10}}$	300.8	0.0723	H-4→LUMO (72%), H-2→LUMO (14%)	LLCT

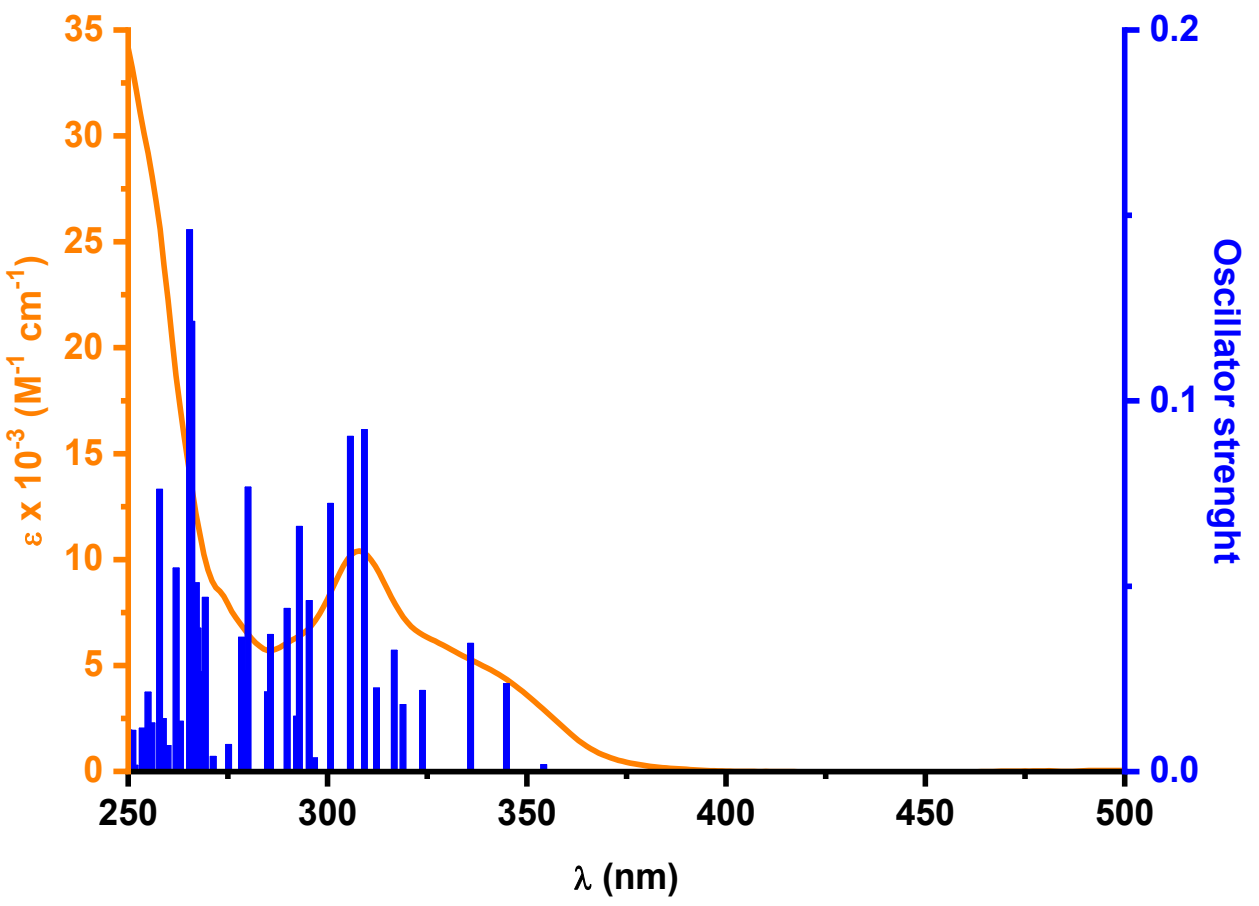

Figure S46. UV-Vis absorption experimental spectra (orange) and calculated transitions (blue) in CH_3CN ***C,C-trans*-1**$_{Cl}$.

Table S18. Plots and composition (%) of the frontier MOs and spin density of the first triplet state in CH_3CN of complex ***C,C-trans*-1**$_{Cl}$

***N,N- C,C-trans*-1**$_{Cl}$		
SOMO	SOMO-1	Spin density
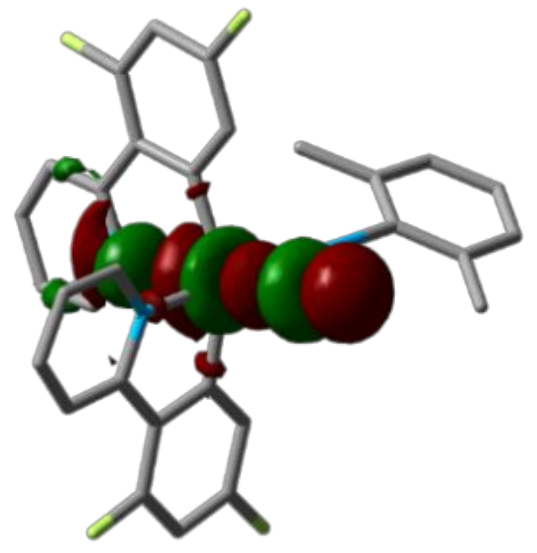 →	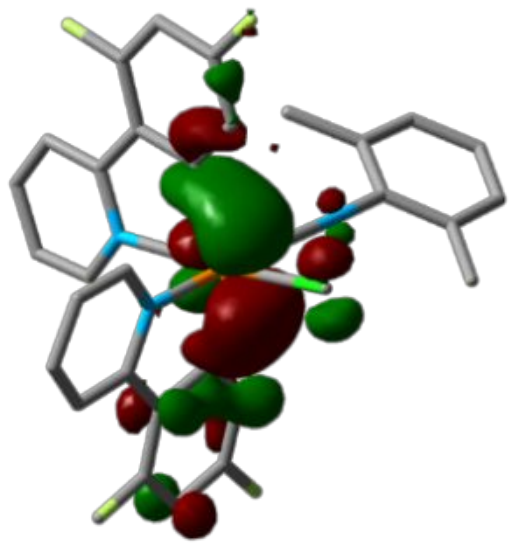	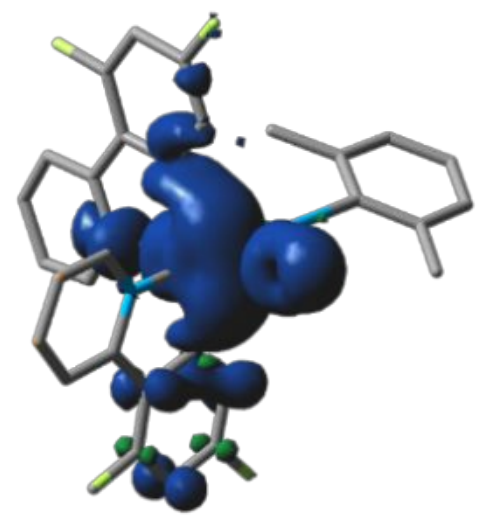
-5.01 eV<br>Ir 45 %, dfppy(1) 21 %, dfppy(2) 6 %, CNXyl 2 %, Cl 26 %	-3.42 eV<br>Ir 2 %, dfppy (1) 1 %, dfppy 2) 0 %, CNXyl 0 %, Cl 97 %	0.632 on Ir

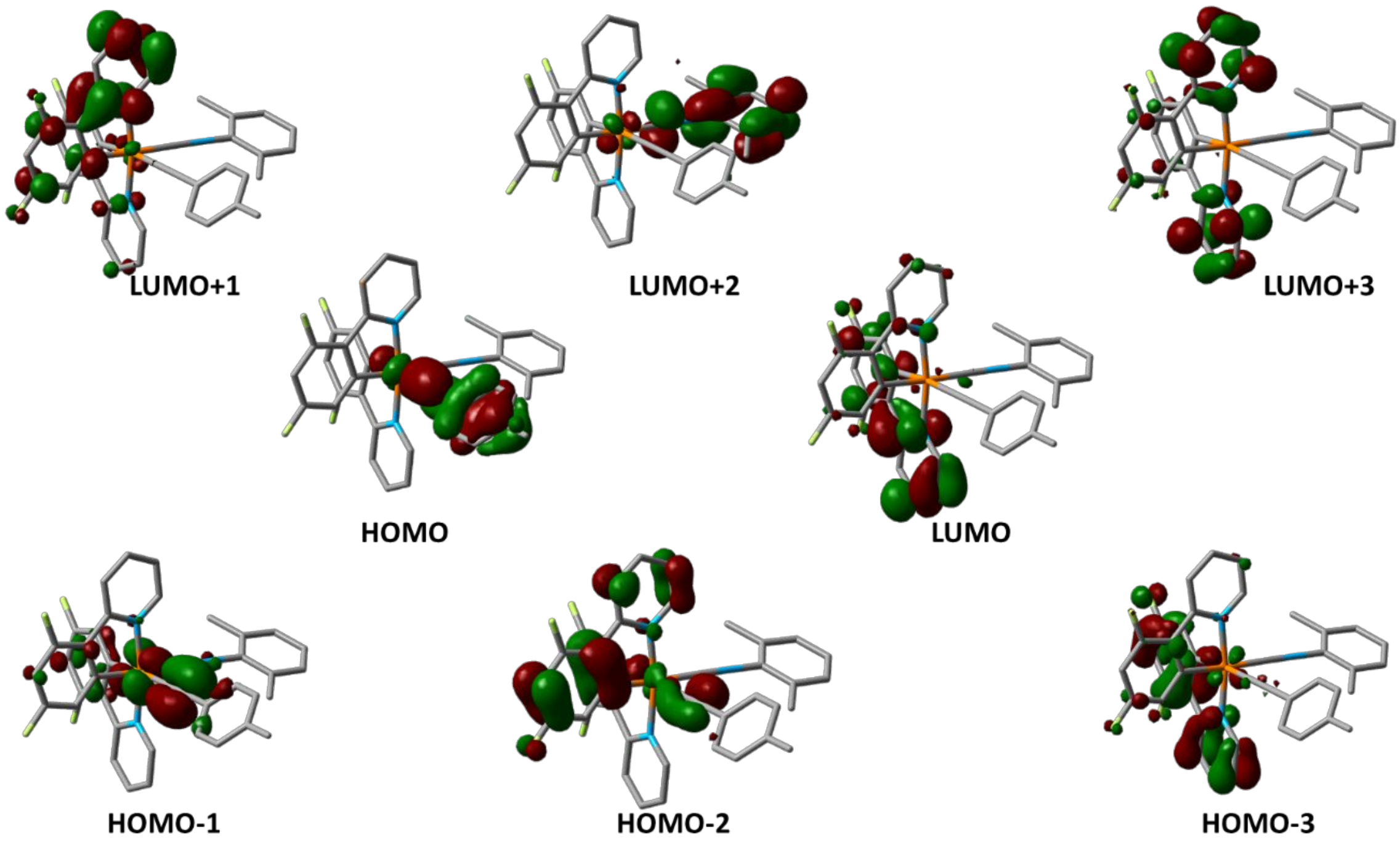

Figure S47. Selected frontier Molecular Orbitals for ***N,N-trans*-1a** in the ground state in CH_2Cl_2.

Table S19. Composition (%) of frontier MOs in terms of ligands and metals in the ground state for ***N,N-trans*-1a** in CH_2Cl_2.

***N,N-trans*-1a**						
**Orbital**	**Energy (eV)**	**Ir**	**dfppy(1)**	**dfppy(2)**	**CNXyl**	**CC-Tol**
**LUMO+5**	-0.27	3	0	5	5	87
**LUMO+4**	-0.90	2	47	51	1	0
**LUMO+3**	-0.99	2	50	47	1	0
**LUMO+2**	-1.22	5	3	3	89	0
**LUMO+1**	-1.53	3	84	11	2	1
**LUMO**	-1.57	2	12	84	2	0
**HOMO**	-5.21	13	2	3	1	81
**HOMO-1**	-5.65	33	10	12	2	43
**HOMO-2**	-6.14	6	67	5	1	22
**HOMO-3**	-6.19	6	14	74	2	5
**HOMO-4**	-6.32	12	55	22	6	4
**HOMO-5**	-6.46	25	20	18	34	4

Table S20. Selected vertical excitation energies singlets (S_0) and first triplets computed by TD-DFT/SCRF (CH_2Cl_2) with the orbitals involved of complex ***N,N-trans*-1a**.

**Complex**	**State**	**λ/nm**	f	Transition (% Contribution)	
***N,N-trans*-1a**	**T_1**	423.8	-	H-3→LUMO (29%), H-1→LUMO (12%), HOMO→LUMO (33%)	L'LCT/IL/MLCT/
	**T_2**	421.4	-	H-4→L+1 (10%), H-2→L+1 (15%), H-1→L+1 (28%), HOMO→L+1 (10%)	L'LCT/MLCT/ILCT
	**T_3**	411.2	-	HOMO→L+1 (59%), HOMO→L+5 (13%)	MLCT/L'LCT/IL'
	**S_1**	404.6	0.0132	HOMO→LUMO (92%)	L'LCT/ MLCT
	**S_2**	401.1	0.0097	HOMO→L+1 (92%)	L'LCT/ MLCT
	**S_3**	367.6	0.0109	H-1→LUMO (96%)	L'LCT/ MLCT
	**S_4**	366.7	0.0059	H-1→L+1 (96%)	L'LCT/ MLCT
	**S_8**	330.9	0.0101	H-1→L+2 (85%), HOMO→L+4 (12%)	L'LCT/ ML"CT/LL"CT/L'L"CT/ MLCT

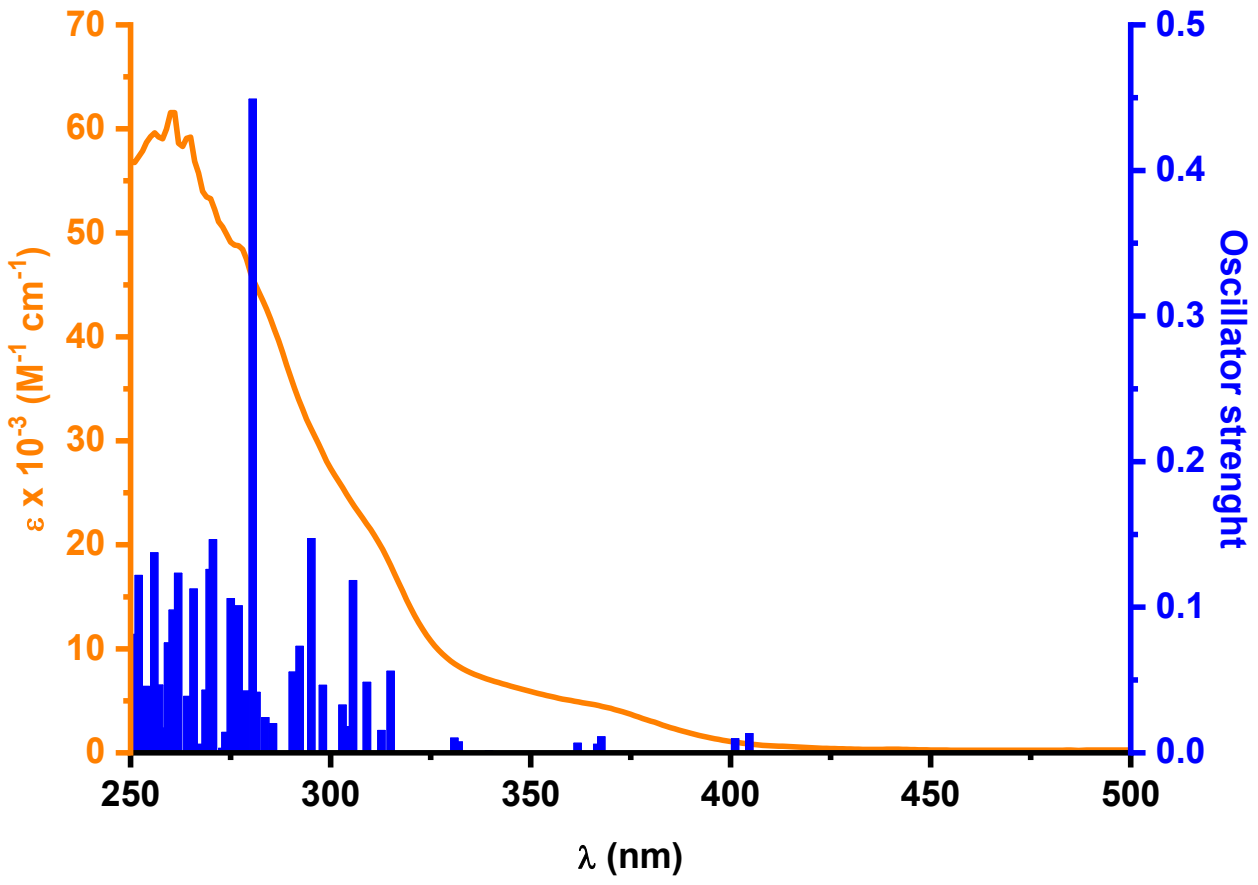

Figure S48. UV-Vis absorption experimental spectra (orange) and calculated transitions (blue) of ***N,N-trans*-1a** in CH_2Cl_2

Table S21. Plots and composition (%) of the frontier MOs and spin density of the first triplet state in CH_2Cl_2 of complex ***N,N-trans*-1a**.

***N,N-trans*-1a**		
SOMO	SOMO-1	Spin density
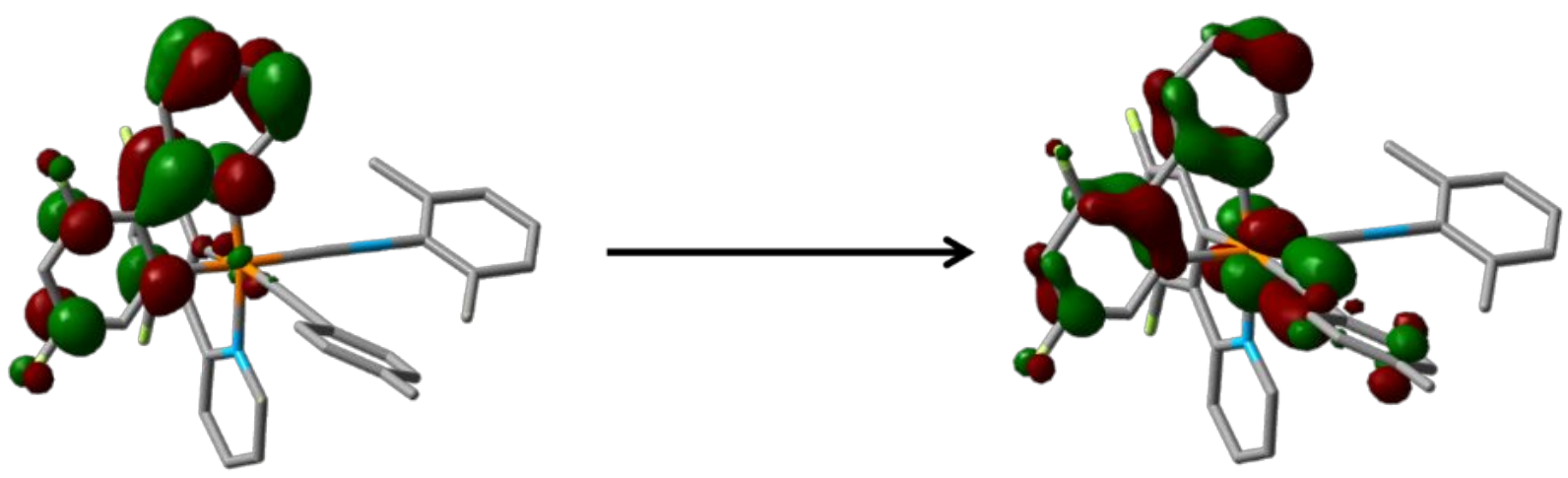		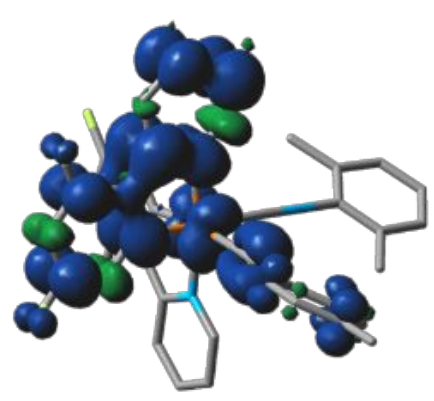
-2.87 eV<br>Ir 2 %, dfppy(1) 96 %, dfppy(2) 1 %, CNXyl 0 %, CC-Tol 1%	-4.30 eV<br>Ir 23 %, dfppy (1) 47 %, dfppy 2) 2 %, CNXyl 1 %, CC-Tol 27 %	0.133 on Ir

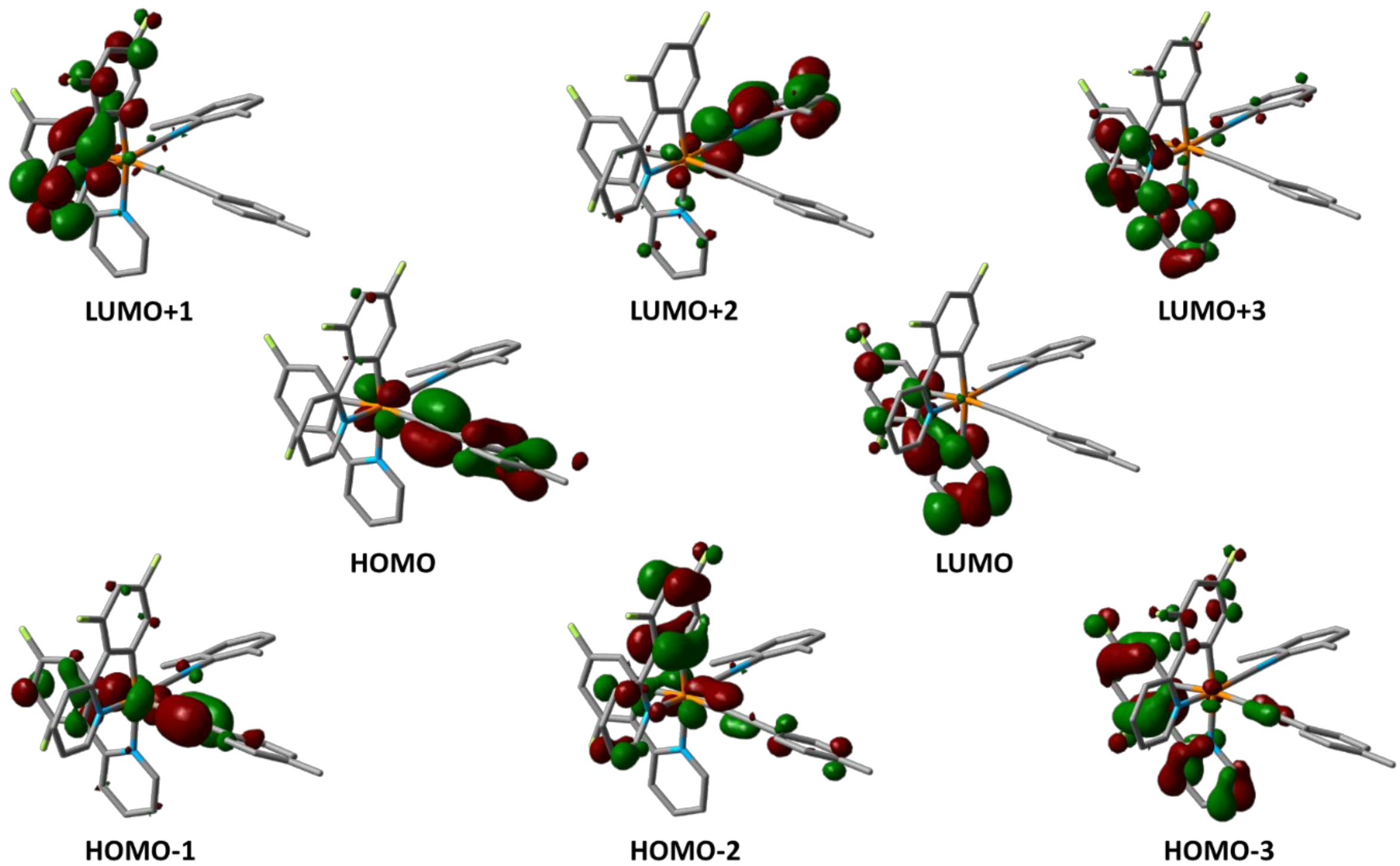

Figure S49. Selected frontier Molecular Orbitals for ***N,N-cis*-(*OC*-6-32)-1a** in the ground state in CH_2Cl_2.

Table S22. Composition (%) of frontier MOs in terms of ligands and metals in the ground state for ***N,N-cis*-(*OC*-6-32)-1a** in CH_2Cl_2.

***N,N-cis*-(*OC*-6-32)-1a**						
**Orbital**	**Energy (eV)**	**Ir**	**dfppy(1)**	**dfppy(2)**	**CNXyl**	**CC-Tol**
**LUMO+5**	-0.31	4	4	1	2	89
**LUMO+4**	-0.84	1	46	53	0	0
**LUMO+3**	-1.02	2	48	42	8	0
**LUMO+2**	-1.14	6	6	6	82	1
**LUMO+1**	-1.54	2	4	90	3	1
**LUMO**	-1.58	2	93	4	1	0
**HOMO**	-5.20	19	3	5	0	73
**HOMO-1**	-5.77	26	20	6	3	45
**HOMO-2**	-6.03	12	7	61	1	19
**HOMO-3**	-6.26	3	74	14	3	7
**HOMO-4**	-6.38	6	18	60	8	8
**HOMO-5**	-6.41	25	8	15	50	2

Table S23. Selected vertical excitation energies singlets (S_0) and first triplets computed by TD-DFT/SCRF (CH_2Cl_2) with the orbitals involved of complex ***N,N-cis-*(*OC*-6-32)-1a.**

Complex	State	λ/nm	f	Transition (% Contribution)	
***N,N-cis-*(*OC*-6-32)-1a**	T_1	429.7	-	H-2→L+1 (21%), HOMO→L+1 (60%)	L'LCT/ MLCT
	T_2	420.1	-	H-3→LUMO (19%), HOMO→LUMO (47%)	L'LCT/ILCT / MLCT
	T_3	410.1	-	H-3→LUMO (19%), H-1→LUMO (11%), HOMO→LUMO (10%), HOMO→L+5 (32%)	ILCT/MLCT /L'LCT/ ML'CT
	S_1	409.9	0.0024	HOMO→LUMO (84%), HOMO→L+1 (14%)	L'LCT/ MLCT
	S_2	409.1	0.0113	HOMO→LUMO (14%), HOMO→L+1 (84%)	L'LCT/ MLCT
	S_3	357.4	0.0189	HOMO→L+2 (94%)	ML"CT/ L'L"CT
	S_4	352.5	0.0315	H-1→LUMO (95%)	L'LCT/ MLCT
	S_5	348.0	0.0418	H-1→L+1 (94%)	L'LCT/ MLCT
	S_6	345.6	0.0143	HOMO→L+3 (93%)	L'LCT/ MLCT
	S_8	321.9	0.0675	H-2→L+1 (91%)	L'LCT/ MLCT

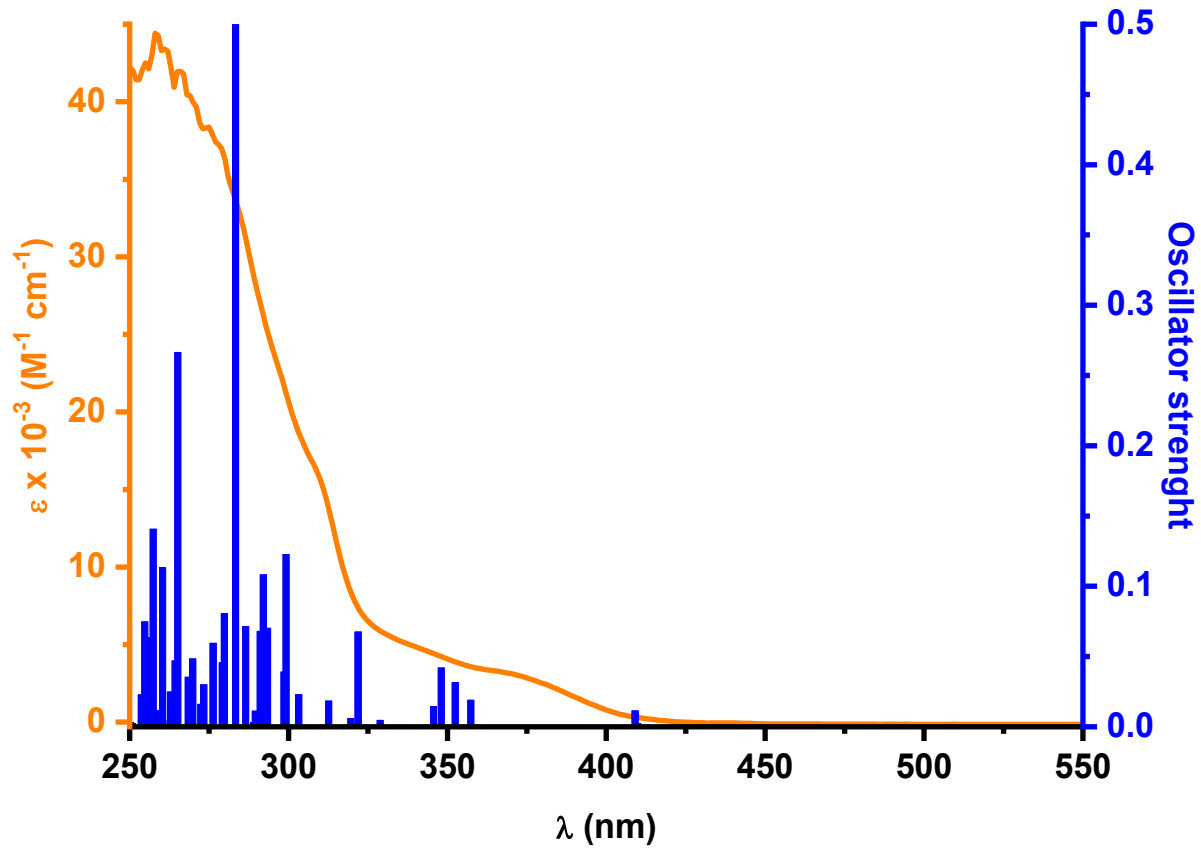

Figure S50. UV-Vis absorption experimental spectra (orange) and calculated transitions (blue) of ***N,N-cis-*(*OC*-6-32)-1a** in CH_2Cl_2

Table S24. Plots and composition (%) of the frontier MOs and spin density of the first triplet state of complex ***N,N-cis-*(*OC*-6-32)-1a** in CH_2Cl_2.

***N,N-cis-*(*OC*-6-32)-1a**		
SOMO	SOMO-1	Spin density
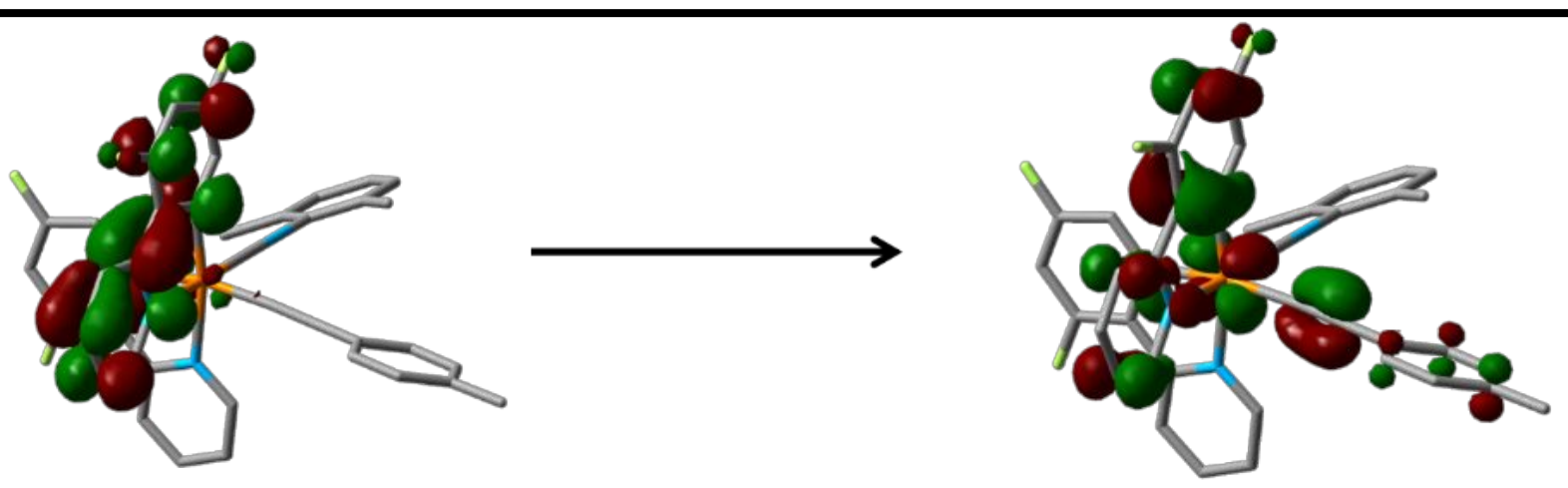		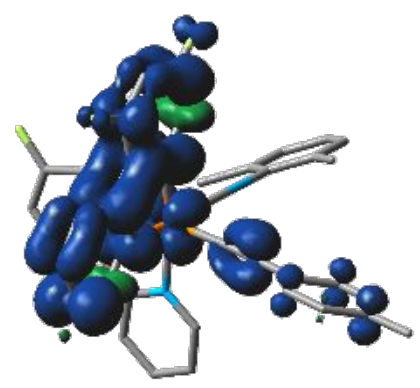
-2.80 eV<br>Ir 2 %, dfppy(1) 1 %, dfppy(2) 95 %, CNXyl 1 %, CC-Tol 1%	-4.29 eV<br>Ir 21 %, dfppy (1) 2 %, dfppy (2) 55 %, CNXyl 1 %, CC-Tol 21 %	0.1210 on Ir

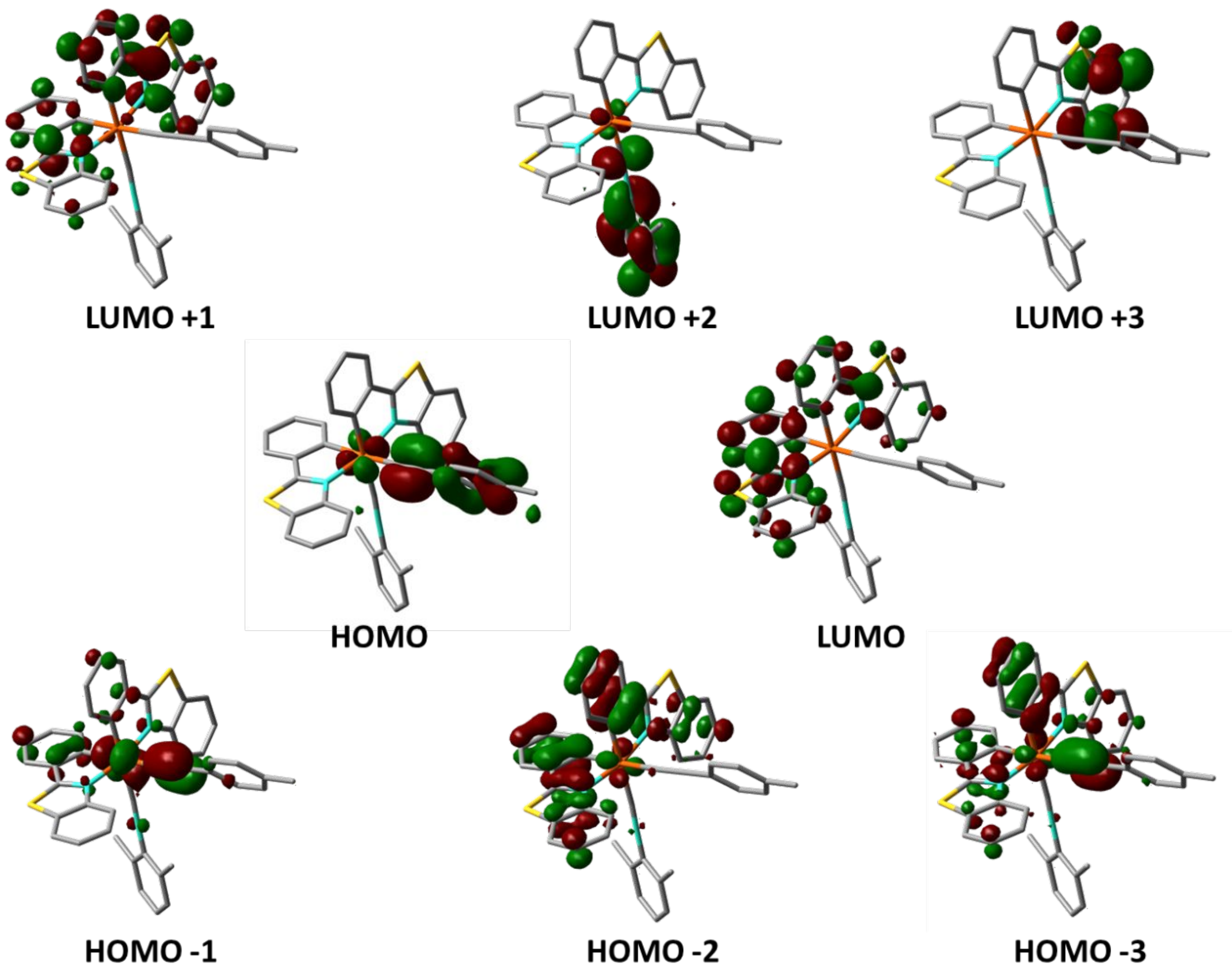

Figure S51. Selected frontier Molecular Orbitals for ***N,N-trans*-2a** in the ground state.

Table S25. Composition (%) of frontier MOs in terms of ligands and metals in the ground state for ***N,N-trans*-2a** in CH_2Cl_2.

N,N-trans-2a						
**Orbital**	**Energy (eV)**	**Ir**	**pbt(1)**	**pbt(2)**	**CNXyl**	**CC-Tol**
**LUMO+5**	-0.26	29	35	35	0	0
**LUMO+4**	-0.38	1	1	94	2	1
**LUMO+3**	-0.44	2	96	1	1	0
**LUMO+2**	-1.18	5	1	0	94	0
**LUMO+1**	-1.77	2	63	34	1	0
**LUMO**	-1.85	1	35	64	0	0
**HOMO**	-5.16	14	1	3	0	82
**HOMO-1**	-5.51	32	14	17	2	35
**HOMO-2**	-6.01	7	44	44	3	3
**HOMO-3**	-6.08	7	34	25	3	31
**HOMO-4**	-6.28	19	47	19	7	9
**HOMO-5**	-6.40	14	24	38	18	5

Table S26. Selected vertical excitation energies singlets (S_0) and first triplets computed by TD-DFT/SCRF (CH_2Cl_2) with the orbitals involved of complex ***N,N-trans*-2a**.

**Complex**	**State**	**λ/nm**	***f***	**Transition (% Contribution)**	
***N,N-trans*-2a**	**T_1**	482.2	-	H-2→L+1 (14%), H-1→LUMO (14%), H-1→L+1 (16%), HOMO→LUMO (12%), HOMO→L+1 (13%)	L'LCT/ MLCT/ IL
	**T_2**	477.3	-	H-3→LUMO (11%), H-2→LUMO (22%), H-2→L+1 (10%), H-1→LUMO (23%), H-1→L+1 (10%)	L'LCT/ MLCT/IL
	**T_3**	451.9	-	H-1→L+1 (14%), HOMO→LUMO (59%), HOMO→L+1 (11%)	L'LCT/ MLCT
	**S_1**	452.9	0.0091	HOMO→LUMO (89%)	L'LCT/MLCT
	**S_2**	441.1	0.0036	HOMO→LUMO (10%), HOMO→L+1 (88%)	L'LCT/MLCT
	**S_3**	416.9	0.0552	H-1→LUMO (94%)	L'LCT/ MLCT
	**S_6**	343.9	0.0574	H-2→LUMO (87%)	IL
	**S_7**	342.1	0.0781	H-3→LUMO (77%), H-3→L+1 (10%)	L'LCT/IL
	**S_9**	338.4	0.2556	H-2→L+1 (87%)	IL

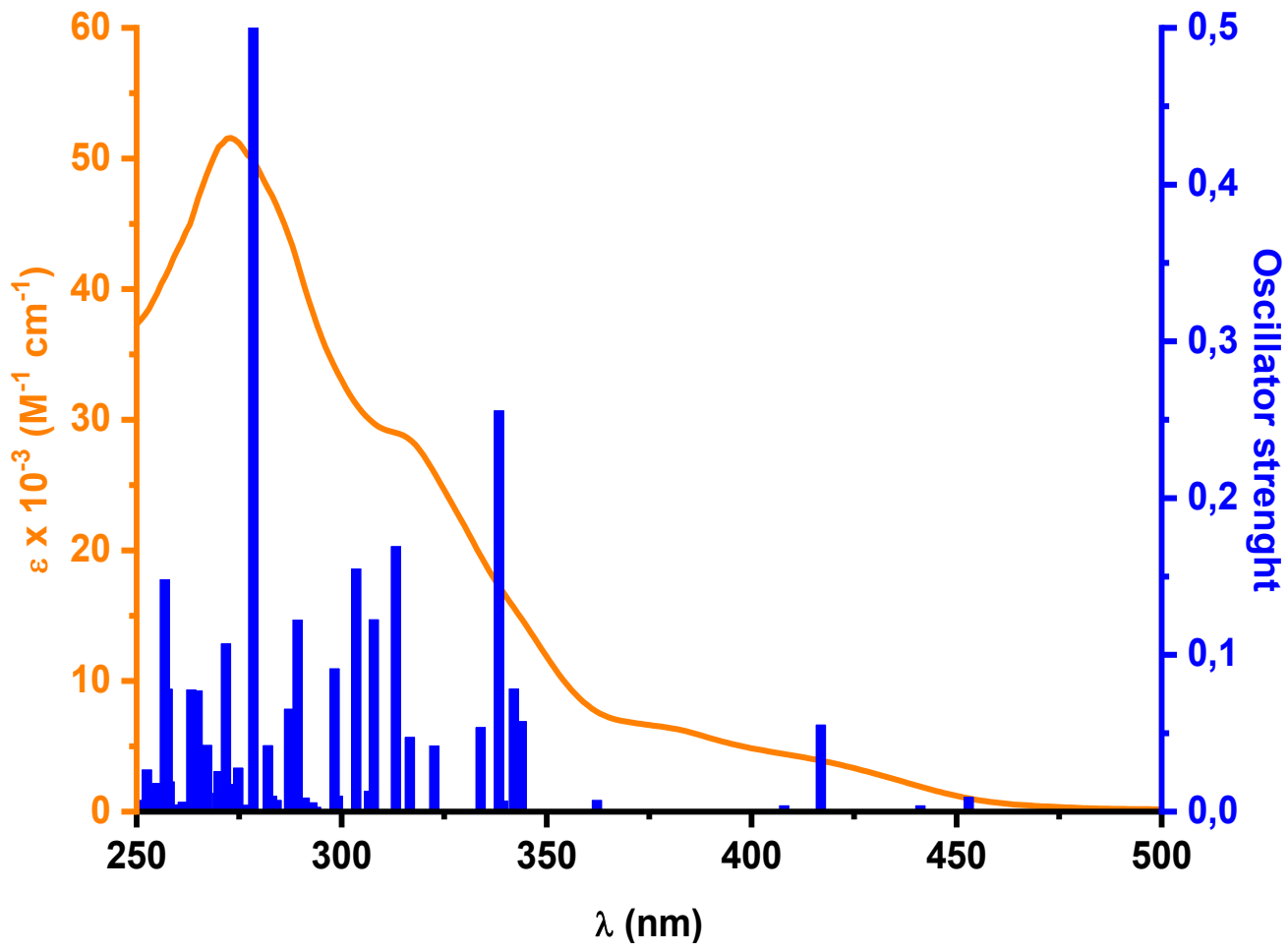

Figure S52. UV-Vis absorption experimental spectra (orange) and calculated transitions (blue) in CH_2Cl_2 ***N,N-trans*-2a**.

Table S27. Plots and composition (%) of the frontier MOs and spin density of the first triplet state in CH_2Cl_2 of ***N,N-trans*-2a**

***N,N-trans*-2a**		
SOMO	SOMO-1	Spin density
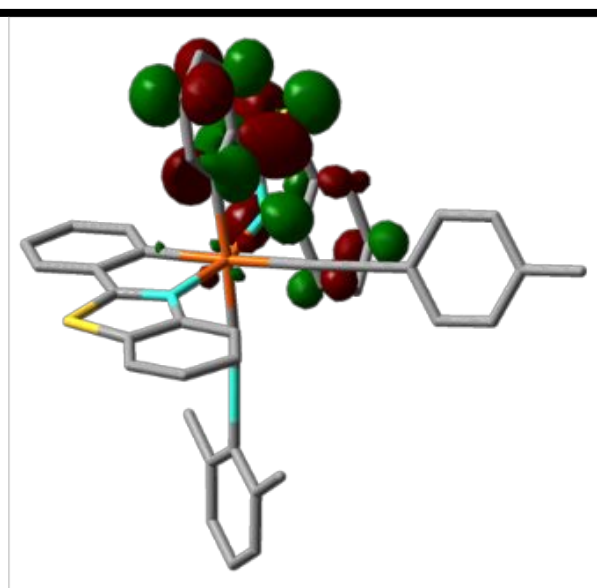	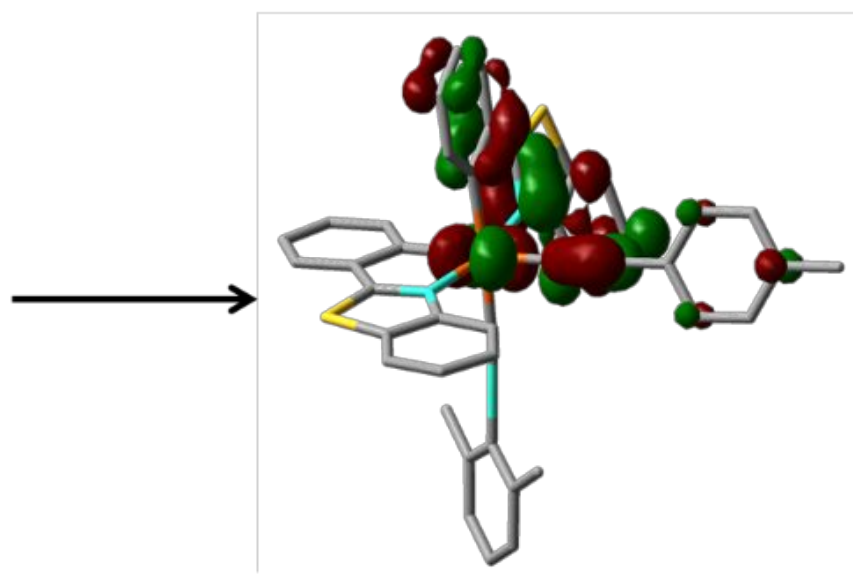	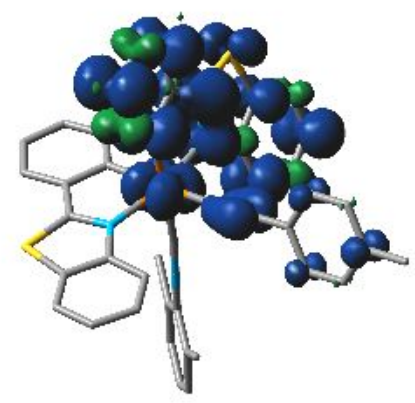
-3.26 eV<br>Ir 2 %, pbt(1) 96 %, pbt(2) 2 %, CNXyl 0 %, CC-Tol 1 %	-4.15 eV<br>Ir 23 %, pbt(1) 54 %, pbt(2) 2 %, CNXyl 1 %, CC-Tol 21 %	0.125 on Ir

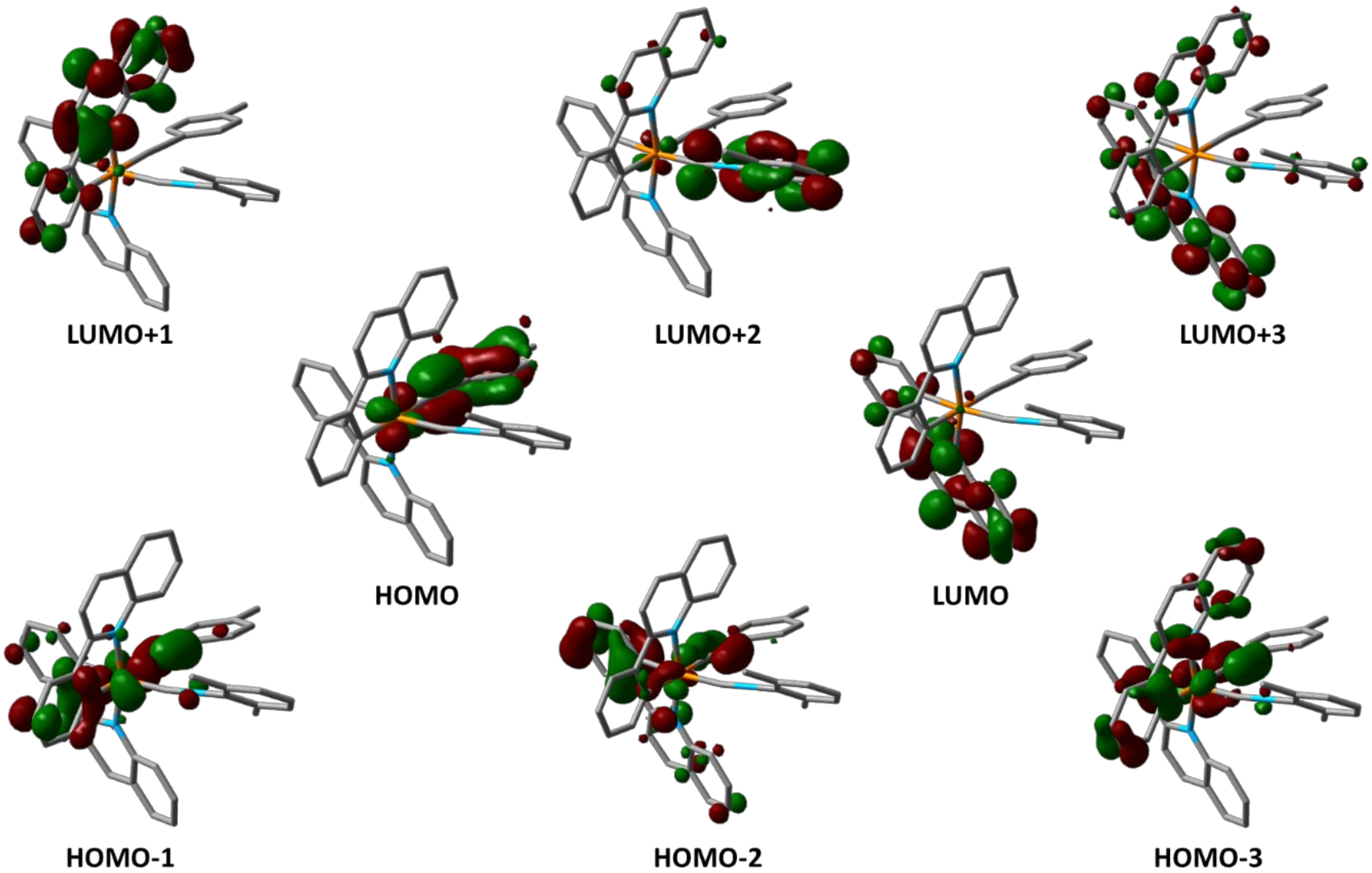

Figure S53. Selected frontier Molecular Orbitals for ***N,N-trans*-3a** in the ground state.

Table S28. Composition (%) of Frontier MOs in terms of ligands and metals in the ground state for ***N,N-trans*-3a** in CH_2Cl_2.

N,N-trans-3a						
**Orbital**	**Energy (eV)**	**Ir**	**dfppy(1)**	**dfppy(2)**	**CNXyl**	**CCR**
**LUMO+5**	-0.23	4	5	3	3	85
**LUMO+4**	-0.91	1	32	65	3	0
**LUMO+3**	-1.02	1	65	25	9	0
**LUMO+2**	-1.11	3	6	10	80	0
**LUMO+1**	-1.95	2	0	97	0	0
**LUMO**	-1.98	2	96	1	2	0
**HOMO**	-5.09	18	2	4	0	77
**HOMO-1**	-5.47	30	12	29	6	25
**HOMO-2**	-5.86	7	63	6	0	24
**HOMO-3**	-6.03	13	6	52	5	24
**HOMO-4**	-6.25	8	69	11	6	5
**HOMO-5**	-6.33	6	14	62	3	15

Table S29. Selected vertical excitation energies singlets (S_0) and first triplets computed by TD-DFT/SCRF (CH_2Cl_2) with the orbitals involved for complex ***N,N-trans*-3a**

Complex	State	λ/nm	*f*	Transition (% Contribution)	
***N,N-trans*-3a**	T_1	520.5	-	H-2→LUMO (14%), HOMO→LUMO (69%)	**L'LCT/MLCT**
	T_2	505.2	-	H-3→L+1 (18%), H-1→L+1 (21%), HOMO→L+1 (49%)	MLCT/L'LCT/ LLCT
	T_3	475.9	-	H-4→LUMO (11%), H-2→LUMO (28%), H-1→LUMO (18%), HOMO→LUMO (29%)	MLCT/LLCT/ L'LCT
	**S_1**	501.4	0.0011	HOMO→LUMO (98%)	MLCT/L'LCT
	**S_2**	484.1	0.0020	HOMO→L+1 (98%)	MLCT/L'LCT
	**S_3**	437.6	0.0257	H-1→LUMO (98%)	MLCT/LLCT/ L'LCT
	**S_5**	380.1	0.0628	H-2→LUMO (91%)	L'LCT
	**S_6**	372.2	0.0025	H-2→L+1 (96%)	L'LCT/LLCT
	**S_8**	361.3	0.0337	H-3→LUMO (84%)	MLCT/LLCT/ L'LCT
	**S_{10}**	352.1	0.0887	H-3→L+1 (92%)	MLCT/L'LCT

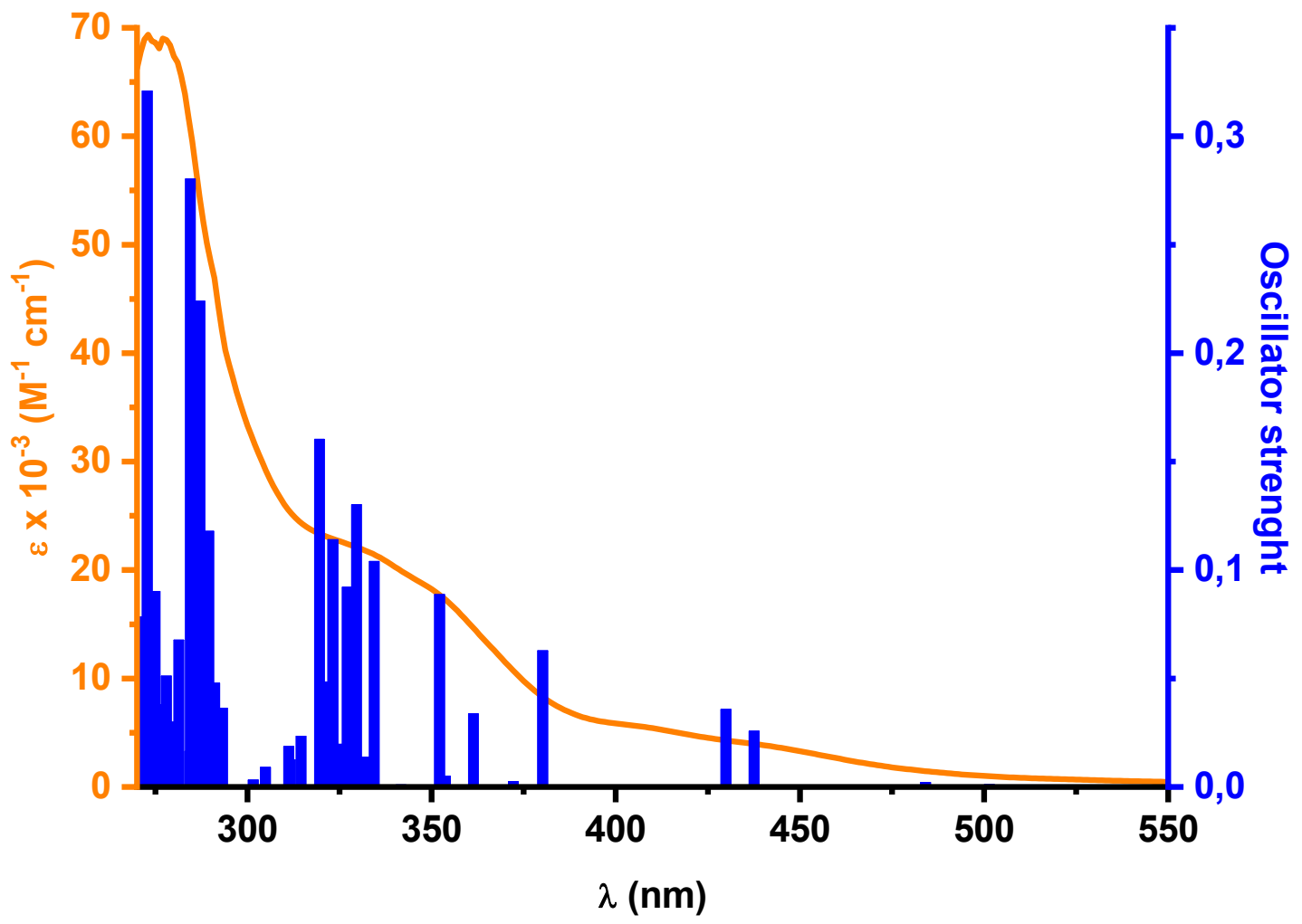

Figure S54. UV-Vis absorption experimental spectra (orange) and calculated transitions (blue) in CH_2Cl_2 ***N,N-trans*-3a.**

Table S30. Plots and composition (%) of the frontier MOs and spin density of the first triplet state in CH_2Cl_2 of complex ***N,N-trans*-3a.**

***N,N-trans*-3a**		
SOMO	SOMO-1	Spin density
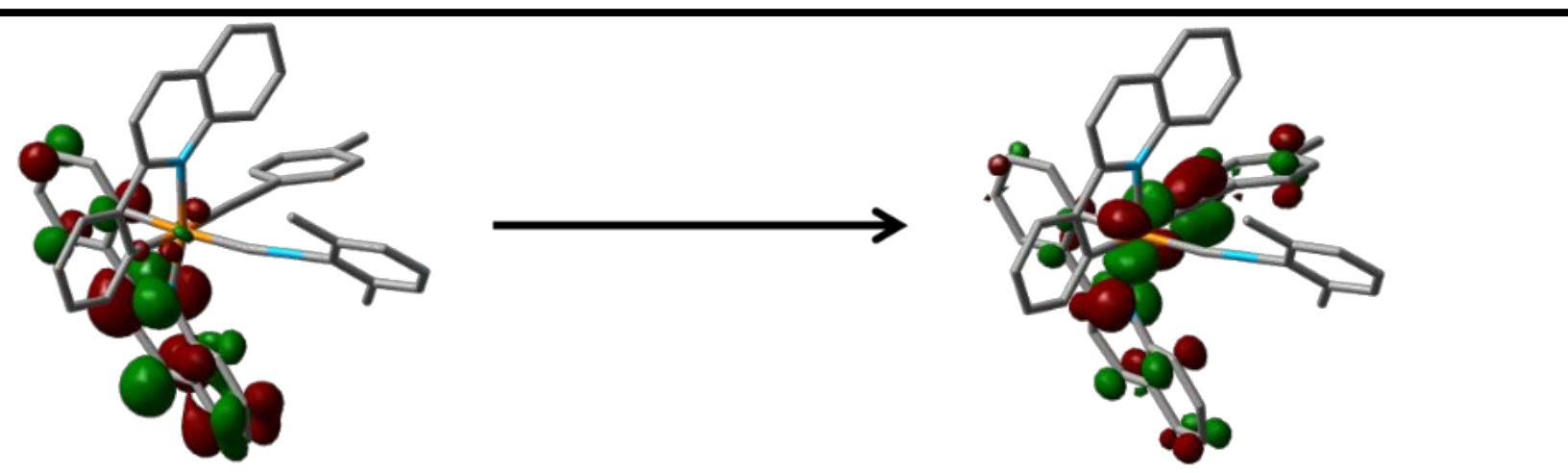		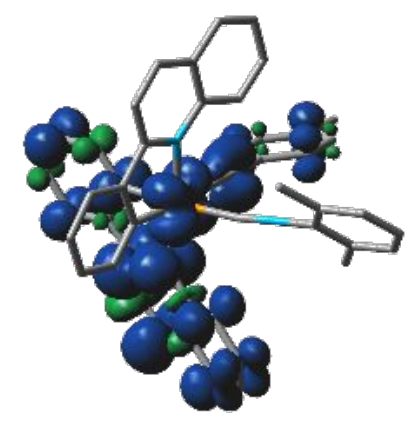
-3.25 eV<br>Ir 2 %, dfppy(1) 95 %, dfppy(2) 2 %, CNXyl 1 %, CCR 0 %	-4.14 eV<br>Ir 31 %, dfppy (1) 39 %, dfppy (2) 3 %, CNXyl 1 %, CCR 27 %	0.180 on Ir

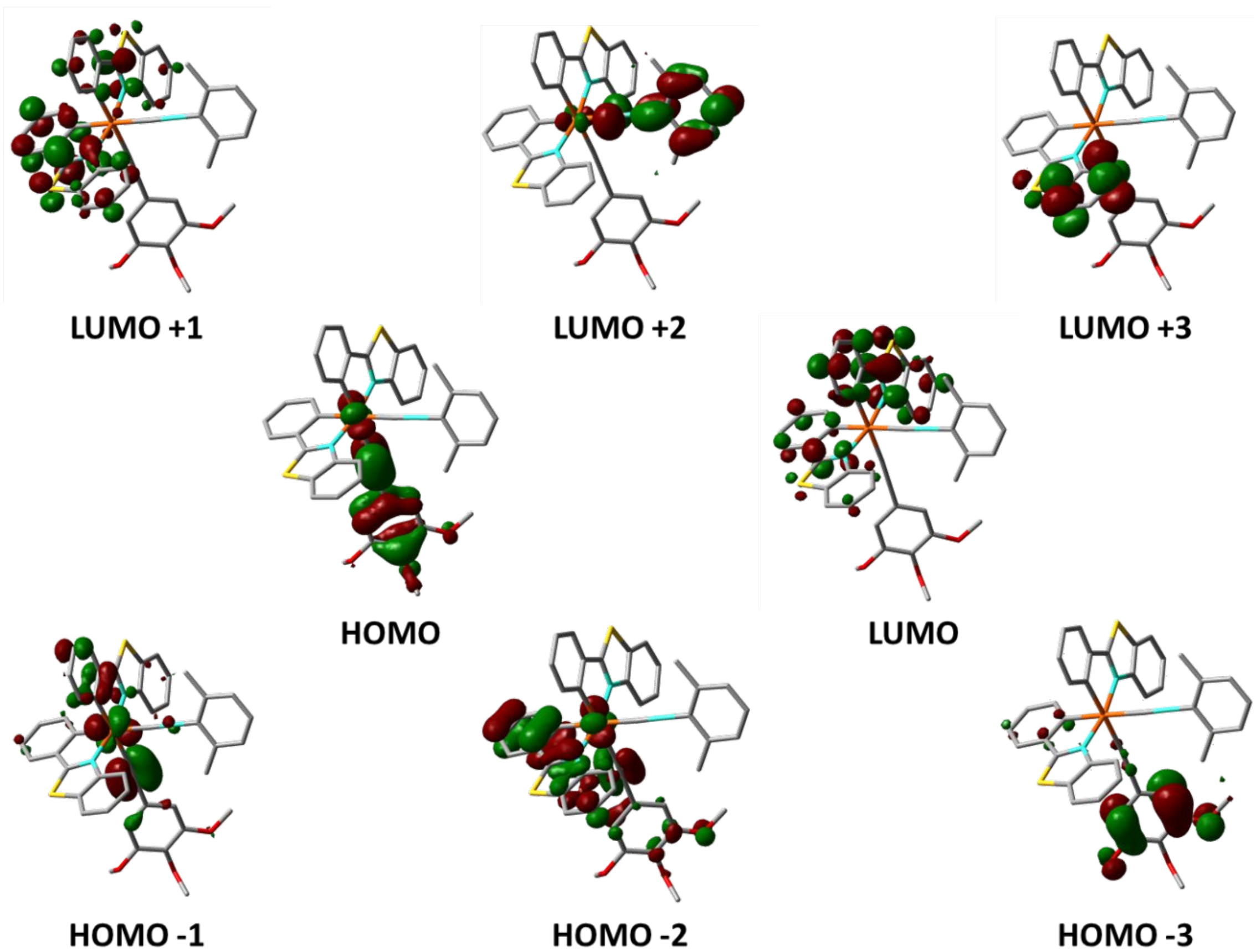

Figure S55. Selected frontier Molecular Orbitals for ***N,N-trans*-2b** in the ground state.

Table S31. Composition (%) of frontier MOs in terms of ligands and metals in the ground state for ***N,N-trans*-2b** in CH_2Cl_2.

***N,N-trans*-2b**						
**Orbital**	**Energy (eV)**	**Ir**	**pbt(1)**	**pbt(2)**	**CNXyl**	**CC-$C_6H_2(OMe)_3$**
**LUMO+5**	-0.28	19	15	26	2	37
**LUMO+4**	-0.38	2	1	87	0	10
**LUMO+3**	-0.45	2	95	1	1	1
**LUMO+2**	-1.19	5	2	1	93	0
**LUMO+1**	-1.79	2	67	30	1	0
**LUMO**	-1.85	1	30	68	0	0
**HOMO**	-5.12	15	2	3	1	79
**HOMO-1**	-5.65	28	7	25	3	36
**HOMO-2**	-5.91	13	56	1	1	28
**HOMO-3**	-6.04	0	10	0	0	89
**HOMO-4**	-6.08	11	10	60	4	15
**HOMO-5**	-6.38	4	69	13	1	14

Table S32. Selected vertical excitation energies singlets (S_0) and first triplets computed by TD-DFT/SCRF (CH_2Cl_2) with the orbitals involved of complex ***N,N-trans*-2b**.

**Complex**	**State**	**λ/nm**	***f***	**Transition (% Contribution)**	
***N,N-trans*-2b**	**T_1**	487.1	-	H-2→LUMO (12%), H-2→L+1 (20%), HOMO→LUMO (31%), HOMO→L+1 (22%)	L'LCT/MLCT
	**T_2**	480.2	-	H-4→LUMO (23%), H-1→LUMO (17%), HOMO→LUMO (13%), HOMO→L+1 (20%)	L'LCT/MLCT
	**T_3**	448.2	-	H-4→LUMO (12%), HOMO→LUMO (47%), HOMO→L+1 (11%)	L'LCT/MLCT
	**S_1**	460.9	0.0078	HOMO→LUMO (72%), HOMO→L+1 (26%)	L'LCT/MLCT
	**S_2**	451.7	0.0044	HOMO→LUMO (26%), HOMO→L+1 (71%)	L'LCT/MLCT
	**S_3**	395.0	0.0754	H-1→LUMO (98%)	L'LCT/MLCT
	**S_6**	357.5	0.0881	H-2→LUMO (88%)	L'LCT/MLCT
	**S_7**	352.0	0.0557	H-2→L+1 (87%)	L'LCT/MLCT
	**S_8**	338.0	0.0466	H-4→LUMO (61%), H-4→L+1 (28%)	L'LCT/MLCT
	**S_9**	333.8	0.209	H-4→LUMO (26%), H-4→L+1 (60%)	L'LCT/MLCT

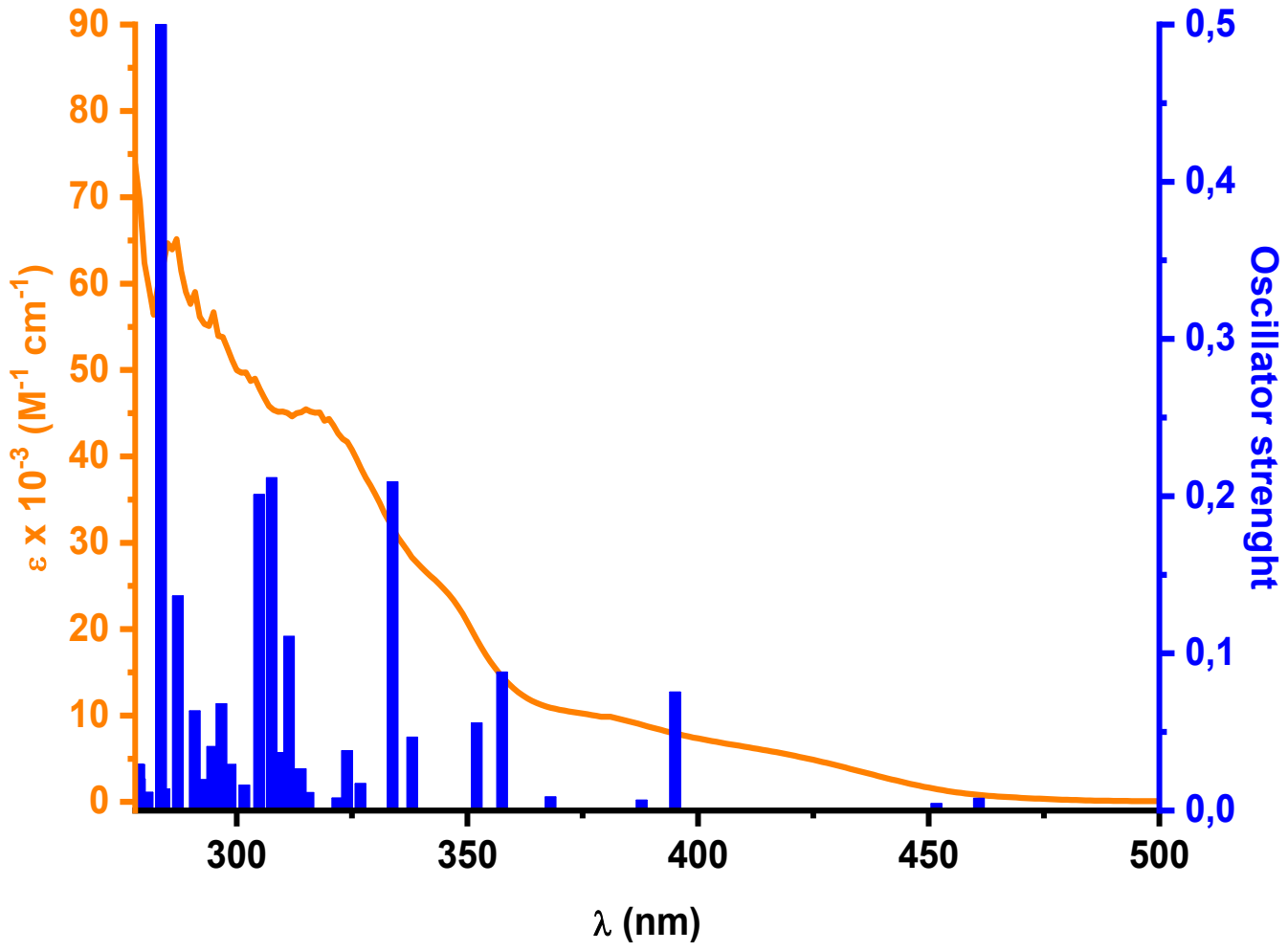

Figure S56. UV-Vis absorption experimental spectra (orange) and calculated transitions (blue) in CH_2Cl_2 ***N,N-trans*-2b**.

Table S33. Plots and composition (%) of the frontier MOs and spin density of the first triplet state in CH_2Cl_2 of ***N,N-trans*-2b**.

***N,N-trans*-2b**		
SOMO	SOMO-1	Spin density
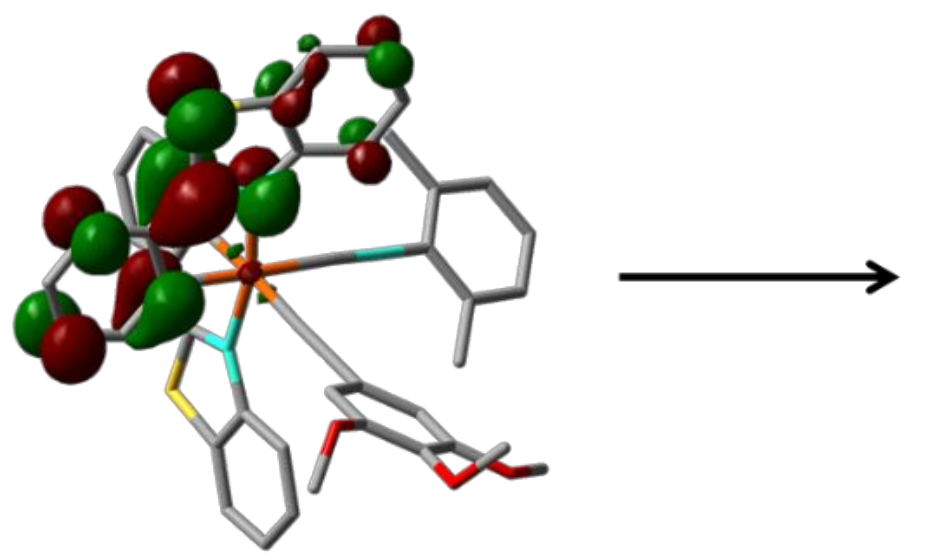	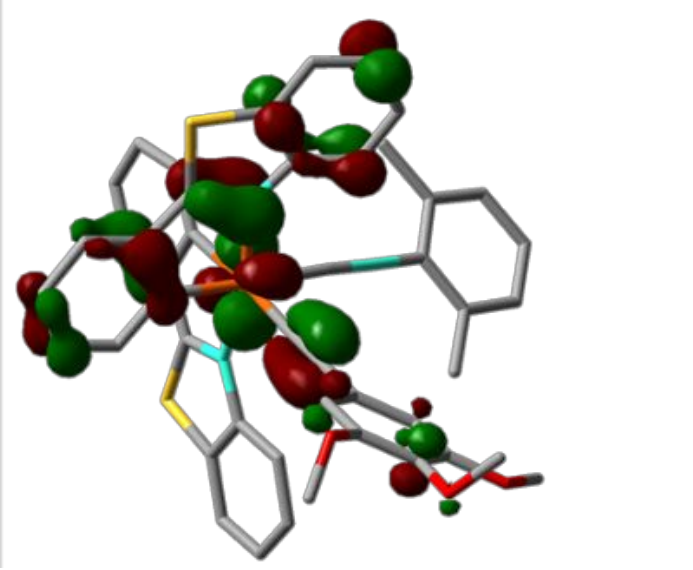	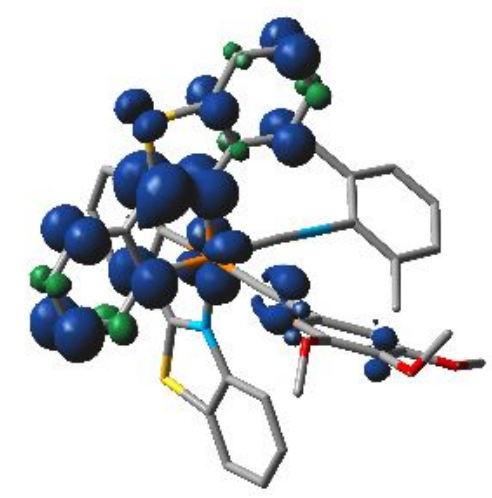
-3.27 eV<br>Ir 2 %, pbt(1) 96 %, pbt(2) 2 %, CNXyl 0 %, CC-$C_6H_2(OMe)_3$ 1 %	-4.16 eV<br>Ir 22 %, pbt(1) 55 %, pbt(2) 2 %, CNXyl 1 %, CC-$C_6H_2(OMe)_3$ 21 %	0.122 on Ir

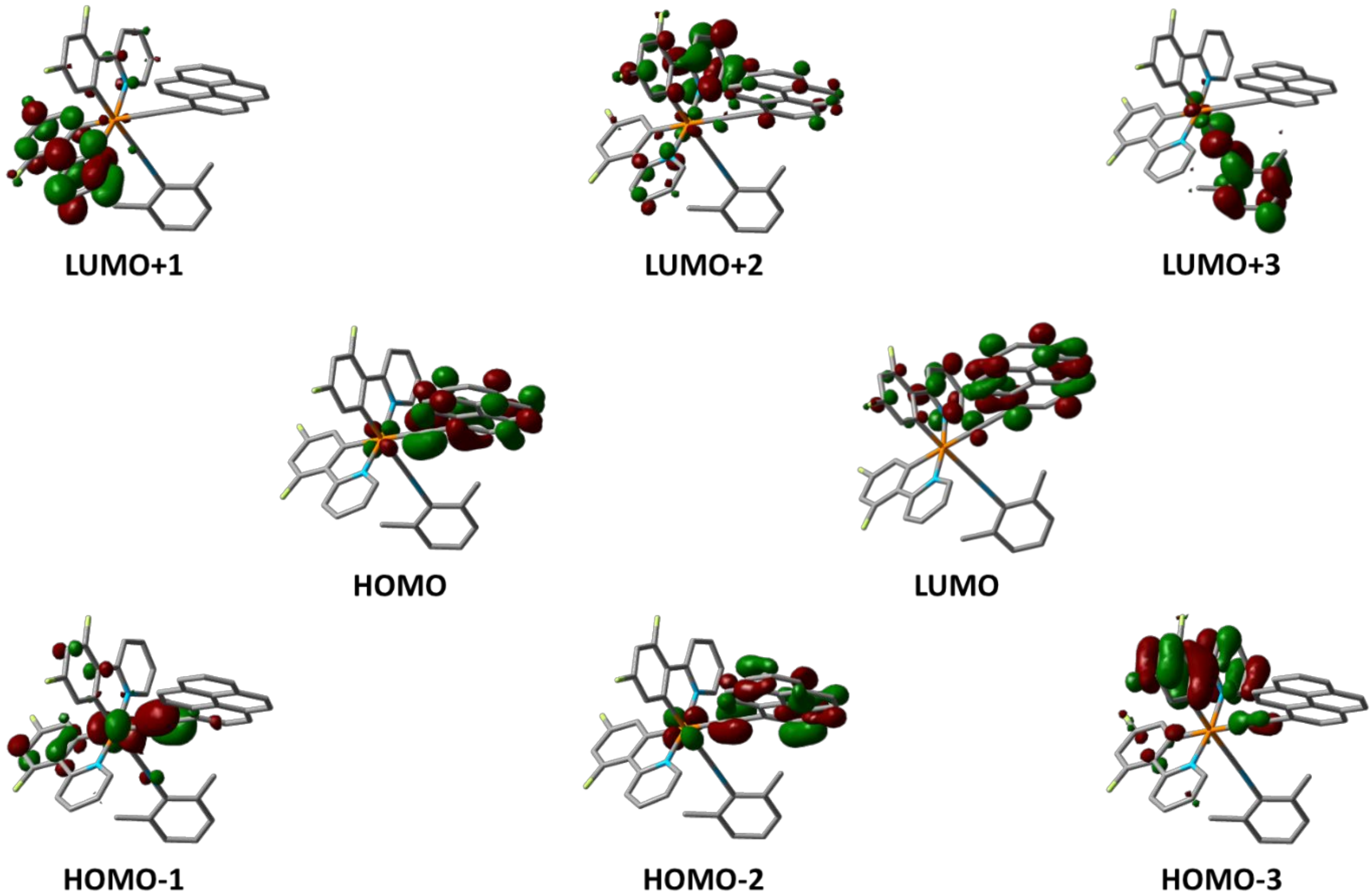

Figure S57. Selected frontier Molecular Orbitals for ***N,N-trans*-1c** in the ground state.

Table S34. Composition (%) of frontier MOs in terms of ligands and metals in the ground state for ***N,N-trans*-1c** in CH_2Cl_2.

***N,N-trans*-1c**							
**Orbital**	**Energy (eV)**	**Ir**	**dfppy(1)**	**dfppy(2)**	**CNXyl**	**CC**	**pyrene**
**LUMO+5**	-0.91	2	54	42	2	0	0
**LUMO+4**	-1.02	2	42	55	0	0	0
**LUMO+3**	-1.24	5	3	2	90	0	1
**LUMO+2**	-1.54	3	14	59	0	2	22
**LUMO+1**	-1.59	2	82	9	1	1	5
**LUMO**	-1.60	1	1	28	1	5	64
**HOMO**	-4.94	6	1	1	0	23	69
**HOMO-1**	-5.68	32	18	11	3	32	3
**HOMO-2**	-5.98	15	3	4	0	16	62
**HOMO-3**	-6.19	1	12	78	0	8	1
**HOMO-4**	-6.25	6	55	5	2	15	17
**HOMO-5**	-6.33	9	24	29	0	2	35

Table S35. Selected vertical excitation energies singlets (S_0) and first triplets computed by TD-DFT/SCRF (CH_2Cl_2) with the orbitals involved for complex ***N,N-trans*-1c**.

**Complex**	**State**	**λ/nm**	***f***	**Transition (% Contribution)**	
***N,N-trans*-1c**	**T_1**	620.0	-	HOMO→L+1 (15%), HOMO→L+2 (72%)	L’$_{pyr}$LCT
	**T_2**	445.0	-	HOMO→LUMO (38%), HOMO→L+1 (33%), HOMO→L+2 (15%)	L’$_{pyr}$LCT/MLCT/L’$_{C≡C}$LCT
	**T_3**	430.8	-	HOMO→LUMO (50%), HOMO→L+1 (40%)	L’$_{pyr}$LCT/MLCT/L’$_{C≡C}$LCT
	**S_1**	435.3	0.016	HOMO→LUMO (60%), HOMO→L+1 (33%)	L’$_{pyr}$LCT/MLCT/L’$_{C≡C}$LCT
	**S_2**	428.7	0.0044	HOMO→LUMO (36%), HOMO→L+1 (59%)	L’$_{pyr}$LCT/MLCT/L’$_{C≡C}$LCT
	**S_3**	392.8	0.6319	HOMO→L+2 (88%)	L’$_{pyr}$LCT
	**S_5**	365.6	0.0307	H-1→LUMO (74%), HOMO→L+3 (22%)	ML’$_{pyr}$CT/MLCT/LL’$_{pyr}$CT/L’$_{pyr}$L”CT
	**S_{11}**	321.6	0.0215	H-2→LUMO (79%)	L’$_{C≡C}$LCT/MLCT

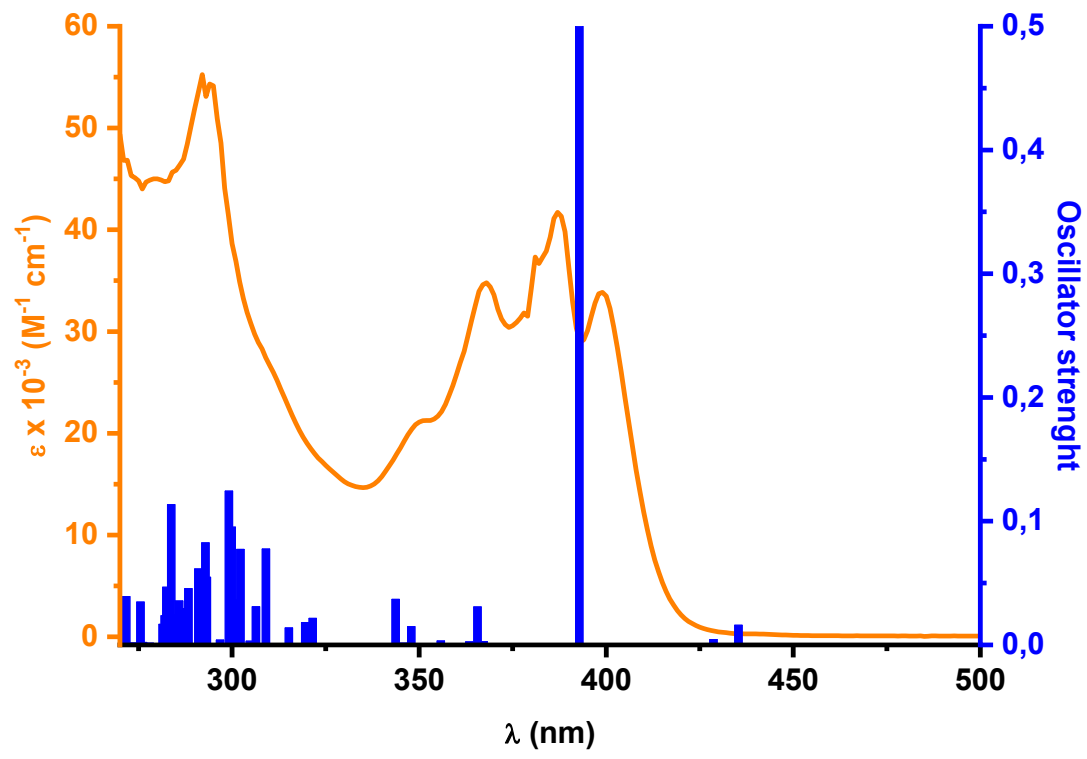

Figure S58. UV-Vis absorption experimental spectra (orange) and calculated transitions (blue) of ***N,N-trans*-1c** in CH_2Cl_2

Table S36. Plots and composition (%) of the frontier MOs and spin density of the first triplet state in CH_2Cl_2 of complex ***N,N-trans*-1c**.

***N,N-trans*-1c**		
SOMO	SOMO-1	Spin density
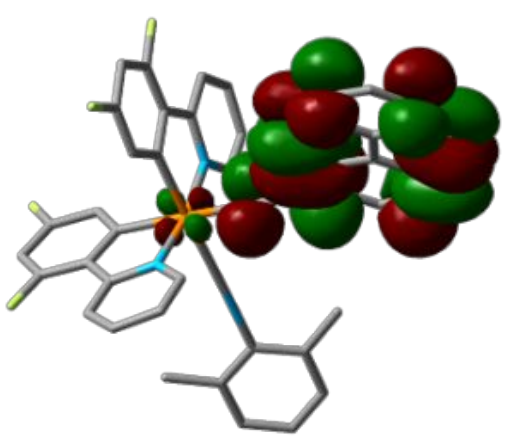	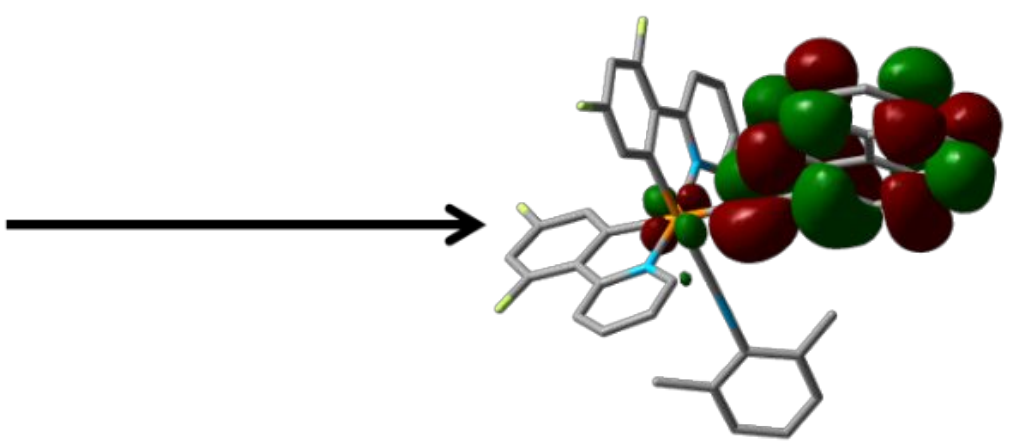	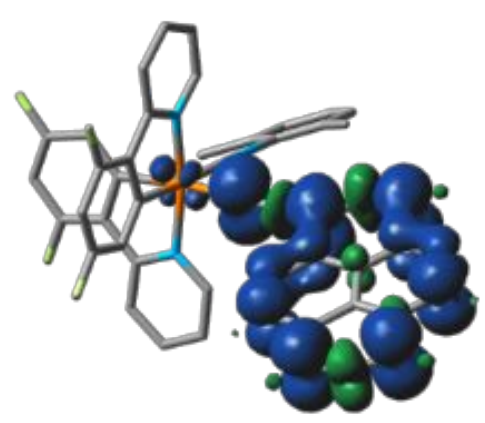
-3.12 eV<br>Ir 1 %, dfppy(1) 0 %, dfppy(2) 0 %, CNXyl 0 %, CC-pyrene 98 %	-3.42 eV<br>Ir 2 %, dfppy (1) 1 %, dfppy 2) 0 %, CNXyl 0 %, CC-pyrene 97 %	0.014 on Ir

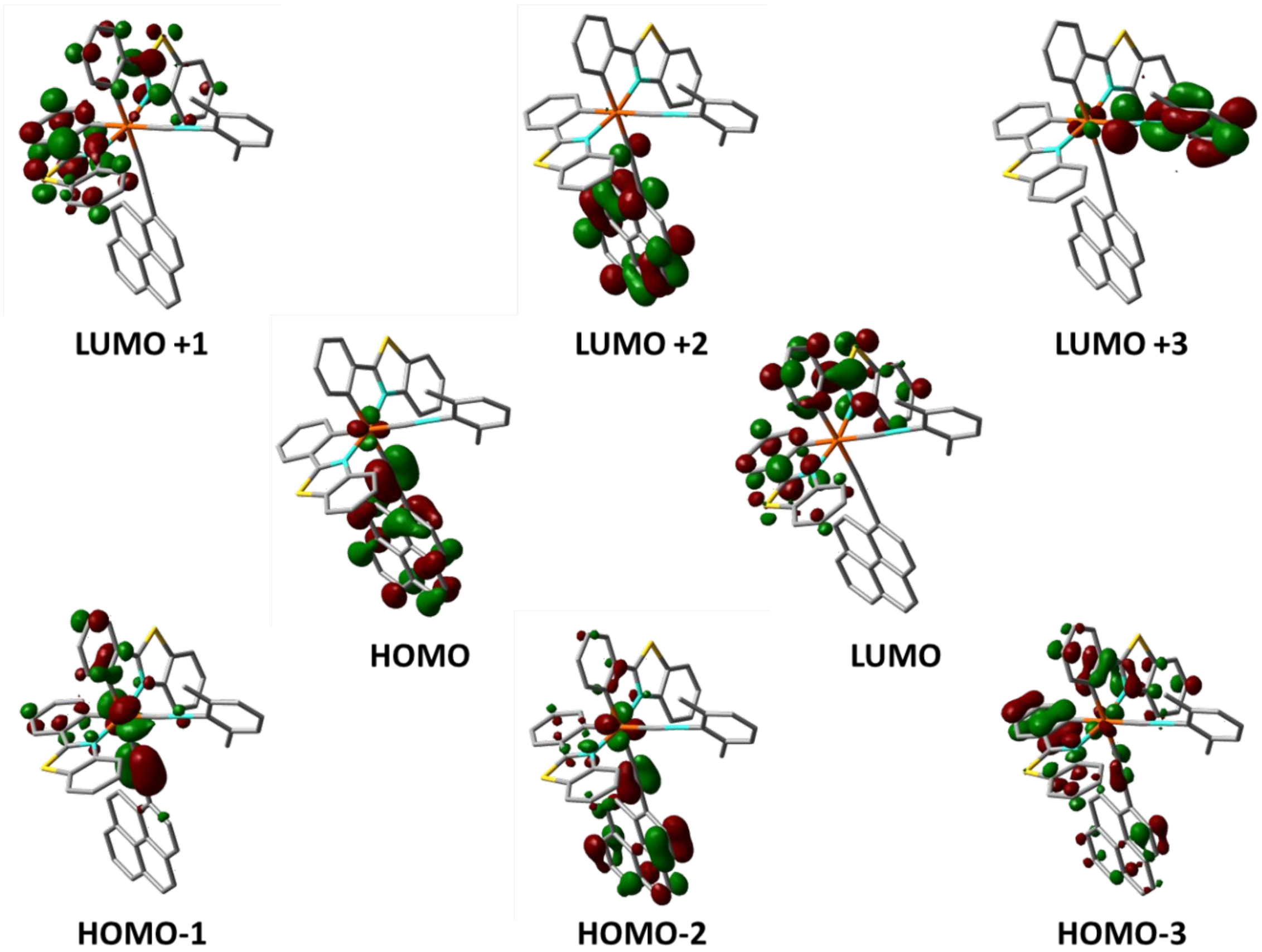

Figure S59. Selected frontier Molecular Orbitals for ***N,N-trans*-2c** in the ground state

Table S37. Composition (%) of Frontier MOs in terms of ligands and metals in the ground state for ***N,N-trans*-2c** in CH_2Cl_2.

***N,N-trans*-2c**							
**Orbital**	**Energy (eV)**	**Ir**	**pbt(1)**	**pbt(2)**	**CNXyl**	**CC**	**pyrene**
**LUMO+5**	-0.37	1	97	2	1	0	0
**LUMO+4**	-0.40	1	0	3	0	1	95
**LUMO+3**	-0.82	4	3	1	92	0	0
**LUMO+2**	-1.40	1	0	2	0	3	95
**LUMO+1**	-1.63	2	68	28	1	0	1
**LUMO**	-1.75	1	29	69	0	0	1
**HOMO**	-5.04	6	1	2	0	18	72
**HOMO-1**	-5.53	35	26	20	3	13	3
**HOMO-2**	-5.81	22	19	26	1	8	25
**HOMO-3**	-6.02	15	12	39	5	7	23
**HOMO-4**	-6.17	12	53	12	6	12	5
**HOMO-5**	-6.21	8	27	5	1	12	47

Table S38. Selected vertical excitation energies singlets (S_0) and first triplets computed by TD-DFT/SCRF (CH_2Cl_2) with the orbitals involved of complex ***N,N-trans*-2c**.

**Complex**	**State**	**λ/nm**	f	Transition (% Contribution)	
***N,N-trans*-2c**	**T_1**	582.3	-	HOMO→L+2 (84%)	IL'$_{pyr}$
	**T_2**	472.5	-	H-3→LUMO (15%), H-2→LUMO (16%), H-1→LUMO (28%), HOMO→LUMO (13%)	L'$_{pyr}$LCT/ MLCT/ L'$_{C≡C}$LCT
	**T_3**	467.7	-	H-4→L+1 (12%), H-2→LUMO (12%), H-2→L+1 (14%), H-1→LUMO (13%), H-1→L+1 (29%)	L'$_{pyr}$LCT/ MLCT/ L'$_{C≡C}$LCT
	**S_1**	438.6	0.0136	HOMO→LUMO (95%)	L'$_{pyr}$LCT
	**S_2**	421.9	0.0063	HOMO→L+1 (96%)	L'$_{pyr}$LCT
	**S_3**	403.3	0.0566	H-1→LUMO (96%)	L'$_{C≡C}$LCT/ MLCT
	**S_5**	381.0	0.5025	HOMO→L+2 (93%)	IL'$_{pyr}$/ ML'$_{pyr}$CT
	**S_6**	355.4	0.0733	H-2→LUMO (90%)	L'$_{pyr}$LCT/ MLCT
	**S_7**	343.6	0.0502	H-2→L+1 (84%)	L'$_{pyr}$LCT/ MLCT
	**S_9**	334.6	0.0685	H-5→LUMO (11%), H-3→LUMO (78%)	L'$_{pyr}$LCT/ MLCT/ LLCT

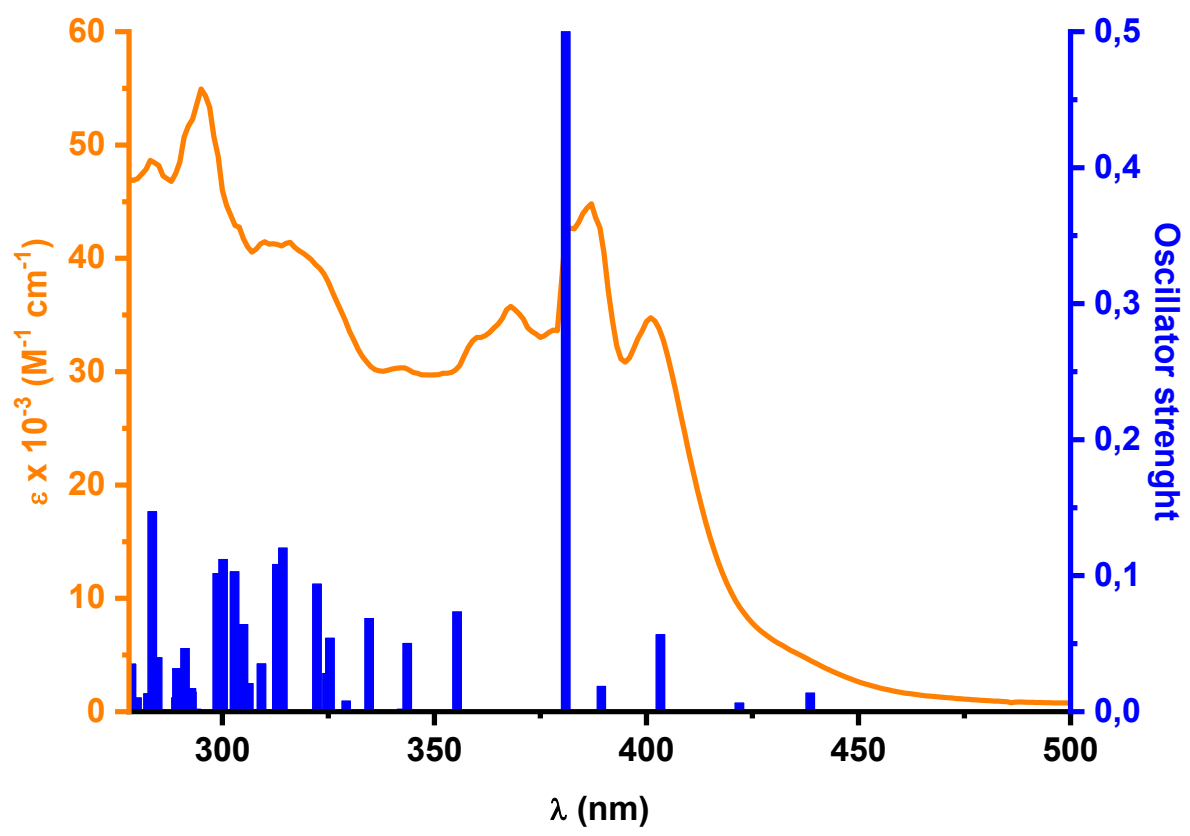

Figure S60. UV-Vis absorption experimental spectra (orange) and calculated transitions (blue) in CH_2Cl_2 ***N,N-trans*-2c**.

Table S39. Plots and composition (%) of the frontier MOs and spin density of the first triplet state in CH_2Cl_2 of complex ***N,N-trans*-2c**.

***N,N-trans*-2c**		
SOMO	SOMO-1	Spin density
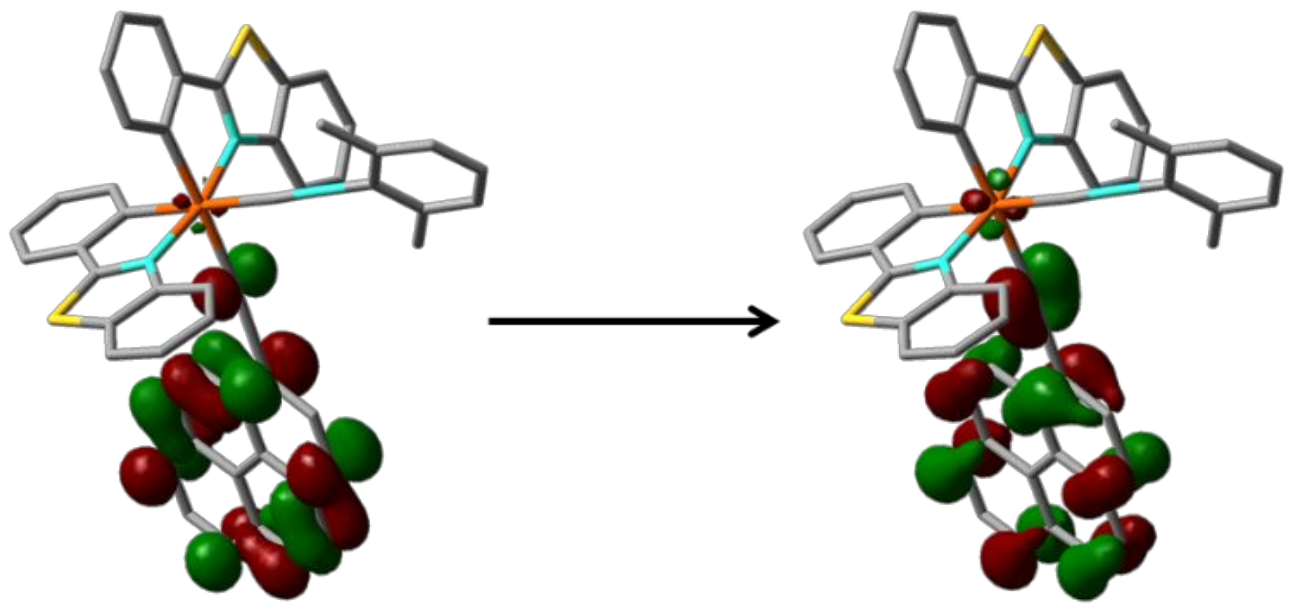		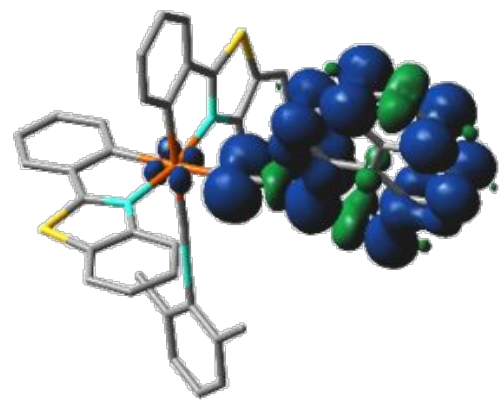
-3.08 eV<br>Ir 1 %, pbt(1) 0 %, pbt(2) 0 %, CNXyl 0 %, CC-pyrene 98 %	-3.38 eV<br>Ir 3 %, pbt(1) 0 %, pbt(2) 1 %, CNXyl 0 %, CC-pyrene 97 %	0.015 on Ir

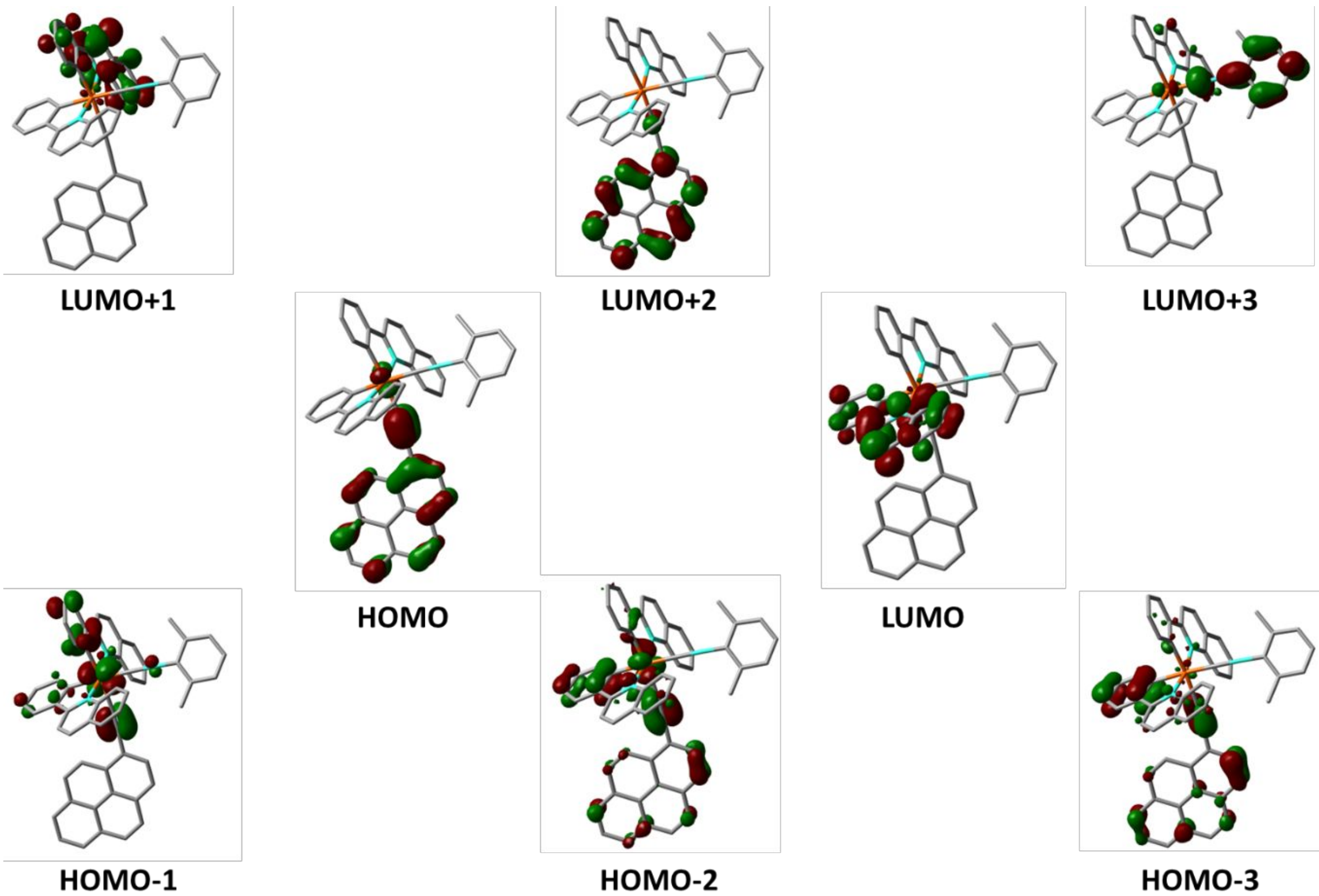

Figure S61. Selected frontier Molecular Orbitals for ***N,N-trans*-3c** in the ground state in CH_2Cl_2.

Table S40. Composition (%) of frontier MOs in terms of ligands and metals in the ground state for ***N,N-trans*-3c** in CH_2Cl_2.

***N,N-trans*-3c**							
**Orbital**	**Energy (eV)**	**Ir**	**pq(1)**	**pq(2)**	**CNR**	CC	pyrene
**LUMO+5**	-0.93	1	31	66	3	0	0
**LUMO+4**	-1.03	1	66	24	9	0	0
**LUMO+3**	-1.13	3	6	11	80	0	0
**LUMO+2**	-1.54	1	0	0	0	7	92
**LUMO+1**	-1.97	2	0	97	0	0	0
**LUMO**	-2.01	1	96	1	2	0	0
**HOMO**	-4.85	8	1	2	0	24	66
**HOMO-1**	-5.49	32	14	31	6	15	3
**HOMO-2**	-5.86	17	30	8	1	15	29
**HOMO-3**	-5.98	2	37	9	0	14	38
**HOMO-4**	-6.07	15	4	45	6	28	3
**HOMO-5**	-6.26	11	44	9	7	2	27

Table S41. Selected vertical excitation energies singlets (S_0) and first triplets computed by TD-DFT/SCRF (CH_2Cl_2) with the orbitals involved of complex ***N,N-trans*-3c**.

**Complex**	**State**	λ/nm	f	Transition (% Contribution)	
***N,N-trans*-3c**	$\mathbf{T_1}$	664.0	-	HOMO→L+2 (90%)	IL'$_{pyr}$/ IL'$_{pyr}$CT/
	$\mathbf{T_2}$	542.5	-	HOMO→LUMO (90%)	L'$_{pyr}$LCT/ MLCT
	$\mathbf{T_3}$	519.7	-	HOMO→L+1 (82%)	L'$_{pyr}$LCT/ MLCT
	$\mathbf{S_1}$	535.0	0.001	HOMO →LUMO (98%)	L'$_{pyr}$LCT/ MLCT
	$\mathbf{S_2}$	512.9	0.004	HOMO→L+1 (97%)	L'$_{pyr}$LCT/ MLCT/
	$\mathbf{S_3}$	438.6	0.021	H-1→LUMO (98%)	L'$_{pyr}$LCT/ MLCT/ LLCT
	$\mathbf{S_4}$	430.6	0.046	H-1→L+1 (97%)	L'$_{pyr}$LCT/ MLCT/ LLCT
	$\mathbf{S_5}$	410.6	0.761	HOMO→L+2 (94%)	IL'$_{pyr}$/ ML'$_{pyr}$CT
	$\mathbf{S_{11}}$	361.5	0.010	H-4→LUMO (78%)	L'$_{pyr}$LCT/IL CT/MLCT
	$\mathbf{S_{12}}$	357.7	0.053	H-4→LUMO (10%), H-3→LUMO (63%), HOMO→L+5 (17%)	L'$_{pyr}$LCT//IL CT/MLCT

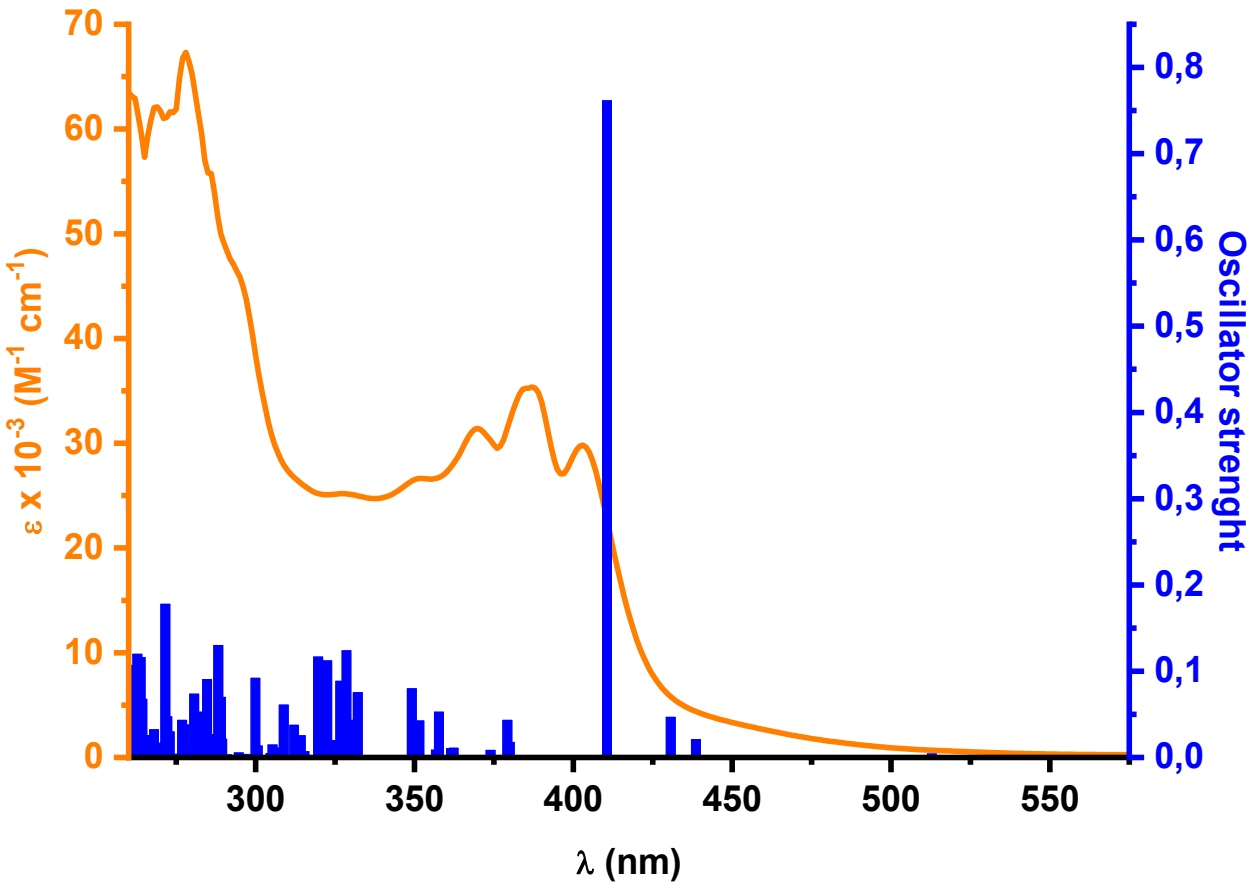

Figure S62. UV-Vis absorption experimental spectra (orange) and calculated transitions (blue) in CH_2Cl_2 ***N,N-trans*-3c.**

Table S42. Plots and composition (%) of the frontier MOs and spin density of the first triplet state in CH_2Cl_2 of complex ***N,N-trans*-3c**.

***N,N-trans*-3c**		
SOMO	SOMO-1	Spin density
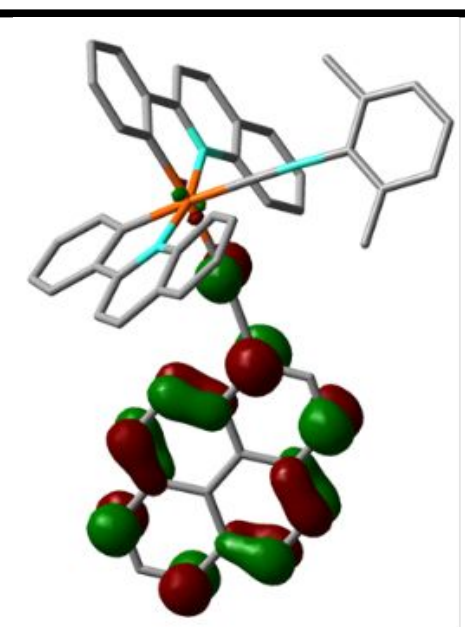	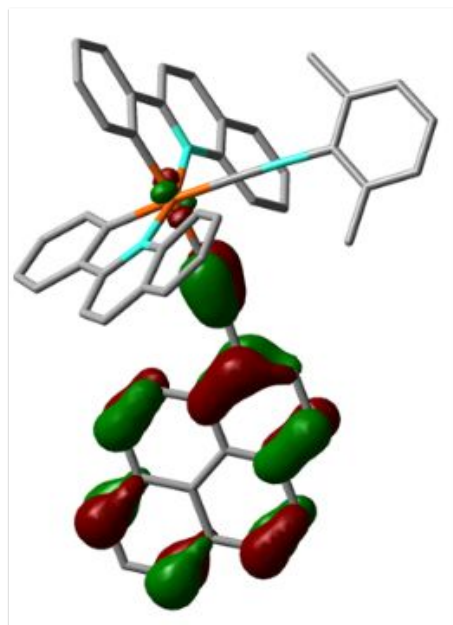	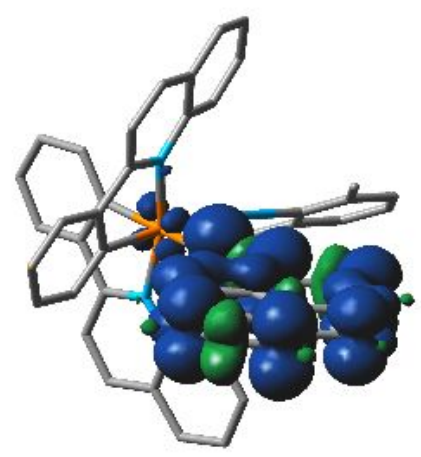
-3.05 eV<br>Ir 2 %, pq(1) 0 %, pq(2) 0 %, CNXyl 0 %, CC-pyrene 98 %	-3.35 eV<br>Ir 3 %, pq(1) 0 %, pq(2) 1 %, CNXyl 0 %, CC-pyrene 96 %	0.016 on Ir

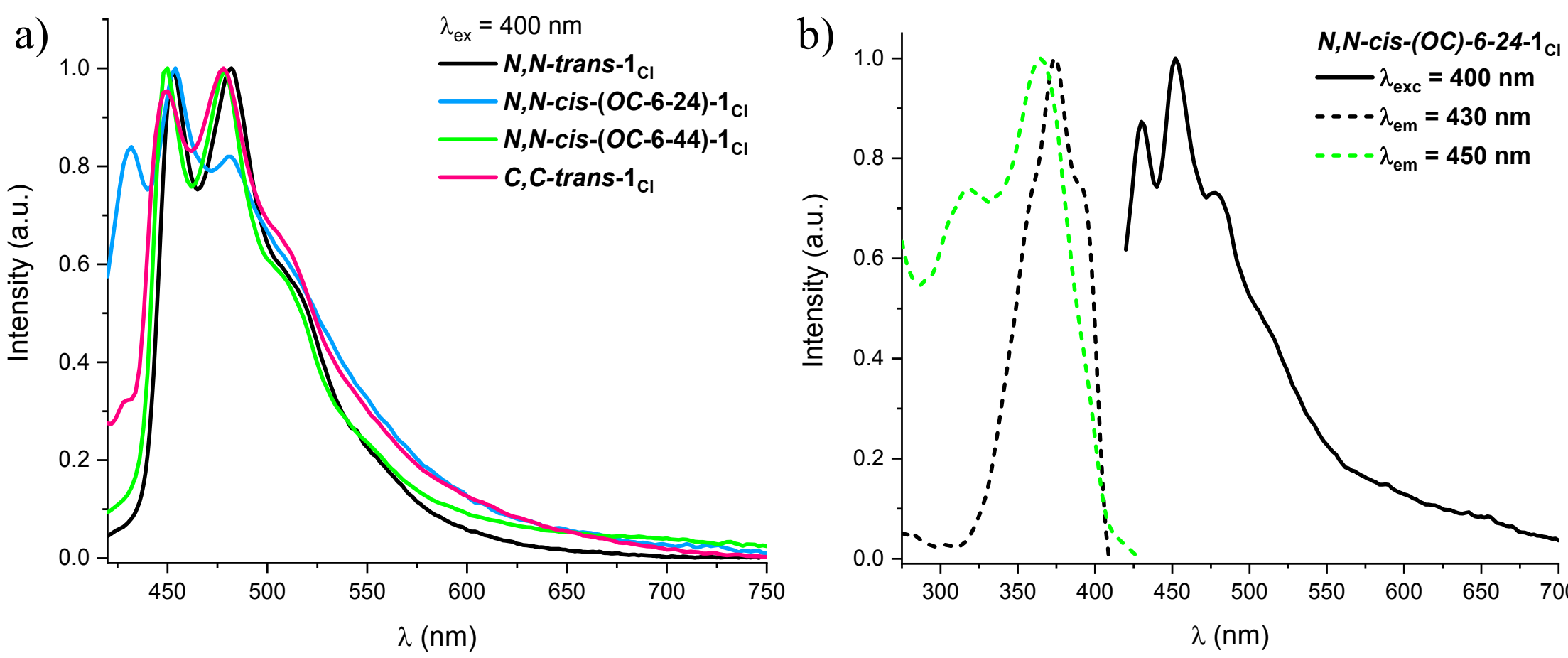

Figure S63. Emission spectra of a) ***N,N-trans*-1$_{Cl}$**, ***N,N-cis*-(*OC*-6-24)-1$_{Cl}$**, ***N,N-cis*-(*OC*-6-44)-1$_{Cl}$**, in CH_2Cl_2 (5 x 10^{-5} M) solution at 298 K and ***C,C-trans*-1$_{Cl}$** in CH_3CN (5 x 10^{-5} M) and b) excitation and emission spectra of ***N,N-cis*-(*OC*-6-24)-1$_{Cl}$** under the same experimental conditions.

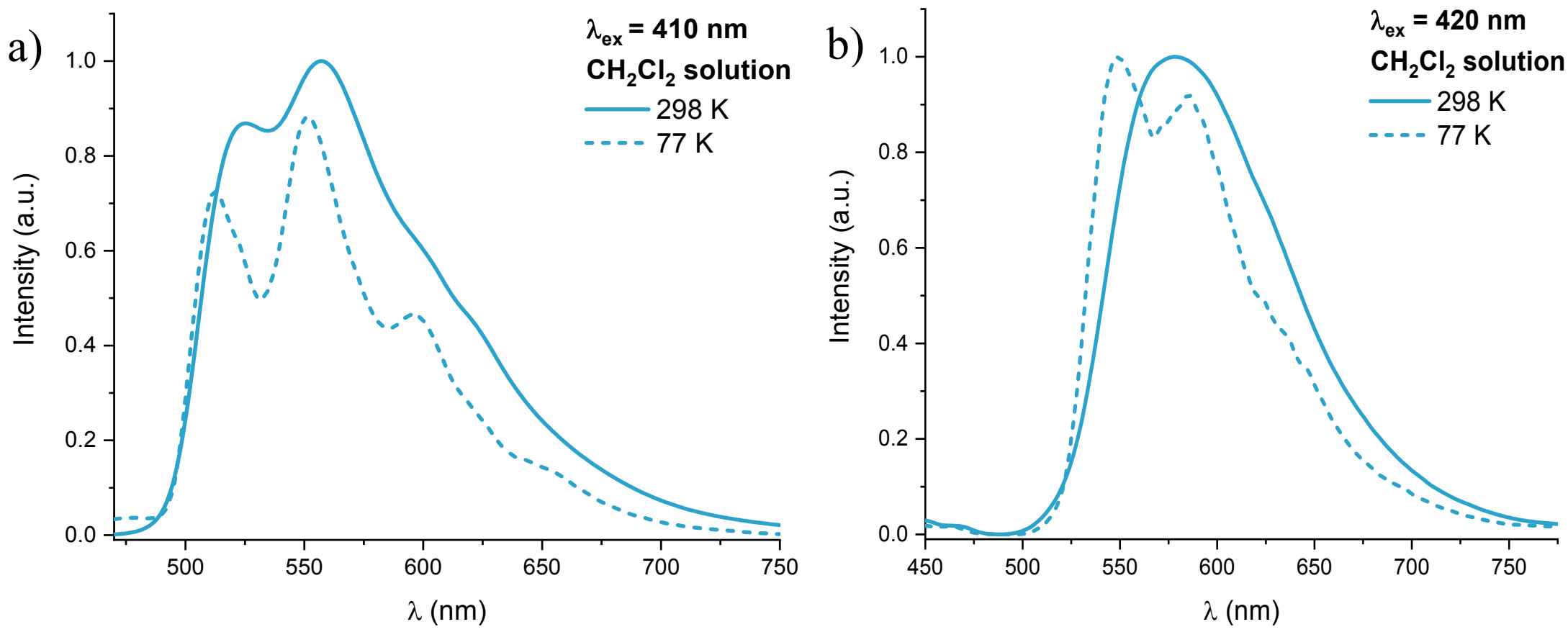

Figure S64. Emission spectra of complexes a) ***N,N-trans*-2**$_{Cl}$ and b) ***N,N-trans*-3**$_{Cl}$ in CH_2Cl_2 (5 x 10^{-5} M) solution at 298 K, 77 K and PS 1% of complexes.

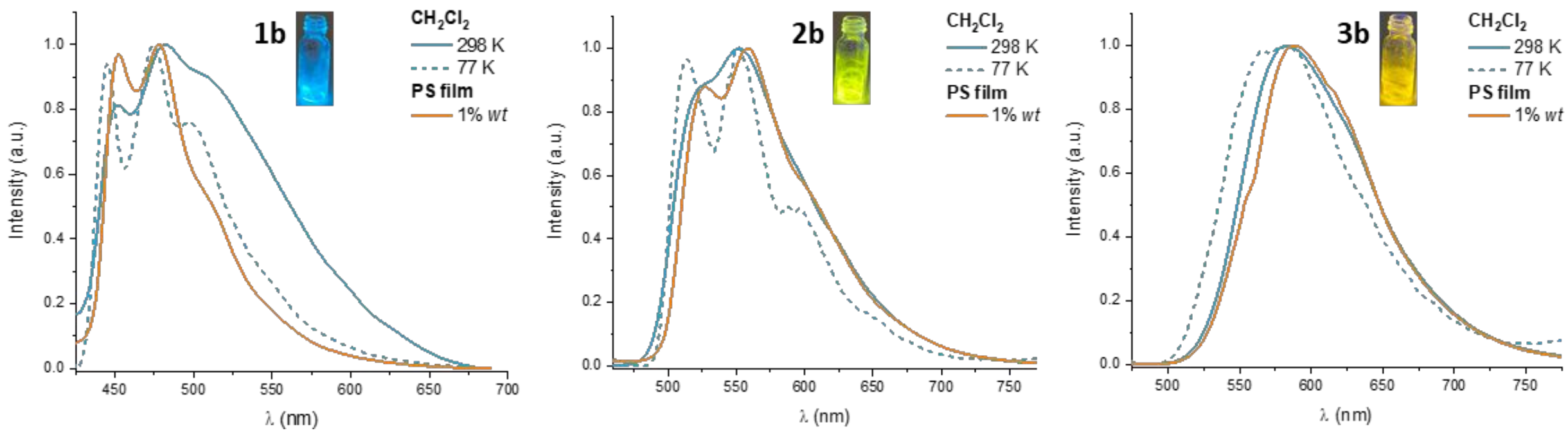

Figure S65. Emission spectra in CH_2Cl_2 solution (λ_{ex} = 400-420 nm) at 298 K, 77 K and PS 1% of ***N,N-trans***- complexes **1b-3b.**

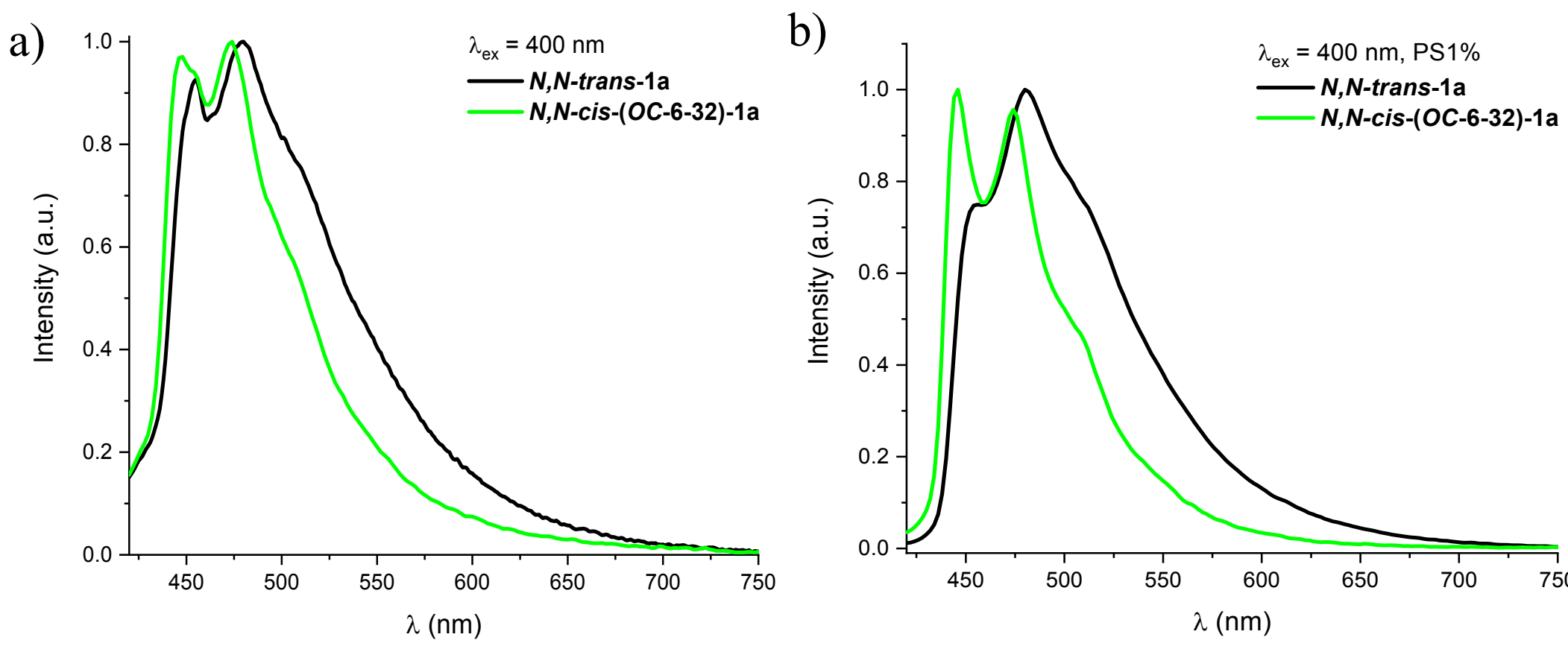

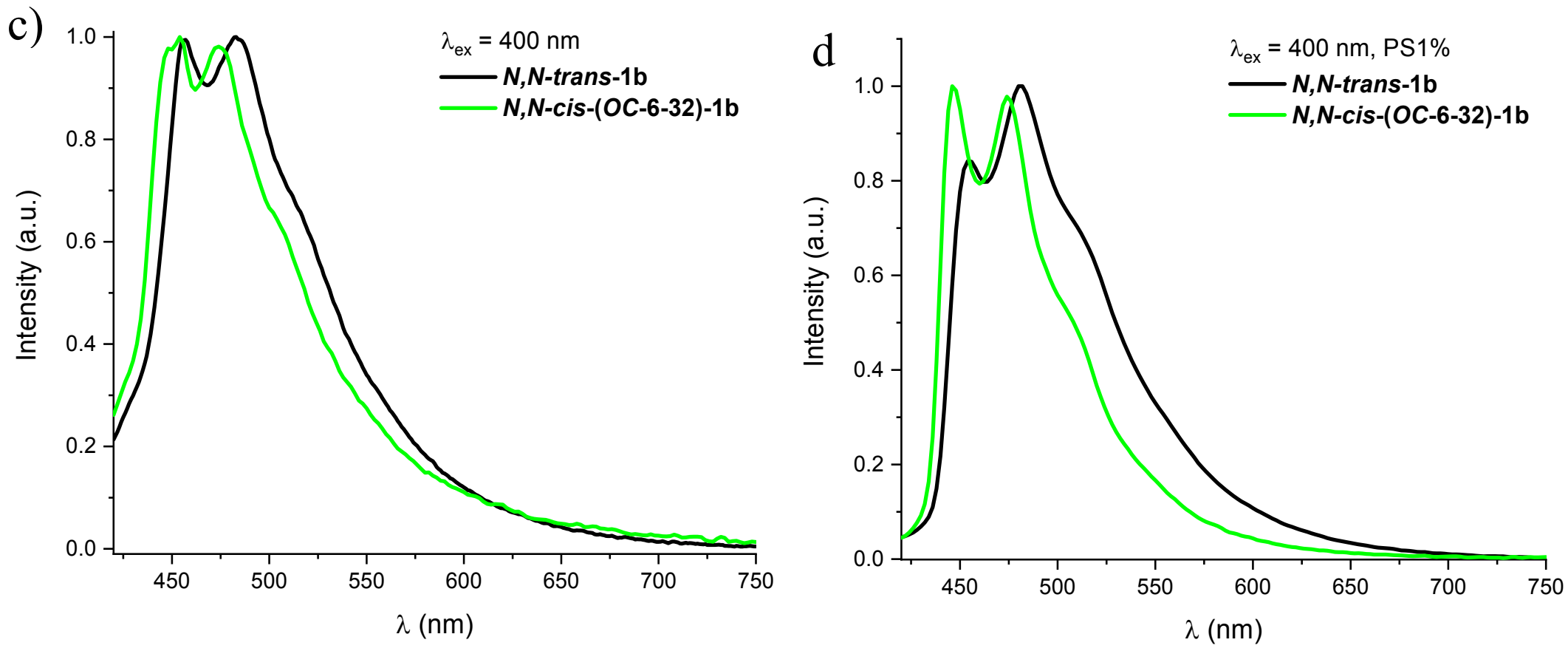

Figure S66. Emission spectra of complexes ***N,N-trans*-1a/b**, ***N,N-cis*-(*OC*-6-43)-1a/b** and ***N,N-cis*-(*OC*-6-32)-1a/b**, in a) and c) deaerated CH_2Cl_2 solution (5 x 10^{-5} M) at 298 K, and b) and d) film PS1% respectively.

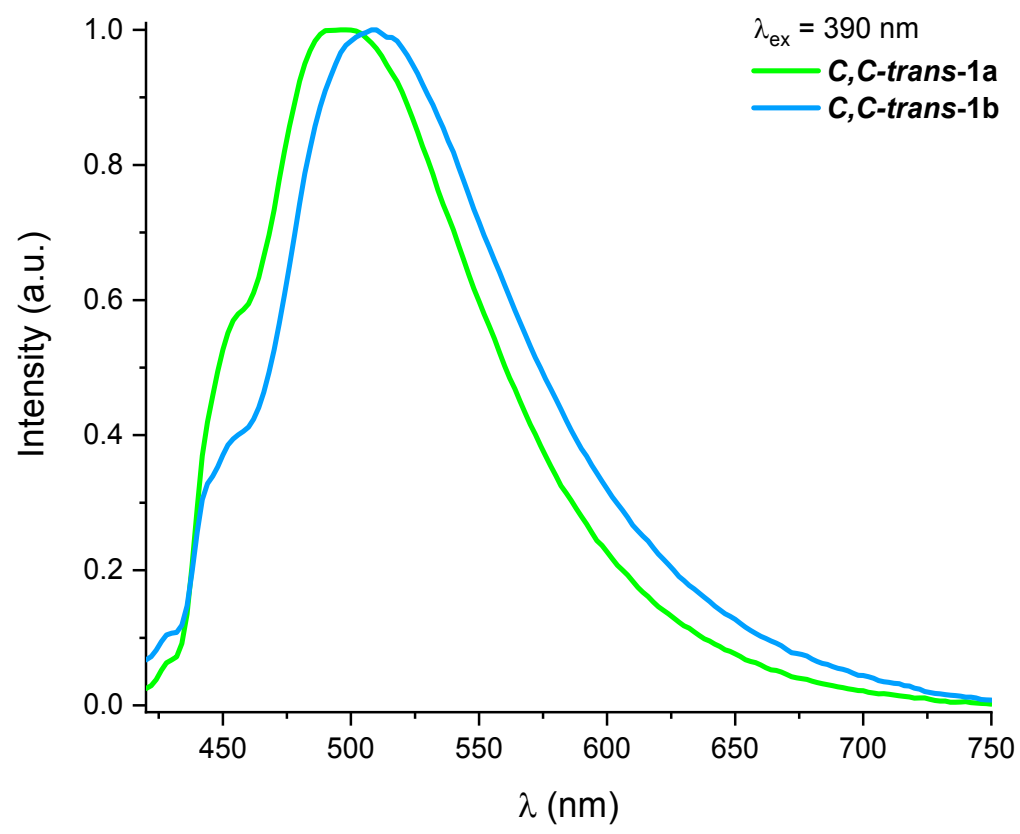

Figure S67. Emission spectra of photoisomers ***C,C-trans*-1$_{Cl}$**, ***C,C-trans*-1a** and ***C,C-trans*-1b** in deaerated CH_3CN (5 x 10^{-5} M).

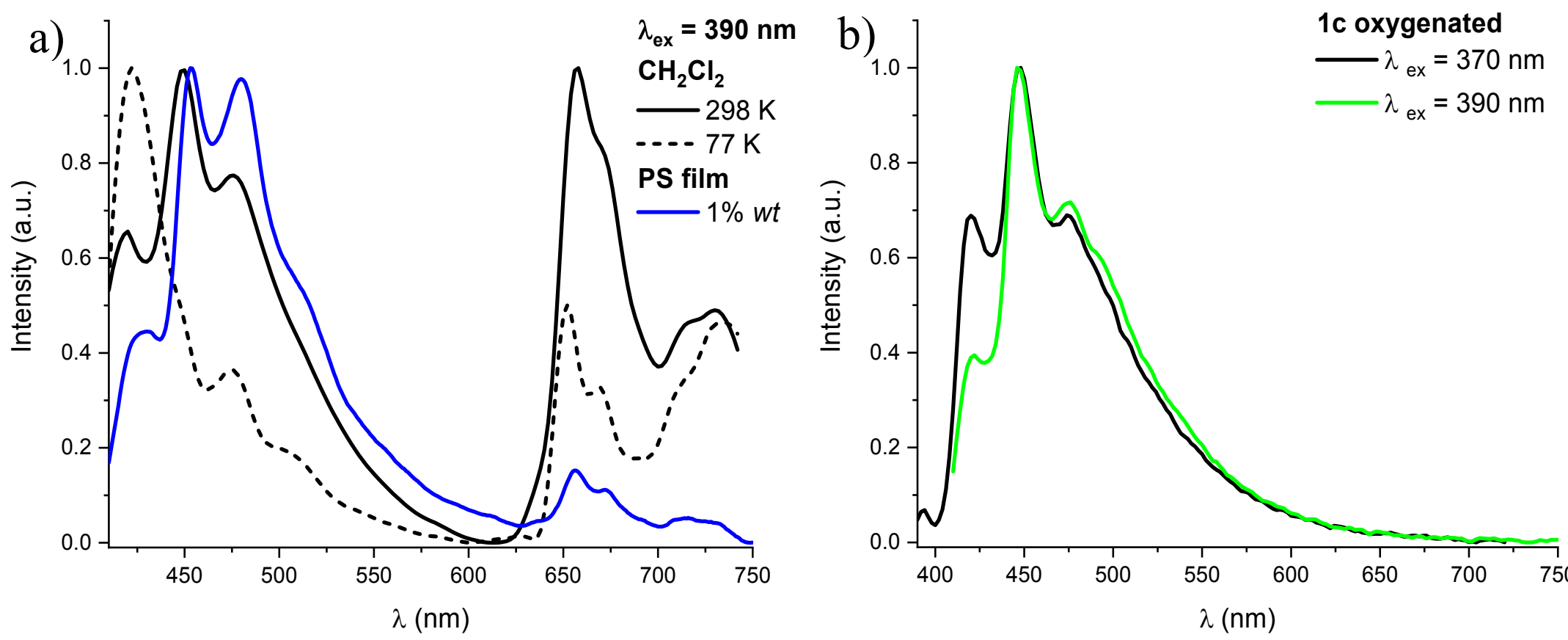

Figure S68. Emission spectra of **1c** in a) in deaerated CH_2Cl_2 solution at 298 K, 77 K and in PS 1% *wt* and b) oxygenated CH_2Cl_2 solution at 298K.

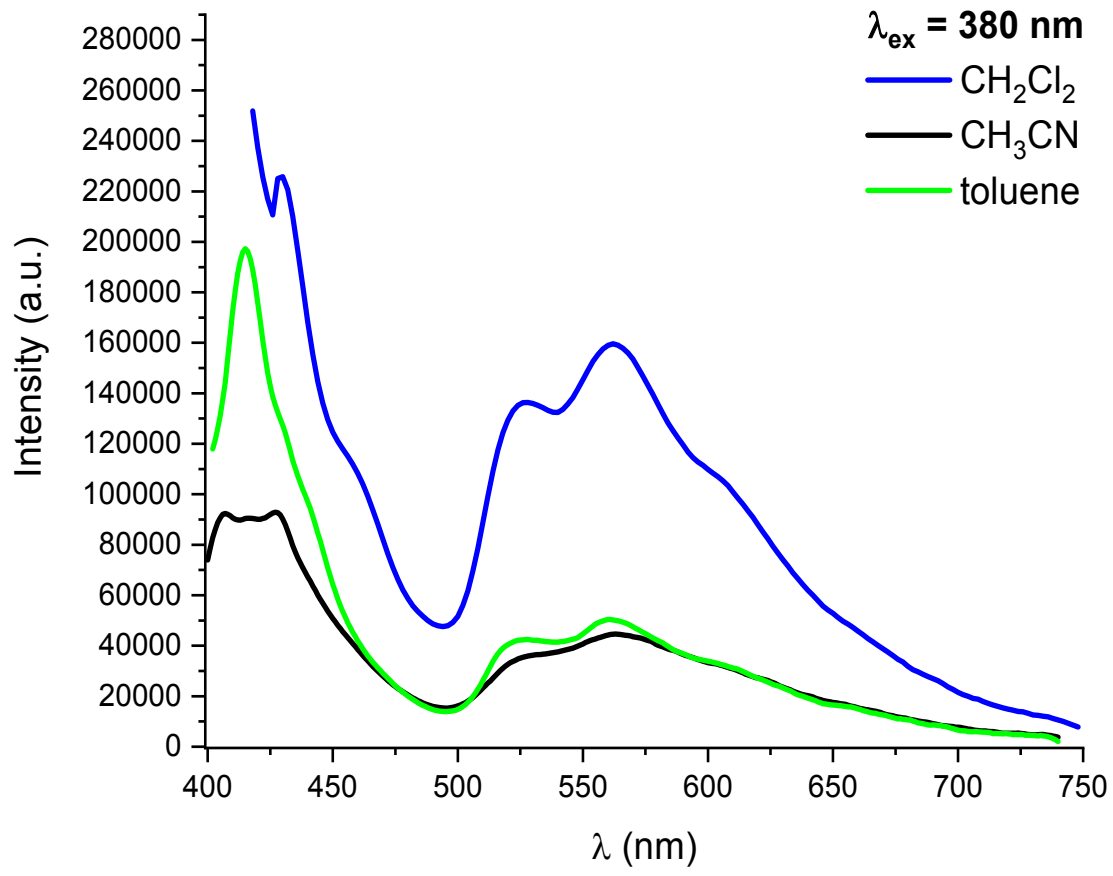

Figure S69. Emission spectra of complex ***N,N-trans*-2c** in different deaerated solvents at 298 K**.**

5. Cyclic voltammograms

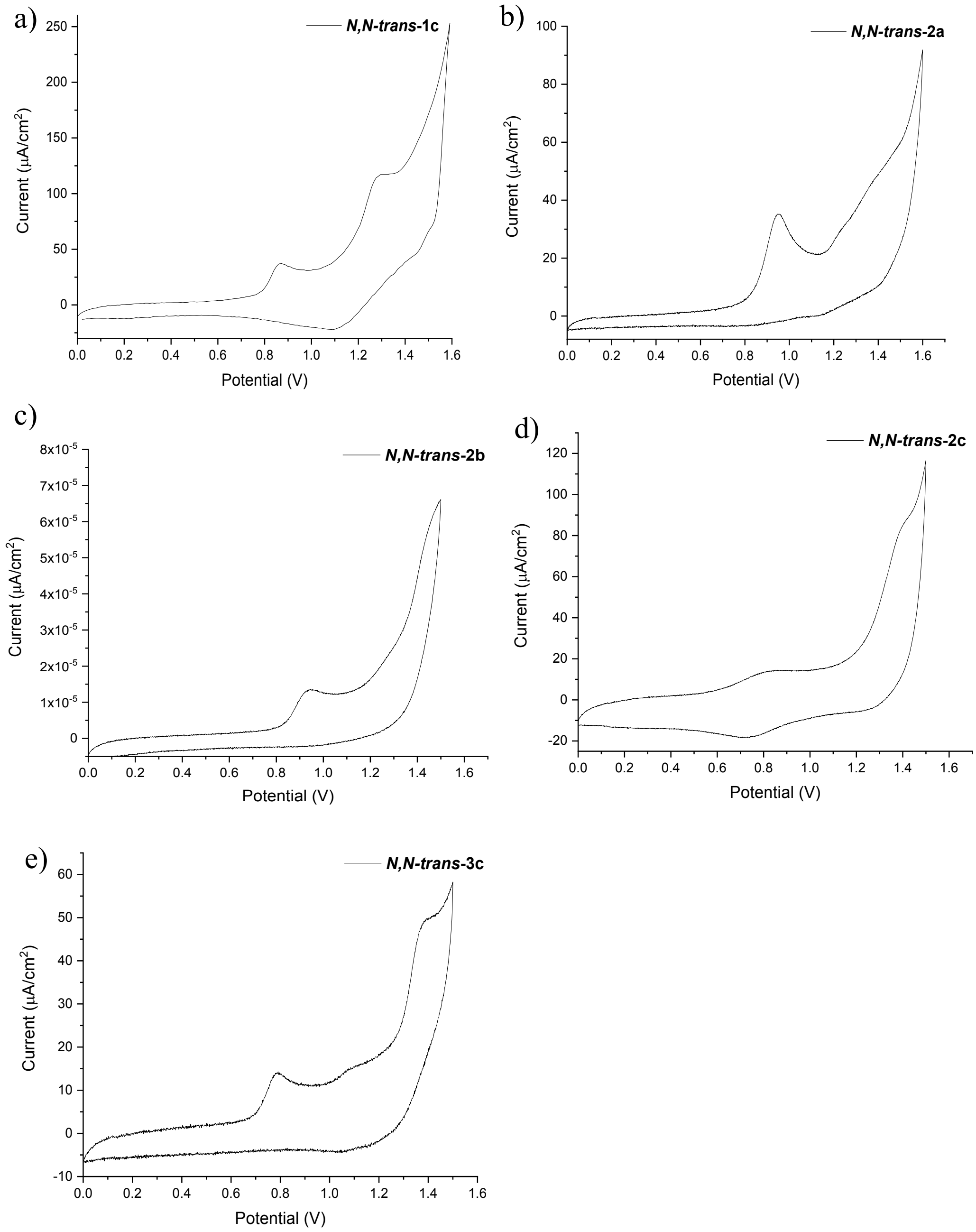

Figure S70. a) Cyclic voltammograms of a) ***N,N-trans*-1c**, b) ***N,N-trans*-2a**, c) ***N,N-trans*-2b**, d) ***N,N-trans*-2c** and e) ***N,N-trans*-3c** in CH_2Cl_2 solution at 100 mVs^{-1}.
